# Sodium aescinate protects renal ischemia-reperfusion and pyroptosis through AKT/NLRP3 signaling pathway

**DOI:** 10.1080/0886022X.2025.2488140

**Published:** 2025-04-22

**Authors:** Liu Xin, Ning Kanghao, Li Jiacheng, Yan Xiaodong, Yan Juhan, Zhao Xinyang, Li Xiangdong

**Affiliations:** a The First Affiliated Hospital of Hebei North University, Hebei Province, China; b Graduate School of Hebei North University, Hebei Province, China

**Keywords:** Kidney ischemia-reperfusion injury, pyroptosis, sodium aescinoside, AKT/NLRP3 signaling

## Abstract

Renal ischemia-reperfusion injury (RIRI) is a common cause of acute renal injury. Studies have shown that sodium aescinate (SA) may serve as a potential therapeutic agent, although its exact mechanism remains unclear. This study first evaluated the efficacy of SA using a mouse renal ischemia-reperfusion model. Subsequently, its mechanism was elucidated through systematic bioinformatics, and finally validated through *in vitro* and *in vivo* experiments. The results demonstrated that SA has a protective effect on renal function in mice with RIRI. Bioinformatic analysis indicated that the pyroptosis pathway is significantly activated during renal ischemia-reperfusion injury, and immunohistochemistry showed that the level of renal pyroptosis is upregulated during ischemia-reperfusion injury. Administration of SA was able to reduce the expression of pyroptosis-related proteins (GSDMD, NLRP3, IL-1β) in RIRI. *In vitro* and *in vivo* experiments further confirmed that SA exerts an anti-pyroptotic effect by inhibiting the AKT/NLRP3 signaling pathway. Ultimately, SA mitigates kidney injury in IRI mice by suppressing renal failure through inhibition of the AKT/NLRP3 signaling pathway.

## 1. Introduction

Renal ischemia-reperfusion injury (RIRI) is common in various types of shock, acute renal artery occlusion, or kidney transplantation, and is the most common cause of acute kidney injury in clinical practice [1–3]. The severity of ischemia-reperfusion is closely related to ischemic time, functional structure, metabolic characteristics of tissues and organs, and reperfusion conditions. The kidney is one of the organs vulnerable to ischemia-reperfusion injury due to its high metabolic demand and relatively little collateral circulation [3,4]. The pathogenesis of RIRI is complex, involving a variety of pathophysiological processes, including oxidative stress, inflammatory response, intracellular calcium overload, renin-angiotensin activation, and microcirculation disorders [5]. These mechanisms interact with each other, leading to renal tubular epithelial cell damage, decreased glomerular filtration function and finally may cause acute tubular necrosis, leading to acute renal failure. Although the mechanism of RIRI has been well understood, effective prevention and treatment methods are still limited.

Elevated levels of inflammatory factors are strong predictors of ischemia-reperfusion injury (IRI) risk [6,7]. Pyroptosis, a highly inflammatory caspase-dependent form of cell death mediated by Gasdermin-D (GSDMD) and the caspase family, is characterized by the disruption of the cell membrane barrier and the release of inflammatory cytokines [8]. Renal tubular epithelial cells, the most severely injured cells in IRI, are the principal cell type responsible for tubulointerstitial inflammation and pyroptosis. Pyroptosis of these cells can initiate a cascade of inflammatory responses [9,10]. Elevated levels of inflammatory proteins can activate the pyroptosis-related NOD-like receptor pyrin domain-containing 3 (NLRP3) inflammasome. Activation of the NLRP3 inflammasome promotes the secretion of proinflammatory factors IL-1β and IL-18, further exacerbating inflammation and causing renal tubular cell apoptosis and damage [11,12].

Sodium aescinate (SA), a natural mixture of triterpene saponins containing ester bonds, is commonly used in clinical settings to treat post-traumatic or postoperative edema, lymphedema, chronic venous insufficiency, hemorrhoids, ischemic reperfusion, and other conditions [13–15]. Studies have reported that SA maintains tissue and blood vessel homeostasis by increasing plasma ACTH concentrations [15,16]. Additionally, the role of SA in preventing tourniquet-induced IRI has garnered considerable research interest. However, its therapeutic effect in RIRI remains unexplored [14]. This study aims to evaluate the effects of SA on NLRP3 inflammasome activation, AKT expression, and inflammatory cytokine release in RIRI.

## 2. Materials and methods

### 2.1. Experimental drugs

SA (CAS: S31432) was purchased from Shanghai Yuanye Bio-Technology Co, Ltd with a purity of 95%.

### 2.2. Experimental animals

Twenty-eight Specific Pathogen Free male C57BL/6 mice, weighing 20 ± 2 g, were purchased from Spiff (Suzhou) Biotechnology Co, LTD and housed in Hebei North University at an ambient temperature of 23 ± 1 °C and relative humidity 50-65%, diet AD libitum; We anesthetized mice with pentobarbital sodium (60 mg/kg). Experimental animals were given humanitarian care according to the 3 R principles and approved by the Hebei North University committee.

### 2.3. Experimental model

According to previously published literature by the research group, 28 male mice were randomly divided into Sham group, model group, low-dose SA treatment group (2.5 mg/kg), SA treatment group high dose (5 mg/kg). Three days before surgery, the treatment group was intraperitoneally injected with saline containing SA, and the other two groups were intraperitoneally injected with the same amount of saline.

SA was injected intraperitoneally 45 min before surgery, and then anesthetized by intraperitoneal injection of sodium pentobarbital and placed on a thermostatic pad at 37 °C. The kidneys were exposed through two small incisions in the back, after the renal pedicle was found, the left and right renal pedicle were quickly blocked with a non-damaging mini-artery clamp. The kidney changed from bright red to purplish black, indicating that the clamp was successful. and the bilateral pedicle was clamped with noninvasive microvascular clips to block the blood supply of the kidneys for 40 min. Then the clips were released. The sham group only exposed the kidneys and did not undergo ischemic treatment. Mice were euthanized with CO_2_ after 24 h of reperfusion, and blood and kidney samples were collected.

### 2.4. Histopathology and immunohistochemistry

Kidney tissues with 4% paraformaldehyde fixed 24 h, paraffin embedding after dehydration and transparent, section 5um thick. The cells were then stained with hematoxylin and eosin (H&E). For immunohistochemical staining, the sections were oven-baked at 60 °C for 40 min, hydrated after immersion in xylene, and antigen repair was performed in 0.01 M citrate buffer. Then, after blocking with 3% BSA for half an hour at room temperature, KIM-1 (30948-1-AP 1:150), NGAL (26991-1-AP 1:200), IL-1β (AF06643 1:200), and NLRP3 (AF300649 1:250) antibody were added and placed flat in a wet box at 4 °C overnight. After the primary antibody was washed, HRP-secondary antibody was added and incubated at room temperature for 2 h. After washing, freshly prepared DAB chromogenic solution was added dropingly. Finally, the nuclei were counterstained with hematoxylin. Images were captured and analyzed with a panoramic slice scanning system (Olympus VS200, Japan).

### 2.5. RNA extraction and real-time PCR analysis

Total RNA of kidney tissue was analyzed with TRizol (ACCURATE BIOTECHNOLOGY (HUNAN) CO, LTD, ChangSha, China) reagent extraction. In brief, about 30 mg of kidney tissue was taken, TRizol reagent was added, the tissue was fragmented using a tissue homogenizer, left at room temperature for 5-10 min, and mixed with chloroform. After centrifugation, isopropanol was added to precipitate the RNA, and the total RNA precipitate was obtained by washing and drying.

PrimeScript^™^ RT was used Master Mix (Perfect Real Time) reagent. The equivalent of 1000 ng of RNA was reverse transcribed into cDNA. SYBR Green Pro Taq HS premixed qPCR Kit IV (ACCURATE BIOTECHNOLOGY (HUNAN) CO, LTD, ChangSha, China) was used for RT-PCR. The primer sequences are shown in Table 1. The relative expression of the target genes was calculated using the 2^−ΔΔCt^ method using β-actin as an internal reference.

**Table 1.** Primer sequences for RT PCR.

No.	Gene(Mouse)	Primer sequence
1	KIM-1-F	CCAGGCGCTGTGGATTCTTA
	KIM-1-R	TGACAAGCAGAAGATGGGCA
2	NGAL-F	ATGTCACCTCCATCCTGGTC
	NGAL-R	GCCACTTGCACATTGTAGCTC
3	IL-1β-F	TCCAGGATGAGGACATGAGCAC
	IL-1β-R	GAACGTCACACACCAGCAGGTTA
4	NLRP3-F	CCAGACACTCATGTTGCCTGTTC
	NLRP3-R	GAGGCTCCGGTTGGTGCTTA
5	CASP1-F	TGCTTTCTGCTCTTCAACACCA
	CASP1-R	CCAAGTCACAAGACCAGGCATAT
6	Gsdmd-F	AACCCCGTTATTCATGTGTCA
	Gsdmd-R	CAAAACACTCCGGTTCTGGT
7	β-actin-F	CACACCCGCCACCAGTTC
	β-actin-R	CCACGATGGAGGGGAATACAG
No.	Gene(Hum)	Primer sequence
1	KIM-1-F	GAACCCACCAGCTCACCATT
	KIM-1-R	AGAGCAAGAAGCACCAAGACA
2	NGAL-F	CAATGTCACCTCCGTCCTGT
	NGAL-R	TGCTGGTTGTAGTTGGTGCT
3	IL-1β-F	GGGACAGGATATGGAGCAACA
	IL-1β-R	ACACGCAGGACAGGTACAGA
4	NLRP3-F	GATCTTCGCTGCGATCAACA
	NLRP3-R	GGGATTCGAAACACGTGCATTA
5	CASP1-F	GCCTGTTCCTGTGATGTGGAG
	CASP1-R	TGCCCACAGACATTCATACAGTTTC
6	Gsdmd-F	GATGGGCAGATACAGGGCAG
	Gsdmd-R	CCAGGTGTTAGGGTCCACAC
7	β-actin-F	TGGCACCCAGCACAATGAA
	β-actin-R	CTAAGTCATAGTCCGCCTAGAAGCA

### 2.6. Bioinformatics analysis

The dynamic sequencing dataset of mouse ischemia-reperfusion was obtained from the Gene Expression Omnibus (GEO) database (GSE98622) and divided into four groups according to different time points (normal, 2 h, 4 h, 24 h). Then, use R package (WGCNA) analysis of sequencing data, obtained the most significant correlation between ischemia-reperfusion and set of genes, with R packet analyzer (clustering) KEGG enrichment analysis, find the key signaling pathways. Through consulting relevant literature and comparing with sequencing results (GSE98622), 45 genes related to pyroptosis were finally obtained. Use STRING for Protein-Protein Interaction (PPI) analysis and CytoScape for data presentation [17]. STRING was used for PPI analysis and cytoscape was used to visualize the data.

### 2.7. Cell culture

The HK-2 cell line is derived from human proximal tubular epithelial cells and is a relevant model for studying renal physiology and pathology, particularly ischemia-reperfusion injury. Human Renal Cortex Proximal Tubule Epithelial Cells (HK2) were purchased from the Center of Excellence in Molecular Cell Science, Chinese Academy of Sciences (Shanghai, China). HK2 was cultured in DMEM/F12 medium supplemented with 10% fetal bovine serum and 1% double antibody. 37 °C in 5% CO_2_, cell culture, in the digestion represented in logarithmic growth phase cells.

### 2.8. Cell viability assay

The cytotoxicity test was performed according to the instructions provided by Cell-Counting Kit-8. Briefly, HK2 cells in the logarithmic growth phase were seeded in 96-well plates with 90 μl of cell suspension per well and allowed to adhere overnight. After treatment with various concentrations of sodium aescinate, 10 μl of CCK8 reagent, as recommended by the manufacturer, was added to each well followed by incubation for 2 h in a cell incubator. Finally, the absorbance (OD) at 450 nm was measured using a microplate reader.

### 2.9. Cell hypoxia/reoxygenation (H/R) model

HK-2 cells in logarithmic growth phase were seeded in 12-well plates. After the cells were grown to 80%–90% density and completely adhered to the wall, different concentrations of sodium aescinate were used for intervention. The cells were washed with sugar-free Earle’s solution and then treated with sugar-free Earle’s solution containing 0.3 mmol·L-1Na_2_S_2_O_4_ for 2 h. After the end of the hypoxic treatment, the cells were washed twice with PBS and the medium was replaced by DMEM/F12 for 24 h. Then, the cells were cultured under normoxic conditions to establish a reoxygenation model. The cells were collected for RT-PCR and cellular immunofluorescence experiments.

### 2.10. Immunofluorescence detection

The expressions of AKT and p-AKT in HK-2 cells were detected by immunofluorescence. In short, HK-2 cells were fixed in 4% paraformaldehyde, added with primary antibodies AKT and p-AKT, incubated in 24-well plates at 4 °C overnight, added with secondary antibodies at room temperature for 1 h, and counterstained with 4 ‘-6-diamino-2-phenylindor (DAPI). The results were observed with a confocal laser scanning microscope (ZEISS LSM900, Germany), and representative images were selected for application.

### 2.11. Western blot assay

Total tissue and cell proteins were extracted using RAPT lysates. Protein concentration was determined by the BCA method. After separation by SDS-PAGE, equal amounts of proteins were transferred to PVDF membranes and then blocked with 5% skim milk. Primary antibodies AKT (ET1609-51), p-AKT (ET1607-73) and β-actin were added and incubated overnight at 4 °C, washed with 1× Tris-Buffered Saline Tween-20 and incubated with secondary antibodies for 2 h at room temperature. Then, ECL chemiluminescence kit was used for exposure development. Finally, ImageJ was used for semi-quantitative analysis of protein bands.

### 2.12. Statistical analysis

SPSS 20.0 software was used to analyze the experimental data, and the results were expressed as mean ± standard deviation (X ± SD). One-way analysis of variance was used for comparison among the three groups, after the ANOVA showed significant differences, the Tukey HSD test was used to compare groups to determine significant differences between specific groups. The *p <* 0.05 was considered statistically significant.

## 3. Results

### 3.1. SA can improve the renal injury in mice induced by IRI

**Figure 1.**
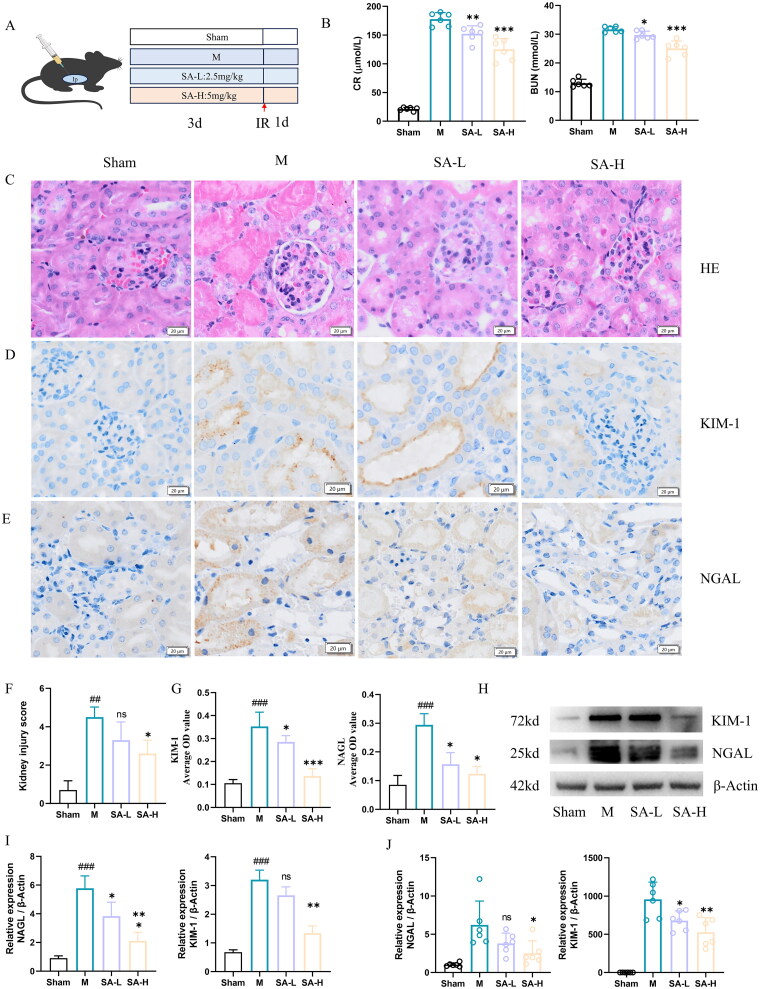
SA can improve the renal injury in mice induced by RIR A. Experimental design sketch. B. Kidney in mice serum creatinine and urea nitrogen levels. C. Representative images of paraffin sections of mouse kidney stained with H&E (scale bar, 20 μm). D. Mice kidney paraffin KIM - 1 immunohistochemical representative figure (scale bar, 20 μm). E. Mice kidney paraffin NGAL immunohistochemical representative figure (scale bar, 20 μm). F. Renal H&E histological and pathological score. G. NAGL,KIM-1 immunohistochemical statistics. H. Protein expression levels of KIM-1 and NAGL in Western blot bands in renal tissues. I statistical chart of KIM-1 and NAGL protein expression levels in renal tissue. J. KIM-1 and NAGL mRNA expression levels in renal tissues. Values are expressed as mean ± SD, *n* = 6. ^#^*p* < 0.05, ^##^*p* < 0.01, ^###^*p* < 0.001 vs sham group (Sham), ^ns^ *p* > 0.05, *p < 0.05, **p < 0.01, ***p < 0.001 vs.model (M).

We investigated the effect of SA on renal function in RIRI mice (Figure 1(A)). First, we examined the changes in renal function of mice in each group after intervention with different concentrations of SA, and the results showed that serum creatinine and urea nitrogen levels in the model group were significantly higher than those in the Sham-operated group. However, the levels of CR and BUN were lower in the 2 SA-treated groups (Figure 1(B)). H and E staining showed that the kidney structure of the Sham operation group was intact, the brush border structure and lumen were clear, while the kidney proximal flexural tubules of the model group showed obvious brush border loss, lumen expansion, and lumen cell debris accumulation and other phenomena. SA administration have attenuated the tubular damage, and the higher the dose, the more obvious the improvement effect on the tubular damage (Figure 1(C,F)). The results of immunohistochemical staining showed that the kidney sections of the RIRI mice showed positive immunoreactivity of KIM-1 and NGAL, while the IRI-induced elevation of these injury markers was improved after SA injection. (Figure 1(D,E,G)). SA treatment attenuated or abolished the IRI-induced increase in KIM-1 and NGAL mRNA expression and protein abundance. (Figure 1(H–J)).

### 3.2. Bioinformatics analysis showed that SA-treated RIRI through PI3K/AKT signaling pathway

**Figure 2.**
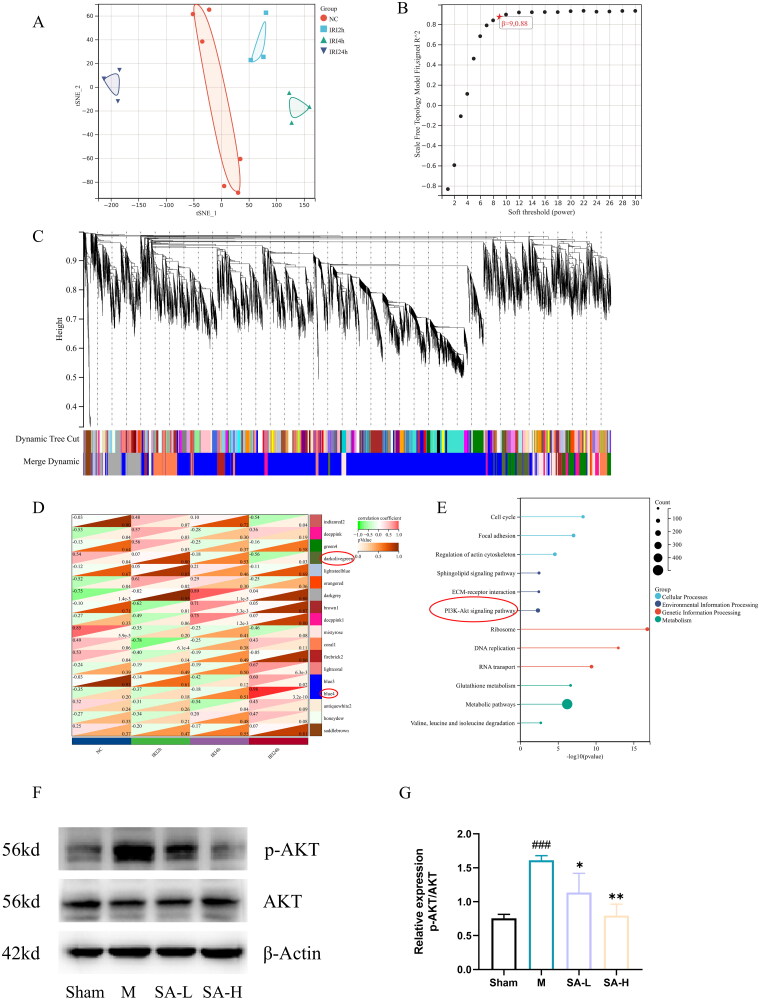
Bioinformatics analysis of dynamic changes in renal ischemia-reperfusion injury. A tSNE analysis of RNA sequencing at different times of renal ischemia-reperfusion. B the mean connectivity plot shows that 9 is the best soft-power. C WGCNA analyzed the 18 modules identified. D associations between identified modules and traits. E KEGG enrichment analysis of genes in modules of interest. F, G Protein expression of AKT and P-AKT.

The results of tSNE analysis showed a significant difference between the normal group and the ischemia-reperfusion groups at different times (Figure 2(A)). Next, we performed WGCNA analysis on the above transcriptome data, which showed good network connectivity at a soft threshold of 9 (Figure 2(B)). The hierarchical clustering tree of module identification is shown in Figure 2(C). The correlation heat map of the model kidney data shows that the blue4 and darkolivegreen are highly correlated with RIRI (Figure 2(D)). Furthermore, KEGG analysis of the genes in the above modules showed that PI3K/Akt signaling pathway played an important role in RIRI (Figure 2(E)). At the same time, Western blot results showed that the expression level of p-Akt in mouse kidney tissue significantly increased after injury, and SA treatment attenuated or abolished the effect of ischemia/reperfusion on p-Akt in a dose-dependent manner (*n* = 6). (Figure 2(F,G)).

### 3.3. SA inhibited RIR-induced pyroptosis

**Figure 3.**
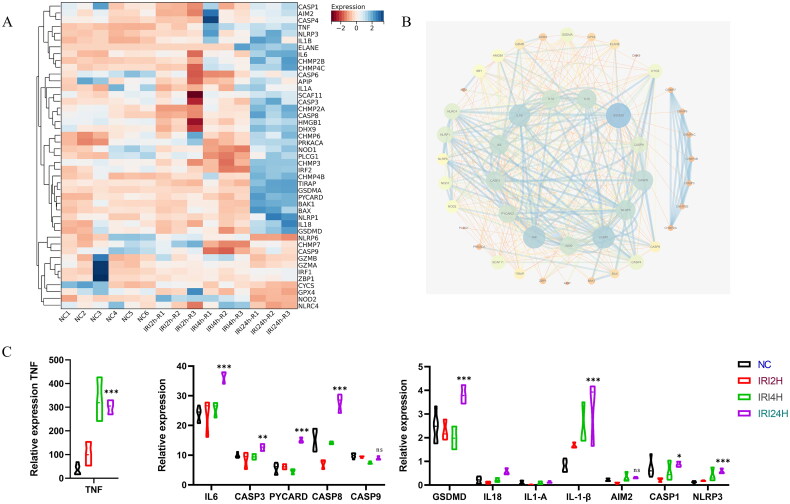
Activation of pyroptosis in the kidneys of IRI mice. A heat map of pyroptosis related genes in RIRI mouse model. B PPI analysis of genes in modules of interest. C the expression of pyroptosis related genes in the dynamic changes of renal ischemia-reperfusion injury. ^ns^ *p* > 0.05, *p < 0.05, **p < 0.01, ***p < 0.001 vs NC.

Subsequently, we analyzed the aforementioned transcriptome data. The heat map of pyroptosis-related genes is presented in Figure 3(A), indicating that the expression of most pyroptosis-associated genes was elevated in the kidneys of IRI mice. Similarly, PPI analysis demonstrated that pyroptosis-related genes play a significant role in the WGCNA network, as shown in Figure 3(B). The expression levels of differentially expressed pyroptosis-related genes in the kidneys of IRI mice at various time points are illustrated in Figure 3(C).

**Figure 4.**
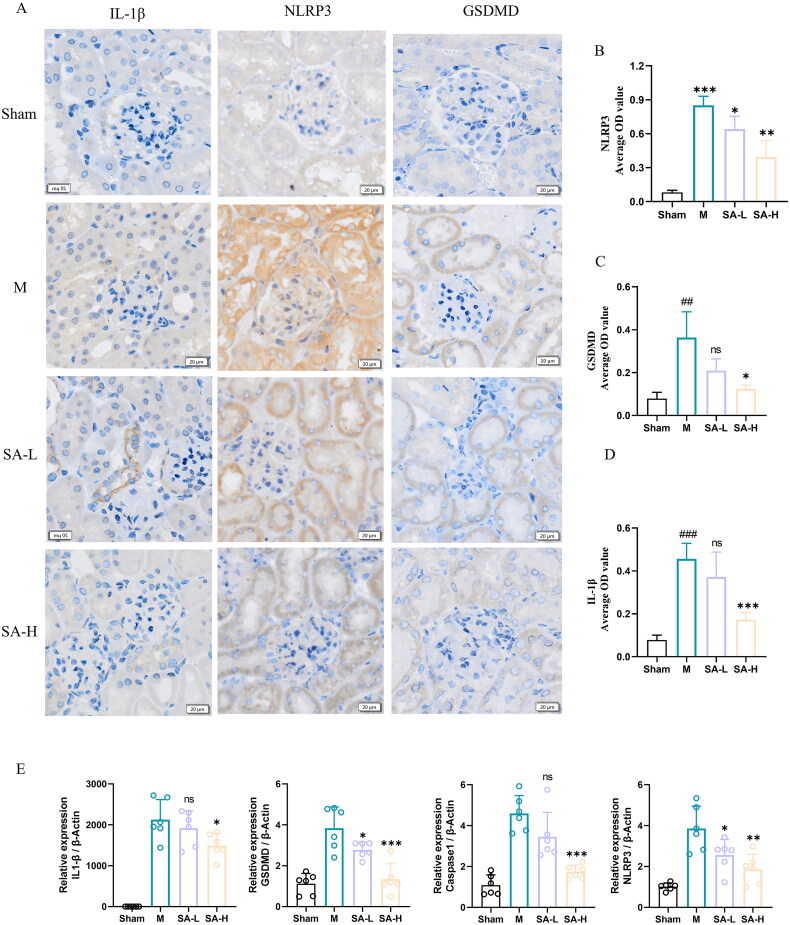
SA inhibited RIR-induced pyroptosis. A. Mice kidney paraffin IL-β, NLRP3, GSDMD immunohistochemical representative figure (scale bar, 20 μm). B-D. GSDMD, IL1-β, NLRP3 immunohistochemical statistics. E. IL-β, NLRP3, Caspase1 and GSDMD mRNA expression levels in renal tissues. Values are expressed as mean ± SD, *n* = 6. ^#^*p* < 0.05, ^##^*p* < 0.01, ^###^*p* < 0.001 vs sham group (Sham), ^ns^ *p* > 0.05, *p < 0.05, **p < 0.01, ***p < 0.001 vs model (M).

To further determine whether SA has an effect on pyroptosis, the expression levels of IL-1β, NLRP3, Caspase1 and GSDMD in renal tissues of each group of mice were measured. Immunohistochemical results showed that the expression of indicators related to pyroptosis was very low in the sham operation group, and the expression of pyroptosis related proteins increased after renal ischemia-reperfusion injury. SA treatment could attenuate the influence of hypoxia/reperfusion on the expression of pyroptosis related proteins (Figure 4(A–D)). At the same time, RT-PCR showed that the expression levels of IL-1β, NLRP3, Caspase1 and GSDMD in renal tissue were significantly increased after IRI, and SA treatment could attenuate the influence of hypoxia/reperfusion on the expression of these genes (Figure 4(E)).

### 3.4. SA inhibits the inflammatory response induced by H/R in HK-2 cells

**Figure 5.**
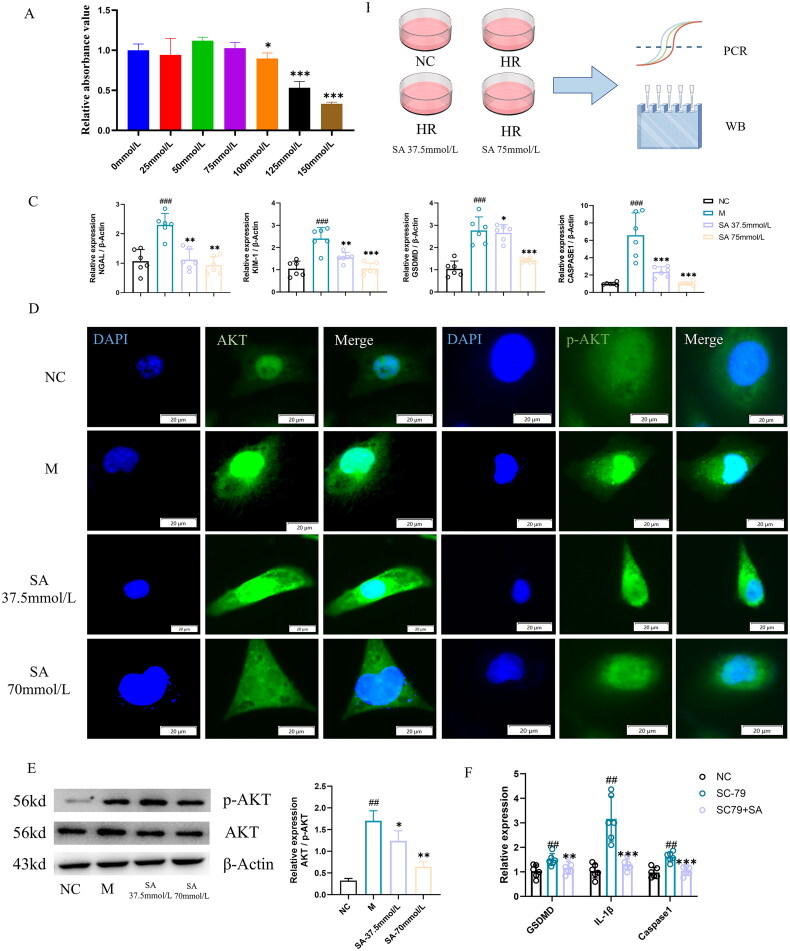
SA inhibits the inflammatory response induced by H/R in HK-2 cells. A. Cytotoxicity of different concentrations of SA on HK-2 cells. B. *In vitro* experiment design sketch. C. HK - 2 cells NAGL, KIM - 1, GSDMD, IL - beta mRNA expression level. D. Immunofluorescence representative plots of AKT and p-AKT in HK-2 cells (scale bar, 20 μm). E. AKT and p-AKT protein expression levels in HK-2 cells. F. Expression levels of GSDMD, IL-1β and Caspase1 mRNA in HK-2 cells after AKT activation. Values are expressed as mean ± SD, *n* = 3. ^#^*p* < 0.05, ^##^*p* < 0.01, ^###^*p* < 0.001 vs sham group (Sham), ^ns^ *p* > 0.05, *p < 0.05, **p < 0.01, ***p < 0.001 vs model (M).

First, we evaluated the effect of different concentrations of SA on HK-2 cells toxicity. CCK8 results showed that SA had no significant effect on the viability of HK-2 cells in the range of 0-75 mmol/L (Figure 5(A)). Then, the HK-2 cells were cultured in hypoxia and reoxygenation environment to simulate the two processes of renal ischemia and reperfusion, and the cells were collected for further detection (Figure 5(B)). Specifically, compared with the control group, the expression levels of IL-1β, NLRP3, Caspase1, and GSDMD mRNA in the hypoxia/reoxygenation model were significantly up-regulated, SA treatment either attenuated or abolished the effect of H/R. (Figure 5(C)). In addition, immunofluorescence and Western blot results showed that SA-cotreatment did not reverse the increase in p-Akt. SA-treatment attenuated or abolished the effect of ischemia/reperfusion on the p-Akt in a dose-dependent manner. (Figure 5(D,E)). To complete our *in vitro* studies, we used the AKT agonist SC79 to assess whether SA improved HK-2 inflammation by inhibiting AKT activation. The results showed that compared with the normal group, the expression levels of IL-1β, Caspase1 and GSDMD mRNA were significantly up-regulated after SC79 intervention, SA treatment either attenuated or abolished the effect of H/R. (Figure 5(F)).

## 4. Discussion

Acute renal injury caused by RIRI poses a major challenge to clinicians. SA a triterpene saponin compound containing ester bonds, has demonstrated high pharmacological activity, low cytotoxicity, and multi-target capabilities, making it a promising candidate for treating multifactorial diseases. In this study, we evaluated the effects of SA on various indicators of RIRI, including serum markers of renal function, renal injury molecules, NLRP3 inflammasome activation, AKT signaling pathway activation, and pyroptosis-related inflammatory cytokines. Utilizing database-based enrichment analysis, coupled with *in vitro* and *in vivo* pharmacological experiments, we discovered that inhibiting AKT/NLRP3 inflammasome activation attenuated inflammatory responses and pyroptosis, thereby ameliorating RIR-induced acute kidney injury.

RIRI is a complex pathological process involving various mechanisms, including inflammatory responses, oxidative stress, intracellular calcium overload, activation of the renin-angiotensin system, and microcirculatory disturbances. These factors interact with each other, leading to renal tubular epithelial cell damage, renal dysfunction, and even progression to chronic kidney disease [1,2]. Numerous studies have identified pyroptosis as a pivotal mechanism in renal IRI [8,9]. Pyroptosis, an inflammatory form of cell death, initiates a cascade of inflammatory responses. The NLRP3 inflammasome, an innate immune receptor, plays a critical role in this process by recognizing multiple danger signals during renal ischemia-reperfusion and being expressed in various tissues, including the kidney [18,19]. Upon activation, NLRP3 stimulates Caspase-1, which cleaves GSDMD, releasing its N-terminal fragment that forms pores in cell membranes, thereby triggering pyroptosis [9]. Interestingly, our findings revealed that the duration of renal ischemia-reperfusion was directly proportional to the expression of inflammatory and pyroptosis-related genes, suggesting enhanced inflammatory cascade amplification in the later stages of injury. Our results confirmed that SA reduced pyroptosis by inhibiting the expression of NLRP3 inflammasome and other pyroptotic proteins during the peak inflammatory response. Notably, GSDMD, a key effector of pyroptosis, was also suppressed by SA, affirming the crucial role of pyroptosis in renal ir injury and its inhibition by SA.

The PI3K/AKT signaling pathway is an essential upstream regulator of NLRP3 inflammasome activation and plays a pivotal role in the survival and pathophysiology of ischemia-reperfusion injury [20]. Enhanced AKT phosphorylation promotes NLRP3 inflammasome activation, thereby increasing cytokine production, including IL-1β and IL-18 [21]. In the rat model of polycystic ovary syndrome, SA can also relieve PCOS symptoms in the rat model by regulating the PI3K/AKT signaling pathway [22]. Furthermore, studies have shown that in rats with post-traumatic stress disorder, NLRP3 inflammasome activation in the dorsal raphe nucleus impairs mitochondrial function by inhibiting ATP synthesis and increasing ROS production, while SA can effectively reverse the pathological progression of mitochondria [13].Our study demonstrated that SA reduced levels of phosphorylated AKT in renal tissues and renal tubular epithelial cells during IRI. Importantly, we found that SA inhibited the activity of activated AKT, leading to reduced GSDMD-induced pyroptosis, suggesting a potential novel therapeutic direction for SA involving the AKT/NLRP3/GSDMD axis.

While the mechanisms underlying IRI are inherently multifaceted, encompassing diverse processes such as inflammation, oxidative stress, and angiogenesis, our study illuminates just one of many potential pathways involved in this complex pathophysiology. Several limitations to this research must be acknowledged, most notably the necessity for prospective clinical trials to validate the efficacy of SA in human subjects, as our current findings are derived exclusively from experimental models. The translation of these findings from the laboratory to clinical application presents notable challenges, including concerns regarding SA’s bioavailability, toxicological safety, and the determination of an optimal dosage regimen. Furthermore, the treatment time window and dose of SA may need to be further optimized. Future studies could consider adjusting the timing and dose of administration to assess its longer-term effect on renal function recovery. In order to improve kidney function impairment more effectively, it may be necessary to combine other treatments. For example, in combination with antioxidants, anti-inflammatory drugs, or cell protectants, the multiple pathological processes caused by RIRI can be better alleviated, leading to a more complete restoration of kidney function. Although SA treatment can improve renal function indicators to a certain extent, there are still significant renal damage, which suggests that the recovery of renal function is a multi-factor, multi-stage process. Future research is needed to further optimize treatment options at multiple levels to achieve a more complete and lasting recovery of kidney function.

In conclusion, while acknowledging the existing limitations and challenges, we posit that SA holds substantial therapeutic promise for the treatment of RIRI. However, successful translation into clinical practice will require a concerted effort through continued research and validation.

## Supplementary Material

Supplementary data 1 Expression of genes related to pyroptosis.docx

Supplementary data 3 wgcna module.docx

Supplementary data 2 KEGG enrichment analysis.docx

Raw data.docx
