# Supplementary material for: Sodium aescinate protects renal ischemia-reperfusion and pyroptosis through AKT/NLRP3 signaling pathway
Source: Ren Fail. 2025 Apr 22;47(1):2488140. doi: 10.1080/0886022X.2025.2488140 (PMC12016278; doi:10.1080/0886022X.2025.2488140)
Supplement: Supplementary data 1 Expression of genes related to pyroptosis.docx [file IRNF_A_2488140_SM1631.docx]

| symbol | NC1 | NC2 | NC3 | NC4 | NC5 | NC6 | IRI2h-R1 | IRI2h-R2 | IRI2h-R3 | IRI4h-R1 | IRI4h-R2 | IRI4h-R3 | IRI24h-R1 | IRI24h-R2 | IRI24h-R3 |
| --- | --- | --- | --- | --- | --- | --- | --- | --- | --- | --- | --- | --- | --- | --- | --- |
| BAK1 | 3.9626 | 4.7049 | 8.6795 | 5.1634 | 4.9393 | 4.9747 | 4.3 | 4.2226 | 6.6752 | 6.6615 | 3.7969 | 3.6255 | 15.6902 | 15.1854 | 14.9088 |
| BAX | 36.0545 | 40.565 | 42.4538 | 45.6066 | 44.5038 | 45.3826 | 38.4533 | 40.5484 | 54.782 | 49.7424 | 37.24 | 38.6202 | 87.2 | 84.6982 | 70.7442 |
| CHMP2A | 88.3047 | 95.43 | 96.3067 | 85.4691 | 81.7187 | 89.6714 | 68.6762 | 68.6885 | 66.2674 | 97.5378 | 84.8423 | 84.6654 | 120.5231 | 113.0316 | 123.7031 |
| CHMP2B | 31.8734 | 34.1931 | 32.2664 | 33.1191 | 34.116 | 33.9402 | 36.3655 | 38.1191 | 27.7513 | 51.7295 | 43.9492 | 42.1083 | 65.4541 | 54.8777 | 67.8239 |
| CHMP3 | 44.6463 | 44.3049 | 46.0355 | 43.3789 | 44.0347 | 43.7283 | 40.2769 | 42.8336 | 36.1382 | 38.8719 | 34.5705 | 38.39 | 51.0894 | 50.8288 | 50.8616 |
| CHMP4B | 77.923 | 81.7877 | 98.5242 | 74.6165 | 77.296 | 75.84 | 78.4196 | 85.8187 | 85.1189 | 113.9342 | 84.1747 | 85.2471 | 126.4633 | 120.7873 | 126.2453 |
| CHMP4C | 3.7281 | 2.8628 | 3.1616 | 3.3883 | 3.3412 | 3.3778 | 2.8749 | 2.9741 | 1.1143 | 5.4635 | 5.0528 | 4.0829 | 5.9032 | 5.9341 | 7.2132 |
| CHMP6 | 50.3802 | 45.5817 | 49.8105 | 57.4899 | 60.9084 | 65.5545 | 53.5523 | 61.5489 | 79.707 | 54.9581 | 50.9773 | 55.4752 | 71.8682 | 66.0734 | 77.5087 |
| CHMP7 | 21.8537 | 20.8815 | 22.7248 | 23.7112 | 25.5806 | 24.9581 | 24.0667 | 26.25 | 27.1586 | 20.7212 | 19.67 | 19.7239 | 23.2463 | 23.519 | 22.3564 |
| CYCS | 123.0781 | 130.2127 | 121.538 | 77.8978 | 73.8515 | 75.9115 | 97.5786 | 96.2061 | 91.747 | 77.6645 | 95.5804 | 83.9299 | 81.0025 | 74.4456 | 72.8501 |
| HMGB1 | 52.9316 | 52.3477 | 55.4668 | 50.0615 | 52.291 | 53.2654 | 40.5512 | 44.0387 | 21.9626 | 45.4582 | 41.6639 | 47.6661 | 61.6146 | 58.3874 | 60.6356 |
| IL1A | 0.0348 | 0.0445 | 0.0103 | 0.197 | 0.097 | 0.1655 | 0.0106 | 0.0332 | 0.0121 | 0.0714 | 0.0123 | 0.1591 | 0.1746 | 0.0969 | 0.0964 |
| IRF1 | 6.1006 | 4.8039 | 40.9942 | 5.1828 | 5.2264 | 6.2987 | 9.2528 | 7.9702 | 9.4939 | 10.2066 | 7.4295 | 10.7652 | 12.6783 | 12.5339 | 13.49 |
| IRF2 | 8.3812 | 9.1055 | 10.4763 | 10.6567 | 10.0047 | 10.8666 | 9.5422 | 10.1908 | 8.9366 | 8.2705 | 7.0116 | 7.1441 | 12.8215 | 12.5129 | 12.4444 |
| CASP6 | 12.8179 | 14.8281 | 14.3182 | 15.4684 | 16.8912 | 16.0896 | 13.12 | 14.2226 | 9.5422 | 10.5103 | 10.6047 | 12.6167 | 14.4173 | 13.1607 | 14.8007 |
| CASP9 | 8.5728 | 9.4036 | 9.5069 | 10.6879 | 10.4796 | 10.1635 | 9.5638 | 9.0339 | 9.63 | 7.2745 | 7.0816 | 7.9552 | 8.4987 | 9.6968 | 8.6045 |
| GPX4 | 656.7508 | 792.1892 | 866.9688 | 735.0149 | 683.5897 | 744.2541 | 602.9992 | 621.0097 | 954.3281 | 631.9649 | 569.4445 | 691.02 | 567.6391 | 572.4416 | 542.86 |
| IL6 | 24.2352 | 21.1674 | 20.8014 | 26.7713 | 24.8357 | 25.1313 | 27.7323 | 26.6164 | 16.1591 | 26.5568 | 27.6034 | 22.6767 | 34.0536 | 37.7081 | 37.9528 |
| NLRP3 | 0.131 | 0.1146 | 0.1621 | 0.0752 | 0.1189 | 0.1748 | 0.1775 | 0.1682 | 0.2073 | 0.7465 | 0.1994 | 0.2991 | 0.5246 | 0.7036 | 0.499 |
| NLRP6 | 18.9705 | 10.5693 | 15.8782 | 46.6746 | 47.2954 | 45.2706 | 27.9019 | 33.0656 | 34.6218 | 24.3501 | 25.8297 | 31.1561 | 8.4611 | 8.665 | 5.2858 |
| NOD1 | 3.2916 | 3.381 | 4.1819 | 4.9656 | 5.3633 | 4.8398 | 3.5267 | 3.8273 | 3.9356 | 2.8822 | 2.6599 | 2.499 | 4.5979 | 4.982 | 5.4989 |
| NOD2 | 0.5728 | 0.7997 | 0.7038 | 0.7275 | 0.7401 | 0.7769 | 1.3036 | 1.1283 | 1.1662 | 1.1781 | 1.0661 | 0.9587 | 0.5703 | 0.6281 | 0.6223 |
| PLCG1 | 8.1792 | 7.7086 | 7.5756 | 10.4536 | 10.3552 | 11.0775 | 8.973 | 10.0684 | 9.5871 | 6.7669 | 6.3995 | 7.1583 | 9.0916 | 11.502 | 10.8884 |
| PRKACA | 29.6997 | 25.2916 | 26.2216 | 37.9408 | 39.5809 | 39.0378 | 39.1045 | 41.3983 | 48.3885 | 27.5848 | 29.9909 | 33.3444 | 45.4611 | 45.9246 | 46.0559 |
| PYCARD | 3.3567 | 3.4646 | 6.2469 | 5.9586 | 7.5207 | 6.8588 | 5.2268 | 6.0536 | 6.9984 | 4.7722 | 3.492 | 5.3014 | 16.0032 | 14.2003 | 14.6958 |
| SCAF11 | 15.9136 | 13.3566 | 12.8001 | 15.3808 | 16.382 | 15.9657 | 13.0717 | 15.162 | 4.4809 | 16.1623 | 15.1382 | 15.2977 | 17.3206 | 18.0432 | 16.9624 |
| TIRAP | 2.5029 | 2.7696 | 2.5012 | 2.5986 | 2.5355 | 2.6561 | 2.0471 | 1.7383 | 1.5244 | 3.094 | 1.8831 | 1.5083 | 5.3934 | 6.0139 | 6.0783 |
| TNF | 52.1459 | 68.4227 | 45.6838 | 19.8246 | 16.716 | 18.7225 | 99.4454 | 53.4632 | 153.8645 | 427.0898 | 319.4267 | 241.7018 | 305.1524 | 269.9998 | 329.7793 |
| APIP | 10.7963 | 15.7861 | 14.8615 | 11.1432 | 12.3461 | 12.6307 | 10.767 | 11.2151 | 8.5443 | 10.2091 | 10.4973 | 12.1817 | 14.2665 | 13.7378 | 11.7116 |
| DHX9 | 45.9591 | 33.9403 | 33.1734 | 33.7089 | 35.0548 | 36.5254 | 23.4469 | 29.1472 | 7.9847 | 35.7026 | 33.1488 | 37.1691 | 57.5507 | 53.6307 | 55.6614 |
| GZMA | 0.3664 | 0.1003 | 1.4199 | 0.0493 | 0 | 0.2008 | 0.3118 | 0.2742 | 0.2449 | 0.3484 | 0.2776 | 0.2689 | 0.0492 | 0.1311 | 0.1931 |
| ZBP1 | 0.2685 | 0.3561 | 2.7559 | 0.279 | 0.2732 | 0.3925 | 0.3155 | 0.4722 | 0.4582 | 0.5429 | 0.2849 | 0.3773 | 0.6212 | 0.5115 | 0.7746 |
| CASP3 | 9.1033 | 9.4591 | 9.4024 | 9.7246 | 10.3295 | 11.12 | 9.5287 | 10.8359 | 5.2994 | 10.4206 | 9.4236 | 8.501 | 12.6924 | 13.7756 | 11.4065 |
| ELANE | 0 | 0 | 0 | 0 | 0 | 0 | 0 | 0 | 0 | 0 | 0 | 0.0285 | 0.0235 | 0.0208 | 0.023 |
| IL18 | 0.0826 | 0.058 | 0.3724 | 0.2492 | 0.3106 | 0.1993 | 0.106 | 0.1522 | 0.0516 | 0.3046 | 0.1286 | 0.1949 | 0.4504 | 0.5058 | 0.7163 |
| IL1B | 0.7113 | 1.0056 | 1.1297 | 0.5586 | 0.5836 | 0.9096 | 1.5831 | 1.8068 | 1.6026 | 3.503 | 1.8865 | 1.8752 | 4.1699 | 3.9299 | 1.6554 |
| GZMB | 0.1455 | 0.0155 | 0.4746 | 0.0152 | 0.0338 | 0.0709 | 0.2815 | 0.0924 | 0.1513 | 0.2483 | 0.2916 | 0.1661 | 0.0304 | 0.0675 | 0.0745 |
| AIM2 | 0.2828 | 0.178 | 0.2875 | 0.1676 | 0.1688 | 0.2392 | 0.1037 | 0.1078 | 0.042 | 0.5627 | 0.2229 | 0.1845 | 0.281 | 0.3036 | 0.3502 |
| CASP8 | 17.0895 | 18.5842 | 18.9399 | 13.5153 | 11.196 | 11.3835 | 7.7376 | 8.3382 | 5.3029 | 14.6484 | 13.6897 | 14.0824 | 30.4469 | 24.2148 | 28.5664 |
| GSDMA | 0.0084 | 0 | 0.015 | 0 | 0 | 0.0092 | 0.0077 | 0 | 0 | 0.0259 | 0 | 0 | 0.174 | 0.1827 | 0.1863 |
| NLRC4 | 1.8317 | 1.44 | 1.6982 | 2.4067 | 1.5575 | 1.2753 | 1.6677 | 1.1367 | 0.6267 | 1.503 | 2.4117 | 2.3021 | 1.1271 | 0.8467 | 0.9518 |
| NLRP1 | 0.0059 | 0.0338 | 0.0366 | 0 | 0 | 0.0322 | 0.0108 | 0.0168 | 0.0061 | 0.018 | 0 | 0.0067 | 0.0497 | 0.1569 | 0.1408 |
| CASP1 | 0.7989 | 0.6621 | 1.3119 | 0.3475 | 0.5134 | 0.5389 | 0.2957 | 0.2634 | 0.1278 | 1.0383 | 0.2282 | 0.4385 | 0.8519 | 0.7695 | 0.9917 |
| CASP4 | 1.3375 | 1.1655 | 1.479 | 0.5678 | 0.5536 | 0.7792 | 0.4086 | 0.4591 | 0.3133 | 4.7997 | 1.1888 | 0.9353 | 1.257 | 1.7803 | 1.7334 |
| GSDMD | 1.7603 | 1.8719 | 3.3272 | 2.5374 | 2.4328 | 2.7241 | 1.9217 | 2.1577 | 2.7728 | 2.4786 | 1.5105 | 1.98 | 3.4426 | 3.7815 | 4.2253 |
