# Supplementary material for: Sodium aescinate protects renal ischemia-reperfusion and pyroptosis through AKT/NLRP3 signaling pathway
Source: Ren Fail. 2025 Apr 22;47(1):2488140. doi: 10.1080/0886022X.2025.2488140 (PMC12016278; doi:10.1080/0886022X.2025.2488140)
Supplement: Supplementary data 3 wgcna module.docx [file IRNF_A_2488140_SM1630.docx]

| Gene | mergeModule |
| --- | --- |
| Sox17 | blue4 |
| RP23-34E15.4 | blue4 |
| Mrpl15 | blue4 |
| Lypla1 | blue4 |
| Tcea1 | green4 |
| Atp6v1h | deeppink |
| Rb1cc1 | deeppink |
| 4732440D04Rik | blue3 |
| Pcmtd1 | mistyrose |
| Gm9826 | mistyrose |
| Gm15452 | blue4 |
| Rrs1 | blue4 |
| Adhfe1 | blue4 |
| Vcpip1 | indianred2 |
| 1700034P13Rik | blue4 |
| Sgk3 | coral1 |
| Snhg6 | blue4 |
| Snord87 | deeppink1 |
| Tcf24 | blue4 |
| Ppp1r42 | blue4 |
| Cops5 | coral1 |
| Cspp1 | blue3 |
| Arfgef1 | blue4 |
| Prex2 | firebrick2 |
| Ncoa2 | lightsteelblue |
| Tram1 | blue4 |
| Lactb2 | blue4 |
| RP23-70F22.2 | darkolivegreen |
| Terf1 | coral1 |
| Rpl7 | blue4 |
| Rdh10 | blue4 |
| Stau2 | blue4 |
| Ube2w | antiquewhite2 |
| Tceb1 | green4 |
| Tmem70 | coral1 |
| Ly96 | blue4 |
| Pi15 | darkgrey |
| Tfap2b | blue3 |
| Pkhd1 | blue3 |
| Mcm3 | blue4 |
| Tram2 | blue4 |
| Gm2693 | blue4 |
| Tmem14a | blue4 |
| Gsta3 | darkgrey |
| Gm4956 | blue4 |
| Gm7658 | blue4 |
| Gm27028 | firebrick2 |
| Ogfrl1 | blue4 |
| B3gat2 | green4 |
| Smap1 | blue4 |
| 1110058L19Rik | blue4 |
| Fam135a | blue4 |
| Gm28439 | blue4 |
| Gm10222 | blue4 |
| Gm28438 | coral1 |
| Gm28437 | blue4 |
| Gm10925 | blue4 |
| Gm28661 | blue4 |
| Gm29216 | blue4 |
| Lmbrd1 | deeppink |
| Gm5524 | darkolivegreen |
| Gm7846 | blue4 |
| Phf3 | blue4 |
| Ptp4a1 | darkgrey |
| Prim2 | blue4 |
| RP24-423J19.1 | blue4 |
| Rab23 | deeppink |
| Bag2 | blue4 |
| Zfp451 | blue4 |
| Dst | lightsteelblue |
| RP24-422C24.2 | brown1 |
| Ccdc115 | blue4 |
| Imp4 | blue4 |
| Ptpn18 | blue3 |
| Gm28417 | blue3 |
| Fam168b | blue4 |
| Plekhb2 | blue4 |
| Hs6st1 | deeppink |
| Uggt1 | blue4 |
| Neurl3 | coral1 |
| Arid5a | darkgrey |
| Kansl3 | darkolivegreen |
| Fer1l5 | green4 |
| Lman2l | green4 |
| Cnnm4 | orangered |
| Cnnm3 | green4 |
| Ankrd39 | blue3 |
| Sema4c | darkgrey |
| D430040D24Rik | mistyrose |
| Cox5b | blue4 |
| Actr1b | deeppink |
| Tmem131 | blue3 |
| Inpp4a | blue3 |
| Coa5 | blue4 |
| Unc50 | mistyrose |
| Mgat4a | blue4 |
| Tsga10 | firebrick2 |
| Lipt1 | blue3 |
| Mitd1 | coral1 |
| Mrpl30 | lightsteelblue |
| Txndc9 | coral1 |
| Eif5b | blue4 |
| Rev1 | blue4 |
| Pdcl3 | blue4 |
| RP23-101P2.6 | blue3 |
| Npas2 | mistyrose |
| Rpl31 | blue4 |
| Tbc1d8 | blue4 |
| Cnot11 | darkolivegreen |
| Gm15832 | blue4 |
| Snord89 | blue4 |
| Rnf149 | green4 |
| Gm23722 | blue4 |
| Map4k4 | blue4 |
| Il1r2 | darkgrey |
| Il1r1 | blue4 |
| Slc9a4 | blue4 |
| Slc9a2 | darkolivegreen |
| Mfsd9 | mistyrose |
| Pantr1 | blue4 |
| Gm20646 | darkolivegreen |
| Pou3f3 | darkolivegreen |
| Pantr2 | blue3 |
| Mrps9 | coral1 |
| Tgfbrap1 | green4 |
| AI597479 | blue4 |
| Fhl2 | blue4 |
| Nck2 | blue4 |
| 1500015O10Rik | blue4 |
| Gm29156 | coral1 |
| Uxs1 | blue4 |
| Tpp2 | blue4 |
| Tex30 | blue4 |
| Kdelc1 | blue4 |
| Bivm | coral1 |
| Ercc5 | blue4 |
| Txn-ps1 | blue4 |
| Gulp1 | blue4 |
| Col3a1 | blue4 |
| Col5a2 | blue4 |
| Wdr75 | blue4 |
| Gm5526 | blue4 |
| Slc40a1 | blue4 |
| Dnah7b | firebrick2 |
| Slc39a10 | blue4 |
| Gm28151 | blue3 |
| Sdpr | coral1 |
| Nabp1 | blue4 |
| Gm17767 | darkgrey |
| Myo1b | blue4 |
| Stat1 | saddlebrown |
| Gls | blue4 |
| Nab1 | coral1 |
| Mfsd6 | blue4 |
| Inpp1 | mistyrose |
| Hibch | mistyrose |
| Pms1 | coral1 |
| Ormdl1 | coral1 |
| Osgepl1 | mistyrose |
| Stk17b | darkgrey |
| Hecw2 | brown1 |
| Gtf3c3 | coral1 |
| Pgap1 | blue3 |
| Ankrd44 | coral1 |
| Sf3b1 | deeppink1 |
| RP23-16N14.2 | blue4 |
| Coq10b | blue3 |
| Gm17971 | darkgrey |
| Hspd1 | darkgrey |
| Hspe1 | darkgrey |
| Mob4 | green4 |
| Rftn2 | blue3 |
| Mars2 | blue3 |
| Gm8292 | blue4 |
| Plcl1 | blue4 |
| Satb2 | blue3 |
| 9130024F11Rik | darkolivegreen |
| 1700066M21Rik | blue4 |
| Tyw5 | green4 |
| 9430016H08Rik | darkgrey |
| Spats2l | lightsteelblue |
| Kctd18 | blue4 |
| Bzw1 | blue4 |
| Clk1 | deeppink |
| Ppil3 | blue4 |
| Nif3l1 | blue3 |
| Orc2 | coral1 |
| Gm23966 | blue4 |
| Fam126b | coral1 |
| Ndufb3 | mistyrose |
| Als2cr12 | coral1 |
| Gm20257 | blue4 |
| Cflar | brown1 |
| Casp8 | blue4 |
| Trak2 | deeppink1 |
| Stradb | darkolivegreen |
| Tmem237 | blue3 |
| Mpp4 | blue3 |
| Als2 | blue4 |
| Fzd7 | blue3 |
| Sumo1 | mistyrose |
| Nop58 | blue4 |
| Snord70 | deeppink1 |
| Bmpr2 | blue4 |
| Fam117b | blue3 |
| Wdr12 | blue4 |
| Carf | blue3 |
| Nbeal1 | blue4 |
| Cyp20a1 | blue4 |
| Abi2 | blue4 |
| Raph1 | blue4 |
| Eif4a-ps4 | blue4 |
| Pard3b | blue4 |
| Nrp2 | blue4 |
| Gm4208 | blue4 |
| Ino80d | blue4 |
| Ino80dos | blue3 |
| Ndufs1 | deeppink |
| Eef1b2 | blue4 |
| Gm26457 | darkgrey |
| Snora41 | darkgrey |
| Gm11605 | blue4 |
| Fastkd2 | coral1 |
| Klf7 | darkgrey |
| Creb1 | blue4 |
| Mettl21a | blue4 |
| Ccnyl1 | blue4 |
| Fzd5 | darkgrey |
| Rpl10a-ps1 | blue4 |
| Gm7329 | mistyrose |
| D630023F18Rik | mistyrose |
| Idh1 | blue4 |
| Pikfyve | blue4 |
| Map2 | blue4 |
| Rpe | coral1 |
| Kansl1l | blue3 |
| RP24-429G21.1 | blue4 |
| Acadl | coral1 |
| Lancl1 | blue4 |
| Erbb4 | blue4 |
| Ikzf2 | blue4 |
| Atic | blue4 |
| Fn1 | mistyrose |
| Mreg | blue4 |
| D230017M19Rik | darkolivegreen |
| Gm25939 | darkolivegreen |
| Pecr | blue4 |
| Tmem169 | blue3 |
| Xrcc5 | coral1 |
| Smarcal1 | blue3 |
| Rpl37a | blue4 |
| Igfbp2 | green4 |
| Igfbp5 | green4 |
| Tns1 | green4 |
| Rufy4 | darkolivegreen |
| Cxcr2 | blue4 |
| Arpc2 | blue4 |
| Gpbar1 | blue4 |
| Aamp | coral1 |
| Pnkd | darkolivegreen |
| RP23-280F6.5 | darkgrey |
| Tmbim1 | blue4 |
| Slc11a1 | honeydew |
| Ctdsp1 | green4 |
| Mir26b | coral1 |
| Vil1 | blue4 |
| Usp37 | blue4 |
| Rqcd1 | blue4 |
| Zfp142 | mistyrose |
| Bcs1l | blue3 |
| Rnf25 | blue4 |
| Stk36 | green4 |
| Ttll4 | blue3 |
| Cyp27a1 | blue4 |
| Prkag3 | darkgrey |
| Wnt10a | darkgrey |
| Ihh | mistyrose |
| Nhej1 | green4 |
| Slc23a3 | blue4 |
| Cnppd1 | blue4 |
| Fam134a | blue4 |
| Zfand2b | indianred2 |
| Atg9a | green4 |
| Ankzf1 | darkolivegreen |
| Glb1l | blue4 |
| Stk16 | green4 |
| Tuba4a | coral1 |
| Dnajb2 | mistyrose |
| Dnpep | blue3 |
| Des | blue4 |
| Speg | blue4 |
| Gmppa | blue3 |
| Chpf | mistyrose |
| Obsl1 | blue4 |
| Inha | blue3 |
| Stk11ip | brown1 |
| Slc4a3 | blue3 |
| Epha4 | lightsteelblue |
| Sgpp2 | blue3 |
| Farsb | blue4 |
| BC035947 | blue3 |
| Mogat1 | blue4 |
| Acsl3 | coral1 |
| Kcne4 | coral1 |
| Ap1s3 | firebrick2 |
| Wdfy1 | mistyrose |
| Mrpl44 | blue4 |
| Serpine2 | blue4 |
| Gm5529 | antiquewhite2 |
| Cul3 | coral1 |
| Dock10 | blue4 |
| Irs1 | mistyrose |
| Rhbdd1 | blue4 |
| Col4a4 | blue4 |
| Col4a3 | firebrick2 |
| Mff | coral1 |
| Gm28941 | blue3 |
| Agfg1 | blue4 |
| Slc19a3 | firebrick2 |
| Pid1 | blue4 |
| Trip12 | coral1 |
| Fbxo36 | blue4 |
| Slc16a14 | blue4 |
| Gm29284 | blue4 |
| Rpl19-ps1 | blue4 |
| Sp110 | blue4 |
| Sp100 | blue4 |
| A630001G21Rik | blue3 |
| Cab39 | coral1 |
| Itm2c | deeppink |
| 2810459M11Rik | blue4 |
| Psmd1 | blue4 |
| Armc9 | green4 |
| B3gnt7 | blue4 |
| Ncl | blue4 |
| Ptma | blue4 |
| Pde6d | blue3 |
| Cops7b | blue3 |
| Nppc | darkgrey |
| Dis3l2 | coral1 |
| Gm6136 | blue4 |
| Eif4e2 | blue4 |
| Efhd1 | green4 |
| Gigyf2 | blue3 |
| Ngef | darkolivegreen |
| Neu2 | darkolivegreen |
| Gm17181 | deeppink1 |
| Inpp5d | honeydew |
| Atg16l1 | blue4 |
| Dgkd | orangered |
| Usp40 | coral1 |
| Ugt1a10 | blue3 |
| Ugt1a7c | darkgrey |
| Ugt1a2 | blue4 |
| Dnajb3 | blue4 |
| Hjurp | blue3 |
| Spp2 | blue4 |
| Arl4c | darkgrey |
| Sh3bp4 | darkgrey |
| Agap1 | blue3 |
| Ackr3 | blue4 |
| Cops8 | blue4 |
| Col6a3 | blue4 |
| Mlph | blue4 |
| Rab17 | green4 |
| Lrrfip1 | blue4 |
| Ube2f | blue4 |
| Scly | green4 |
| Ilkap | blue3 |
| Hes6 | blue4 |
| Per2 | darkgrey |
| Traf3ip1 | blue4 |
| Asb1 | blue3 |
| Hdac4 | blue4 |
| Ndufa10 | blue4 |
| Myeov2 | coral1 |
| Gpc1 | green4 |
| Dusp28 | blue4 |
| Rnpepl1 | green4 |
| Capn10 | blue4 |
| 2310007B03Rik | blue3 |
| Sned1 | blue3 |
| Mterf4 | coral1 |
| Ppp1r7 | blue4 |
| Hdlbp | deeppink1 |
| 2-Sep | blue4 |
| Farp2 | blue3 |
| Stk25 | blue4 |
| Bok | blue4 |
| Thap4 | blue4 |
| Atg4b | deeppink |
| Dtymk | blue4 |
| Ing5 | blue4 |
| D2hgdh | blue4 |
| Fam174a | blue4 |
| Gm15427 | blue4 |
| Slco4c1 | darkolivegreen |
| Panct2 | darkolivegreen |
| Ppip5k2 | blue4 |
| Gin1 | coral1 |
| Pam | blue4 |
| Gm3531 | blue4 |
| B230216N24Rik | blue4 |
| Gm24465 | blue3 |
| Tdpx-ps1 | blue4 |
| Gm29668 | blue3 |
| Gm29012 | blue4 |
| Rnf152 | firebrick2 |
| Gm17634 | darkolivegreen |
| Pign | blue4 |
| 2310035C23Rik | blue4 |
| Gm10193 | blue4 |
| Tnfrsf11a | blue4 |
| Gm7160 | blue3 |
| Zcchc2 | darkgrey |
| Phlpp1 | darkgrey |
| Bcl2 | blue3 |
| Kdsr | blue4 |
| Vps4b | coral1 |
| Serpinb8 | blue4 |
| Dsel | blue4 |
| Tsn | blue4 |
| Nifk | blue4 |
| Clasp1 | blue4 |
| Tfcp2l1 | green4 |
| Inhbb | darkgrey |
| Ralb | blue4 |
| Gm27184 | darkolivegreen |
| Epb4.1l5 | blue4 |
| Ptpn4 | firebrick2 |
| Tmem177 | darkgrey |
| Tmem37 | blue3 |
| Dbi | coral1 |
| 3110009E18Rik | blue4 |
| Steap3 | green4 |
| Insig2 | coral1 |
| Ccdc93 | blue3 |
| Ddx18 | blue4 |
| Actr3 | blue4 |
| Slc35f5 | blue4 |
| Nckap5 | lightsteelblue |
| Mgat5 | mistyrose |
| Gm8451 | blue4 |
| Acmsd | blue4 |
| Ccnt2 | blue3 |
| Rab3gap1 | coral1 |
| Rpl28-ps1 | blue4 |
| R3hdm1 | blue4 |
| Ubxn4 | blue4 |
| Mcm6 | blue4 |
| Dars | coral1 |
| Cxcr4 | blue3 |
| Cd55 | deeppink |
| Pfkfb2 | blue3 |
| Yod1 | deeppink1 |
| AA986860 | blue3 |
| Fcamr | blue4 |
| Gm15848 | blue4 |
| Pigr | saddlebrown |
| Mapkapk2 | mistyrose |
| Dyrk3 | blue4 |
| Eif2d | blue3 |
| Rassf5 | darkgrey |
| Ikbke | blue3 |
| Srgap2 | blue3 |
| Ctse | lightcoral |
| 5430435G22Rik | honeydew |
| Pm20d1 | blue4 |
| Slc41a1 | blue4 |
| Rab29 | blue4 |
| Nucks1 | blue4 |
| Slc45a3 | deeppink |
| Elk4 | darkgrey |
| Mfsd4 | blue4 |
| Cdk18 | darkgrey |
| Klhdc8a | blue3 |
| Nuak2 | blue4 |
| Tmcc2 | deeppink1 |
| Dstyk | coral1 |
| Rbbp5 | blue4 |
| Tmem81 | blue3 |
| Mdm4 | blue3 |
| Ppp1r15b | darkgrey |
| Plekha6 | green4 |
| Ren1 | blue4 |
| Etnk2 | blue3 |
| Sox13 | green4 |
| Snrpe | blue4 |
| Zc3h11a | blue4 |
| Atp2b4 | blue3 |
| Prelp | green4 |
| Fmod | green4 |
| Btg2 | coral1 |
| Adora1 | lightcoral |
| Tmem183a | firebrick2 |
| Cyb5r1 | blue3 |
| Adipor1 | firebrick2 |
| Klhl12 | brown1 |
| Rabif | blue4 |
| Gm25612 | blue4 |
| Kdm5b | darkgrey |
| Ppp1r12b | blue3 |
| Arl8a | blue3 |
| Gm26280 | blue3 |
| Gm15445 | blue3 |
| Gm4204 | blue4 |
| Elf3 | mistyrose |
| Rnpep | green4 |
| Timm17a | deeppink |
| Lmod1 | blue4 |
| Shisa4 | blue4 |
| Ipo9 | blue4 |
| Csrp1 | mistyrose |
| Phlda3 | blue4 |
| Lad1 | mistyrose |
| Tmem9 | blue4 |
| 5730559C18Rik | darkgrey |
| Camsap2 | blue4 |
| Ddx59 | mistyrose |
| Zfp281 | coral1 |
| Gm19705 | blue3 |
| Ptprc | blue4 |
| Atp6v1g3 | mistyrose |
| Nek7 | green4 |
| 2310009B15Rik | blue4 |
| Dennd1b | coral1 |
| Zbtb41 | blue4 |
| F13b | mistyrose |
| Cfh | blue4 |
| Gm5835 | blue4 |
| Cdc73 | blue4 |
| Glrx2 | blue3 |
| Trove2 | blue4 |
| Uchl5 | blue4 |
| Rgs2 | darkgrey |
| Pla2g4a | blue4 |
| Ptgs2 | darkgrey |
| Gm15453 | blue4 |
| BC003331 | coral1 |
| Tpr | blue4 |
| Gm8941 | blue3 |
| C730036E19Rik | blue3 |
| Gm10138 | mistyrose |
| Ivns1abp | blue3 |
| Swt1 | brown1 |
| Trmt1l | blue3 |
| RP24-167M10.4 | blue3 |
| Rnf2 | brown1 |
| Gm19503 | blue3 |
| Fam129a | blue4 |
| 2810414N06Rik | blue4 |
| Edem3 | blue4 |
| 1700025G04Rik | blue4 |
| Tsen15 | blue4 |
| Colgalt2 | green4 |
| Rgl1 | blue4 |
| Gm7278 | blue3 |
| Arpc5 | blue4 |
| Ncf2 | honeydew |
| Smg7 | blue4 |
| Lamc2 | blue4 |
| Lamc1 | blue4 |
| Dhx9 | blue4 |
| Npl | blue4 |
| Gm6652 | blue4 |
| Rgs16 | blue4 |
| Rnasel | blue4 |
| Glul | mistyrose |
| Zfp648 | darkgrey |
| Ier5 | darkgrey |
| Mr1 | blue4 |
| Stx6 | blue3 |
| BC034090 | green4 |
| RP23-320I7.3 | darkolivegreen |
| Xpr1 | firebrick2 |
| Acbd6 | blue4 |
| Qsox1 | blue4 |
| Cep350 | blue4 |
| Tor1aip1 | blue4 |
| Tor1aip2 | brown1 |
| Fam163a | blue4 |
| Nphs2 | darkgrey |
| Gm2000 | blue4 |
| Soat1 | blue3 |
| Abl2 | darkgrey |
| Tor3a | blue4 |
| Fam20b | blue3 |
| Ralgps2 | blue4 |
| Angptl1 | blue3 |
| Rasal2 | blue4 |
| 2810025M15Rik | blue4 |
| BC026585 | blue4 |
| Sec16b | darkgrey |
| Rfwd2 | coral1 |
| Gm26472 | blue4 |
| 4930523C07Rik | darkgrey |
| Tnn | blue4 |
| Mrps14 | mistyrose |
| Cacybp | blue4 |
| Rabgap1l | firebrick2 |
| RP24-400M20.2 | darkgrey |
| RP24-400M20.4 | darkgrey |
| Rc3h1 | deeppink |
| Zbtb37 | blue4 |
| Gas5 | blue4 |
| Dars2 | darkgrey |
| Cenpl | saddlebrown |
| Klhl20 | coral1 |
| Ankrd45 | blue3 |
| Prdx6 | blue4 |
| Gm15429 | blue4 |
| Suco | brown1 |
| Pigc | blue3 |
| Dnm3 | blue3 |
| Mettl13 | blue3 |
| Vamp4 | blue3 |
| Prrc2c | blue4 |
| Gm27616 | blue4 |
| Fmo4 | blue4 |
| Fmo1 | darkolivegreen |
| Fmo2 | mistyrose |
| Prrx1 | blue4 |
| Gorab | blue4 |
| Kifap3 | blue4 |
| Scyl3 | mistyrose |
| BC055324 | blue4 |
| Mettl18 | coral1 |
| Sele | darkgrey |
| F5 | blue4 |
| Slc19a2 | blue4 |
| Ccdc181 | firebrick2 |
| Blzf1 | coral1 |
| Nme7 | coral1 |
| Atp1b1 | darkolivegreen |
| RP24-272N10.3 | blue4 |
| RP24-272N10.6 | darkolivegreen |
| Dpt | blue4 |
| RP23-152I6.2 | darkolivegreen |
| RP23-152I6.3 | blue3 |
| Sft2d2 | coral1 |
| Tiprl | brown1 |
| Dcaf6 | firebrick2 |
| Mpc2 | blue4 |
| Mpzl1 | blue4 |
| Rcsd1 | coral1 |
| Creg1 | blue4 |
| Pou2f1 | blue4 |
| Ildr2 | blue4 |
| Tada1 | blue4 |
| Pogk | blue4 |
| Gm16418 | blue3 |
| Uck2 | blue4 |
| Tmco1 | mistyrose |
| Gm25911 | blue4 |
| Gm24182 | blue4 |
| Aldh9a1 | blue4 |
| Mgst3 | indianred2 |
| Lrrc52 | blue3 |
| Pbx1 | blue3 |
| Rgs5 | mistyrose |
| Hsd17b7 | darkolivegreen |
| 3110045C21Rik | darkolivegreen |
| Ddr2 | blue4 |
| Uap1 | darkgrey |
| Uhmk1 | coral1 |
| Gm7694 | green4 |
| Nos1ap | orangered |
| Olfml2b | mistyrose |
| Atf6 | coral1 |
| Gm9929 | blue3 |
| Dusp12 | blue3 |
| Gm2962 | antiquewhite2 |
| Fcgr2b | blue4 |
| RP24-65D16.3 | darkolivegreen |
| RP24-65D16.4 | blue3 |
| Fcgr4 | saddlebrown |
| Fcgr3 | honeydew |
| 1700009P17Rik | blue4 |
| Sdhc | blue4 |
| Pcp4l1 | deeppink |
| Tomm40l | blue3 |
| Apoa2 | blue4 |
| Fcer1g | honeydew |
| Ndufs2 | blue4 |
| Adamts4 | darkgrey |
| B4galt3 | blue4 |
| Ppox | blue3 |
| Usp21 | blue3 |
| Ufc1 | blue4 |
| Dedd | blue3 |
| Nit1 | blue4 |
| Pfdn2 | green4 |
| RP23-191A19.1 | deeppink1 |
| Klhdc9 | blue4 |
| Arhgap30 | honeydew |
| Usf1 | blue3 |
| Tstd1 | blue4 |
| Gm26641 | darkolivegreen |
| F11r | blue4 |
| Cd48 | saddlebrown |
| Tma7-ps | blue4 |
| Vangl2 | blue4 |
| Ncstn | blue4 |
| Copa | blue3 |
| Pex19 | blue4 |
| Dcaf8 | blue4 |
| Pea15a | blue4 |
| Igsf8 | blue3 |
| Atp1a2 | lightcoral |
| Kcnj10 | green4 |
| Pigm | blue4 |
| Slamf9 | blue4 |
| Igsf9 | blue3 |
| Tagln2 | blue4 |
| Dusp23 | green4 |
| Apcs | blue3 |
| AI607873 | darkgrey |
| Ifi204 | honeydew |
| Gm16340 | blue3 |
| Mndal | blue3 |
| Mnda | blue4 |
| Ifi203 | blue4 |
| Grem2 | blue4 |
| Fh1 | deeppink |
| Kmo | blue4 |
| Opn3 | blue4 |
| Chml | blue4 |
| Cep170 | blue4 |
| Sdccag8 | blue4 |
| RP23-293I7.1 | blue4 |
| Akt3 | blue4 |
| Zbtb18 | blue4 |
| 2310043L19Rik | blue3 |
| 1700016C15Rik | blue4 |
| Adss | blue3 |
| RP23-243J5.2 | blue3 |
| Desi2 | darkgrey |
| RP24-252K15.3 | darkgrey |
| Cox20 | darkolivegreen |
| Gm16586 | blue3 |
| Hnrnpu | blue4 |
| Efcab2 | deeppink |
| Gm5561 | blue4 |
| Tfb2m | coral1 |
| Cnst | blue3 |
| Sccpdh | coral1 |
| Ahctf1 | blue4 |
| Cdc42bpa | blue4 |
| Adck3 | blue4 |
| RP23-226J18.4 | blue4 |
| Psen2 | deeppink |
| RP23-226J18.5 | darkolivegreen |
| Gm5069 | darkolivegreen |
| Itpkb | coral1 |
| Parp1 | blue3 |
| Lin9 | coral1 |
| Acbd3 | blue4 |
| RP23-201I6.5 | blue3 |
| H3f3a | green4 |
| Gm17275 | blue3 |
| Sde2 | darkgrey |
| Pycr2 | blue4 |
| Lefty1 | blue4 |
| Tmem63a | blue4 |
| Ephx1 | blue4 |
| Nvl | blue4 |
| Cnih4 | blue4 |
| Wdr26 | brown1 |
| A430110L20Rik | blue4 |
| Rpl35a-ps2 | blue4 |
| Lbr | blue4 |
| Enah | blue4 |
| Srp9 | blue3 |
| Degs1 | blue4 |
| Fbxo28 | blue4 |
| Trp53bp2 | blue4 |
| Capn2 | blue4 |
| Susd4 | darkolivegreen |
| CAAA01116243.1 | blue4 |
| Disp1 | blue3 |
| Brox | coral1 |
| Aida | blue4 |
| Mia3 | coral1 |
| Taf1a | blue4 |
| Dusp10 | darkgrey |
| 1700056E22Rik | darkgrey |
| Hlx | mistyrose |
| 2-Mar | blue4 |
| Gm23780 | darkgrey |
| C130074G19Rik | blue3 |
| Mark1 | blue4 |
| Rab3gap2 | darkgrey |
| Iars2 | coral1 |
| Mir194-1 | blue4 |
| Bpnt1 | blue4 |
| Eprs | blue4 |
| Lyplal1 | darkolivegreen |
| Rrp15 | blue4 |
| Spata17 | blue4 |
| Gpatch2 | blue3 |
| RP23-306B14.3 | blue4 |
| RP23-349L18.2 | blue3 |
| Esrrg | firebrick2 |
| Kctd3 | blue4 |
| RP23-411B11.2 | blue4 |
| Ptpn14 | blue3 |
| RP24-288J3.2 | blue3 |
| Smyd2 | blue4 |
| Prox1 | blue3 |
| Rps6kc1 | blue4 |
| Angel2 | blue3 |
| Mfsd7b | green4 |
| Tatdn3 | blue3 |
| Batf3 | blue3 |
| Atf3 | darkgrey |
| D730003I15Rik | darkolivegreen |
| Nenf | blue4 |
| Tmem206 | blue4 |
| Ppp2r5a | mistyrose |
| Dtl | blue4 |
| Ints7 | blue4 |
| Lpgat1 | firebrick2 |
| Gm26203 | darkolivegreen |
| Nek2 | blue4 |
| 1700034H15Rik | blue3 |
| Slc30a1 | mistyrose |
| Traf5 | blue4 |
| WI1-1003N17.1 | blue4 |
| Rcor3 | blue3 |
| Gm10516 | darkolivegreen |
| Hhat | blue4 |
| Sertad4 | blue4 |
| Gm15867 | blue4 |
| Syt14 | blue4 |
| RP23-95L22.2 | blue4 |
| Diexf | blue4 |
| Irf6 | deeppink |
| A130010J15Rik | saddlebrown |
| Lamb3 | blue4 |
| Hsd11b1 | mistyrose |
| G0s2 | mistyrose |
| Camk1g | blue4 |
| Plxna2 | blue4 |
| Cd34 | blue4 |
| A330023F24Rik | darkolivegreen |
| RP23-445K23.6 | blue4 |
| Cd46 | blue3 |
| Cr1l | blue4 |
| Fam171a1 | blue3 |
| Nmt2 | blue4 |
| Rpp38 | blue4 |
| Dclre1c | blue3 |
| Hspa14 | blue4 |
| Fam107b | darkgrey |
| Frmd4a | blue4 |
| Prpf18 | coral1 |
| Bend7 | firebrick2 |
| Sephs1 | darkgrey |
| Phyh | blue4 |
| Gm13194 | darkolivegreen |
| Gm13193 | blue4 |
| Gm13192 | blue4 |
| Mcm10 | darkolivegreen |
| Optn | blue4 |
| Cdc123 | coral1 |
| Nudt5 | blue4 |
| Gm13199 | blue3 |
| Sec61a2 | blue4 |
| Dhtkd1 | blue4 |
| Upf2 | blue4 |
| Proser2 | coral1 |
| Echdc3 | blue4 |
| Usp6nl | blue4 |
| Gata3 | blue3 |
| 4930412O13Rik | blue3 |
| Taf3 | blue3 |
| Atp5c1 | blue4 |
| Gm23608 | blue3 |
| Kin | coral1 |
| Itih2 | blue4 |
| Itih5 | blue4 |
| Prkcq | blue3 |
| Pfkfb3 | darkgrey |
| Rbm17 | blue4 |
| Il15ra | blue3 |
| Fbxo18 | blue3 |
| Ankrd16 | blue3 |
| Itga8 | blue4 |
| Fam188a | coral1 |
| Pter | darkolivegreen |
| C1ql3 | blue4 |
| RP23-415C3.6 | darkolivegreen |
| Rsu1 | blue4 |
| Cubn | darkolivegreen |
| Trdmt1 | coral1 |
| Vim | blue4 |
| St8sia6 | blue4 |
| Ptpla | blue4 |
| Stamos | darkolivegreen |
| Stam | green4 |
| Gm13320 | honeydew |
| Mrc1 | blue4 |
| Nsun6 | blue4 |
| Arl5b | darkgrey |
| Malrd1 | firebrick2 |
| Plxdc2 | firebrick2 |
| A930004D18Rik | blue3 |
| Skida1 | blue3 |
| 1810059C17Rik | blue3 |
| Mllt10 | blue3 |
| Mir7655 | darkgrey |
| Dnajc1 | blue4 |
| Commd3 | coral1 |
| Bmi1 | coral1 |
| Pip4k2a | blue4 |
| Msrb2 | darkolivegreen |
| Otud1 | blue4 |
| Etl4 | blue3 |
| Arhgap21 | blue4 |
| Gm13375 | blue4 |
| Thnsl1 | firebrick2 |
| Gm13342 | blue4 |
| Gm13341 | coral1 |
| Gm13340 | deeppink |
| Gm13339 | coral1 |
| Apbb1ip | blue4 |
| A130006I12Rik | blue3 |
| Pdss1 | blue4 |
| Abi1 | blue4 |
| Acbd5 | mistyrose |
| Yme1l1 | coral1 |
| Spopl | blue4 |
| Gm13392 | darkgrey |
| Hnmt | mistyrose |
| Pax8 | darkgrey |
| Ehmt1 | blue4 |
| Arrdc1 | blue3 |
| Zmynd19 | blue4 |
| Dph7 | blue4 |
| Mrpl41 | mistyrose |
| Pnpla7 | blue4 |
| Nsmf | blue3 |
| Mir7664 | mistyrose |
| Entpd8 | blue4 |
| Tor4a | blue3 |
| Nelfb | blue3 |
| Gm22572 | blue3 |
| Tubb4b | darkgrey |
| Slc34a3 | blue4 |
| Rnf208 | green4 |
| Ndor1 | blue3 |
| Tmem203 | coral1 |
| Tprn | darkgrey |
| Ssna1 | blue4 |
| Anapc2 | deeppink |
| Lrrc26 | lightcoral |
| Man1b1 | green4 |
| Gm25220 | blue3 |
| Dpp7 | blue4 |
| Uap1l1 | blue4 |
| Entpd2 | blue4 |
| Npdc1 | blue4 |
| Abca2 | green4 |
| BC029214 | green4 |
| Clic3 | green4 |
| Ptgds | coral1 |
| C8g | blue4 |
| Fbxw5 | green4 |
| Traf2 | blue4 |
| Edf1 | deeppink |
| Mamdc4 | blue3 |
| Rabl6 | deeppink |
| Tmem141 | blue4 |
| Bmyc | darkgrey |
| Kcnt1 | darkolivegreen |
| Camsap1 | blue4 |
| Ubac1 | blue3 |
| Nacc2 | blue3 |
| C330006A16Rik | green4 |
| 1810012K08Rik | blue3 |
| Qsox2 | blue3 |
| Gpsm1 | darkgrey |
| Dnlz | blue4 |
| Gm13562 | blue3 |
| Snapc4 | blue3 |
| Gm13563 | blue3 |
| Sdccag3 | blue4 |
| Pmpca | blue3 |
| Inpp5e | green4 |
| Sec16a | blue3 |
| Notch1 | mistyrose |
| Egfl7 | blue4 |
| Agpat2 | blue4 |
| Fam69b | green4 |
| Snhg7 | blue4 |
| Surf6 | blue4 |
| Med22 | blue3 |
| Rpl7a | blue4 |
| Gm23969 | darkgrey |
| Surf1 | blue4 |
| Surf2 | blue3 |
| Surf4 | blue4 |
| Rexo4 | blue4 |
| Cacfd1 | blue3 |
| Adamtsl2 | mistyrose |
| Sardh | blue4 |
| Vav2 | blue4 |
| Brd3 | blue3 |
| Wdr5 | blue4 |
| Gm13421 | blue3 |
| Rxra | blue4 |
| Col5a1 | blue4 |
| Olfm1 | blue3 |
| Ppp1r26 | blue3 |
| 1700007K13Rik | green4 |
| Mrps2 | blue3 |
| Ralgds | darkgrey |
| Gtf3c5 | blue3 |
| Tsc1 | deeppink |
| Gtf3c4 | coral1 |
| Ddx31 | blue4 |
| Ttf1 | coral1 |
| Setx | blue4 |
| Ntng2 | green4 |
| 6530402F18Rik | blue4 |
| Med27 | blue4 |
| Rapgef1 | blue4 |
| Gm13420 | blue3 |
| Trub2 | darkgrey |
| Coq4 | deeppink |
| Slc27a4 | deeppink |
| Urm1 | deeppink |
| Odf2 | blue4 |
| Gle1 | green4 |
| Sptan1 | blue4 |
| Wdr34 | blue4 |
| Set | blue4 |
| Pkn3 | blue4 |
| Zdhhc12 | blue4 |
| Zer1 | green4 |
| Tbc1d13 | blue4 |
| Endog | blue4 |
| D2Wsu81e | blue3 |
| Ccbl1 | blue4 |
| Lrrc8a | coral1 |
| Phyhd1 | blue3 |
| Dolk | brown1 |
| Nup188 | blue4 |
| Sh3glb2 | blue3 |
| Fam73b | green4 |
| Dolpp1 | blue4 |
| Crat | blue4 |
| Ppp2r4 | deeppink |
| Ier5l | blue4 |
| 1700001O22Rik | blue3 |
| Ntmt1 | blue4 |
| Asb6 | darkgrey |
| Ptges | mistyrose |
| Tor1b | blue4 |
| Tor1a | blue4 |
| BC005624 | blue3 |
| Usp20 | blue3 |
| Fnbp1 | blue3 |
| D330023K18Rik | blue4 |
| Gpr107 | blue3 |
| Gm22516 | blue3 |
| Ncs1 | blue4 |
| Hmcn2 | antiquewhite2 |
| Ass1 | blue4 |
| Fubp3 | blue4 |
| Exosc2 | blue4 |
| Abl1 | blue3 |
| Lamc3 | coral1 |
| Aif1l | blue3 |
| Nup214 | blue3 |
| Fam78a | blue3 |
| Ppapdc3 | green4 |
| Prrc2b | blue3 |
| Gm22192 | blue4 |
| Gm25632 | blue4 |
| Pomt1 | green4 |
| Uck1 | blue4 |
| Gm13611 | blue4 |
| Swi5 | antiquewhite2 |
| Golga2 | blue4 |
| Dnm1 | blue3 |
| Ciz1 | blue3 |
| 1110008P14Rik | blue4 |
| Lcn2 | blue4 |
| Ptges2 | deeppink |
| Slc25a25 | darkgrey |
| Naif1 | deeppink |
| Gm13412 | blue4 |
| Fam102a | mistyrose |
| Dpm2 | blue4 |
| St6galnac4 | mistyrose |
| St6galnac6 | blue4 |
| Ak1 | green4 |
| Eng | blue4 |
| Mir1954 | blue4 |
| Gm26236 | blue4 |
| Fpgs | blue4 |
| Cdk9 | darkgrey |
| Sh2d3c | mistyrose |
| Tor2a | blue4 |
| Ptrh1 | blue4 |
| 1700019L03Rik | blue4 |
| Stxbp1 | mistyrose |
| Fam129b | blue4 |
| Lrsam1 | blue3 |
| Rpl12 | blue4 |
| Slc2a8 | blue4 |
| Ralgps1 | blue4 |
| Gm13528 | darkolivegreen |
| Angptl2 | blue4 |
| Zbtb34 | blue4 |
| Zbtb43 | darkgrey |
| C130021I20Rik | blue3 |
| Mvb12b | green4 |
| Pbx3 | blue4 |
| Mapkap1 | firebrick2 |
| Gapvd1 | blue4 |
| Hspa5 | darkgrey |
| Rabepk | blue3 |
| Fbxw2 | coral1 |
| Psmd5 | blue4 |
| Cutal | darkolivegreen |
| Traf1 | blue4 |
| Cntrl | blue3 |
| Rab14 | firebrick2 |
| Gsn | green4 |
| Stom | blue4 |
| Gm13443 | blue4 |
| Ggta1 | blue4 |
| Dab2ip | blue4 |
| Ttll11 | deeppink1 |
| Ndufa8 | green4 |
| Rbm18 | green4 |
| Mrrf | blue4 |
| Gm13431 | blue3 |
| Ptgs1 | blue4 |
| Gm13436 | blue4 |
| Pdcl | coral1 |
| Rc3h2 | blue4 |
| Zbtb6 | blue4 |
| Zbtb26 | blue3 |
| Rabgap1 | darkgrey |
| Strbp | coral1 |
| Crb2 | blue3 |
| Dennd1a | mistyrose |
| Nek6 | blue4 |
| Psmb7 | orangered |
| Nr6a1 | darkgrey |
| Rpl35 | blue4 |
| Arpc5l | blue4 |
| Golga1 | blue3 |
| Scai | firebrick2 |
| Ppp6c | green4 |
| Gm13453 | blue4 |
| Gm13456 | blue4 |
| Gm13461 | blue4 |
| Kynu | blue3 |
| Arhgap15 | blue4 |
| Gtdc1 | coral1 |
| Zeb2 | blue4 |
| Acvr2a | coral1 |
| Orc4 | coral1 |
| Mbd5 | blue4 |
| Gm13509 | blue4 |
| Epc2 | brown1 |
| Lypd6b | lightcoral |
| Lypd6 | darkolivegreen |
| Mmadhc | green4 |
| Gm13483 | firebrick2 |
| Rnd3 | darkgrey |
| Gm13493 | blue4 |
| Rbm43 | blue4 |
| Nmi | saddlebrown |
| Tnfaip6 | darkgrey |
| Rif1 | blue4 |
| Arl5a | coral1 |
| Cacnb4 | darkolivegreen |
| Bloc1s2-ps | blue4 |
| Stam2 | blue4 |
| Fmnl2 | blue4 |
| Prpf40a | coral1 |
| Arl6ip6 | blue4 |
| Nr4a2 | darkgrey |
| Gpd2 | mistyrose |
| Cytip | blue3 |
| Acvr1 | mistyrose |
| Upp2 | mistyrose |
| Pkp4 | darkolivegreen |
| Gm13552 | blue4 |
| Tanc1 | blue3 |
| Wdsub1 | blue4 |
| Baz2b | deeppink1 |
| 7-Mar | brown1 |
| Cd302 | green4 |
| Itgb6 | blue4 |
| Rbms1 | blue4 |
| Tank | blue4 |
| Psmd14 | blue4 |
| Gm22403 | blue4 |
| Gm25966 | darkgrey |
| Dpp4 | firebrick2 |
| Ifih1 | blue4 |
| Gca | blue3 |
| Fign | firebrick2 |
| Gm13577 | blue4 |
| Grb14 | blue3 |
| Cobll1 | blue4 |
| Gm13594 | mistyrose |
| Csrnp3 | blue3 |
| Galnt3 | lightsteelblue |
| Ttc21b | blue3 |
| Stk39 | blue4 |
| Gm13597 | blue4 |
| Cers6 | firebrick2 |
| Nostrin | darkgrey |
| Spc25 | blue4 |
| Lrp2 | darkolivegreen |
| Bbs5 | antiquewhite2 |
| Fastkd1 | darkolivegreen |
| Ppig | blue4 |
| Ccdc173 | blue3 |
| Phospho2 | mistyrose |
| Ssb | coral1 |
| Mettl5 | darkgrey |
| Ubr3 | coral1 |
| Myo3b | blue4 |
| Rpl9-ps7 | blue4 |
| Sp5 | coral1 |
| Erich2os | blue4 |
| Erich2 | darkolivegreen |
| Gorasp2 | blue4 |
| Tlk1 | coral1 |
| Mettl8 | blue4 |
| Dcaf17 | blue3 |
| Dync1i2 | blue4 |
| Slc25a12 | mistyrose |
| Hat1 | blue4 |
| Metap1d | blue3 |
| Dlx1as | blue3 |
| Itga6 | blue4 |
| Pdk1 | mistyrose |
| Rapgef4 | blue4 |
| Zak | blue4 |
| Cdca7 | blue4 |
| Ak3l2-ps | mistyrose |
| Sp3 | green4 |
| Gm11084 | blue3 |
| Sp3os | blue4 |
| Ola1 | blue4 |
| Cir1 | mistyrose |
| Scrn3 | mistyrose |
| Gpr155 | firebrick2 |
| Wipf1 | blue4 |
| Chn1os3 | blue3 |
| Chn1 | blue4 |
| Atf2 | coral1 |
| Atp5g3 | blue4 |
| Lnp | blue4 |
| Mrpl23-ps1 | blue4 |
| Hoxd10 | blue3 |
| Gm28793 | blue3 |
| Hoxd9 | green4 |
| Hoxd3os1 | darkolivegreen |
| Hoxd8 | blue3 |
| Hoxd4 | blue3 |
| Mtx2 | coral1 |
| Gm13652 | blue3 |
| Rps6-ps4 | blue4 |
| Gm13655 | blue3 |
| Hnrnpa3 | blue4 |
| Nfe2l2 | blue4 |
| Gm24996 | blue4 |
| E030042O20Rik | blue3 |
| Gm13669 | blue3 |
| Agps | blue3 |
| Ttc30b | darkolivegreen |
| Ttc30a1 | blue3 |
| Rbm45 | blue3 |
| Osbpl6 | firebrick2 |
| Prkra | blue3 |
| Fkbp7 | darkolivegreen |
| Plekha3 | blue4 |
| Ccdc141 | blue3 |
| Sestd1 | blue4 |
| Zfp385b | darkgrey |
| Cwc22 | blue4 |
| Ube2e3 | blue4 |
| Ssfa2 | brown1 |
| Pde1a | mistyrose |
| Dnajc10 | coral1 |
| Frzb | blue4 |
| Nckap1 | blue4 |
| Dusp19 | blue3 |
| Nup35 | blue4 |
| Gm4735 | blue4 |
| Gm13680 | blue4 |
| Zc3h15 | coral1 |
| Itgav | blue4 |
| Calcrl | blue4 |
| Tfpi | blue4 |
| Ctnnd1 | deeppink |
| 2700094K13Rik | blue4 |
| Tmx2 | mistyrose |
| Med19 | blue4 |
| Zdhhc5 | blue3 |
| Gm27942 | blue3 |
| Clp1 | green4 |
| Gm19426 | green4 |
| Serping1 | darkgrey |
| Ube2l6 | saddlebrown |
| Timm10 | blue4 |
| Slc43a1 | mistyrose |
| Rtn4rl2 | darkgrey |
| Slc43a3 | darkolivegreen |
| Ssrp1 | blue4 |
| Tnks1bp1 | blue4 |
| Aplnr | green4 |
| Olfr1033 | blue4 |
| Gm13736 | blue4 |
| Ptprj | blue4 |
| Nup160 | blue4 |
| Fnbp4 | blue4 |
| Gm13772 | darkgrey |
| Mtch2 | coral1 |
| C1qtnf4 | blue4 |
| Ndufs3 | blue4 |
| Kbtbd4 | blue3 |
| Ptpmt1 | blue3 |
| Celf1 | blue4 |
| Rapsn | green4 |
| Gm13778 | blue3 |
| Psmc3 | blue4 |
| Slc39a13 | blue3 |
| Spi1 | honeydew |
| Madd | blue3 |
| Nr1h3 | green4 |
| Acp2 | blue3 |
| Ddb2 | blue3 |
| A330069E16Rik | blue3 |
| Pacsin3 | blue4 |
| Arfgap2 | indianred2 |
| 1110051M20Rik | green4 |
| Lrp4 | darkolivegreen |
| Ckap5 | blue4 |
| F2 | blue4 |
| Zfp408 | blue4 |
| Arhgap1 | blue4 |
| Atg13 | blue4 |
| Harbi1 | mistyrose |
| Ambra1 | green4 |
| Mdk | blue4 |
| Dgkz | green4 |
| Creb3l1 | blue4 |
| Phf21a | blue3 |
| Gyltl1b | green4 |
| Pex16 | blue4 |
| 1700029I15Rik | green4 |
| Mapk8ip1 | coral1 |
| Cry2 | darkgrey |
| D930015M05Rik | darkgrey |
| Slc35c1 | blue4 |
| Chst1 | blue4 |
| Syt13 | coral1 |
| Trp53i11 | blue4 |
| Tspan18 | blue3 |
| Gm13807 | blue4 |
| Cd82 | blue4 |
| Mir7001 | darkgrey |
| Gm10804 | darkolivegreen |
| Ext2 | blue4 |
| Accs | darkgrey |
| Gm13889 | darkgrey |
| Alkbh3 | mistyrose |
| Hsd17b12 | blue4 |
| E530001K10Rik | blue4 |
| Ttc17 | blue4 |
| Itpa-ps1 | blue4 |
| 2810002D19Rik | blue3 |
| Api5 | blue4 |
| B230118H07Rik | mistyrose |
| Traf6 | brown1 |
| Prr5l | blue4 |
| Commd9 | green4 |
| Ldlrad3 | blue3 |
| Trim44 | blue4 |
| Fjx1 | blue4 |
| Pamr1 | green4 |
| Cd44 | blue4 |
| Pdhx | coral1 |
| Apip | coral1 |
| Ehf | blue4 |
| Elf5 | lightcoral |
| Cat | darkolivegreen |
| Abtb2 | darkgrey |
| Nat10 | blue4 |
| Caprin1 | blue4 |
| Lmo2 | green4 |
| 4931422A03Rik | blue3 |
| Fbxo3 | mistyrose |
| Gm24644 | deeppink1 |
| Cd59b | blue4 |
| Cd59a | coral1 |
| Hipk3 | deeppink |
| Cstf3 | blue4 |
| Tcp11l1 | blue4 |
| Depdc7 | mistyrose |
| Qser1 | blue4 |
| Prrg4 | darkgrey |
| Eif3m | mistyrose |
| Wt1 | mistyrose |
| Them7 | mistyrose |
| Rcn1 | blue4 |
| Elp4 | coral1 |
| Immp1l | darkgrey |
| Dnajc24 | blue4 |
| BB218582 | darkolivegreen |
| Mpped2 | darkolivegreen |
| Arl14ep | firebrick2 |
| Rpl35a-ps4 | lightsteelblue |
| Gm13910 | blue4 |
| Mettl15 | darkgrey |
| Bdnf | blue4 |
| Lin7c | coral1 |
| Lgr4 | blue3 |
| Ccdc34 | blue4 |
| Fibin | mistyrose |
| Slc5a12 | blue4 |
| Ano3 | blue3 |
| Lpcat4 | blue4 |
| Nop10 | blue4 |
| Slc12a6 | darkolivegreen |
| Emc4 | blue4 |
| Katnbl1 | blue4 |
| Emc7 | mistyrose |
| Aven | blue3 |
| Fmn1 | blue4 |
| Scg5 | blue4 |
| Arhgap11a | blue4 |
| Aqr | blue4 |
| Zfp770 | coral1 |
| Dph6 | blue4 |
| 3110099E03Rik | blue3 |
| BC052040 | blue4 |
| Meis2 | blue4 |
| Gm13991 | blue4 |
| Spred1 | blue4 |
| Fam98b | blue4 |
| Rasgrp1 | blue4 |
| Thbs1 | darkgrey |
| Gpr176 | blue4 |
| Eif2ak4 | blue4 |
| Srp14 | blue4 |
| Bmf | darkgrey |
| Pak6 | blue3 |
| Inafm2 | darkgrey |
| A430105I19Rik | blue4 |
| Ivd | blue4 |
| Bahd1 | mistyrose |
| Chst14 | blue4 |
| Gm14088 | indianred2 |
| Ccdc32 | blue3 |
| Rpusd2 | blue4 |
| Rad51 | blue4 |
| Rmdn3 | blue4 |
| Gchfr | blue4 |
| Dnajc17 | blue4 |
| Zfyve19 | blue3 |
| Ppp1r14d | blue3 |
| Spint1 | darkgrey |
| Rhov | green4 |
| Vps18 | green4 |
| Gm14207 | green4 |
| Dll4 | green4 |
| Chac1 | darkgrey |
| Ino80 | blue4 |
| Chp1 | blue4 |
| 1700020I14Rik | coral1 |
| Oip5 | antiquewhite2 |
| Nusap1 | mistyrose |
| Ndufaf1 | deeppink |
| H3f3c | blue4 |
| Rtf1 | coral1 |
| Rpap1 | green4 |
| Mga | blue4 |
| Mapkbp1 | blue4 |
| Ehd4 | blue4 |
| Vps39 | blue3 |
| Tmem87a | deeppink |
| Ganc | darkolivegreen |
| Zfp106 | firebrick2 |
| Snap23 | blue4 |
| Lrrc57 | blue4 |
| Haus2 | green4 |
| Stard9 | blue4 |
| Cdan1 | blue3 |
| Ttbk2 | darkgrey |
| Ubr1 | blue4 |
| Tmem62 | blue4 |
| Ccndbp1 | blue3 |
| Tgm7 | blue4 |
| Lcmt2 | coral1 |
| Adal | blue3 |
| Zscan29 | blue3 |
| Tubgcp4 | blue4 |
| Trp53bp1 | blue3 |
| Ppip5k1 | blue4 |
| Ckmt1 | green4 |
| Catsper2 | darkolivegreen |
| Pdia3 | blue4 |
| Serf2 | coral1 |
| Mfap1b | green4 |
| Gm14017 | blue4 |
| Gm14018 | coral1 |
| Mfap1a | coral1 |
| Wdr76 | blue4 |
| Casc4 | mistyrose |
| Ctdspl2 | brown1 |
| Eif3j1 | blue4 |
| Spg11 | blue4 |
| B2m | saddlebrown |
| Sord | blue4 |
| Shf | deeppink |
| Gatm | blue4 |
| Spata5l1 | blue3 |
| AA467197 | blue4 |
| Slc30a4 | blue4 |
| Bloc1s6 | darkgrey |
| Sqrdl | mistyrose |
| Sema6d | darkgrey |
| Myef2 | blue4 |
| Gm22352 | blue4 |
| Slc12a1 | blue4 |
| Dut | blue4 |
| Fbn1 | blue4 |
| Cep152 | blue3 |
| Eid1 | blue4 |
| Secisbp2l | deeppink |
| Cops2 | coral1 |
| Galk2 | mistyrose |
| Dtwd1 | blue4 |
| Gm26697 | mistyrose |
| Slc27a2 | mistyrose |
| Hdc | blue3 |
| Gabpb1 | blue4 |
| Gm27003 | blue3 |
| Usp8 | firebrick2 |
| Trpm7 | firebrick2 |
| Sppl2a | coral1 |
| Mir3473g | lightsteelblue |
| Ap4e1 | blue4 |
| Blvra | blue4 |
| Itpripl1 | blue3 |
| Snrnp200 | lightsteelblue |
| Ciao1 | darkgrey |
| Tmem127 | blue3 |
| RP23-206D14.7 | firebrick2 |
| Stard7 | mistyrose |
| Dusp2 | darkgrey |
| Adra2b | blue4 |
| Gpat2 | blue3 |
| Fahd2a | blue4 |
| Kcnip3 | blue4 |
| Prom2 | green4 |
| Zfp661 | blue3 |
| Mrps5 | blue3 |
| Mal | blue4 |
| Mall | blue4 |
| Nphp1 | mistyrose |
| 1500011K16Rik | deeppink1 |
| Bcl2l11 | brown1 |
| Anapc1 | blue4 |
| Mertk | deeppink |
| Gm23172 | blue3 |
| Tmem87b | coral1 |
| Fbln7 | coral1 |
| Zc3h8 | blue4 |
| Gm10762 | blue4 |
| Zc3h6 | mistyrose |
| Gm14027 | darkolivegreen |
| Ttl | blue4 |
| Polr1b | blue4 |
| Chchd5 | darkolivegreen |
| RP23-160G19.10 | blue4 |
| Slc20a1 | darkgrey |
| 9830144P21Rik | darkgrey |
| Gm25703 | blue3 |
| Il1b | blue4 |
| Spcs2-ps | blue4 |
| Sirpa | green4 |
| Stk35 | darkgrey |
| Snrpb | blue4 |
| Nop56 | blue4 |
| Idh3b | mistyrose |
| Ebf4 | blue3 |
| Cpxm1 | coral1 |
| Pced1a | blue3 |
| Vps16 | blue3 |
| Ptpra | blue4 |
| Mrps26 | blue3 |
| Ubox5 | darkgrey |
| Fastkd5 | blue4 |
| Lzts3 | green4 |
| Ddrgk1 | blue4 |
| Itpa | blue4 |
| Slc4a11 | blue3 |
| 4930402H24Rik | blue3 |
| Gm14057 | blue3 |
| A730017L22Rik | blue3 |
| Atrn | firebrick2 |
| Hspa12b | blue4 |
| 1700037H04Rik | blue3 |
| Spef1 | blue3 |
| Cenpb | deeppink |
| Cdc25b | blue3 |
| Ap5s1 | deeppink |
| Mavs | blue3 |
| Pank2 | blue4 |
| Mir103-2 | blue4 |
| Rnf24 | blue4 |
| Gm14284 | coral1 |
| Smox | blue4 |
| Prnp | green4 |
| Rassf2 | green4 |
| Slc23a2 | darkgrey |
| Tmem230 | blue3 |
| Pcna | blue4 |
| Cds2 | blue4 |
| Gpcpd1 | blue3 |
| 1110034G24Rik | blue3 |
| Trmt6 | blue4 |
| Mcm8 | blue4 |
| Crls1 | coral1 |
| Fermt1 | blue4 |
| Bmp2 | mistyrose |
| Tmx4 | firebrick2 |
| Plcb1 | firebrick2 |
| Plcb4 | blue4 |
| Mkks | mistyrose |
| Slx4ip | blue4 |
| Jag1 | blue3 |
| Btbd3 | blue4 |
| Tasp1 | mistyrose |
| Esf1 | coral1 |
| Ndufaf5 | blue4 |
| Macrod2 | blue4 |
| Flrt3 | darkgrey |
| Kif16b | mistyrose |
| Snrpb2 | blue4 |
| Dstn | blue4 |
| Rrbp1 | blue4 |
| Snx5 | green4 |
| Mgme1 | blue3 |
| Ovol2 | honeydew |
| Csrp2bp | blue3 |
| Zfp133-ps | blue3 |
| Dzank1 | darkolivegreen |
| Polr3f | coral1 |
| Rbbp9 | blue3 |
| Sec23b | blue3 |
| Gm561 | darkgrey |
| Dtd1 | blue3 |
| Rin2 | green4 |
| Naa20 | blue4 |
| Crnkl1 | coral1 |
| Ralgapa2 | coral1 |
| Kiz | deeppink |
| Xrn2 | coral1 |
| Thbd | blue4 |
| Cd93 | darkgrey |
| Nxt1 | blue4 |
| Gzf1 | green4 |
| Napb | deeppink1 |
| Cst3 | firebrick2 |
| Zfp120 | coral1 |
| Zfp937 | blue3 |
| 3300002I08Rik | blue3 |
| Gm10130 | blue4 |
| Zfp442 | blue4 |
| Apmap | deeppink |
| Acss1 | blue4 |
| E130215H24Rik | blue4 |
| Entpd6 | blue3 |
| Pygb | blue3 |
| Abhd12 | deeppink |
| Gins1 | blue4 |
| Ninl | blue3 |
| Nsfl1c | blue4 |
| Gm14167 | green4 |
| Fkbp1a | blue4 |
| Sdcbp2 | darkgrey |
| Psmf1 | blue4 |
| Fam110a | deeppink |
| Slc52a3 | deeppink |
| Srxn1 | blue4 |
| Tcf15 | blue4 |
| Rps15a-ps7 | blue4 |
| Tbc1d20 | deeppink |
| Rbck1 | deeppink |
| Trib3 | blue4 |
| Gm14165 | blue4 |
| Sox12 | blue3 |
| Zcchc3 | blue3 |
| 6820408C15Rik | blue3 |
| Gm27343 | blue3 |
| Defb29 | blue4 |
| Gm14161 | blue4 |
| Gm17416 | blue4 |
| Defb19 | darkgrey |
| Rem1 | blue4 |
| H13 | deeppink |
| Mcts2 | blue4 |
| Id1 | darkolivegreen |
| Cox4i2 | coral1 |
| Gm23802 | darkgrey |
| Bcl2l1 | darkgrey |
| Foxs1 | blue4 |
| Dusp15 | darkolivegreen |
| Pdrg1 | blue4 |
| Ccm2l | blue3 |
| Hck | honeydew |
| Tm9sf4 | blue3 |
| Plagl2 | blue4 |
| Pofut1 | blue3 |
| Kif3b | blue4 |
| 2500004C02Rik | blue3 |
| Asxl1 | blue4 |
| Nol4l | mistyrose |
| Commd7 | blue4 |
| Dnmt3b | blue3 |
| Mapre1 | blue4 |
| Gm14494 | blue4 |
| Cdk5rap1 | darkgrey |
| Snta1 | deeppink |
| Cbfa2t2 | blue3 |
| E2f1 | blue4 |
| Pxmp4 | mistyrose |
| Zfp341 | blue3 |
| Chmp4b | blue4 |
| Raly | mistyrose |
| Eif2s2 | blue4 |
| Ahcy | blue4 |
| Itch | mistyrose |
| Dynlrb1 | blue4 |
| Map1lc3a | coral1 |
| Pigu | blue4 |
| Trp53inp2 | coral1 |
| Ncoa6 | blue4 |
| Acss2 | blue4 |
| Gss | blue4 |
| Gssos1 | blue4 |
| Gm14257 | darkolivegreen |
| Trpc4ap | darkgrey |
| Edem2 | deeppink |
| Procr | blue4 |
| BC029722 | blue4 |
| Eif6 | blue4 |
| Uqcc1 | darkolivegreen |
| Cep250 | blue3 |
| Ergic3 | coral1 |
| Rbm12 | blue4 |
| Nfs1 | blue4 |
| Romo1 | blue4 |
| Rbm39 | brown1 |
| Phf20 | blue4 |
| Scand1 | coral1 |
| Cnbd2 | blue4 |
| Gm14253 | blue4 |
| RP24-114E18.7 | deeppink |
| Epb4.1l1 | blue3 |
| Aar2 | coral1 |
| Dlgap4 | blue4 |
| Myl9 | blue4 |
| Gm14230 | blue4 |
| 4930518I15Rik | blue3 |
| Gm27651 | blue3 |
| 1110008F13Rik | blue4 |
| Ndrg3 | darkolivegreen |
| Gm14276 | blue4 |
| Dsn1 | blue4 |
| Soga1 | deeppink |
| Tldc2 | darkolivegreen |
| Samhd1 | blue4 |
| Rbl1 | blue4 |
| Rpn2 | deeppink |
| Manbal | blue4 |
| Src | blue4 |
| Gm14287 | blue4 |
| Gm14286 | darkgrey |
| Blcap | blue4 |
| Nnat | blue4 |
| Ctnnbl1 | deeppink |
| Tti1 | blue4 |
| Rprd1b | blue4 |
| Tgm2 | blue4 |
| D630003M21Rik | blue3 |
| Lbp | blue4 |
| 9430008C03Rik | blue4 |
| Snhg11 | darkolivegreen |
| Gm25129 | blue3 |
| Ralgapb | blue4 |
| Gm24379 | blue4 |
| Adig | lightcoral |
| Actr5 | blue3 |
| Ppp1r16b | blue4 |
| Dhx35 | blue3 |
| Mafb | blue4 |
| Top1 | blue4 |
| Plcg1 | blue3 |
| Zhx3 | blue3 |
| Lpin3 | blue4 |
| Chd6 | firebrick2 |
| Gm11451 | blue4 |
| Srsf6 | mistyrose |
| Sgk2 | blue3 |
| Ift52 | mistyrose |
| Mybl2 | blue3 |
| Jph2 | coral1 |
| Oser1 | darkgrey |
| 2900093K20Rik | blue4 |
| Fitm2 | blue4 |
| R3hdml | blue3 |
| Hnf4aos | darkolivegreen |
| Hnf4a | darkgrey |
| Ttpal | blue4 |
| Serinc3 | mistyrose |
| Pkig | deeppink |
| Ada | blue4 |
| Ywhab | blue4 |
| Tomm34 | mistyrose |
| Stk4 | blue4 |
| Wfdc15b | blue4 |
| Matn4 | green4 |
| Sdc4 | coral1 |
| Sys1 | firebrick2 |
| Gm14302 | blue3 |
| Dbndd2 | blue4 |
| Pigt | deeppink |
| Wfdc2 | blue4 |
| Wfdc16 | blue4 |
| Dnttip1 | honeydew |
| Gm22596 | blue3 |
| Ube2c | firebrick2 |
| Snx21 | green4 |
| Acot8 | indianred2 |
| Zswim3 | blue3 |
| Zswim1 | mistyrose |
| Ctsa | blue4 |
| Pltp | coral1 |
| Pcif1 | blue4 |
| Zfp335 | mistyrose |
| Zfp335os | antiquewhite2 |
| Mmp9 | blue4 |
| Ncoa5 | green4 |
| Cd40 | mistyrose |
| Slc35c2 | deeppink |
| Elmo2 | blue4 |
| Slc13a3 | green4 |
| Gm14437 | blue4 |
| 2810408M09Rik | blue4 |
| Eya2 | blue4 |
| Zmynd8 | blue3 |
| Gm11465 | blue4 |
| Ncoa3 | lightsteelblue |
| Sulf2 | blue4 |
| Gm11466 | blue4 |
| Prex1 | deeppink |
| Arfgef2 | blue4 |
| Cse1l | blue4 |
| Gm17096 | darkgrey |
| Stau1 | blue3 |
| Ddx27 | blue4 |
| Znfx1 | green4 |
| 1500012F01Rik | blue4 |
| Gm11471 | blue4 |
| Gm14290 | blue4 |
| Kcnb1 | blue4 |
| Ptgis | deeppink |
| B4galt5 | coral1 |
| Slc9a8 | blue4 |
| Spata2 | darkgrey |
| Rnf114 | deeppink |
| Snai1 | blue4 |
| Tmem189 | blue4 |
| Gm22355 | darkolivegreen |
| Gm14320 | darkolivegreen |
| Cebpb | darkgrey |
| Gm14321 | blue3 |
| Gm14319 | green4 |
| Ptpn1 | blue3 |
| Fam65c | darkolivegreen |
| Pard6b | darkgrey |
| Gm14235 | blue4 |
| Adnp | blue4 |
| Mocs3 | mistyrose |
| Kcng1 | blue4 |
| Nfatc2 | mistyrose |
| Atp9a | deeppink |
| Zfp64 | darkgrey |
| Zfp217 | darkgrey |
| Gm14270 | blue4 |
| Gm14269 | blue4 |
| Cyp24a1 | blue4 |
| Pfdn4 | blue4 |
| Fam210b | brown1 |
| Aurka | blue4 |
| Cstf1 | blue3 |
| Rtfdc1 | blue4 |
| Gm14303 | blue4 |
| Bmp7 | green4 |
| Rae1 | blue4 |
| Rbm38 | darkgrey |
| Pck1 | green4 |
| Pmepa1 | blue4 |
| Ppp4r1l-ps | blue3 |
| Rab22a | blue4 |
| Vapb | blue3 |
| Vamp7-ps | blue4 |
| Stx16 | blue3 |
| Npepl1 | green4 |
| Gnas | coral1 |
| Nelfcd | blue4 |
| Ctsz | blue4 |
| Atp5e | blue4 |
| Slmo2 | blue4 |
| Edn3 | darkolivegreen |
| Gm14393 | coral1 |
| Gm14439 | blue4 |
| Gm14399 | blue3 |
| Gm14305 | blue3 |
| Gm14295 | coral1 |
| Gm14410 | blue4 |
| Gm14412 | blue3 |
| Gm14418 | blue4 |
| Gm14420 | coral1 |
| Gm14403 | blue4 |
| Gm14322 | blue4 |
| Gm14325 | coral1 |
| Gm14326 | coral1 |
| Etohi1 | blue3 |
| Zfp931 | blue3 |
| Gm14292 | blue4 |
| Taf4a | blue3 |
| 4921531C22Rik | blue3 |
| Lsm14b | blue4 |
| Psma7 | blue4 |
| Ss18l1 | blue3 |
| Mtg2 | green4 |
| Osbpl2 | blue4 |
| Adrm1 | darkgrey |
| Lama5 | blue4 |
| Rps21 | firebrick2 |
| Cables2 | blue3 |
| Gata5 | blue3 |
| Slco4a1 | blue4 |
| Mrgbp | blue3 |
| Ogfr | saddlebrown |
| Dido1 | blue3 |
| Gid8 | blue3 |
| Ythdf1 | darkgrey |
| Arfgap1 | mistyrose |
| 9230112E08Rik | blue3 |
| Chrna4 | green4 |
| Ppdpf | blue4 |
| Helz2 | blue4 |
| Gmeb2 | darkgrey |
| Rtel1 | blue4 |
| Arfrp1 | blue3 |
| Zgpat | green4 |
| Slc2a4rg-ps | blue3 |
| Zbtb46 | blue4 |
| Tpd52l2 | blue4 |
| Dnajc5 | darkgrey |
| Uckl1 | blue3 |
| Znf512b | blue3 |
| Prpf6 | blue3 |
| Samd10 | blue4 |
| Sox18 | blue4 |
| Tcea2 | blue3 |
| Rgs19 | blue4 |
| Pcmtd2 | mistyrose |
| Polr3k | blue4 |
| Hnf4g | coral1 |
| Pex2 | mistyrose |
| UBC | blue3 |
| Pkia | blue4 |
| Zc2hc1a | blue4 |
| Gm16685 | blue4 |
| Hey1 | blue4 |
| Mrps28 | lightsteelblue |
| Tpd52 | blue4 |
| Zbtb10 | darkgrey |
| Zfp704 | blue3 |
| Pag1 | darkgrey |
| Fabp5 | mistyrose |
| Gm9833 | blue4 |
| Fabp4 | lightcoral |
| RP23-436F15.7 | blue3 |
| Impa1 | firebrick2 |
| Slc10a5 | darkolivegreen |
| Zfand1 | darkolivegreen |
| Chmp4c | blue4 |
| Snx16 | blue4 |
| RP23-187F18.1 | blue4 |
| RP24-81A22.2 | blue4 |
| Slc7a12 | darkolivegreen |
| Lrrcc1 | blue3 |
| E2f5 | darkgrey |
| 1810022K09Rik | blue4 |
| Car13 | blue4 |
| Car3 | lightcoral |
| Car2 | blue4 |
| Gm24694 | blue4 |
| Ythdf3 | deeppink |
| Bhlhe22 | blue4 |
| Gm23726 | darkolivegreen |
| Cyp7b1 | blue4 |
| Armc1 | mistyrose |
| Mtfr1 | blue4 |
| Pde7a | firebrick2 |
| 4632415L05Rik | coral1 |
| Cp | blue4 |
| Hps3 | blue4 |
| Hltf | firebrick2 |
| Gyg | blue4 |
| Gm7488 | blue4 |
| Tbl1xr1 | coral1 |
| Gm7536 | blue4 |
| Ect2 | blue4 |
| Nceh1 | firebrick2 |
| Tnfsf10 | saddlebrown |
| Fndc3b | blue4 |
| Pld1 | blue3 |
| Slc2a2 | coral1 |
| 1700112D23Rik | coral1 |
| RP24-468O23.2 | coral1 |
| Eif5a2 | green4 |
| RP24-388D8.4 | lightcoral |
| Rpl22l1 | blue3 |
| RP24-388D8.6 | blue3 |
| Mecom | firebrick2 |
| Mynn | deeppink |
| Lrrc31 | darkolivegreen |
| Sec62 | deeppink |
| RP24-393I15.4 | mistyrose |
| Gpr160 | blue4 |
| Phc3 | blue4 |
| Prkci | blue4 |
| Skil | darkgrey |
| Zmat3 | blue4 |
| Pik3ca | mistyrose |
| RP23-372I17.5 | darkgrey |
| Zfp639 | coral1 |
| RP23-372I17.3 | blue3 |
| Mfn1 | firebrick2 |
| Gnb4 | blue4 |
| Actl6a | blue4 |
| RP23-370O17.1 | blue4 |
| Mrpl47 | coral1 |
| Ndufb5 | blue4 |
| Ttc14 | blue3 |
| Ccdc39 | blue3 |
| Fxr1 | coral1 |
| Dnajc19 | blue4 |
| Mir1897 | blue3 |
| Atp11b | blue4 |
| Dcun1d1 | coral1 |
| Gm15952 | blue4 |
| Mccc1 | mistyrose |
| Acad9 | mistyrose |
| D3Ertd254e | blue4 |
| Anxa5 | blue4 |
| 1810062G17Rik | blue3 |
| Exosc9 | coral1 |
| Bbs7 | blue4 |
| 4932438A13Rik | blue4 |
| Cetn4 | blue4 |
| Bbs12 | blue3 |
| Nudt6 | honeydew |
| Spata5 | darkolivegreen |
| Spry1 | mistyrose |
| Gm5148 | blue4 |
| Ankrd50 | blue4 |
| Intu | firebrick2 |
| Hspa4l | blue4 |
| Plk4 | blue4 |
| Mfsd8 | blue3 |
| 3110057O12Rik | firebrick2 |
| Larp1b | deeppink |
| Pgrmc2 | blue3 |
| Jade1 | firebrick2 |
| Sclt1 | blue4 |
| D3Ertd751e | darkolivegreen |
| Pcdh18 | blue4 |
| Ccrn4l | blue3 |
| RP24-88F2.3 | darkgrey |
| Elf2 | darkgrey |
| RP23-403H2.3 | blue3 |
| Ndufc1 | blue4 |
| Naa15 | blue4 |
| Rab33b | blue4 |
| Setd7 | blue4 |
| 5031434O11Rik | blue3 |
| Maml3 | blue4 |
| Foxo1 | coral1 |
| Cog6 | coral1 |
| Lhfp | blue4 |
| Nhlrc3 | coral1 |
| Proser1 | mistyrose |
| Frem2 | blue3 |
| Ufm1 | coral1 |
| Postn | lightcoral |
| Supt20 | blue3 |
| Exosc8 | blue4 |
| Alg5 | blue4 |
| Rfxap | blue4 |
| Gm10254 | green4 |
| Spg20 | darkgrey |
| Nbea | blue4 |
| Tm4sf1 | darkgrey |
| Tm4sf4 | blue4 |
| Wwtr1 | blue4 |
| Commd2 | blue4 |
| Rnf13 | mistyrose |
| Pfn2 | blue4 |
| Tsc22d2 | darkgrey |
| Serp1 | blue4 |
| Eif2a | coral1 |
| Selt | coral1 |
| Siah2 | darkgrey |
| P2ry14 | blue4 |
| Aadac | mistyrose |
| Sucnr1 | darkolivegreen |
| Mbnl1 | indianred2 |
| Gm8325 | blue4 |
| P2ry1 | blue3 |
| Arhgef26 | blue3 |
| Dhx36 | coral1 |
| Mme | mistyrose |
| Plch1 | blue4 |
| E130311K13Rik | firebrick2 |
| Slc33a1 | blue4 |
| Gmps | coral1 |
| Ssr3 | coral1 |
| 4931440P22Rik | blue3 |
| Tiparp | darkgrey |
| Mir8120 | darkgrey |
| Lekr1 | darkolivegreen |
| Ccnl1 | darkgrey |
| RP24-372I24.4 | blue3 |
| Veph1 | blue3 |
| Rsrc1 | blue4 |
| Mlf1 | darkgrey |
| Gfm1 | mistyrose |
| Lxn | blue4 |
| RP23-267M9.1 | darkolivegreen |
| Mfsd1 | blue4 |
| 1110032F04Rik | blue4 |
| Ift80 | firebrick2 |
| Gm18588 | mistyrose |
| Smc4 | blue4 |
| Trim59 | blue4 |
| Kpna4 | blue4 |
| Gm22009 | blue4 |
| Ppm1l | blue4 |
| B3galnt1 | blue4 |
| Nmd3 | darkgrey |
| Sptssb | blue4 |
| Pdcd10 | green4 |
| Golim4 | blue4 |
| Gm9762 | blue4 |
| Rapgef2 | darkgrey |
| RP24-253I2.1 | blue4 |
| Fnip2 | deeppink |
| Ppid | blue4 |
| Etfdh | mistyrose |
| Tmem144 | blue4 |
| Pdgfc | blue3 |
| Ctso | saddlebrown |
| Gucy1b3 | blue4 |
| Gucy1a3 | antiquewhite2 |
| Map9 | coral1 |
| RP24-180K20.1 | firebrick2 |
| Fgg | blue4 |
| Fga | blue4 |
| Fgb | blue4 |
| Plrg1 | coral1 |
| Sfrp2 | green4 |
| Tlr2 | blue4 |
| D930015E06Rik | blue3 |
| Mnd1 | blue4 |
| Trim2 | blue4 |
| Fhdc1 | darkgrey |
| Arfip1 | blue4 |
| Fbxw7 | blue4 |
| Gatb | blue4 |
| Fam160a1 | blue4 |
| Glt28d2 | blue4 |
| Gm9790 | antiquewhite2 |
| Sh3d19 | blue4 |
| Gm25188 | blue4 |
| Rps3a1 | blue4 |
| Snord73a | blue4 |
| Rnu73b | blue4 |
| Lrba | firebrick2 |
| Gm3788 | blue4 |
| Cd1d1 | lightcoral |
| Kirrel | blue4 |
| Gm10705 | blue4 |
| Etv3 | darkgrey |
| Arhgef11 | blue3 |
| Pear1 | blue3 |
| Ntrk1 | blue4 |
| Insrr | darkolivegreen |
| Prcc | green4 |
| Hdgf | blue4 |
| Mrpl24 | blue3 |
| Rrnad1 | blue3 |
| Isg20l2 | blue4 |
| Nes | blue4 |
| Gpatch4 | blue4 |
| Apoa1bp | blue4 |
| Mef2d | darkgrey |
| 1700113A16Rik | darkolivegreen |
| Rhbg | coral1 |
| Tsacc | blue3 |
| RP23-168E14.10 | blue3 |
| Cct3 | blue4 |
| Glmp | indianred2 |
| Tmem79 | blue3 |
| Smg5 | blue3 |
| Pmf1 | blue3 |
| Slc25a44 | blue4 |
| Sema4a | mistyrose |
| Lmna | darkgrey |
| Rab25 | blue3 |
| Lamtor2 | blue4 |
| Ubqln4 | blue3 |
| Ssr2 | firebrick2 |
| Arhgef2 | blue3 |
| 2810403A07Rik | blue4 |
| Gm25820 | darkgrey |
| Gm25945 | darkgrey |
| Rit1 | orangered |
| Syt11 | blue3 |
| 5830417I10Rik | blue3 |
| Gon4l | blue4 |
| Msto1 | blue4 |
| n-R5s197 | blue3 |
| Dap3 | blue4 |
| Ash1l | blue4 |
| Rusc1 | blue4 |
| Fdps | deeppink |
| Pklr | blue4 |
| Clk2 | blue3 |
| Scamp3 | blue3 |
| Fam189b | blue3 |
| Gba | blue4 |
| Mtx1 | blue3 |
| Thbs3 | blue3 |
| Muc1 | blue4 |
| Trim46 | blue3 |
| Krtcap2 | blue4 |
| Dpm3 | deeppink |
| Slc50a1 | blue4 |
| Efna1 | darkgrey |
| Efna4 | blue4 |
| Adam15 | deeppink |
| Dcst1 | blue4 |
| Zbtb7b | deeppink |
| Gm15417 | blue4 |
| Flad1 | blue3 |
| Cks1b | blue4 |
| Shc1 | blue4 |
| Pygo2 | blue4 |
| Pbxip1 | blue3 |
| Pmvk | mistyrose |
| Adar | blue4 |
| Chrnb2 | blue3 |
| 4632404H12Rik | blue3 |
| Ube2q1 | darkgrey |
| She | blue4 |
| Il6ra | darkgrey |
| Atp8b2 | blue4 |
| Hax1 | darkolivegreen |
| Ubap2l | blue3 |
| Gm24608 | lightcoral |
| 4933434E20Rik | blue3 |
| Tpm3 | blue4 |
| Rps27 | blue4 |
| Rab13 | blue4 |
| Jtb | coral1 |
| Creb3l4 | blue3 |
| Slc39a1 | blue4 |
| Crtc2 | mistyrose |
| Dennd4b | blue3 |
| Gatad2b | darkgrey |
| Slc27a3 | blue3 |
| Ints3 | blue3 |
| Npr1 | green4 |
| Ilf2 | blue4 |
| Snapin | coral1 |
| Chtop | blue4 |
| S100a1 | lightsteelblue |
| S100a13 | blue4 |
| S100a14 | blue4 |
| S100a16 | blue4 |
| S100a4 | blue4 |
| S100a6 | blue4 |
| S100a8 | darkgrey |
| S100a9 | darkgrey |
| Lor | darkgrey |
| Sprr2g | blue4 |
| Gm9774 | blue4 |
| Sprr1a | blue4 |
| Smcp | coral1 |
| Gm4202 | blue4 |
| Tchh | green4 |
| Tchhl1 | blue4 |
| S100a11 | blue4 |
| S100a10 | blue4 |
| Them4 | mistyrose |
| C2cd4d | blue4 |
| Rorc | mistyrose |
| Tdrkh | blue4 |
| Mrpl9 | coral1 |
| Snx27 | blue3 |
| Tuft1 | darkgrey |
| Gm10972 | darkgrey |
| BC021767 | blue3 |
| Selenbp2 | blue4 |
| Cgn | blue4 |
| Pogz | blue3 |
| Psmb4 | blue4 |
| Gm26279 | blue3 |
| Selenbp1 | blue4 |
| Rfx5 | blue4 |
| Pi4kb | blue3 |
| A730011C13Rik | blue3 |
| Gm15265 | blue4 |
| Mir7013 | blue4 |
| Zfp687 | blue3 |
| 4930481B07Rik | blue3 |
| Psmd4 | honeydew |
| Pip5k1a | blue4 |
| Vps72 | blue3 |
| Scnm1 | blue3 |
| Tnfaip8l2 | saddlebrown |
| Gabpb2 | blue4 |
| Mllt11 | blue3 |
| Cdc42se1 | mistyrose |
| Gm128 | green4 |
| Prune | blue4 |
| Fam63a | blue4 |
| Anxa9 | blue3 |
| Cers2 | blue4 |
| Setdb1 | blue4 |
| Arnt | blue4 |
| Ctsk | blue4 |
| Ctss | saddlebrown |
| Golph3l | darkgrey |
| Rps10-ps1 | blue4 |
| Ensa | blue4 |
| Mcl1 | darkgrey |
| Adamtsl4 | blue4 |
| Ecm1 | coral1 |
| Tars2 | blue3 |
| Rprd2 | blue4 |
| Prpf3 | blue4 |
| Mrps21 | blue4 |
| Ciart | blue4 |
| BC028528 | blue4 |
| Aph1a | deeppink |
| Car14 | blue4 |
| Anp32e | coral1 |
| Plekho1 | blue4 |
| Vps45 | blue4 |
| Otud7b | brown1 |
| Mtmr11 | blue4 |
| Gm17690 | darkgrey |
| Sf3b4 | blue4 |
| Gm22027 | blue4 |
| Sv2a | darkolivegreen |
| Bola1 | blue4 |
| Hist2h2be | blue3 |
| Hist2h3c2 | green4 |
| Fcgr1 | blue4 |
| Gm15441 | blue3 |
| Txnip | blue3 |
| Polr3gl | darkolivegreen |
| Lix1l | blue4 |
| Rbm8a | blue4 |
| Pex11b | darkgrey |
| Pias3 | blue3 |
| Polr3c | blue4 |
| Gm22581 | blue4 |
| Rnf115 | green4 |
| Pdzk1 | blue4 |
| 4930442L01Rik | antiquewhite2 |
| Gpr89 | blue4 |
| Gja5 | blue4 |
| Acp6 | darkolivegreen |
| Bcl9 | blue3 |
| Chd1l | blue4 |
| Fmo5 | blue4 |
| Prkab2 | blue4 |
| Pde4dip | firebrick2 |
| Sec22b | blue4 |
| Notch2 | blue3 |
| Hmgcs2 | mistyrose |
| Phgdh | blue4 |
| Zfp697 | blue3 |
| Gm4450 | darkolivegreen |
| Gm10681 | coral1 |
| Hsd3b2 | blue4 |
| Hsd3b3 | blue4 |
| Hao2 | blue4 |
| 5730437C11Rik | blue3 |
| Gm22341 | blue4 |
| Wars2 | blue3 |
| Mir6481 | blue4 |
| Wdr3 | blue4 |
| Gdap2 | green4 |
| Man1a2 | blue4 |
| Vtcn1 | blue4 |
| Ttf2 | blue4 |
| Ptgfrn | blue4 |
| Igsf3 | darkgrey |
| Atp1a1 | blue4 |
| Mab21l3 | blue4 |
| Vangl1 | blue4 |
| Ngf | blue4 |
| Tspan2 | blue4 |
| Sike1 | blue4 |
| Csde1 | coral1 |
| Nras | blue4 |
| Bcas2 | blue4 |
| Trim33 | coral1 |
| Olfml3 | blue4 |
| Hipk1 | blue3 |
| Dclre1b | green4 |
| Ap4b1 | mistyrose |
| Ptpn22 | blue3 |
| Phtf1 | blue3 |
| Magi3 | blue4 |
| Lrig2 | blue4 |
| Slc16a1 | green4 |
| Gm6485 | blue4 |
| Ppm1j | blue4 |
| Rhoc | darkgrey |
| Mov10 | blue3 |
| Capza1 | blue4 |
| St7l | mistyrose |
| Cttnbp2nl | darkgrey |
| Kcnd3 | blue4 |
| Ddx20 | blue4 |
| Rap1a | coral1 |
| Atp5f1 | mistyrose |
| Wdr77 | blue4 |
| Gm4540 | blue4 |
| Dennd2d | blue3 |
| Cept1 | mistyrose |
| Dram2 | mistyrose |
| Lrif1 | coral1 |
| Cd53 | honeydew |
| Cym | blue4 |
| A630076J17Rik | blue4 |
| Prok1 | blue4 |
| Lamtor5 | darkolivegreen |
| Slc16a4 | blue4 |
| Rbm15 | darkgrey |
| Slc6a17 | mistyrose |
| Strip1 | blue4 |
| Gm10961 | darkgrey |
| Ahcyl1 | firebrick2 |
| Csf1 | blue4 |
| Eps8l3 | honeydew |
| 4933431E20Rik | darkolivegreen |
| Gstm5 | blue4 |
| Gstm7 | blue4 |
| Gstm6 | blue4 |
| Gstm2 | green4 |
| Gstm1 | blue3 |
| Gstm4 | brown1 |
| Ampd2 | blue3 |
| Gnai3 | blue4 |
| Amigo1 | green4 |
| Cyb561d1 | blue3 |
| Atxn7l2 | blue3 |
| Sypl2 | green4 |
| Gm12501 | blue4 |
| Psma5 | blue4 |
| Sort1 | blue3 |
| Celsr2 | green4 |
| Sars | blue4 |
| Scarna2 | blue3 |
| Tmem167b | deeppink |
| Taf13 | coral1 |
| Wdr47 | blue3 |
| Clcc1 | coral1 |
| Gpsm2 | coral1 |
| Stxbp3a | coral1 |
| Prpf38b | blue4 |
| Henmt1 | coral1 |
| Fam102b | blue4 |
| Slc25a24 | blue4 |
| Vav3 | brown1 |
| Prmt6 | blue4 |
| Amy1 | blue3 |
| Rnpc3 | blue4 |
| Olfm3 | blue4 |
| S1pr1 | darkgrey |
| A930005H10Rik | blue3 |
| Dph5 | blue4 |
| Slc30a7 | blue4 |
| Extl2 | blue3 |
| Vcam1 | darkgrey |
| Cdc14a | blue4 |
| Rtca | green4 |
| Dbt | firebrick2 |
| Gm9761 | blue4 |
| Trmt13 | blue4 |
| Hiat1 | coral1 |
| Slc35a3 | mistyrose |
| Agl | blue3 |
| Frrs1 | orangered |
| Palmd | brown1 |
| Snx7 | blue4 |
| Dpyd | deeppink |
| Ptbp2 | blue4 |
| Rwdd3 | mistyrose |
| Tmem56 | mistyrose |
| Alg14 | blue4 |
| Cnn3 | blue4 |
| Slc44a3 | blue3 |
| A730020M07Rik | firebrick2 |
| F3 | darkgrey |
| Abcd3 | mistyrose |
| Arhgap29 | coral1 |
| Abca4 | blue4 |
| Gm22903 | darkolivegreen |
| Gclm | blue4 |
| Gm17494 | darkgrey |
| Dnttip2 | brown1 |
| Mir760 | blue4 |
| Bcar3 | blue4 |
| Fnbp1l | coral1 |
| Pde5a | blue4 |
| 1810037I17Rik | blue4 |
| Usp53 | darkgrey |
| Synpo2 | blue4 |
| Sec24d | darkgrey |
| Mettl14 | blue4 |
| Snora24 | blue4 |
| Gm4617 | blue4 |
| Ugt8a | mistyrose |
| Camk2d | blue4 |
| Larp7 | blue4 |
| Neurog2 | blue4 |
| Tifa | blue4 |
| Ap1ar | blue4 |
| 5730508B09Rik | blue4 |
| Pitx2 | darkgrey |
| Enpep | coral1 |
| Elovl6 | blue4 |
| Egf | blue4 |
| Gm24515 | darkolivegreen |
| Rrh | blue4 |
| Gar1 | blue4 |
| Cfi | blue4 |
| Pla2g12a | blue4 |
| Casp6 | coral1 |
| Ccdc109b | blue4 |
| Sec24b | blue4 |
| Etnppl | mistyrose |
| Ostc | blue4 |
| Rpl34 | blue4 |
| Hadh | blue4 |
| Cyp2u1 | green4 |
| Sgms2 | darkgrey |
| Papss1 | blue4 |
| Dkk2 | blue3 |
| Aimp1 | blue4 |
| Tbck | firebrick2 |
| Npnt | blue3 |
| Gstcd | blue4 |
| Ints12 | blue4 |
| Arhgef38 | firebrick2 |
| Ppa2 | blue4 |
| Tet2 | darkolivegreen |
| Bdh2 | blue4 |
| Cisd2 | firebrick2 |
| 4930539J05Rik | blue4 |
| Ube2d3 | green4 |
| Manba | blue4 |
| Oaz2-ps | blue4 |
| Nfkb1 | blue4 |
| Slc39a8 | blue4 |
| Ppp3ca | coral1 |
| Emcn | blue4 |
| Ddit4l | mistyrose |
| H2afz | blue4 |
| Dnajb14 | blue4 |
| Lamtor3 | blue4 |
| Dapp1 | blue4 |
| Mttp | blue4 |
| Trmt10a | deeppink1 |
| 0610031O16Rik | mistyrose |
| Adh7 | coral1 |
| Adh1 | mistyrose |
| Gm16559 | darkolivegreen |
| Adh5 | blue4 |
| Metap1 | blue4 |
| Eif4e | blue4 |
| Mir1956 | deeppink1 |
| Tspan5 | blue4 |
| Rap1gds1 | blue4 |
| Unc5c | lightsteelblue |
| Bmpr1b | blue4 |
| Pdlim5 | blue4 |
| Gbp5 | saddlebrown |
| Gbp7 | saddlebrown |
| Gbp3 | saddlebrown |
| Gbp2 | saddlebrown |
| Ccbl2 | darkolivegreen |
| Gtf2b | green4 |
| Pkn2 | coral1 |
| Gm25965 | darkgrey |
| Lmo4 | coral1 |
| Hs2st1 | blue4 |
| 15-Sep | blue3 |
| Sh3glb1 | blue4 |
| Clca1 | blue4 |
| Odf2l | blue3 |
| Znhit6 | blue4 |
| Cyr61 | coral1 |
| Gm17501 | darkgrey |
| Ddah1 | darkgrey |
| Bcl10 | darkgrey |
| 2410004B18Rik | blue3 |
| Syde2 | firebrick2 |
| Mcoln3 | darkolivegreen |
| Mcoln2 | darkolivegreen |
| Lpar3 | antiquewhite2 |
| Ssx2ip | firebrick2 |
| Ctbs | firebrick2 |
| Spata1 | firebrick2 |
| Gng5 | blue4 |
| Rpf1 | coral1 |
| Prkacb | blue4 |
| Gm10288 | blue4 |
| Lphn2 | blue4 |
| Rpsa-ps10 | blue4 |
| Eltd1 | blue4 |
| Ifi44 | blue4 |
| Ptgfr | brown1 |
| Gipc2 | blue4 |
| Dnajb4 | darkgrey |
| Fubp1 | blue3 |
| Nexn | blue4 |
| Fam73a | coral1 |
| Usp33 | lightsteelblue |
| Zzz3 | blue4 |
| Ak5 | blue4 |
| Pigk | coral1 |
| St6galnac3 | blue3 |
| Rabggtb | blue4 |
| Snord45b | blue4 |
| Gm24494 | darkgrey |
| Acadm | mistyrose |
| Tyw3 | darkolivegreen |
| Cryz | mistyrose |
| Fpgt | mistyrose |
| Lrriq3 | mistyrose |
| Zranb2 | blue3 |
| Mir186 | blue3 |
| Ptger3 | darkolivegreen |
| Cth | mistyrose |
| Ankrd13c | blue4 |
| Srsf11 | blue4 |
| Lrrc40 | coral1 |
| Wls | firebrick2 |
| AI838599 | darkolivegreen |
| Tmem68 | coral1 |
| Tgs1 | blue4 |
| Lyn | blue4 |
| Gm22781 | darkgrey |
| Rps20 | blue4 |
| Chchd7 | blue4 |
| Gm11808 | blue4 |
| Penk | darkgrey |
| Impad1 | coral1 |
| Ubxn2b | blue3 |
| Sdcbp | blue4 |
| RP23-89L15.6 | darkgrey |
| Nsmaf | coral1 |
| Car8 | blue4 |
| Rab2a | deeppink |
| Chd7 | darkgrey |
| Asph | firebrick2 |
| 4930412C18Rik | firebrick2 |
| Gm12918 | blue4 |
| 2610301B20Rik | coral1 |
| Plekhf2 | brown1 |
| Ndufaf6 | blue4 |
| Gm11827 | mistyrose |
| Trp53inp1 | blue3 |
| Ccne2 | blue4 |
| Ints8 | blue4 |
| Dpy19l4 | firebrick2 |
| Esrp1 | darkolivegreen |
| 1110037F02Rik | blue4 |
| Gem | coral1 |
| Pdp1 | blue4 |
| 1700123M08Rik | blue4 |
| Tmem67 | blue3 |
| Rbm12b2 | blue3 |
| Rbm12b1 | blue3 |
| Fam92a | coral1 |
| Gm11847 | blue4 |
| Gm11836 | blue4 |
| Slc26a7 | darkolivegreen |
| Otud6b | blue4 |
| Tmem55a | blue4 |
| Gm11837 | blue3 |
| Gm11844 | blue4 |
| Tmem64 | blue4 |
| Calb1 | blue4 |
| Decr1 | mistyrose |
| Mir6400 | blue4 |
| Nbn | blue4 |
| Osgin2 | blue4 |
| Gm11874 | deeppink |
| Ripk2 | blue4 |
| Cpne3 | blue4 |
| Rmdn1 | darkolivegreen |
| Gm12353 | deeppink1 |
| Wwp1 | firebrick2 |
| Gm12354 | blue4 |
| Slc7a13 | blue4 |
| Atp6v0d2 | firebrick2 |
| Ttpa | antiquewhite2 |
| Ggh | green4 |
| Ccnc | coral1 |
| Tstd3 | blue4 |
| Usp45 | blue4 |
| Pnisr | blue3 |
| Coq3 | darkgrey |
| Fbxl4 | mistyrose |
| Ndufaf4 | coral1 |
| Ufl1 | coral1 |
| Fut9 | darkolivegreen |
| Manea | coral1 |
| Gm11914 | coral1 |
| Map3k7 | blue4 |
| Gm26254 | blue3 |
| Casp8ap2 | blue4 |
| Mdn1 | blue4 |
| Lyrm2 | coral1 |
| Ankrd6 | blue4 |
| Gm11942 | blue4 |
| Rragd | firebrick2 |
| 4933421O10Rik | blue3 |
| Ube2j1 | darkolivegreen |
| Pm20d2 | blue4 |
| Pnrc1 | darkgrey |
| Rngtt | blue4 |
| Gm12751 | blue4 |
| Gm12350 | blue4 |
| Akirin2 | darkgrey |
| Orc3 | blue4 |
| Rars2 | coral1 |
| Slc35a1 | blue4 |
| Smim8 | blue4 |
| Zfp292 | blue4 |
| Mob3b | coral1 |
| 3110043O21Rik | blue4 |
| Aco1 | blue4 |
| Ddx58 | blue4 |
| Topors | darkgrey |
| Ndufb6 | blue4 |
| Aptx | blue3 |
| Gm6297 | blue3 |
| Dnaja1 | darkgrey |
| Smu1 | coral1 |
| Gm12396 | blue4 |
| B4galt1 | darkolivegreen |
| Spink4 | blue4 |
| Bag1 | coral1 |
| Chmp5 | darkgrey |
| Nfx1 | blue4 |
| Aqp7 | blue3 |
| Gm25637 | blue3 |
| Aqp3 | blue4 |
| Nol6 | mistyrose |
| Ube2r2 | mistyrose |
| Ubap2 | blue3 |
| Gm22888 | blue4 |
| Gm24837 | blue3 |
| Dcaf12 | blue4 |
| n-R5s183 | blue3 |
| Ubap1 | darkgrey |
| Nudt2 | blue4 |
| AI464131 | blue3 |
| Fam219a | blue3 |
| Gm12404 | blue3 |
| Enho | firebrick2 |
| Cntfr | blue4 |
| Il11ra1 | blue4 |
| Rpp25l | deeppink |
| Dctn3 | blue3 |
| Sigmar1 | blue4 |
| Galt | green4 |
| Ccl27a | blue3 |
| Gm12407 | blue4 |
| Ccl19-ps1 | saddlebrown |
| Ccl19 | darkgrey |
| Ccl21a | blue4 |
| Dnajb5 | darkgrey |
| Vcp | blue4 |
| Fancg | blue3 |
| Pigo | deeppink |
| Stoml2 | blue4 |
| Fam214b | darkgrey |
| Unc13b | blue4 |
| Rusc2 | darkgrey |
| Tesk1 | deeppink |
| Cd72 | honeydew |
| Ccdc107 | blue4 |
| Arhgef39 | darkolivegreen |
| Gm12454 | darkolivegreen |
| Car9 | blue4 |
| Tpm2 | blue4 |
| Tln1 | blue3 |
| Creb3 | blue3 |
| Gba2 | blue3 |
| Rgp1 | blue3 |
| Gm12473 | blue4 |
| Npr2 | darkolivegreen |
| Hint2 | blue4 |
| Tmem8b | green4 |
| Hrct1 | blue4 |
| Reck | blue4 |
| Glipr2 | blue4 |
| Clta | firebrick2 |
| Gne | blue4 |
| Rnf38 | blue4 |
| Zcchc7 | blue3 |
| Gm12678 | green4 |
| Grhpr | blue4 |
| Zbtb5 | blue3 |
| Polr1e | blue4 |
| Fbxo10 | blue4 |
| Tomm5 | blue4 |
| Trmt10b | coral1 |
| Exosc3 | blue4 |
| Dcaf10 | green4 |
| Slc25a51 | blue4 |
| Shb | darkgrey |
| Aldh1b1 | saddlebrown |
| Stra6l | blue4 |
| Tdrd7 | blue4 |
| Tstd2 | darkgrey |
| Ncbp1 | blue3 |
| Xpa | mistyrose |
| 5830415F09Rik | blue4 |
| Anp32b | blue4 |
| Nans | antiquewhite2 |
| Coro2a | green4 |
| Tbc1d2 | blue4 |
| Anks6 | blue3 |
| Galnt12 | blue4 |
| Gm12426 | blue4 |
| Col15a1 | blue4 |
| Tgfbr1 | blue4 |
| Alg2 | blue3 |
| Sec61b | blue4 |
| Stx17 | blue3 |
| Erp44 | darkgrey |
| Invs | darkolivegreen |
| Tex10 | blue4 |
| Msantd3 | blue4 |
| Acnat2 | antiquewhite2 |
| Acnat1 | firebrick2 |
| Mrpl50 | coral1 |
| Zfp189 | coral1 |
| Aldob | mistyrose |
| Tmem246 | green4 |
| Rnf20 | blue4 |
| Smc2 | blue4 |
| Vma21-ps | blue4 |
| Nipsnap3b | blue4 |
| Abca1 | blue4 |
| Slc44a1 | blue4 |
| Fktn | blue4 |
| Tmem38b | mistyrose |
| Zfp462 | blue4 |
| Rad23b | blue4 |
| Klf4 | darkgrey |
| Ikbkap | blue4 |
| Gm22952 | lightsteelblue |
| Fam206a | blue4 |
| Ctnnal1 | deeppink1 |
| Tmem245 | blue4 |
| Gm25053 | blue3 |
| Epb4.1l4b | green4 |
| Ptpn3 | blue3 |
| D630039A03Rik | blue3 |
| Txn1 | blue4 |
| Svep1 | blue4 |
| Lpar1 | blue4 |
| AI314180 | coral1 |
| Ptgr1 | mistyrose |
| Dnajc25 | green4 |
| Ugcg | darkgrey |
| Susd1 | blue4 |
| Ptbp3 | blue4 |
| Hsdl2 | orangered |
| E130308A19Rik | blue4 |
| Inip | blue4 |
| Snx30 | blue4 |
| Slc31a2 | blue4 |
| Fkbp15 | green4 |
| Slc31a1 | blue4 |
| Cdc26 | blue4 |
| Prpf4 | blue4 |
| Rnf183 | mistyrose |
| Wdr31 | darkolivegreen |
| Bspry | blue4 |
| Hdhd3 | green4 |
| Alad | green4 |
| Pole3 | blue4 |
| Rgs3 | deeppink |
| Zfp618 | blue3 |
| Ambp | darkgrey |
| Kif12 | blue3 |
| Col27a1 | blue3 |
| Whrn | mistyrose |
| Atp6v1g1 | orangered |
| 6330416G13Rik | blue4 |
| Tnfsf15 | blue4 |
| Tnc | blue4 |
| Pappa | blue4 |
| Astn2 | deeppink |
| Trim32 | green4 |
| Tlr4 | blue4 |
| Gm11249 | blue4 |
| Gm11223 | blue4 |
| Cdk5rap2 | blue4 |
| Megf9 | blue4 |
| Tle1 | darkgrey |
| Rasef | blue4 |
| Kdm4c | blue4 |
| Tmem261 | blue4 |
| Ptprd | firebrick2 |
| Lurap1l | blue4 |
| Mpdz | blue4 |
| Nfib | blue3 |
| Zdhhc21 | blue4 |
| Frem1 | blue3 |
| Ttc39b | blue4 |
| Snapc3 | blue3 |
| Psip1 | blue4 |
| Gm10154 | blue4 |
| Ccdc171 | blue3 |
| Bnc2 | orangered |
| Cntln | blue4 |
| Sh3gl2 | blue4 |
| Rraga | blue4 |
| Haus6 | blue4 |
| Plin2 | blue4 |
| Dennd4c | blue4 |
| Rps6 | blue4 |
| Acer2 | darkolivegreen |
| Mllt3 | blue4 |
| Focad | blue4 |
| Ptplad2 | blue4 |
| Mrpl48-ps | blue4 |
| Klhl9 | blue3 |
| Mir31 | blue4 |
| Mtap | blue4 |
| Gm26490 | blue4 |
| Cdkn2b | darkolivegreen |
| Gm12669 | blue4 |
| Tusc1 | blue4 |
| Caap1 | blue3 |
| Plaa | blue4 |
| Ift74 | coral1 |
| Lrrc19 | blue4 |
| Tek | blue4 |
| Gm12693 | blue4 |
| Mysm1 | blue4 |
| Jun | coral1 |
| Junos | lightcoral |
| Fggy | blue4 |
| Hook1 | blue4 |
| Cyp2j13 | blue4 |
| Hspe1-ps6 | lightsteelblue |
| Cyp2j7 | blue4 |
| Cyp2j11 | blue4 |
| Cyp2j6 | blue4 |
| Cyp2j9 | deeppink |
| Cyp2j5 | mistyrose |
| Gm12696 | blue4 |
| Nfia | blue3 |
| 0610025J13Rik | blue4 |
| Gm12788 | blue4 |
| Tm2d1 | mistyrose |
| Gm25997 | darkgrey |
| Inadl | blue3 |
| Kank4 | green4 |
| Usp1 | coral1 |
| Dock7 | blue4 |
| Angptl3 | coral1 |
| Atg4c | blue3 |
| Alg6 | blue4 |
| Itgb3bp | mistyrose |
| Efcab7 | coral1 |
| Pgm2 | blue4 |
| Ror1 | blue3 |
| Ube2u | deeppink |
| Cachd1 | blue3 |
| Jak1 | blue4 |
| Gm12796 | blue4 |
| E130102H24Rik | mistyrose |
| 0610043K17Rik | mistyrose |
| Gm12795 | darkolivegreen |
| Gm12798 | darkolivegreen |
| Ak4 | darkolivegreen |
| Dnajc6 | blue4 |
| Leprot | blue4 |
| Lepr | green4 |
| Pde4b | darkgrey |
| Wdr78 | blue3 |
| Mier1 | green4 |
| Slc35d1 | blue3 |
| Oma1 | blue3 |
| Gm12715 | blue4 |
| Dab1 | blue3 |
| C8a | blue4 |
| Gm17662 | darkolivegreen |
| 1700024P16Rik | green4 |
| Prkaa2 | firebrick2 |
| Ppap2b | blue4 |
| Usp24 | blue3 |
| Pcsk9 | blue4 |
| Bsnd | green4 |
| Tmem61 | darkolivegreen |
| Dhcr24 | deeppink |
| Ttc22 | blue4 |
| Pars2 | darkgrey |
| Ttc4 | blue3 |
| Mroh7 | blue4 |
| Fam151a | blue4 |
| Acot11 | blue4 |
| Ssbp3 | green4 |
| Mrpl37 | deeppink |
| Cyb5rl | green4 |
| Tceanc2 | mistyrose |
| Tmem59 | blue4 |
| Lrrc42 | blue4 |
| Hspb11 | blue3 |
| Dio1 | blue4 |
| Yipf1 | coral1 |
| Ndc1 | blue4 |
| Glis1 | antiquewhite2 |
| Lrp8 | blue4 |
| Magoh | green4 |
| 0610037L13Rik | blue4 |
| Cpt2 | mistyrose |
| Podn | blue4 |
| Scp2 | mistyrose |
| Mir6397 | darkgrey |
| Echdc2 | blue4 |
| Zyg11b | mistyrose |
| Coa7 | coral1 |
| Gpx7 | blue4 |
| Zcchc11 | blue4 |
| Prpf38a | darkolivegreen |
| Cc2d1b | blue3 |
| Gm17354 | blue3 |
| Zfyve9 | firebrick2 |
| Btf3l4 | coral1 |
| Txndc12 | blue4 |
| Kti12 | blue4 |
| Rab3b | coral1 |
| Nrd1 | blue4 |
| Osbpl9 | mistyrose |
| Eps15 | blue4 |
| Ttc39a | blue4 |
| Rnf11 | blue4 |
| 9630013D21Rik | blue4 |
| Cdkn2c | blue4 |
| Faf1 | blue4 |
| Bend5 | blue4 |
| n-R5s191 | blue3 |
| Spata6 | coral1 |
| Slc5a9 | darkolivegreen |
| Trabd2b | green4 |
| Foxd2 | blue4 |
| Cmpk1 | blue4 |
| Gm24045 | blue4 |
| Pdzk1ip1 | green4 |
| Cyp4a12a | blue4 |
| Cyp4a12b | blue4 |
| Cyp4a14 | blue3 |
| Cyp4a10 | firebrick2 |
| Cyp4a31 | mistyrose |
| Cyp4a32 | firebrick2 |
| Cyp4b1 | blue4 |
| Efcab14 | blue4 |
| Atpaf1 | coral1 |
| Gm12847 | blue3 |
| Mob3c | blue3 |
| Mknk1 | blue3 |
| Faah | coral1 |
| Nsun4 | blue4 |
| Uqcrh | blue4 |
| Gm12854 | blue4 |
| Lrrc41 | blue4 |
| Pomgnt1 | green4 |
| Lurap1 | blue3 |
| Tspan1 | blue4 |
| Pik3r3 | blue4 |
| Mast2 | blue4 |
| Ipp | mistyrose |
| Tmem69 | darkgrey |
| Gpbp1l1 | blue4 |
| Ccdc17 | darkolivegreen |
| Nasp | blue4 |
| Akr1a1 | blue4 |
| Prdx1 | blue4 |
| Mmachc | blue3 |
| Ccdc163 | blue3 |
| Tesk2 | blue4 |
| Toe1 | antiquewhite2 |
| Mutyh | blue4 |
| Hpdl | antiquewhite2 |
| Gm12996 | blue4 |
| Gm12993 | blue4 |
| Zswim5 | blue3 |
| Urod | blue4 |
| Hectd3 | green4 |
| Eif2b3 | blue4 |
| Ptch2 | blue4 |
| Btbd19 | darkgrey |
| Plk3 | darkgrey |
| Rps8 | blue4 |
| Gm22980 | darkgrey |
| Snord55 | darkgrey |
| Tmem53 | blue4 |
| Rnf220 | blue4 |
| Eri3 | deeppink |
| Dmap1 | blue4 |
| Slc6a9 | blue4 |
| Atp6v0b | deeppink |
| Dph2 | blue4 |
| Ipo13 | deeppink |
| Artn | blue3 |
| St3gal3 | coral1 |
| Kdm4a | blue3 |
| Ptprf | green4 |
| Hyi | blue4 |
| Szt2 | blue3 |
| Med8 | blue3 |
| Elovl1 | blue4 |
| Cdc20 | mistyrose |
| Tie1 | deeppink |
| 2610528J11Rik | blue4 |
| Tmem125 | blue4 |
| Ebna1bp2 | blue4 |
| Slc2a1 | mistyrose |
| Mir1957a | darkgrey |
| Gm12867 | darkgrey |
| Gm12868 | darkgrey |
| Zfp691 | blue3 |
| Ccdc23 | blue4 |
| AU022252 | darkgrey |
| Lepre1 | blue4 |
| Gm12927 | blue4 |
| Cldn19 | green4 |
| Ybx1 | blue4 |
| Ppih | blue4 |
| Ccdc30 | mistyrose |
| Ldha-ps2 | blue4 |
| Ppcs | orangered |
| Zmynd12 | blue4 |
| AA415398 | darkolivegreen |
| Foxj3 | deeppink1 |
| Guca2a | blue4 |
| Guca2b | coral1 |
| Scmh1 | mistyrose |
| Ctps | blue4 |
| Gm8439 | blue4 |
| Cited4 | darkgrey |
| Nfyc | darkolivegreen |
| Exo5 | coral1 |
| Zfp69 | firebrick2 |
| Smap2 | mistyrose |
| Zmpste24 | coral1 |
| Rlf | brown1 |
| Ppt1 | mistyrose |
| Cap1 | blue4 |
| Mfsd2a | coral1 |
| Mycl | blue3 |
| Trit1 | brown1 |
| Ppie | blue4 |
| Nt5c1a | blue4 |
| Heyl | blue4 |
| Pabpc4 | blue4 |
| Gm25788 | darkgrey |
| Gm22154 | darkgrey |
| Macf1 | blue4 |
| Ndufs5 | blue4 |
| Akirin1 | brown1 |
| Rhbdl2 | blue4 |
| Mycbp | coral1 |
| Rragc | blue4 |
| Gm12902 | darkgrey |
| Utp11l | blue4 |
| Fhl3 | blue4 |
| Sf3a3 | blue4 |
| Mir697 | blue3 |
| Inpp5b | mistyrose |
| Mtf1 | blue4 |
| 1110065P20Rik | blue4 |
| Yrdc | darkgrey |
| 9930104L06Rik | blue4 |
| Gnl2 | blue4 |
| Snip1 | darkgrey |
| Gm12932 | blue4 |
| Meaf6 | blue4 |
| Zc3h12a | darkgrey |
| Csf3r | blue4 |
| Mrps15 | blue4 |
| Rpl28-ps3 | blue4 |
| Lsm10 | blue3 |
| Stk40 | darkgrey |
| Gm12946 | blue4 |
| Eva1b | blue4 |
| Sh3d21 | antiquewhite2 |
| Thrap3 | blue4 |
| Map7d1 | blue4 |
| Trappc3 | deeppink1 |
| Adprhl2 | blue4 |
| Ago3 | blue4 |
| Ago1 | blue3 |
| Ago4 | blue3 |
| 5730409E04Rik | blue4 |
| Psmb2 | blue4 |
| Ncdn | blue4 |
| AU040320 | green4 |
| Gm25604 | darkgrey |
| Zmym4 | blue4 |
| Sfpq | blue4 |
| Gm12940 | blue3 |
| Gm12941 | blue3 |
| Zmym1 | coral1 |
| Zmym6 | blue3 |
| Gm12942 | green4 |
| Smim12 | deeppink |
| Gja4 | blue3 |
| Gjb3 | blue4 |
| Gjb4 | blue4 |
| Tlr12 | green4 |
| Phc2 | blue4 |
| Zfp362 | blue3 |
| Trim62 | blue3 |
| Azin2 | blue4 |
| Ak2 | blue3 |
| Rnf19b | darkgrey |
| Tmem54 | blue4 |
| S100pbp | coral1 |
| Yars | blue4 |
| C77080 | mistyrose |
| Rbbp4 | blue4 |
| Zbtb8os | blue4 |
| Bsdc1 | blue4 |
| Marcksl1 | blue4 |
| Hdac1 | blue4 |
| Fam167b | blue4 |
| Eif3i | blue4 |
| Tmem234 | deeppink |
| Dcdc2b | blue3 |
| Iqcc | blue3 |
| Ccdc28b | blue3 |
| Txlna | blue3 |
| Kpna6 | blue4 |
| Tmem39b | blue3 |
| Mir7016 | blue3 |
| Khdrbs1 | blue4 |
| Ptp4a2 | blue4 |
| Gm12966 | deeppink1 |
| Col16a1 | blue4 |
| Pef1 | blue4 |
| Tinagl1 | blue4 |
| Gm853 | blue4 |
| Serinc2 | blue4 |
| Fabp3 | blue4 |
| Zcchc17 | blue3 |
| Snrnp40 | blue4 |
| Nkain1 | blue4 |
| Pum1 | coral1 |
| Sdc3 | blue4 |
| Laptm5 | darkgrey |
| Gm12962 | blue4 |
| Ptpru | deeppink |
| Mecr | blue4 |
| Srsf4 | blue3 |
| RP23-13A13.6 | blue4 |
| Tmem200b | blue4 |
| Epb4.1 | blue3 |
| Ythdf2 | blue4 |
| Rps15a-ps4 | blue4 |
| Gmeb1 | blue4 |
| Taf12 | blue3 |
| Rab42 | darkolivegreen |
| Snhg12 | darkgrey |
| Snora16a | blue3 |
| Trnau1ap | blue4 |
| Rcc1 | blue4 |
| Phactr4 | mistyrose |
| Med18 | coral1 |
| Sesn2 | blue3 |
| Atpif1 | blue3 |
| Dnajc8 | blue4 |
| Gm24762 | blue3 |
| Eya3 | blue4 |
| Xkr8 | blue3 |
| Smpdl3b | blue4 |
| Rpa2 | blue4 |
| Themis2 | honeydew |
| Ppp1r8 | blue4 |
| Stx12 | coral1 |
| Gm13033 | blue4 |
| Fam76a | blue3 |
| Ahdc1 | blue3 |
| Wasf2 | blue4 |
| Cd164l2 | blue4 |
| Map3k6 | darkgrey |
| Sytl1 | green4 |
| Tmem222 | firebrick2 |
| Wdtc1 | coral1 |
| Slc9a1 | blue3 |
| Fam46b | coral1 |
| Kdf1 | green4 |
| Nudc | darkgrey |
| Nr0b2 | coral1 |
| Gm13258 | blue4 |
| Gpatch3 | coral1 |
| Gpn2 | blue3 |
| Sfn | blue4 |
| Zdhhc18 | blue4 |
| Pigv | green4 |
| Arid1a | blue3 |
| Rps6ka1 | blue4 |
| Hmgn2 | blue4 |
| Dhdds | coral1 |
| Aim1l | blue4 |
| Cd52 | saddlebrown |
| Sh3bgrl3 | blue4 |
| Cep85 | blue4 |
| Cnksr1 | darkgrey |
| Grrp1 | blue4 |
| Pdik1l | coral1 |
| Trim63 | blue4 |
| Gm13195 | blue3 |
| Slc30a2 | blue3 |
| Extl1 | blue3 |
| Pafah2 | brown1 |
| Stmn1 | blue4 |
| Paqr7 | blue4 |
| Mtfr1l | blue3 |
| Sepn1 | blue4 |
| Man1c1 | blue4 |
| Ldlrap1 | blue4 |
| Tmem57 | coral1 |
| Tmem50a | blue4 |
| Rsrp1 | blue3 |
| Syf2 | blue3 |
| Clic4 | blue4 |
| Srrm1 | mistyrose |
| Rcan3 | blue4 |
| Nipal3 | green4 |
| Ifnlr1 | blue4 |
| Gm12989 | blue4 |
| Il22ra1 | blue3 |
| Myom3 | lightsteelblue |
| Srsf10 | blue4 |
| Pnrc2 | coral1 |
| Fuca1 | blue4 |
| Hmgcl | blue4 |
| Gale | blue4 |
| Lypla2 | blue4 |
| Pithd1 | blue4 |
| Tceb3 | blue4 |
| Rpl11 | blue4 |
| Id3 | blue4 |
| E2f2 | blue4 |
| Asap3 | green4 |
| Tcea3 | darkolivegreen |
| Zfp46 | blue4 |
| Hnrnpr | blue4 |
| Gm17388 | blue3 |
| Luzp1 | blue4 |
| Kdm1a | blue4 |
| Ephb2 | blue4 |
| C1qb | honeydew |
| C1qc | honeydew |
| C1qa | honeydew |
| Zbtb40 | green4 |
| Wnt4 | lightcoral |
| Cdc42 | blue4 |
| Gm25772 | darkgrey |
| 2810405F17Rik | blue3 |
| Hspg2 | mistyrose |
| Usp48 | blue4 |
| Rap1gap | darkolivegreen |
| Alpl | blue3 |
| Ece1 | mistyrose |
| Eif4g3 | lightsteelblue |
| 2310026L22Rik | blue4 |
| Hp1bp3 | blue3 |
| Ddost | blue4 |
| Pink1 | blue4 |
| Cda | blue4 |
| Rpl38-ps1 | blue4 |
| Mul1 | coral1 |
| Camk2n1 | green4 |
| Ubxn10 | blue3 |
| Pla2g5 | deeppink |
| Otud3 | blue4 |
| Rnf186 | coral1 |
| Tmco4 | blue4 |
| Nbl1 | blue4 |
| Minos1 | blue3 |
| Capzb | blue4 |
| Pqlc2 | green4 |
| Akr7a5 | blue4 |
| Gm24245 | blue4 |
| Mrto4 | blue4 |
| Emc1 | blue3 |
| Ubr4 | blue3 |
| Iffo2 | darkgrey |
| Aldh4a1 | blue4 |
| Mir7020 | blue4 |
| Klhdc7a | blue3 |
| Arhgef10l | coral1 |
| Rcc2 | blue4 |
| Sdhb | blue4 |
| Atp13a2 | green4 |
| Mfap2 | blue4 |
| Crocc | green4 |
| Necap2 | deeppink |
| Szrd1 | deeppink |
| Fbxo42 | mistyrose |
| Rsg1 | blue4 |
| Arhgef19 | green4 |
| Gm13056 | blue4 |
| Epha2 | darkgrey |
| Fam131c | blue3 |
| Clcnka | green4 |
| Gm13075 | blue3 |
| Clcnkb | green4 |
| Hspb7 | blue4 |
| Zbtb17 | mistyrose |
| Spen | mistyrose |
| B330016D10Rik | blue3 |
| Fblim1 | blue4 |
| Tmem82 | darkgrey |
| Slc25a34 | mistyrose |
| Plekhm2 | blue4 |
| Ddi2 | coral1 |
| Agmat | blue4 |
| Dnajc16 | blue3 |
| Casp9 | darkgrey |
| Efhd2 | darkgrey |
| Tmem51 | mistyrose |
| Gm13053 | blue4 |
| Kazn | blue3 |
| Prdm2 | orangered |
| Pdpn | blue4 |
| Gm13127 | blue4 |
| Dhrs3 | blue4 |
| Vps13d | blue3 |
| Tnfrsf1b | blue4 |
| Znf41-ps | blue4 |
| Gm13241 | blue4 |
| Gm13245 | blue3 |
| Gm13139 | coral1 |
| Gm13157 | blue4 |
| Rps19-ps3 | blue3 |
| Zfp933 | coral1 |
| Miip | blue3 |
| Fv1 | blue3 |
| Mfn2 | green4 |
| Plod1 | deeppink |
| 2510039O18Rik | deeppink |
| Clcn6 | coral1 |
| Mthfr | green4 |
| Agtrap | blue4 |
| Mad2l2 | blue4 |
| Fbxo6 | blue4 |
| Fbxo44 | blue4 |
| Fbxo2 | blue4 |
| Ubiad1 | blue4 |
| Gm23303 | blue4 |
| Mtor | blue4 |
| Angptl7 | green4 |
| Gm23318 | coral1 |
| Exosc10 | blue4 |
| Srm | blue4 |
| Tardbp | blue3 |
| Casz1 | mistyrose |
| Gm13205 | blue3 |
| Pex14 | coral1 |
| Dffa | blue4 |
| Apitd1 | blue4 |
| Pgd | blue4 |
| Kif1b | blue4 |
| Ube4b | blue3 |
| Ube4bos2 | blue3 |
| Trmt112-ps2 | blue4 |
| Nmnat1 | blue3 |
| Lzic | blue4 |
| Ctnnbip1 | coral1 |
| Clstn1 | green4 |
| Pik3cd | honeydew |
| Tmem201 | green4 |
| Slc25a33 | blue3 |
| Spsb1 | darkgrey |
| H6pd | blue4 |
| RP23-169H13.6 | blue3 |
| Slc2a5 | mistyrose |
| Eno1 | brown1 |
| Rere | mistyrose |
| Errfi1 | blue3 |
| Park7 | blue4 |
| Per3 | blue3 |
| Vamp3 | blue4 |
| Dnajc11 | blue4 |
| Thap3 | blue3 |
| Phf13 | darkgrey |
| Klhl21 | blue3 |
| Zbtb48 | blue3 |
| Nol9 | blue4 |
| Plekhg5 | mistyrose |
| Tnfrsf25 | blue3 |
| Espn | green4 |
| Acot7 | blue4 |
| Gpr153 | blue4 |
| Icmt | blue4 |
| Rpl22 | blue4 |
| Kcnab2 | coral1 |
| Nphp4 | blue3 |
| A430005L14Rik | darkgrey |
| Dffb | blue4 |
| Cep104 | blue3 |
| Lrrc47 | mistyrose |
| Smim1 | deeppink1 |
| Ccdc27 | blue3 |
| Wrap73 | blue3 |
| Tprgl | deeppink1 |
| Arhgef16 | mistyrose |
| Prdm16 | green4 |
| Gm13111 | darkolivegreen |
| Gm27202 | green4 |
| Fam213b | darkolivegreen |
| Tnfrsf14 | blue4 |
| Pank4 | blue3 |
| Pex10 | green4 |
| Rer1 | blue4 |
| Ski | mistyrose |
| 2610002J02Rik | blue3 |
| Prkcz | blue4 |
| Tmem52 | darkolivegreen |
| Gnb1 | blue4 |
| Nadk | blue4 |
| Slc35e2 | blue3 |
| Gm16024 | blue4 |
| Cdk11b | blue4 |
| Mmp23 | blue3 |
| Mib2 | green4 |
| B930041F14Rik | blue3 |
| Ssu72 | blue4 |
| Gm22573 | blue3 |
| Atad3a | blue3 |
| Vwa1 | blue3 |
| Gm26840 | blue3 |
| Tmem88b | darkolivegreen |
| Ankrd65 | blue4 |
| Mrpl20 | blue4 |
| Ccnl2 | blue3 |
| Aurkaip1 | blue4 |
| Mxra8 | blue3 |
| Dvl1 | blue3 |
| Cptp | blue3 |
| Cpsf3l | blue3 |
| Pusl1 | blue4 |
| Acap3 | blue4 |
| Ube2j2 | darkgrey |
| Fam132a | blue4 |
| B3galt6 | blue4 |
| Sdf4 | blue4 |
| Gm16008 | blue3 |
| Gm25982 | blue4 |
| Tnfrsf18 | blue4 |
| Ttll10 | blue3 |
| Gm13648 | blue3 |
| 9430015G10Rik | blue3 |
| Agrn | blue4 |
| Isg15 | saddlebrown |
| AW011738 | blue3 |
| Perm1 | darkolivegreen |
| Plekhn1 | blue4 |
| Klhl17 | green4 |
| Noc2l | blue4 |
| Gm15772 | blue4 |
| Cdk6 | blue4 |
| Fam133b | brown1 |
| 1700109H08Rik | darkgrey |
| Rbm48 | green4 |
| Pex1 | firebrick2 |
| Gatad1 | blue4 |
| Ankib1 | firebrick2 |
| Krit1 | blue4 |
| Mterf1a | coral1 |
| Akap9 | blue4 |
| Cyp51 | blue4 |
| Mterf1b | blue3 |
| Fzd1 | darkgrey |
| Cdk14 | blue4 |
| Cldn12 | blue4 |
| Gtpbp10 | coral1 |
| Cfap69 | blue4 |
| Steap2 | blue4 |
| Steap1 | blue4 |
| Tubb4b-ps1 | blue4 |
| Steap4 | darkgrey |
| Sri | blue4 |
| Dbf4 | blue4 |
| Slc25a40 | blue3 |
| Abcb1a | blue4 |
| Abcb1b | blue4 |
| Crot | mistyrose |
| Gm15610 | blue4 |
| Gm15611 | blue4 |
| Tmem243 | mistyrose |
| Dmtf1 | blue3 |
| Gm7332 | blue4 |
| Sema3d | blue4 |
| Gm10108 | antiquewhite2 |
| Pclo | green4 |
| Cacna2d1 | blue4 |
| Hgf | blue4 |
| Sema3c | blue4 |
| Cd36 | coral1 |
| Gnai1 | mistyrose |
| Magi2 | coral1 |
| Phtf2 | coral1 |
| Tmem60 | blue4 |
| Rsbn1l | firebrick2 |
| Ptpn12 | blue4 |
| Gsap | darkolivegreen |
| Fgl2 | darkgrey |
| Fam185a | blue3 |
| Armc10 | blue4 |
| Napepld | blue4 |
| Pmpcb | blue4 |
| Dnajc2 | blue4 |
| Psmc2 | blue4 |
| Orc5 | coral1 |
| Rpl17-ps5 | blue4 |
| 5031425E22Rik | blue3 |
| Kmt2e | deeppink1 |
| Gm25219 | blue3 |
| Srpk2 | blue3 |
| Pus7 | blue4 |
| Rint1 | blue3 |
| Tomm7 | blue4 |
| 2700038G22Rik | blue4 |
| Fam126a | blue4 |
| Klhl7 | deeppink1 |
| Nupl2 | blue4 |
| Kcnh2 | blue4 |
| Nos3 | blue4 |
| Abcb8 | blue4 |
| Cdk5 | green4 |
| Slc4a2 | green4 |
| Fastk | blue3 |
| Tmub1 | green4 |
| Agap3 | brown1 |
| Abcf2 | deeppink |
| Gm10221 | antiquewhite2 |
| Chpf2 | blue4 |
| Smarcd3 | blue3 |
| Nub1 | blue3 |
| Wdr86 | green4 |
| Rheb | coral1 |
| Prkag2 | blue3 |
| Prkag2os2 | blue4 |
| 2900005J15Rik | blue3 |
| E130116L18Rik | blue4 |
| Galnt11 | blue4 |
| Kmt2c | lightsteelblue |
| 4831440E17Rik | blue3 |
| 1700096K18Rik | darkolivegreen |
| Xrcc2 | blue4 |
| Paxip1 | blue4 |
| Insig1 | blue4 |
| Rbm33 | orangered |
| Rnf32 | blue3 |
| Lmbr1 | blue4 |
| Nom1 | coral1 |
| Ube3c | blue4 |
| Dnajb6 | blue3 |
| Il6 | darkgrey |
| Tyms | blue4 |
| Hadha | firebrick2 |
| Hadhb | mistyrose |
| Ept1 | blue4 |
| Slc35f6 | blue4 |
| Cenpa | coral1 |
| Mapre3 | blue4 |
| Tmem214 | deeppink |
| Agbl5 | green4 |
| Ost4 | deeppink |
| Emilin1 | blue4 |
| Khk | blue4 |
| Cgref1 | blue4 |
| Preb | mistyrose |
| Slc5a6 | blue4 |
| Atraid | mistyrose |
| Cad | blue4 |
| Mpv17 | darkgrey |
| Gtf3c2 | blue3 |
| Eif2b4 | deeppink |
| Snx17 | firebrick2 |
| Zfp513 | blue4 |
| Ppm1g | blue4 |
| Nrbp1 | blue4 |
| Krtcap3 | blue4 |
| Ift172 | blue3 |
| Fndc4 | blue4 |
| Zfp512 | blue4 |
| Gpn1 | blue4 |
| Supt7l | blue3 |
| Slc4a1ap | blue4 |
| Mrpl33 | blue4 |
| Rbks | blue4 |
| Mir3473e | blue3 |
| Bre | blue4 |
| Fosl2 | darkgrey |
| Ppp1cb | indianred2 |
| Yes1 | blue4 |
| Pisd | blue4 |
| Prr14l | blue4 |
| Depdc5 | blue3 |
| Ywhah | blue4 |
| Slc5a1 | blue4 |
| Ctbp1 | deeppink |
| Maea | blue4 |
| Uvssa | blue3 |
| Fam53a | blue4 |
| Slbp | blue4 |
| Tmem129 | blue3 |
| Tacc3 | blue4 |
| Fgfr3 | green4 |
| Letm1 | blue4 |
| Gm10059 | blue4 |
| Whsc1 | blue4 |
| Nelfa | blue4 |
| Gm1673 | blue4 |
| Haus3 | blue4 |
| Mxd4 | deeppink1 |
| Rnf4 | green4 |
| Fam193a | blue3 |
| Gm26931 | blue4 |
| Tnip2 | blue4 |
| Sh3bp2 | mistyrose |
| Add1 | blue3 |
| Mfsd10 | blue4 |
| Nop14 | blue4 |
| Grk4 | blue3 |
| Htt | blue4 |
| Rgs12 | blue3 |
| Hgfac | deeppink |
| Dok7 | antiquewhite2 |
| Lrpap1 | blue4 |
| Trmt44 | blue3 |
| Acox3 | blue4 |
| Sh3tc1 | deeppink |
| Ablim2 | green4 |
| Afap1 | blue3 |
| Grpel1 | green4 |
| Tada2b | mistyrose |
| Tbc1d14 | mistyrose |
| D5Ertd579e | coral1 |
| Bloc1s4 | darkgrey |
| Mrfap1 | blue4 |
| Man2b2 | deeppink |
| Wfs1 | blue4 |
| Evc | coral1 |
| Evc2 | green4 |
| Stk32b | blue3 |
| Stx18 | blue4 |
| Nsg1 | darkolivegreen |
| Zbtb49 | blue3 |
| Lyar | blue4 |
| Tmem128 | blue4 |
| Slc2a9 | darkgrey |
| Wdr1 | blue4 |
| Gm22249 | darkgrey |
| Zfp518b | blue3 |
| Hs3st1 | blue4 |
| Gm7816 | darkgrey |
| Rab28 | blue3 |
| Bod1l | blue4 |
| Cpeb2 | darkgrey |
| C1qtnf7 | mistyrose |
| Cc2d2a | blue3 |
| Fbxl5 | coral1 |
| Bst1 | blue4 |
| Cd38 | blue4 |
| Gm16015 | darkgrey |
| Fgfbp1 | blue3 |
| Prom1 | blue4 |
| Tapt1 | deeppink |
| Ldb2 | blue3 |
| Qdpr | blue4 |
| Lap3 | blue4 |
| Med28 | deeppink |
| Lcorl | coral1 |
| Gm7931 | blue4 |
| Slit2 | blue3 |
| Pacrgl | blue4 |
| 5730480H06Rik | blue3 |
| Gm5555 | blue4 |
| Gpr125 | green4 |
| Ppargc1a | firebrick2 |
| Dhx15 | blue4 |
| 9230114K14Rik | blue4 |
| Sod3 | green4 |
| Ccdc149 | blue4 |
| Sepsecs | firebrick2 |
| Pi4k2b | green4 |
| Zcchc4 | blue3 |
| Anapc4 | blue4 |
| Slc34a2 | blue4 |
| Sel1l3 | green4 |
| n-R5s171 | blue4 |
| Smim20 | darkolivegreen |
| Rbpj | blue4 |
| Cckar | blue4 |
| Tbc1d19 | blue4 |
| Stim2 | blue4 |
| Gm8121 | blue4 |
| Pcdh7 | blue4 |
| Arap2 | blue4 |
| 0610040J01Rik | blue4 |
| Rell1 | darkgrey |
| Pgm1 | blue4 |
| Tbc1d1 | blue4 |
| Gm15823 | blue4 |
| Gm20033 | darkgrey |
| Klf3 | darkgrey |
| Tlr1 | blue4 |
| Fam114a1 | blue4 |
| Klhl5 | blue4 |
| Wdr19 | firebrick2 |
| Rfc1 | blue4 |
| Rpl9 | blue4 |
| Lias | deeppink |
| Ugdh | blue4 |
| Smim14 | blue4 |
| Ube2k | blue4 |
| Pds5a | blue4 |
| Gm24399 | blue4 |
| N4bp2os | blue3 |
| N4bp2 | firebrick2 |
| Rbm47 | orangered |
| Apbb2 | blue3 |
| Uchl1 | darkolivegreen |
| Limch1 | lightsteelblue |
| Gm23841 | blue4 |
| Tmem33 | blue4 |
| Slc30a9 | mistyrose |
| Shisa3 | blue4 |
| Atp8a1 | firebrick2 |
| Guf1 | blue4 |
| Gnpda2 | coral1 |
| Commd8 | coral1 |
| Atp10d | blue4 |
| Corin | blue4 |
| Nfxl1 | blue4 |
| Nipal1 | darkgrey |
| Tec | mistyrose |
| Slain2 | deeppink |
| Gm24776 | blue4 |
| Fryl | blue3 |
| Ociad1 | blue4 |
| Ociad2 | blue3 |
| Cwh43 | blue4 |
| Dcun1d4 | blue4 |
| Lrrc66 | darkolivegreen |
| Sgcb | blue4 |
| Usp46 | blue4 |
| Dancr | blue3 |
| Rasl11b | blue4 |
| Scfd2 | blue3 |
| Fip1l1 | blue4 |
| Lnx1 | blue4 |
| Chic2 | blue3 |
| Pdgfra | blue4 |
| Kit | darkolivegreen |
| Kdr | blue3 |
| Srd5a3 | blue4 |
| Tmem165 | blue4 |
| Clock | blue4 |
| Gm7467 | green4 |
| Exoc1 | blue3 |
| Cep135 | blue4 |
| Aasdh | blue3 |
| Ppat | coral1 |
| 2310040G07Rik | blue4 |
| Paics | mistyrose |
| Srp72 | coral1 |
| Arl9 | blue3 |
| Hopx | blue4 |
| Rest | coral1 |
| Noa1 | blue3 |
| Polr2b | coral1 |
| Igfbp7 | darkgrey |
| Hmgn2-ps1 | green4 |
| Cenpc1 | coral1 |
| Stap1 | deeppink |
| Uba6 | blue4 |
| Ythdc1 | deeppink1 |
| Ugt2b34 | blue4 |
| Ugt2b5 | blue3 |
| Ugt2b37 | blue4 |
| Ugt2b38 | blue4 |
| Sult1b1 | blue4 |
| Sult1d1 | mistyrose |
| Igj | blue3 |
| Utp3 | blue4 |
| Rufy3 | firebrick2 |
| Grsf1 | deeppink |
| Mob1b | coral1 |
| Dck | blue4 |
| Slc4a4 | firebrick2 |
| Gc | blue4 |
| Eif5al3-ps | blue4 |
| Cox18 | blue3 |
| Ankrd17 | indianred2 |
| Gm9958 | blue3 |
| Alb | blue3 |
| Afm | darkolivegreen |
| 5830473C10Rik | darkolivegreen |
| Rassf6 | blue4 |
| Ppbp | darkgrey |
| Pf4 | blue4 |
| Cxcl1 | darkgrey |
| Cxcl2 | darkgrey |
| Mthfd2l | blue4 |
| Areg | darkgrey |
| Btc | coral1 |
| Parm1 | blue3 |
| Rchy1 | blue3 |
| THAP6 | firebrick2 |
| Cdkl2 | coral1 |
| G3bp2 | coral1 |
| Gm22728 | darkgrey |
| Uso1 | coral1 |
| Gm15710 | blue4 |
| Gm25290 | blue4 |
| Naaa | saddlebrown |
| Sdad1 | blue4 |
| Cxcl9 | saddlebrown |
| Art3 | saddlebrown |
| Cxcl10 | coral1 |
| Nup54 | blue4 |
| Scarb2 | blue4 |
| Stbd1 | blue3 |
| Shroom3 | blue4 |
| Sowahb | blue4 |
| 11-Sep | blue4 |
| Ccni | blue4 |
| 2010109A12Rik | blue4 |
| Ccng2 | blue3 |
| Cxcl13 | lightcoral |
| Cnot6l | firebrick2 |
| Mrpl1 | coral1 |
| Fras1 | darkolivegreen |
| Anxa3 | blue4 |
| Bmp2k | brown1 |
| Gm5560 | coral1 |
| Antxr2 | blue4 |
| Bmp3 | blue4 |
| Gm3470 | blue4 |
| Rasgef1b | darkgrey |
| Hnrnpd | blue4 |
| Gm17092 | darkgrey |
| 4930524J08Rik | darkgrey |
| Hnrnpdl | blue4 |
| Enoph1 | blue4 |
| Tmem150c | blue4 |
| Sec31a | blue3 |
| 5430416N02Rik | blue4 |
| Lin54 | blue4 |
| Cops4 | blue4 |
| Plac8 | blue4 |
| Coq2 | blue4 |
| Gm15777 | blue3 |
| Helq | blue3 |
| Mrps18c | antiquewhite2 |
| Fam175a | blue4 |
| Gm26286 | blue4 |
| Agpat9 | darkolivegreen |
| Cds1 | blue4 |
| Wdfy3 | blue3 |
| Gm20548 | blue4 |
| Arhgap24 | blue3 |
| Ptpn13 | blue4 |
| Slc10a6 | blue3 |
| Aff1 | darkgrey |
| Klhl8 | blue4 |
| Hsd17b11 | blue3 |
| Nudt9 | darkgrey |
| Sparcl1 | blue4 |
| Spp1 | blue4 |
| Pkd2 | darkolivegreen |
| BC005561 | coral1 |
| D930016D06Rik | blue3 |
| Gbp9 | saddlebrown |
| Gbp4 | saddlebrown |
| Gbp6 | saddlebrown |
| Lrrc8b | brown1 |
| Lrrc8c | mistyrose |
| Lrrc8d | blue4 |
| Rps15a-ps5 | blue4 |
| Zfp326 | deeppink1 |
| Zfp644 | coral1 |
| Gm24191 | blue4 |
| Tgfbr3 | mistyrose |
| Btbd8 | firebrick2 |
| Glmn | coral1 |
| Rpap2 | coral1 |
| Evi5 | deeppink |
| Rpl5 | blue4 |
| Gm22270 | blue4 |
| Gm26387 | blue3 |
| Fam69a | saddlebrown |
| Mtf2 | blue4 |
| Tmed5 | blue4 |
| Dr1 | coral1 |
| Pigg | mistyrose |
| Atp5k | blue4 |
| Pcgf3 | blue4 |
| Gak | blue4 |
| Tmem175 | darkolivegreen |
| Dgkq | blue3 |
| Idua | blue3 |
| Slc26a1 | coral1 |
| Fgfrl1 | blue4 |
| Crlf2 | blue4 |
| 5430403G16Rik | coral1 |
| 4930522L14Rik | coral1 |
| Gm8508 | blue4 |
| Gm26779 | blue3 |
| Gm26808 | darkolivegreen |
| Zfp932 | blue3 |
| Gm17655 | blue3 |
| Plcxd1 | blue4 |
| Gtpbp6 | blue3 |
| Zfp605 | blue4 |
| Chfr | darkgrey |
| Gm15787 | blue3 |
| Golga3 | blue4 |
| Ankle2 | blue4 |
| Pgam5 | darkolivegreen |
| Gm15788 | blue3 |
| Pxmp2 | blue4 |
| Pole | blue4 |
| Fbrsl1 | mistyrose |
| Noc4l | blue4 |
| Ddx51 | blue4 |
| Ep400 | blue4 |
| Mir7026 | blue3 |
| Gm22716 | blue3 |
| Pus1 | blue4 |
| Ulk1 | blue4 |
| Hscb | blue4 |
| Chek2 | blue4 |
| Ttc28 | mistyrose |
| Pitpnb | blue4 |
| Tpst2 | mistyrose |
| Tfip11 | mistyrose |
| Srrd | mistyrose |
| Hps4 | deeppink |
| Gm20636 | blue3 |
| Adrbk2 | blue3 |
| Crybb3 | darkgrey |
| 2900026A02Rik | deeppink |
| Sgsm1 | green4 |
| Wscd2 | green4 |
| Cmklr1 | blue4 |
| Ficd | green4 |
| Sart3 | blue4 |
| Iscu | blue4 |
| Tmem119 | blue4 |
| Selplg | honeydew |
| Coro1c | blue4 |
| Ssh1 | blue4 |
| Dao | blue4 |
| Usp30 | blue4 |
| Alkbh2 | blue3 |
| Ung | blue4 |
| Acacb | darkolivegreen |
| Kctd10 | blue4 |
| Ube3b | green4 |
| Mir7027 | blue3 |
| Mmab | darkolivegreen |
| Mvk | green4 |
| Fam222a | green4 |
| Trpv4 | deeppink |
| Gltp | blue3 |
| Tchp | blue3 |
| Mir7028 | darkgrey |
| Git2 | blue4 |
| Ankrd13a | blue4 |
| 1500011B03Rik | mistyrose |
| 2610524H06Rik | blue4 |
| Oasl2 | blue4 |
| Gm13822 | green4 |
| Oasl1 | darkgrey |
| 2210016L21Rik | blue3 |
| Hnf1a | darkolivegreen |
| Hnf1aos1 | darkolivegreen |
| Hnf1aos2 | blue3 |
| Sppl3 | blue4 |
| Rpl37rt | blue4 |
| Acads | blue4 |
| Unc119b | blue4 |
| Mlec | green4 |
| Pop5 | blue3 |
| Rnf10 | green4 |
| Coq5 | blue3 |
| Dynll1 | blue4 |
| Srsf9 | blue4 |
| Gatc | blue4 |
| Triap1 | blue4 |
| Cox6a1 | indianred2 |
| Sirt4 | blue3 |
| Gm24265 | blue3 |
| Pxn | coral1 |
| Gm13840 | coral1 |
| Rplp0 | blue4 |
| Gcn1l1 | blue3 |
| Rab35 | blue4 |
| Mir7030 | blue4 |
| Ccdc64 | darkolivegreen |
| Gm13841 | blue4 |
| Cit | antiquewhite2 |
| Gm14508 | blue4 |
| Prkab1 | darkgrey |
| Hspb8 | darkgrey |
| Suds3 | blue4 |
| Taok3 | blue4 |
| Pebp1 | blue4 |
| Gm10399 | mistyrose |
| Vsig10 | blue4 |
| Gm15727 | blue4 |
| Wsb2 | darkgrey |
| Rfc5 | blue4 |
| Gm15728 | green4 |
| Ksr2 | coral1 |
| Fbxo21 | darkgrey |
| Tesc | blue3 |
| Fbxw8 | deeppink1 |
| Rnft2 | blue4 |
| 2410131K14Rik | mistyrose |
| Gm25076 | green4 |
| Med13l | darkgrey |
| Tbx3 | blue4 |
| Rbm19 | blue4 |
| Sdsl | blue4 |
| Plbd2 | deeppink |
| Slc8b1 | blue4 |
| Tpcn1 | blue3 |
| Iqcd | blue3 |
| Rita1 | blue4 |
| Ddx54 | mistyrose |
| Rasal1 | lightcoral |
| Oas2 | blue4 |
| Oas1c | blue4 |
| Oas1b | blue4 |
| Oas1a | blue4 |
| Rph3a | green4 |
| Ptpn11 | blue4 |
| Rpl6 | blue4 |
| Gm26205 | blue3 |
| Gm15800 | blue3 |
| Trafd1 | saddlebrown |
| Naa25 | blue4 |
| Erp29 | blue4 |
| Tmem116 | blue4 |
| Mapkapk5 | blue4 |
| Gm15547 | darkgrey |
| Aldh2 | blue4 |
| Acad12 | blue4 |
| Acad10 | blue4 |
| Brap | blue4 |
| Atxn2 | green4 |
| Sh2b3 | darkgrey |
| Mir7031 | blue3 |
| Fam109a | darkgrey |
| Ppp1cc | antiquewhite2 |
| Hvcn1 | darkgrey |
| Tctn1 | blue3 |
| Pptc7 | deeppink |
| Rad9b | blue3 |
| Vps29 | mistyrose |
| Fam216a | blue4 |
| Gpn3 | mistyrose |
| Arpc3 | blue4 |
| Anapc7 | lightsteelblue |
| Gm15846 | darkgrey |
| Atp2a2 | orangered |
| Gm22965 | blue3 |
| Ift81 | coral1 |
| P2rx7 | brown1 |
| P2rx4 | blue4 |
| Camkk2 | blue4 |
| Anapc5 | blue3 |
| Rnf34 | blue4 |
| Kdm2b | blue3 |
| Orai1 | blue4 |
| Gm6444 | deeppink1 |
| Tmem120b | blue4 |
| Rhof | deeppink |
| AI480526 | blue3 |
| RP23-371A16.2 | blue3 |
| Setd1b | blue3 |
| Hpd | mistyrose |
| Psmd9 | blue4 |
| Gm15860 | darkolivegreen |
| Bcl7a | blue3 |
| Gm23458 | blue3 |
| Mlxip | blue4 |
| Gm15751 | blue4 |
| Diablo | blue3 |
| Gm24682 | blue3 |
| Mir7647 | blue3 |
| Vps33a | darkgrey |
| Clip1 | blue4 |
| Zcchc8 | blue3 |
| Rsrc2 | brown1 |
| Hcar2 | darkgrey |
| Hcar1 | blue3 |
| Denr | blue4 |
| Ccdc62 | darkolivegreen |
| Hip1r | mistyrose |
| RP24-492O4.4 | blue3 |
| Vps37b | darkgrey |
| Abcb9 | green4 |
| Gm16001 | blue3 |
| Ogfod2 | blue4 |
| Arl6ip4 | blue4 |
| Pitpnm2 | blue4 |
| Mphosph9 | blue4 |
| 2810006K23Rik | blue3 |
| Cdk2ap1 | blue4 |
| Sbno1 | blue4 |
| RP23-180G16.1 | blue4 |
| Setd8 | blue3 |
| Rilpl2 | deeppink |
| Snrnp35 | blue4 |
| Rilpl1 | blue3 |
| Tmed2 | blue4 |
| Ddx55 | blue3 |
| Eif2b1 | blue4 |
| Gtf2h3 | blue3 |
| Tctn2 | blue3 |
| Atp6v0a2 | blue3 |
| Ccdc92 | green4 |
| Zfp664 | coral1 |
| Fam101a | green4 |
| Ncor2 | mistyrose |
| Scarb1 | blue4 |
| Gm10382 | orangered |
| Dhx37 | blue3 |
| Bri3bp | blue3 |
| Aacs | blue4 |
| Slc15a4 | coral1 |
| Glt1d1 | mistyrose |
| Stx2 | blue3 |
| Ran | blue4 |
| Rps16-ps2 | blue4 |
| Sfswap | blue4 |
| Zfp11 | blue3 |
| Mrps17 | blue4 |
| Gbas | darkolivegreen |
| Psph | blue4 |
| Cct6a | coral1 |
| Gm23245 | darkgrey |
| Sumf2 | blue3 |
| Chchd2 | blue4 |
| Zbed5 | orangered |
| Nupr1l | darkolivegreen |
| Vkorc1l1 | blue4 |
| Gusb | blue3 |
| Asl | blue4 |
| Crcp | coral1 |
| Tpst1 | blue4 |
| Kctd7 | blue4 |
| Rabgef1 | darkgrey |
| Gm15920 | deeppink |
| Tmem248 | darkgrey |
| Sbds | green4 |
| Tyw1 | blue3 |
| Auts2 | mistyrose |
| Gm10051 | blue4 |
| Gm15627 | blue3 |
| Gatsl2 | coral1 |
| Wbscr16 | blue4 |
| Gtf2ird2 | blue3 |
| Ncf1 | blue4 |
| Gtf2i | green4 |
| Gtf2ird1 | blue4 |
| Clip2 | mistyrose |
| Rfc2 | deeppink |
| Lat2 | honeydew |
| Eif4h | blue4 |
| Limk1 | blue4 |
| Eln | mistyrose |
| Wbscr27 | darkolivegreen |
| Cldn4 | darkgrey |
| Cldn3 | darkgrey |
| Abhd11 | blue4 |
| Abhd11os | blue4 |
| Stx1a | blue4 |
| Wbscr22 | blue4 |
| Gm25492 | blue3 |
| Dnajc30 | blue4 |
| Vps37d | blue4 |
| Mlxipl | green4 |
| Tbl2 | blue4 |
| Bcl7b | blue4 |
| Baz1b | blue4 |
| Nsun5 | blue3 |
| Pom121 | blue4 |
| Hip1 | blue4 |
| Rhbdd2 | green4 |
| Por | blue4 |
| Tmem120a | firebrick2 |
| Mdh2 | green4 |
| Hspb1 | darkgrey |
| Ywhag | blue4 |
| Dtx2 | blue4 |
| Upk3b | lightcoral |
| Rasa4 | blue3 |
| Polr2j | blue4 |
| Lrwd1 | blue3 |
| Alkbh4 | blue4 |
| Orai2 | blue4 |
| Prkrip1 | blue4 |
| Sh2b2 | blue4 |
| Cux1 | mistyrose |
| Col26a1 | green4 |
| Ift22 | blue4 |
| 4933404O12Rik | blue3 |
| Fis1 | blue4 |
| Cldn15 | blue4 |
| Znhit1 | deeppink |
| Plod3 | blue4 |
| Vgf | blue4 |
| Ap1s1 | blue4 |
| Serpine1 | blue4 |
| Trim56 | blue4 |
| Ache | green4 |
| Mir8116 | green4 |
| Ufsp1 | brown1 |
| Srrt | blue3 |
| Trip6 | darkgrey |
| Slc12a9 | green4 |
| Ephb4 | blue3 |
| Pop7 | blue4 |
| Gigyf1 | blue3 |
| Gnb2 | blue4 |
| Gm17112 | blue3 |
| Mospd3 | blue4 |
| Pcolce | mistyrose |
| Gm16089 | orangered |
| Agfg2 | darkolivegreen |
| Nyap1 | blue4 |
| Tsc22d4 | mistyrose |
| Ppp1r35 | blue4 |
| Mepce | brown1 |
| Zcwpw1 | blue3 |
| Pilrb1 | blue4 |
| Azgp1 | mistyrose |
| Gm4963 | darkolivegreen |
| Zkscan1 | firebrick2 |
| Zscan21 | blue4 |
| Zfp113 | coral1 |
| Cops6 | blue4 |
| Mcm7 | blue4 |
| Ap4m1 | coral1 |
| Taf6 | blue3 |
| Cnpy4 | blue4 |
| Mblac1 | blue3 |
| Lamtor4 | blue4 |
| Zfp157 | coral1 |
| Zfp68 | coral1 |
| A430033K04Rik | coral1 |
| Fam20c | green4 |
| Pdgfa | darkgrey |
| 6330403L08Rik | darkgrey |
| Prkar1b | blue4 |
| Heatr2 | blue4 |
| Sun1 | blue3 |
| Get4 | blue4 |
| Adap1 | coral1 |
| Cox19 | green4 |
| 3110082I17Rik | blue4 |
| Mir339 | blue3 |
| Gpr146 | blue3 |
| C130050O18Rik | blue3 |
| Gper1 | blue3 |
| Zfand2a | darkgrey |
| Uncx | darkgrey |
| Micall2 | blue4 |
| Ints1 | blue3 |
| Mafk | darkgrey |
| Tmem184a | darkolivegreen |
| Psmg3 | blue3 |
| Elfn1 | green4 |
| Mad1l1 | blue4 |
| Ftsj2 | darkgrey |
| Nudt1 | lightsteelblue |
| Snx8 | green4 |
| Eif3b | blue4 |
| Chst12 | blue4 |
| Lfng | blue4 |
| Ttyh3 | mistyrose |
| Iqce | blue3 |
| Brat1 | blue3 |
| Amz1 | green4 |
| Gna12 | green4 |
| Gm16035 | blue3 |
| Foxk1 | blue3 |
| Ap5z1 | green4 |
| Wipi2 | blue4 |
| Tnrc18 | deeppink |
| Fbxl18 | blue3 |
| Actb | mistyrose |
| Fscn1 | blue4 |
| Rnf216 | blue3 |
| Rbak | coral1 |
| Zfp12 | darkgrey |
| Zfp316 | blue3 |
| E130309D02Rik | blue3 |
| Zdhhc4 | deeppink |
| 0610040B10Rik | blue4 |
| Kdelr2 | blue4 |
| Daglb | green4 |
| Rac1 | blue4 |
| Fam220a | deeppink |
| Cyth3 | darkgrey |
| Usp42 | blue4 |
| D130017N08Rik | blue3 |
| Eif2ak1 | blue4 |
| Aimp2 | blue4 |
| Pms2 | blue4 |
| Ccz1 | coral1 |
| Lmtk2 | blue4 |
| Tecpr1 | blue3 |
| Bri3 | blue4 |
| Baiap2l1 | darkgrey |
| Nptx2 | darkgrey |
| Trrap | blue3 |
| Smurf1 | blue4 |
| Arpc1a | blue4 |
| Arpc1b | blue4 |
| Pdap1 | blue4 |
| Bud31 | blue3 |
| Ptcd1 | darkgrey |
| Cpsf4 | blue4 |
| Atp5j2 | blue4 |
| Zkscan14 | blue3 |
| Zkscan5 | darkgrey |
| Zfp655 | deeppink |
| Zscan25 | darkgrey |
| 1700001J03Rik | darkgrey |
| Rnf6 | coral1 |
| Cdk8 | coral1 |
| Wasf3 | darkolivegreen |
| Usp12 | firebrick2 |
| Rpl21 | blue4 |
| Rasl11a | blue3 |
| Gtf3a | blue3 |
| Mtif3 | darkgrey |
| Lnx2 | darkgrey |
| Polr1d | blue4 |
| Pan3 | blue3 |
| Flt1 | blue4 |
| Pomp | blue4 |
| Slc46a3 | blue4 |
| Gm10167 | brown1 |
| Slc7a1 | darkgrey |
| Ubl3 | blue4 |
| Katnal1 | blue3 |
| Gm15410 | blue4 |
| 1810059H22Rik | blue3 |
| Gm15408 | darkolivegreen |
| Hmgb1 | coral1 |
| Gm8615 | blue4 |
| Uspl1 | darkgrey |
| Alox5ap | honeydew |
| Hsph1 | darkgrey |
| Gm5566 | orangered |
| B3glct | blue4 |
| Fry | blue3 |
| N4bp2l1 | mistyrose |
| N4bp2l2 | coral1 |
| Pds5b | blue3 |
| Kl | blue4 |
| Stard13 | blue4 |
| Rfc3 | blue4 |
| Samd9l | coral1 |
| Hepacam2 | mistyrose |
| Ccdc132 | coral1 |
| Tfpi2 | blue3 |
| Gng11 | blue4 |
| Bet1 | darkgrey |
| Col1a2 | blue4 |
| Casd1 | firebrick2 |
| Sgce | blue4 |
| Peg10 | darkgrey |
| Ppp1r9a | blue3 |
| Pon3 | blue4 |
| Pon2 | coral1 |
| Pdk4 | blue3 |
| Slc25a13 | mistyrose |
| Shfm1 | blue4 |
| Acn9 | coral1 |
| Asns | blue4 |
| C1galt1 | coral1 |
| Mios | coral1 |
| Rpa3 | blue4 |
| Gm16039 | mistyrose |
| A430035B10Rik | blue4 |
| Ica1 | blue4 |
| Ndufa4 | mistyrose |
| Phf14 | blue4 |
| Thsd7a | blue4 |
| Tmem106b | mistyrose |
| Tmem168 | blue4 |
| B630005N14Rik | green4 |
| 2610001J05Rik | antiquewhite2 |
| 1110019D14Rik | firebrick2 |
| Mdfic | blue4 |
| Tfec | darkgrey |
| Tes | darkgrey |
| Cav2 | blue4 |
| Cav1 | blue4 |
| Met | blue4 |
| Capza2 | coral1 |
| St7 | blue4 |
| Gm27960 | darkgrey |
| Cftr | blue4 |
| Cttnbp2 | blue3 |
| Gm26809 | blue4 |
| Lsm8 | blue4 |
| Tspan12 | blue4 |
| Ing3 | deeppink1 |
| Cped1 | blue4 |
| Fam3c | blue4 |
| Aass | mistyrose |
| Cadps2 | blue4 |
| Slc13a1 | blue4 |
| Ndufa5 | blue3 |
| Asb15 | blue3 |
| Wasl | blue4 |
| Tmem229a | mistyrose |
| Pot1a | darkgrey |
| Zfp800 | indianred2 |
| Gcc1 | darkgrey |
| Arf5 | deeppink |
| Snd1 | blue4 |
| Rbm28 | blue3 |
| Impdh1 | blue4 |
| Hilpda | darkgrey |
| Calu | blue4 |
| Ccdc136 | blue4 |
| Flnc | blue4 |
| Atp6v1f | blue4 |
| Kcp | green4 |
| Gm26484 | blue4 |
| Irf5 | blue4 |
| Tnpo3 | blue4 |
| Tspan33 | green4 |
| Smo | brown1 |
| Ahcyl2 | blue4 |
| Nrf1 | mistyrose |
| Ube2h | darkgrey |
| Zc3hc1 | darkgrey |
| Klhdc10 | darkgrey |
| Tmem209 | blue4 |
| Cep41 | blue4 |
| Copg2 | firebrick2 |
| Gm13834 | blue3 |
| Lincpint | blue4 |
| Gm13835 | blue4 |
| 2210408F21Rik | mistyrose |
| Mkln1 | coral1 |
| Podxl | blue4 |
| Chchd3 | blue4 |
| Exoc4 | blue4 |
| Slc35b4 | blue4 |
| Gm13855 | darkolivegreen |
| Akr1b3 | blue4 |
| Akr1b8 | blue4 |
| Akr1b10 | blue4 |
| Akr1b7 | darkgrey |
| Bpgm | darkgrey |
| Cald1 | blue4 |
| Agbl3 | darkolivegreen |
| Tmem140 | saddlebrown |
| 3110062M04Rik | blue3 |
| Wdr91 | darkolivegreen |
| Cnot4 | blue4 |
| Nup205 | blue4 |
| 1810058I24Rik | mistyrose |
| Slc13a4 | blue4 |
| Mtpn | blue4 |
| Creb3l2 | green4 |
| Akr1d1 | mistyrose |
| Gm15487 | blue4 |
| Trim24 | brown1 |
| Svopl | mistyrose |
| Atp6v0a4 | darkgrey |
| Tmem213 | coral1 |
| D630045J12Rik | blue4 |
| Zc3hav1 | blue4 |
| Ttc26 | coral1 |
| Ubn2 | blue4 |
| 1110001J03Rik | blue4 |
| Luc7l2 | blue4 |
| Klrg2 | blue4 |
| Hipk2 | blue4 |
| Tbxas1 | green4 |
| Parp12 | saddlebrown |
| 4930599N23Rik | blue4 |
| Kdm7a | deeppink |
| Slc37a3 | darkolivegreen |
| Rab19 | blue3 |
| Mkrn1 | blue3 |
| Dennd2a | blue4 |
| Adck2 | darkgrey |
| Ndufb2 | darkolivegreen |
| Braf | coral1 |
| Mrps33 | mistyrose |
| Gm26833 | blue4 |
| Agk | blue4 |
| E330009J07Rik | coral1 |
| Ssbp1 | blue4 |
| Mgam | mistyrose |
| Trbc1 | blue4 |
| Trbc2 | blue4 |
| Ephb6 | blue3 |
| Trpv6 | blue4 |
| Trpv5 | blue4 |
| Gstk1 | blue4 |
| Tmem139 | darkolivegreen |
| Casp2 | blue4 |
| Gm25108 | blue4 |
| Zyx | darkgrey |
| Epha1 | blue4 |
| Fam115a | blue4 |
| Arhgef5 | blue4 |
| Tpk1 | mistyrose |
| Cul1 | coral1 |
| Ezh2 | blue4 |
| Gm24975 | blue3 |
| Pdia4 | mistyrose |
| Zfp398 | blue3 |
| Zfp282 | blue4 |
| Zfp212 | darkgrey |
| Zfp956 | blue3 |
| Zfp777 | blue3 |
| Zfp746 | darkgrey |
| Krba1 | blue3 |
| Gm24325 | green4 |
| Zfp467 | blue3 |
| Gm10243 | blue4 |
| Gm7887 | blue4 |
| Atp6v0e2 | coral1 |
| Lrrc61 | green4 |
| Rarres2 | blue4 |
| Repin1 | blue3 |
| Zfp775 | darkgrey |
| AI854703 | green4 |
| Gimap8 | blue3 |
| Gimap9 | coral1 |
| Gimap4 | blue4 |
| Gimap6 | darkgrey |
| Gimap1 | blue3 |
| Gimap5 | darkgrey |
| Gimap3 | saddlebrown |
| Tmem176b | firebrick2 |
| Tmem176a | coral1 |
| Gm7932 | blue4 |
| Aoc1 | blue4 |
| Malsu1 | darkolivegreen |
| Tra2a | blue4 |
| Ccdc126 | blue3 |
| Npy | green4 |
| Mpp6 | coral1 |
| Dfna5 | blue4 |
| Osbpl3 | antiquewhite2 |
| Cycs | blue3 |
| 5430402O13Rik | blue4 |
| 4921507P07Rik | blue4 |
| Nfe2l3 | lightcoral |
| Hnrnpa2b1 | blue4 |
| Cbx3 | blue4 |
| Snx10 | blue4 |
| Skap2 | blue4 |
| Halr1 | blue3 |
| Hotairm1 | blue4 |
| Gm27861 | blue3 |
| Gm27477 | deeppink |
| Hoxaas2 | blue3 |
| Hoxa3 | blue3 |
| Gm15050 | blue3 |
| 5730596B20Rik | blue3 |
| Hoxa4 | blue4 |
| Hoxa5 | antiquewhite2 |
| Hoxa6 | green4 |
| Hoxa7 | blue3 |
| Hoxa9 | blue3 |
| Hoxa10 | blue3 |
| Hoxa11 | blue4 |
| Gm8129 | blue4 |
| Hibadh | mistyrose |
| Tax1bp1 | deeppink |
| Creb5 | darkgrey |
| Tril | blue3 |
| Chn2 | blue4 |
| Prr15 | darkgrey |
| Wipf3 | blue4 |
| Scrn1 | blue3 |
| Fkbp14 | blue4 |
| Plekha8 | blue4 |
| Gm28402 | blue4 |
| Mturn | blue4 |
| Znrf2 | deeppink |
| Nod1 | blue3 |
| Ggct | blue4 |
| Gars | blue4 |
| Inmt | blue3 |
| Fam188b | blue4 |
| Aqp1 | darkolivegreen |
| Adcyap1r1 | blue4 |
| Lsm5 | blue4 |
| Avl9 | green4 |
| Kbtbd2 | coral1 |
| Fkbp9 | blue3 |
| Nt5c3 | coral1 |
| 4930533I22Rik | darkolivegreen |
| Ppm1k | firebrick2 |
| Herc6 | saddlebrown |
| Pyurf | blue3 |
| Lancl2 | blue4 |
| Vopp1 | blue4 |
| Rps15-ps2 | blue4 |
| Abcg2 | coral1 |
| Herc3 | coral1 |
| Nap1l5 | blue4 |
| Fam13a | firebrick2 |
| Tigd2 | blue4 |
| Snca | mistyrose |
| Mmrn1 | blue4 |
| Ccser1 | blue4 |
| Smarcad1 | blue4 |
| Hpgds | blue4 |
| Ndnf | blue3 |
| Prdm5 | mistyrose |
| Gm23543 | brown1 |
| Mad2l1 | blue4 |
| Gng12 | coral1 |
| Gadd45a | darkgrey |
| E230016M11Rik | deeppink1 |
| Gm8566 | mistyrose |
| Serbp1 | blue4 |
| Tacstd2 | blue4 |
| Igkv2-137 | blue4 |
| Igkv1-117 | blue3 |
| Igkv1-110 | blue3 |
| Igkv8-27 | darkgrey |
| Igkc | blue3 |
| Rpia | blue4 |
| Eif2ak3 | darkgrey |
| Rpl34-ps1 | blue4 |
| Thnsl2 | blue4 |
| Fabp1 | darkolivegreen |
| Mir8112 | lightsteelblue |
| Krcc1 | coral1 |
| Cd8a | green4 |
| Rmnd5a | mistyrose |
| Rnf103 | blue3 |
| Chmp3 | blue4 |
| Kdm3a | deeppink |
| Mrpl35 | blue4 |
| Immt | deeppink |
| Ptcd3 | coral1 |
| Gm22486 | blue3 |
| Polr1a | blue4 |
| St3gal5 | darkgrey |
| Atoh8 | blue3 |
| Usp39 | blue4 |
| Tmem150a | green4 |
| Rnf181 | darkgrey |
| Vamp5 | blue3 |
| Vamp8 | blue4 |
| Ggcx | green4 |
| Mat2a | firebrick2 |
| Capg | blue4 |
| Elmod3 | blue4 |
| Retsat | blue4 |
| Tgoln1 | coral1 |
| Tcf7l1 | blue3 |
| Kcmf1 | blue4 |
| Tmsb10 | blue4 |
| Suclg1 | mistyrose |
| Ctnna2 | mistyrose |
| Gcfc2 | blue3 |
| Mrpl19 | blue4 |
| Eva1a | blue3 |
| Pole4 | blue4 |
| Hk2 | darkgrey |
| Dok1 | blue4 |
| Loxl3 | blue4 |
| Htra2 | blue4 |
| Aup1 | deeppink |
| Dqx1 | darkolivegreen |
| Pcgf1 | blue3 |
| Ccdc142 | blue3 |
| Mogs | blue3 |
| Wbp1 | blue4 |
| Ino80b | deeppink |
| Rtkn | blue3 |
| Wdr54 | blue3 |
| 1700003E16Rik | darkolivegreen |
| Dctn1 | green4 |
| Mthfd2 | blue4 |
| Mob1a | blue4 |
| Bola3 | blue4 |
| Tet3 | darkgrey |
| Dguok | blue3 |
| Actg2 | lightcoral |
| Stambp | blue4 |
| Atp6v1b1 | darkolivegreen |
| Tex261 | deeppink |
| Nagk | blue4 |
| Paip2b | coral1 |
| Zfml | blue4 |
| Dysf | blue4 |
| Cyp26b1 | darkgrey |
| Exoc6b | blue4 |
| Npm3-ps1 | blue4 |
| Spr | blue4 |
| Spr-ps1 | darkolivegreen |
| Emx1 | blue4 |
| Sfxn5 | darkolivegreen |
| Rab11fip5 | deeppink |
| Smyd5 | blue4 |
| Pradc1 | deeppink |
| Cct7 | blue4 |
| Alms1 | blue3 |
| Gm4477 | darkolivegreen |
| Gm27317 | blue3 |
| Cml3 | darkolivegreen |
| Alms1-ps2 | darkolivegreen |
| Gm11128 | darkolivegreen |
| Cml5 | mistyrose |
| Nat8 | blue4 |
| Gm9769 | blue4 |
| Cml2 | blue3 |
| 1700019G17Rik | blue3 |
| Cml1 | blue4 |
| Tprkb | darkolivegreen |
| Dusp11 | blue3 |
| Tgfa | blue4 |
| Gm10443 | blue4 |
| Fam136a | blue4 |
| Snrpg | blue4 |
| Pcyox1 | blue3 |
| Tia1 | blue3 |
| C87436 | coral1 |
| Pcbp1 | mistyrose |
| 1600020E01Rik | blue3 |
| Asprv1 | blue4 |
| Mxd1 | darkgrey |
| Snrnp27 | blue3 |
| Gmcl1 | blue3 |
| Anxa4 | blue4 |
| 2610306M01Rik | blue4 |
| Aak1 | blue4 |
| Nfu1 | blue4 |
| Gfpt1 | blue4 |
| Antxr1 | blue4 |
| Aplf | blue4 |
| Rab43 | mistyrose |
| Isy1 | blue3 |
| Cnbp | green4 |
| Copg1 | blue3 |
| Hmces | blue4 |
| Rab7 | darkgrey |
| Rpn1 | deeppink |
| Gata2 | blue3 |
| Eefsec | coral1 |
| Ruvbl1 | blue4 |
| Sec61a1 | mistyrose |
| Mgll | blue3 |
| Abtb1 | blue4 |
| Mcm2 | blue4 |
| Tpra1 | blue3 |
| Plxna1 | blue4 |
| Chchd6 | coral1 |
| Txnrd3 | blue4 |
| Chst13 | blue4 |
| Uroc1 | darkolivegreen |
| Zxdc | blue4 |
| Klf15 | blue4 |
| Aldh1l1 | blue4 |
| Slc41a3 | mistyrose |
| Iqsec1 | green4 |
| Nup210 | blue4 |
| Hdac11 | darkolivegreen |
| Fbln2 | blue4 |
| 1810044D09Rik | darkolivegreen |
| Chchd4 | blue4 |
| Tmem43 | blue4 |
| Xpc | darkgrey |
| Lsm3 | blue4 |
| Slc6a6 | darkgrey |
| Grip2 | green4 |
| Ccdc174 | brown1 |
| Fgd5 | blue4 |
| Nr2c2 | coral1 |
| Mrps25 | blue4 |
| Zfyve20 | lightsteelblue |
| Prickle2 | blue4 |
| Adamts9 | darkgrey |
| A730049H05Rik | blue4 |
| Magi1 | blue4 |
| Slc25a26 | darkgrey |
| Lrig1 | blue3 |
| Kbtbd8 | darkgrey |
| Suclg2 | deeppink |
| Eogt | blue3 |
| Tmf1 | coral1 |
| Gm26175 | blue4 |
| Uba3 | blue4 |
| Arl6ip5 | blue4 |
| Frmd4b | brown1 |
| Mitf | firebrick2 |
| Foxp1 | blue4 |
| Eif4e3 | blue4 |
| Gpr27 | blue4 |
| Tpt1-ps3 | blue4 |
| Rybp | darkgrey |
| Shq1 | blue4 |
| Gm6565 | coral1 |
| Ppp4r2 | blue4 |
| Pdzrn3 | blue4 |
| Il5ra | green4 |
| Trnt1 | coral1 |
| Crbn | mistyrose |
| Lrrn1 | blue3 |
| Setmar | mistyrose |
| Sumf1 | mistyrose |
| Gm4604 | blue4 |
| Itpr1 | firebrick2 |
| 0610040F04Rik | mistyrose |
| Bhlhe40 | darkgrey |
| Arl8b | blue4 |
| Edem1 | blue4 |
| Lmcd1 | darkgrey |
| Rad18 | blue4 |
| Srgap3 | blue4 |
| Thumpd3 | blue4 |
| Gt(ROSA)26Sor | blue4 |
| Setd5 | blue4 |
| Mtmr14 | blue4 |
| Gm16161 | blue3 |
| Brpf1 | darkgrey |
| Ogg1 | blue4 |
| Camk1 | blue3 |
| Tada3 | green4 |
| Arpc4 | mistyrose |
| Ttll3 | blue3 |
| Rpusd3 | deeppink |
| Cidec | lightcoral |
| Jagn1 | blue4 |
| Il17re | blue3 |
| Il17rc | blue4 |
| Creld1 | deeppink |
| Emc3 | blue4 |
| Fancd2os | blue4 |
| Brk1 | blue4 |
| Vhl | blue3 |
| Irak2 | darkgrey |
| Tatdn2 | mistyrose |
| Gm26982 | blue3 |
| Ghrl | darkgrey |
| Sec13 | blue4 |
| Atg7 | blue4 |
| Vgll4 | coral1 |
| Tamm41 | blue4 |
| Syn2 | darkolivegreen |
| Pparg | lightcoral |
| Tsen2 | blue3 |
| Mkrn2os | blue4 |
| Mkrn2 | coral1 |
| Raf1 | coral1 |
| Cand2 | blue3 |
| Rpl32 | blue4 |
| Snora7a | blue4 |
| Efcab12 | green4 |
| Mbd4 | darkolivegreen |
| Ift122 | blue4 |
| Plxnd1 | blue3 |
| Tmcc1 | blue4 |
| Gm8203 | blue4 |
| Fam21 | blue4 |
| 8-Mar | blue4 |
| Zfp422 | darkgrey |
| 8430408G22Rik | mistyrose |
| Tmem72 | darkolivegreen |
| Cxcl12 | darkolivegreen |
| Zfp637 | blue3 |
| Zfp239 | blue4 |
| Hnrnpf | blue4 |
| 4933440N22Rik | blue3 |
| Fxyd4 | blue3 |
| Gm25535 | blue3 |
| Csgalnact2 | coral1 |
| Gm23908 | blue4 |
| Bms1 | blue4 |
| Zfp248 | blue3 |
| Ankrd26 | blue3 |
| Dcp1b | blue3 |
| Lrtm2 | blue3 |
| Adipor2 | blue3 |
| Wnt5b | blue3 |
| Fbxl14 | blue3 |
| Erc1 | blue4 |
| 3110021A11Rik | blue3 |
| Rad52 | blue4 |
| Wnk1 | mistyrose |
| B4galnt3 | coral1 |
| D330020A13Rik | blue3 |
| Ccdc77 | blue4 |
| Kdm5a | brown1 |
| Il17ra | deeppink |
| Cecr5 | darkolivegreen |
| Atp6v1e1 | blue4 |
| Bcl2l13 | blue3 |
| Bid | brown1 |
| Mical3 | blue3 |
| Pex26 | darkgrey |
| Usp18 | saddlebrown |
| Slc6a13 | blue4 |
| Gm24855 | darkgrey |
| Slc6a12 | darkolivegreen |
| M6pr | blue4 |
| Phc1 | green4 |
| Gm8430 | blue4 |
| Apobec1 | honeydew |
| Gm4482 | blue4 |
| Foxj2 | blue3 |
| Necap1 | blue3 |
| Clec4a1 | honeydew |
| Clec4a3 | saddlebrown |
| Clec4a2 | saddlebrown |
| Clec4d | blue4 |
| Pex5 | antiquewhite2 |
| C1rl | darkolivegreen |
| C1ra | honeydew |
| C1s1 | darkgrey |
| Lpcat3 | blue4 |
| Emg1 | blue3 |
| Phb2 | deeppink |
| Gm29008 | blue4 |
| Ptpn6 | green4 |
| Grcc10 | green4 |
| Atn1 | green4 |
| Eno2 | blue4 |
| Spsb2 | darkgrey |
| Tpi1 | blue4 |
| Usp5 | deeppink |
| Cdca3 | blue3 |
| Leprel2 | blue4 |
| Gpr162 | green4 |
| Ptms | green4 |
| A230083G16Rik | blue4 |
| Mlf2 | deeppink |
| Cops7a | blue4 |
| Gm23851 | blue3 |
| Zfp384 | mistyrose |
| Ing4 | blue3 |
| Acrbp | blue3 |
| Chd4 | blue4 |
| Nop2 | blue4 |
| Iffo1 | blue3 |
| Gapdh | firebrick2 |
| Ncapd2 | blue4 |
| Mrpl51 | coral1 |
| Mir3098 | darkolivegreen |
| Vamp1 | blue3 |
| Tapbpl | saddlebrown |
| Gm24175 | blue4 |
| Ltbr | blue4 |
| Scnn1a | blue4 |
| Tnfrsf1a | blue4 |
| Plekhg6 | blue4 |
| Cd9 | blue4 |
| Vwf | lightcoral |
| Ntf3 | darkgrey |
| Kcna5 | blue3 |
| Ndufa9 | blue4 |
| D6Wsu163e | blue4 |
| 9630033F20Rik | blue4 |
| Ccnd2 | blue4 |
| Parp11 | blue3 |
| Tspan9 | mistyrose |
| Tead4 | blue4 |
| Tulp3 | green4 |
| Rhno1 | blue4 |
| Foxm1 | blue4 |
| Nrip2 | blue3 |
| Itfg2 | coral1 |
| Fkbp4 | green4 |
| Pzp | blue3 |
| Clec2h | blue4 |
| BC064078 | firebrick2 |
| Clec2d | darkgrey |
| Gm27514 | blue4 |
| Clec1a | coral1 |
| Clec7a | saddlebrown |
| Tmem52b | mistyrose |
| 1700101I11Rik | blue4 |
| Gabarapl1 | blue3 |
| Klra2 | honeydew |
| Magohb | blue4 |
| Ybx3 | blue4 |
| Gm22362 | darkgrey |
| 2700089E24Rik | blue4 |
| Kap | mistyrose |
| Etv6 | blue4 |
| Lrp6 | firebrick2 |
| Mansc1 | blue3 |
| Loh12cr1 | green4 |
| Dusp16 | darkgrey |
| Crebl2 | darkgrey |
| Gpr19 | blue3 |
| Cdkn1b | mistyrose |
| 1190002F15Rik | blue3 |
| Apold1 | coral1 |
| Ddx47 | blue4 |
| Gprc5a | darkgrey |
| Hebp1 | blue4 |
| 8430419L09Rik | blue3 |
| Dynlt1-ps1 | blue4 |
| Emp1 | darkgrey |
| Rpl36a-ps3 | blue4 |
| Atf7ip | blue4 |
| Plbd1 | saddlebrown |
| H2afj | blue3 |
| Wbp11 | mistyrose |
| Smco3 | coral1 |
| Art4 | blue3 |
| Mgp | honeydew |
| Arhgdib | blue4 |
| Rerg | blue4 |
| Ptpro | blue4 |
| Eps8 | darkgrey |
| Strap | blue4 |
| Dera | blue3 |
| Mgst1 | blue4 |
| Rergl | mistyrose |
| Pik3c2g | blue4 |
| Plekha5 | coral1 |
| Aebp2 | blue4 |
| Rpl38-ps2 | blue4 |
| Pde3a | blue4 |
| Slco1a4 | blue3 |
| Slco1a1 | blue3 |
| Gm6614 | mistyrose |
| Slco1a6 | mistyrose |
| Gm18159 | blue3 |
| Gm20400 | blue3 |
| Pyroxd1 | deeppink |
| Recql | blue4 |
| Golt1b | blue4 |
| Gys2 | blue4 |
| Ldhb | blue4 |
| Kcnj8 | darkgrey |
| Abcc9 | blue4 |
| Cmas | coral1 |
| Gm7457 | darkolivegreen |
| St8sia1 | lightsteelblue |
| C2cd5 | blue3 |
| Etnk1 | firebrick2 |
| Bcat1 | blue4 |
| Gm15687 | blue4 |
| Lrmp | honeydew |
| Lyrm5 | mistyrose |
| Kras | blue4 |
| Gm15706 | blue3 |
| Rps25-ps1 | blue4 |
| Rassf8 | darkgrey |
| Bhlhe41 | darkgrey |
| Sspn | blue4 |
| Itpr2 | lightsteelblue |
| Asun | blue4 |
| Fgfr1op2 | blue4 |
| Tm7sf3 | blue4 |
| Med21 | blue4 |
| Stk38l | blue4 |
| Ppfibp1 | blue4 |
| Mrps35 | mistyrose |
| Mansc4 | blue4 |
| Klhl42 | darkgrey |
| Ccdc91 | blue4 |
| Ergic2 | green4 |
| Tmtc1 | deeppink |
| Ipo8 | blue4 |
| Caprin2 | blue3 |
| Fam60a | blue4 |
| Dennd5b | deeppink1 |
| Mettl20 | mistyrose |
| Amn1 | mistyrose |
| 2810474O19Rik | blue3 |
| Gm21814 | blue4 |
| Myadm | blue4 |
| Tarm1 | darkolivegreen |
| Ndufa3 | blue4 |
| Tfpt | blue4 |
| Prpf31 | blue4 |
| Cnot3 | blue3 |
| Leng1 | blue4 |
| Tmc4 | green4 |
| Mboat7 | blue3 |
| Tsen34 | deeppink |
| Rps9 | blue4 |
| Pirb | honeydew |
| Lair1 | saddlebrown |
| Leng8 | blue3 |
| Leng9 | coral1 |
| Cdc42ep5 | blue4 |
| Lilra5 | blue4 |
| Rdh13 | blue3 |
| Eps8l1 | mistyrose |
| Ppp1r12c | mistyrose |
| Tmem86b | coral1 |
| Ppp6r1 | green4 |
| Hspbp1 | deeppink |
| Suv420h2 | green4 |
| Cox6b2 | blue3 |
| Tmem190 | blue4 |
| Tmem238 | blue4 |
| Rpl28 | blue4 |
| Ube2s | coral1 |
| Shisa7 | green4 |
| Isoc2b | blue4 |
| Isoc2a | blue4 |
| Zfp628 | deeppink |
| Nat14 | blue3 |
| Zfp579 | blue3 |
| Fiz1 | deeppink |
| Zfp524 | blue3 |
| Zfp865 | blue3 |
| Zfp784 | blue3 |
| Gm15510 | blue3 |
| Zfp580 | blue4 |
| Ccdc106 | green4 |
| U2af2 | mistyrose |
| Epn1 | green4 |
| Rasl2-9 | blue4 |
| Zfp787 | blue4 |
| Zfp444 | blue4 |
| Zfp667 | blue3 |
| Zfp78 | coral1 |
| Zfp28 | blue3 |
| Peg3 | indianred2 |
| Zfp954 | green4 |
| Zfp418 | coral1 |
| Zfp772 | blue4 |
| Gm18190 | blue3 |
| Clcn4-2 | firebrick2 |
| Mir5620 | darkgrey |
| Gm6900 | blue4 |
| Zfp551 | firebrick2 |
| Zfp606 | coral1 |
| Zfp329 | blue3 |
| Zfp110 | brown1 |
| Zfp128 | coral1 |
| Zscan22 | blue3 |
| Rps5 | blue4 |
| Zfp324 | blue3 |
| Zfp446 | green4 |
| Zbtb45 | blue4 |
| Trim28 | blue4 |
| Chmp2a | blue4 |
| Ube2m | deeppink |
| Mzf1 | blue3 |
| 6330408A02Rik | blue3 |
| Lig1 | blue4 |
| Gm8086 | blue4 |
| Sepw1 | blue4 |
| Gltscr2 | firebrick2 |
| Snord23 | green4 |
| Ehd2 | blue4 |
| Gltscr1 | blue3 |
| Napa | darkgrey |
| Kptn | green4 |
| Meis3 | blue4 |
| Dhx34 | blue3 |
| C5ar1 | blue4 |
| Inafm1 | blue4 |
| Ccdc9 | darkgrey |
| Bbc3 | darkgrey |
| Sae1 | blue4 |
| Zc3h4 | blue3 |
| Tmem160 | firebrick2 |
| Arhgap35 | blue3 |
| Ap2s1 | blue4 |
| Slc1a5 | blue4 |
| Fkrp | brown1 |
| Strn4 | blue3 |
| Prkd2 | blue4 |
| Dact3 | blue4 |
| Ptgir | blue4 |
| Calm3 | deeppink |
| Pnmal2 | green4 |
| Ppp5c | blue3 |
| Hif3a | saddlebrown |
| Mill2 | blue3 |
| Pglyrp1 | blue4 |
| Ccdc61 | blue3 |
| Mypop | blue3 |
| Mypopos | blue3 |
| Irf2bp1 | blue3 |
| Sympk | blue3 |
| Dmwd | blue3 |
| Dmpk | blue3 |
| Six5 | blue3 |
| Fbxo46 | blue3 |
| Qpctl | blue3 |
| Snrpd2 | blue4 |
| Eml2 | blue4 |
| Gpr4 | darkgrey |
| Opa3 | deeppink |
| Gm25134 | blue4 |
| Vasp | blue4 |
| 1700058P15Rik | blue4 |
| Fosb | coral1 |
| Ercc1 | darkgrey |
| Cd3eap | blue4 |
| Ppp1r13l | darkgrey |
| Ercc2 | blue3 |
| Klc3 | blue4 |
| Mark4 | blue3 |
| Exoc3l2 | blue3 |
| Bloc1s3 | blue3 |
| Trappc6a | blue4 |
| Ppp1r37 | coral1 |
| Gemin7 | deeppink |
| Zfp296 | blue3 |
| Clasrp | blue3 |
| Relb | darkgrey |
| Clptm1 | coral1 |
| Apoc1 | lightcoral |
| Apoe | darkgrey |
| Tomm40 | blue4 |
| Pvrl2 | mistyrose |
| Bcam | blue4 |
| Cblc | blue4 |
| Bcl3 | blue4 |
| Gm16174 | blue4 |
| Pvr | darkgrey |
| Gm16184 | darkgrey |
| Gm10175 | lightsteelblue |
| Zfp180 | blue4 |
| Zfp235 | blue3 |
| Zfp111 | coral1 |
| Zfp109 | blue3 |
| Zfp108 | mistyrose |
| Zfp93 | darkgrey |
| Zfp61 | coral1 |
| Zfp94 | firebrick2 |
| Smg9 | blue4 |
| Plaur | darkgrey |
| Cadm4 | coral1 |
| Zfp428 | mistyrose |
| Irgq | mistyrose |
| Xrcc1 | blue3 |
| Ethe1 | blue4 |
| Phldb3 | mistyrose |
| Lypd3 | darkgrey |
| Rps19 | blue4 |
| Arhgef1 | blue3 |
| Rabac1 | firebrick2 |
| Grik5 | green4 |
| Zfp574 | mistyrose |
| Dedd2 | blue3 |
| Mir7048 | darkgrey |
| Zfp526 | blue3 |
| Gsk3a | deeppink |
| 9130221H12Rik | blue3 |
| Erf | darkgrey |
| Cic | mistyrose |
| Pafah1b3 | blue4 |
| Tmem145 | blue4 |
| Megf8 | blue3 |
| Lipe | lightcoral |
| Ceacam1 | blue4 |
| Ceacam2 | darkolivegreen |
| Erich4 | blue4 |
| Atp5sl | darkolivegreen |
| B3gnt8 | blue4 |
| Bckdha | blue4 |
| Exosc5 | deeppink |
| B9d2 | mistyrose |
| Tgfb1 | blue4 |
| Ccdc97 | deeppink |
| Hnrnpul1 | blue4 |
| Axl | blue4 |
| Cyp2s1 | blue4 |
| Cyp2b10 | mistyrose |
| Cyp2a4 | mistyrose |
| Cyp2a5 | mistyrose |
| Cyp2f2 | blue4 |
| Gm15883 | blue4 |
| Cyp2t4 | darkgrey |
| Egln2 | coral1 |
| Rab4b | blue3 |
| Snrpa | mistyrose |
| BC024978 | blue3 |
| Mir1191 | blue3 |
| Itpkc | darkgrey |
| Adck4 | blue4 |
| Ltbp4 | coral1 |
| Shkbp1 | blue4 |
| Blvrb | blue4 |
| Pgam1-ps2 | blue4 |
| Sertad3 | blue3 |
| Sertad1 | darkgrey |
| Prx | deeppink |
| Pld3 | deeppink |
| 2310022A10Rik | blue4 |
| Akt2 | deeppink |
| Map3k10 | brown1 |
| Zfp60 | darkgrey |
| Zfp59 | coral1 |
| Zfp607 | darkgrey |
| 1700049G17Rik | blue3 |
| Zfp780b | coral1 |
| Psmc4 | blue4 |
| Fcgbp | green4 |
| Fbl | blue4 |
| Dyrk1b | blue4 |
| Eid2 | blue3 |
| Eid2b | mistyrose |
| Timm50 | deeppink |
| Supt5 | blue3 |
| Rps16 | blue4 |
| AF357399 | blue4 |
| Plekhg2 | blue4 |
| Zfp36 | coral1 |
| Med29 | blue4 |
| Paf1 | blue4 |
| Pak4 | mistyrose |
| Gmfg | blue4 |
| Lrfn1 | blue3 |
| Sycn | blue4 |
| Nccrp1 | green4 |
| Mir7049 | blue4 |
| Fbxo17 | blue3 |
| Mrps12 | deeppink |
| Sars2 | blue4 |
| Nfkbib | darkgrey |
| Sirt2 | green4 |
| Rinl | blue4 |
| Hnrnpl | blue4 |
| Ech1 | blue4 |
| Lgals4 | blue3 |
| Actn4 | blue4 |
| Eif3k | deeppink |
| Fam98c | blue3 |
| Spred3 | blue4 |
| Psmd8 | darkolivegreen |
| Yif1b | coral1 |
| 2200002D01Rik | blue4 |
| Spint2 | blue4 |
| Ppp1r14a | blue4 |
| Sipa1l3 | blue3 |
| Zfp84 | firebrick2 |
| Mir1964 | lightsteelblue |
| Zfp30 | firebrick2 |
| Zfp790 | coral1 |
| Zfp940 | blue3 |
| Zfp420 | blue3 |
| Zfp27 | blue3 |
| ZNF383 | mistyrose |
| Zfp74 | blue3 |
| Gm10169 | blue4 |
| Zfp568 | darkgrey |
| Zfp14 | blue3 |
| Zfp566 | blue3 |
| Zfp260 | coral1 |
| Gm23368 | blue4 |
| Zfp146 | blue4 |
| Gm5113 | darkgrey |
| Cox7a1 | blue4 |
| Capns1 | blue4 |
| Tbcb | blue4 |
| Polr2i | deeppink |
| Wdr62 | blue4 |
| Rps12-ps5 | blue4 |
| Alkbh6 | blue3 |
| Syne4 | green4 |
| Sdhaf1 | coral1 |
| Lrfn3 | blue3 |
| Tyrobp | honeydew |
| Hcst | saddlebrown |
| Nfkbid | blue4 |
| Aplp1 | blue4 |
| Nphs1 | orangered |
| Prodh2 | blue4 |
| Arhgap33 | blue3 |
| Proser3 | blue3 |
| Hspb6 | blue4 |
| Lin37 | blue4 |
| Psenen | deeppink |
| U2af1l4 | blue3 |
| Kmt2b | blue3 |
| Zbtb32 | blue3 |
| Upk1a | lightcoral |
| Cox6b1 | blue4 |
| Haus5 | blue4 |
| 2200002J24Rik | blue4 |
| Atp4a | blue4 |
| Tmem147 | blue4 |
| C630016N16Rik | blue3 |
| Gapdhs | blue3 |
| Gm26935 | blue3 |
| Ffar3 | coral1 |
| Usf2 | deeppink |
| Gm4673 | blue3 |
| Lsr | blue4 |
| Fam187b | green4 |
| Fxyd5 | blue4 |
| Fxyd1 | blue3 |
| Hpn | blue4 |
| Scn1b | blue4 |
| Gramd1a | blue3 |
| RP24-427N13.6 | blue3 |
| Wtip | blue4 |
| Uba2 | blue4 |
| Pdcd2l | blue4 |
| Gpi1 | deeppink |
| Gm12762 | green4 |
| 4931406P16Rik | blue4 |
| Lsm14a | blue4 |
| Kctd15 | blue4 |
| Gm12781 | blue4 |
| Chst8 | coral1 |
| Pepd | blue4 |
| Cebpg | blue4 |
| Cebpa | blue4 |
| Lrp3 | green4 |
| Gpatch1 | blue4 |
| Rhpn2 | darkgrey |
| C230052I12Rik | blue4 |
| Cep89 | darkolivegreen |
| Slc7a9 | blue4 |
| Nudt19 | blue4 |
| Ankrd27 | blue4 |
| Pdcd5 | blue4 |
| Dpy19l3 | blue4 |
| Zfp507 | blue4 |
| Uri1 | coral1 |
| 1600014C10Rik | blue4 |
| Plekhf1 | coral1 |
| Pop4 | blue4 |
| Zfp939 | blue3 |
| Gm4790 | coral1 |
| 6230415J03Rik | blue3 |
| Gm28582 | blue3 |
| Gm6851 | blue4 |
| AI987944 | coral1 |
| AW146154 | coral1 |
| Gm15470 | coral1 |
| 2610021A01Rik | blue3 |
| Zfp788 | blue4 |
| Zfp141 | blue4 |
| Gm21028 | blue3 |
| 9830147E19Rik | coral1 |
| Gm5595 | coral1 |
| Zfp715 | blue4 |
| Nkg7 | saddlebrown |
| Etfb | blue4 |
| Vsig10l | blue3 |
| Cd33 | honeydew |
| Zfp658 | coral1 |
| Zfp719 | coral1 |
| Siglece | honeydew |
| Ctu1 | blue4 |
| Klk1b9 | blue4 |
| Klk1b27 | darkolivegreen |
| Klk1b22 | blue4 |
| Klk1b3 | blue4 |
| Klk1b5 | blue3 |
| Gm10109 | mistyrose |
| Klk1 | blue4 |
| 1700028J19Rik | green4 |
| Gm25386 | deeppink1 |
| 2410002F23Rik | darkgrey |
| Snord88c | blue4 |
| Clec11a | green4 |
| Syt3 | darkolivegreen |
| Aspdh | blue4 |
| Josd2 | green4 |
| Mir7052 | blue3 |
| Emc10 | deeppink |
| Fam71e1 | blue3 |
| Pold1 | blue4 |
| Nr1h2 | coral1 |
| Napsa | blue4 |
| Kcnc3 | blue3 |
| Myh14 | blue4 |
| 2310016G11Rik | blue4 |
| Vrk3 | blue3 |
| Atf5 | blue4 |
| Nup62 | blue4 |
| Tbc1d17 | blue4 |
| Akt1s1 | deeppink |
| Pnkp | blue3 |
| Ptov1 | mistyrose |
| Med25 | mistyrose |
| Fuz | darkolivegreen |
| Ap2a1 | blue4 |
| Cpt1c | blue3 |
| Prmt1 | blue4 |
| Gm15545 | blue3 |
| Bcl2l12 | blue4 |
| Irf3 | blue3 |
| Scaf1 | mistyrose |
| Rras | blue4 |
| Prr12 | blue3 |
| Prrg2 | green4 |
| Nosip | deeppink |
| Rcn3 | blue4 |
| Fcgrt | blue4 |
| Rps11 | blue4 |
| Snord35b | blue4 |
| Rpl13a | blue4 |
| Snord35a | blue3 |
| Flt3l | blue3 |
| Aldh16a1 | blue4 |
| Pih1d1 | deeppink |
| Tead2 | blue4 |
| Cd37 | blue4 |
| Trpm4 | green4 |
| Rpl14-ps1 | blue4 |
| Lin7b | antiquewhite2 |
| Snrnp70 | mistyrose |
| Gm22478 | blue3 |
| Ruvbl2 | blue4 |
| Gys1 | blue3 |
| Ftl1 | blue3 |
| Bax | blue4 |
| Dhdh | mistyrose |
| Nucb1 | green4 |
| Ppp1r15a | coral1 |
| Plekha4 | blue3 |
| Hsd17b14 | green4 |
| 0610005C13Rik | green4 |
| Bcat2 | darkolivegreen |
| Rasip1 | darkgrey |
| Gm16047 | darkgrey |
| Mamstr | darkgrey |
| Car11 | blue3 |
| Dbp | blue4 |
| Sphk2 | coral1 |
| Rpl18 | blue4 |
| Sult2b1 | blue4 |
| Lmtk3 | blue3 |
| Cyth2 | mistyrose |
| Grwd1 | blue4 |
| Kdelr1 | deeppink |
| Tmem143 | darkolivegreen |
| Emp3 | blue4 |
| Ccdc114 | blue3 |
| Abcc6 | green4 |
| Nomo1 | deeppink |
| Ush1c | green4 |
| Sergef | blue4 |
| Saal1 | blue4 |
| Saa1 | blue4 |
| Saa2 | lightcoral |
| Hps5 | blue4 |
| Gtf2h1 | coral1 |
| Gm9392 | blue3 |
| Ldha | coral1 |
| Tsg101 | blue4 |
| Uevld | blue4 |
| Spty2d1 | darkgrey |
| Tmem86a | blue4 |
| Zdhhc13 | blue4 |
| Nav2 | blue3 |
| Htatip2 | blue4 |
| Prmt3 | blue4 |
| Fancf | blue4 |
| Gas2 | blue3 |
| Svip | firebrick2 |
| Gm22211 | saddlebrown |
| Gm5776 | blue4 |
| Tubgcp5 | blue4 |
| Cyfip1 | blue4 |
| Gm17907 | blue4 |
| Nipa2 | green4 |
| A230056P14Rik | blue3 |
| Nipa1 | blue4 |
| Herc2 | blue4 |
| Gabrb3 | blue3 |
| Ube3a | coral1 |
| D7Ertd715e | blue4 |
| Snrpn | blue4 |
| Gm7367 | saddlebrown |
| Gm23962 | mistyrose |
| Mir344d-1 | mistyrose |
| Ndn | blue4 |
| Chrna7 | coral1 |
| Klf13 | darkgrey |
| Gm27252 | blue3 |
| Mtmr10 | mistyrose |
| Gm20457 | blue4 |
| Fan1 | coral1 |
| Mphosph10 | coral1 |
| Mcee | blue4 |
| Ndnl2 | blue4 |
| Tjp1 | blue4 |
| Tarsl2 | blue3 |
| Tm2d3 | blue4 |
| Pcsk6 | blue4 |
| Snrpa1 | blue4 |
| Vimp | green4 |
| Chsy1 | darkgrey |
| Lrrk1 | blue4 |
| Aldh1a3 | coral1 |
| Asb7 | coral1 |
| Lins | blue3 |
| Lysmd4 | darkgrey |
| Mef2a | coral1 |
| Lrrc28 | mistyrose |
| Ttc23 | darkgrey |
| Igf1r | coral1 |
| Pgpep1l | blue4 |
| Gm16157 | darkolivegreen |
| Fam169b | blue3 |
| Arrdc4 | blue4 |
| Nr2f2 | blue4 |
| B130024G19Rik | blue3 |
| Mctp2 | brown1 |
| Rpl17-ps10 | blue4 |
| Rgma | blue4 |
| Chd2 | deeppink1 |
| RP23-32A8.1 | blue4 |
| Fam174b | blue4 |
| Slco3a1 | blue4 |
| Akap13 | darkgrey |
| AU020206 | blue4 |
| Klhl25 | blue4 |
| Ntrk3 | blue3 |
| Mrpl46 | coral1 |
| Mrps11 | deeppink |
| Det1 | blue3 |
| Aen | blue4 |
| Isg20 | blue4 |
| Mfge8 | blue4 |
| Abhd2 | blue4 |
| Polg | blue3 |
| Rhcg | darkolivegreen |
| Plin1 | lightcoral |
| Pex11a | mistyrose |
| Wdr93 | darkolivegreen |
| Anpep | green4 |
| Ap3s2 | blue3 |
| 2610034B18Rik | blue4 |
| Zfp710 | mistyrose |
| Idh2 | blue4 |
| Sema4b | darkgrey |
| Cib1 | deeppink |
| Gdpgp1 | darkgrey |
| Ttll13 | darkolivegreen |
| Ngrn | blue3 |
| Vps33b | blue4 |
| Prc1 | blue4 |
| Rccd1 | blue4 |
| Unc45a | mistyrose |
| Hddc3 | blue3 |
| Man2a2 | blue3 |
| Fes | blue4 |
| Furin | deeppink |
| Blm | blue4 |
| Crtc3 | mistyrose |
| Iqgap1 | blue4 |
| Zscan2 | blue4 |
| Wdr73 | blue4 |
| Nmb | blue3 |
| Sec11a | blue3 |
| Zfp592 | coral1 |
| Slc28a1 | blue4 |
| Pde8a | green4 |
| Rps17 | blue4 |
| Cpeb1 | blue4 |
| 2900076A07Rik | blue3 |
| WI1-1616M8.3 | blue3 |
| Whamm | blue4 |
| Fam103a1 | mistyrose |
| 3110040N11Rik | blue4 |
| Btbd1 | coral1 |
| Tm6sf1 | honeydew |
| Hdgfrp3 | blue4 |
| Eftud1 | blue4 |
| Mex3b | blue4 |
| Stard5 | mistyrose |
| Gm7964 | blue4 |
| Mesdc1 | blue4 |
| Mesdc2 | blue4 |
| Abhd17c | green4 |
| Arnt2 | blue4 |
| Gm2115 | blue4 |
| Fah | blue4 |
| Zfand6 | deeppink |
| Folh1 | deeppink |
| Nox4 | firebrick2 |
| Ctsc | blue4 |
| Rab38 | blue4 |
| Rps13-ps2 | blue4 |
| Tmem135 | blue3 |
| Fzd4 | coral1 |
| Prss23 | blue4 |
| Me3 | blue4 |
| l7Rn6 | blue3 |
| Eed | blue4 |
| 2310010J17Rik | blue4 |
| Picalm | coral1 |
| Sytl2 | blue4 |
| Crebzf | blue3 |
| Tmem126a | blue4 |
| Tmem126b | coral1 |
| Ccdc90b | coral1 |
| Ankrd42 | mistyrose |
| RP23-474B13.1 | blue3 |
| Gm26944 | blue3 |
| RP23-474B13.2 | blue3 |
| Pcf11 | brown1 |
| Rab30 | green4 |
| Prcp | mistyrose |
| Gm15501 | blue4 |
| Nars2 | mistyrose |
| Gab2 | brown1 |
| Kctd21 | blue3 |
| Alg8 | blue4 |
| Ndufc2 | blue4 |
| Thrsp | saddlebrown |
| Ints4 | blue4 |
| Gm24412 | blue3 |
| Aamdc | blue4 |
| Rsf1 | coral1 |
| Clns1a | blue4 |
| Aqp11 | darkolivegreen |
| Pak1 | blue4 |
| Myo7a | blue3 |
| Capn5 | blue4 |
| Acer3 | blue4 |
| Tsku | mistyrose |
| Lrrc32 | blue4 |
| 2210018M11Rik | blue4 |
| Prkrir | green4 |
| Wnt11 | green4 |
| Uvrag | blue4 |
| Dgat2 | blue4 |
| Mogat2 | deeppink |
| Serpinh1 | mistyrose |
| Gdpd5 | blue3 |
| Rps3 | blue4 |
| Arrb1 | blue4 |
| Slco2b1 | blue4 |
| Gm15635 | blue3 |
| Neu3 | blue4 |
| Spcs2 | blue4 |
| Rnf169 | blue3 |
| Pold3 | blue4 |
| Lipt2 | blue4 |
| Pgm2l1 | blue4 |
| Ppme1 | blue4 |
| C2cd3 | blue3 |
| Gm10603 | green4 |
| Ucp3 | blue4 |
| Ucp2 | blue4 |
| Dnajb13 | blue4 |
| Coa4 | blue4 |
| Mrpl48 | orangered |
| Rab6a | blue4 |
| Plekhb1 | blue4 |
| Fam168a | blue3 |
| Gm3200 | firebrick2 |
| Relt | darkgrey |
| Arhgef17 | blue3 |
| P2ry6 | blue4 |
| P2ry2 | darkgrey |
| Fchsd2 | blue3 |
| Atg16l2 | blue3 |
| Stard10 | blue4 |
| Arap1 | blue3 |
| Pde2a | blue4 |
| Gm7027 | blue4 |
| Clpb | blue4 |
| Inppl1 | blue3 |
| Gm10602 | blue3 |
| Folr2 | blue3 |
| Folr1 | blue4 |
| Gm22655 | blue3 |
| Anapc15 | blue3 |
| Lamtor1 | deeppink |
| Lrrc51 | darkolivegreen |
| Numa1 | blue3 |
| Il18bp | saddlebrown |
| Rnf121 | blue4 |
| Xndc1 | blue3 |
| Nup98 | blue4 |
| Pgap2 | blue3 |
| Rhog | blue4 |
| Stim1 | blue3 |
| Rrm1 | blue4 |
| Trim21 | saddlebrown |
| Trim68 | blue3 |
| Hbb-bt | coral1 |
| Hbb-bs | blue4 |
| Dnajc19-ps | blue4 |
| Trim34a | blue4 |
| Trim12a | blue4 |
| Gm15133 | blue4 |
| Trim12c | blue4 |
| Trim30a | blue4 |
| Gm5900 | blue4 |
| Fam160a2 | blue4 |
| Prkcdbp | blue4 |
| Smpd1 | coral1 |
| Apbb1 | green4 |
| Trim3 | blue3 |
| Arfip2 | brown1 |
| Timm10b | blue3 |
| Rrp8 | blue4 |
| Taf10 | blue4 |
| Tpp1 | darkolivegreen |
| Dchs1 | deeppink |
| Mrpl17 | blue4 |
| Gm8995 | blue4 |
| Olfml1 | green4 |
| Ppfibp2 | darkolivegreen |
| Gm10156 | blue4 |
| Eif3f | mistyrose |
| Lmo1 | mistyrose |
| Rpl27a | blue4 |
| Gm24888 | darkgrey |
| St5 | blue4 |
| Akip1 | mistyrose |
| Ascl3 | blue4 |
| Tmem9b | lightsteelblue |
| Dennd5a | darkgrey |
| Tmem41b | darkgrey |
| Ipo7 | blue4 |
| Gm25636 | darkgrey |
| Snora23 | blue4 |
| Zfp143 | blue4 |
| Wee1 | darkgrey |
| Swap70 | darkgrey |
| Sbf2 | blue4 |
| Adm | blue4 |
| Ampd3 | blue4 |
| Rnf141 | coral1 |
| Mrvi1 | blue4 |
| Ctr9 | blue4 |
| Eif4g2 | deeppink |
| Galnt18 | green4 |
| Usp47 | coral1 |
| Dkk3 | green4 |
| Mical2 | blue4 |
| Parva | blue4 |
| 2310014F06Rik | blue3 |
| Tead1 | blue4 |
| Arntl | mistyrose |
| Btbd10 | blue4 |
| Far1 | firebrick2 |
| Far1os | blue3 |
| Spon1 | darkolivegreen |
| Rras2 | blue4 |
| Copb1 | coral1 |
| Psma1 | coral1 |
| Pde3b | blue4 |
| Cyp2r1 | blue4 |
| Calca | darkgrey |
| Gm15500 | blue4 |
| Insc | blue4 |
| Sox6 | lightsteelblue |
| Sox6os | saddlebrown |
| 1110004F10Rik | blue4 |
| Plekha7 | darkolivegreen |
| Rps13 | blue4 |
| Snord14a | darkgrey |
| Pik3c2a | coral1 |
| Nucb2 | blue4 |
| Xylt1 | blue4 |
| Rps15a | blue4 |
| Arl6ip1 | mistyrose |
| Smg1 | blue4 |
| Syt17 | blue3 |
| Itpripl2 | blue4 |
| Coq7 | green4 |
| Gde1 | blue4 |
| Ccp110 | blue3 |
| 9030624J02Rik | blue4 |
| Knop1 | coral1 |
| Gprc5b | blue3 |
| Gp2 | darkolivegreen |
| Umod | blue4 |
| Pdilt | coral1 |
| Acsm5 | blue4 |
| Acsm2 | blue4 |
| Acsm1 | darkolivegreen |
| Thumpd1 | coral1 |
| Gm5601 | blue4 |
| Acsm3 | mistyrose |
| Eri2 | mistyrose |
| 2610020H08Rik | blue3 |
| Dcun1d3 | blue3 |
| Lyrm1 | deeppink |
| Tmem159 | blue4 |
| Anks4b | blue4 |
| Crym | blue3 |
| Uqcrc2 | deeppink |
| 9030407P20Rik | blue3 |
| BC030336 | blue4 |
| Eef2k | blue4 |
| Polr3e | blue3 |
| Cdr2 | blue3 |
| 4933427G17Rik | blue4 |
| Mettl9 | mistyrose |
| Igsf6 | blue4 |
| Usp31 | blue4 |
| Scnn1g | mistyrose |
| Scnn1b | green4 |
| Cog7 | darkgrey |
| Gga2 | blue3 |
| Ears2 | green4 |
| Ubfd1 | blue3 |
| Ndufab1 | blue4 |
| Palb2 | blue4 |
| Dctn5 | blue3 |
| Plk1 | blue4 |
| Gm14388 | blue4 |
| Rbbp6 | blue4 |
| Tnrc6a | blue3 |
| Slc5a11 | coral1 |
| Arhgap17 | blue4 |
| Lcmt1 | blue4 |
| Aqp8 | deeppink |
| Kdm8 | blue4 |
| Nsmce1 | blue4 |
| Il4ra | darkgrey |
| Gtf3c1 | green4 |
| D430042O09Rik | blue3 |
| Xpo6 | blue4 |
| Sbk1 | darkgrey |
| Lat | blue4 |
| Spns1 | green4 |
| Nfatc2ip | blue4 |
| Rabep2 | blue3 |
| Sh2b1 | green4 |
| Tufm | green4 |
| Atxn2l | mistyrose |
| Eif3c | blue4 |
| Gm25579 | blue4 |
| Cln3 | green4 |
| Nupr1 | blue4 |
| Ccdc101 | blue4 |
| Sult1a1 | mistyrose |
| Slx1b | blue3 |
| Bola2 | blue4 |
| Coro1a | honeydew |
| Mapk3 | blue4 |
| Gdpd3 | blue4 |
| Ypel3 | blue4 |
| Ppp4c | blue4 |
| Aldoa | deeppink |
| Gm15676 | blue4 |
| Ino80e | blue4 |
| Hirip3 | blue3 |
| Taok2 | blue3 |
| Tmem219 | coral1 |
| Kctd13 | darkgrey |
| Cdipt | deeppink |
| Mvp | blue4 |
| Pagr1a | green4 |
| Maz | blue3 |
| Kif22 | blue4 |
| AI467606 | blue4 |
| Qprt | blue4 |
| Spn | saddlebrown |
| Cd2bp2 | blue3 |
| Tbc1d10b | blue3 |
| 1-Sep | blue4 |
| Zfp553 | blue4 |
| Zfp771 | green4 |
| Dctpp1 | blue4 |
| Sephs2 | mistyrose |
| Itgal | blue4 |
| Zfp768 | blue3 |
| Zfp747 | blue4 |
| E430018J23Rik | darkgrey |
| Zfp764 | blue4 |
| Zfp688 | blue3 |
| Prr14 | deeppink |
| Fbrs | mistyrose |
| 1700008J07Rik | blue3 |
| Srcap | blue4 |
| Snora30 | blue3 |
| Phkg2 | green4 |
| Gm166 | darkolivegreen |
| Rnf40 | mistyrose |
| 1700120K04Rik | blue3 |
| Zfp629 | blue3 |
| Bcl7c | blue4 |
| Ctf1 | blue3 |
| Fbxl19 | blue3 |
| Orai3 | brown1 |
| Setd1a | blue4 |
| Hsd3b7 | blue4 |
| Stx4a | blue3 |
| Zfp668 | blue4 |
| Zfp646 | blue4 |
| Vkorc1 | coral1 |
| Bckdk | blue4 |
| Kat8 | blue4 |
| Prss8 | blue4 |
| Prss36 | green4 |
| Fus | blue4 |
| Pycard | blue4 |
| Itgax | honeydew |
| Cox6a2 | blue4 |
| 9130023H24Rik | blue3 |
| Armc5 | coral1 |
| Tgfb1i1 | blue4 |
| Slc5a2 | blue4 |
| BC017158 | blue3 |
| Rgs10 | saddlebrown |
| Tial1 | blue3 |
| Bag3 | darkgrey |
| Inpp5f | blue3 |
| Mcmbp | blue4 |
| Sec23ip | blue4 |
| Wdr11 | blue3 |
| Fgfr2 | deeppink1 |
| Ate1 | blue4 |
| Nsmce4a | mistyrose |
| Tacc2 | blue4 |
| Plekha1 | blue4 |
| Htra1 | mistyrose |
| 2310057M21Rik | blue4 |
| Pstk | green4 |
| Acadsb | mistyrose |
| Hmx2 | darkgrey |
| Bub3 | blue4 |
| Chst15 | blue3 |
| Oat | blue3 |
| Lhpp | blue4 |
| Fam53b | green4 |
| Mettl10 | blue4 |
| Fam175b | blue4 |
| Zranb1 | brown1 |
| Gm15718 | darkgrey |
| Ctbp2 | blue4 |
| Edrf1 | blue4 |
| Uros | blue4 |
| Bccip | coral1 |
| Dhx32 | blue4 |
| Adam12 | blue4 |
| Dock1 | blue4 |
| Clrn3 | darkgrey |
| Mki67 | blue4 |
| Mgmt | blue3 |
| Glrx3 | blue4 |
| Mapk1ip1 | blue3 |
| Ppp2r2d | darkgrey |
| Bnip3 | mistyrose |
| Stk32c | mistyrose |
| Pwwp2b | blue4 |
| Inpp5a | darkolivegreen |
| Gm4459 | blue4 |
| Adam8 | blue4 |
| Tubgcp2 | blue4 |
| Zfp511 | blue4 |
| Fuom | blue4 |
| Echs1 | blue4 |
| Paox | darkgrey |
| Mtg1 | blue3 |
| Cyp2e1 | blue4 |
| Zfp941 | darkgrey |
| Urah | blue3 |
| Scgb1c1 | blue4 |
| Bet1l | blue3 |
| Ric8 | blue4 |
| Sirt3 | blue4 |
| Psmd13 | blue4 |
| Gm15542 | darkgrey |
| Cox8b | lightcoral |
| Nlrp6 | darkolivegreen |
| Athl1 | green4 |
| Ifitm2 | blue4 |
| Ifitm1 | blue4 |
| Ifitm3 | blue4 |
| B4galnt4 | mistyrose |
| Pkp3 | darkgrey |
| Sigirr | green4 |
| Ano9 | blue4 |
| Ptdss2 | green4 |
| Rnh1 | blue4 |
| Hras | deeppink |
| Lrrc56 | darkolivegreen |
| Lmntd2 | blue3 |
| Rassf7 | green4 |
| Phrf1 | blue4 |
| Irf7 | saddlebrown |
| Cdhr5 | green4 |
| Deaf1 | green4 |
| Tmem80 | blue3 |
| Eps8l2 | mistyrose |
| B230206H07Rik | darkgrey |
| Taldo1 | blue4 |
| Pddc1 | darkgrey |
| Slc25a22 | mistyrose |
| Pidd1 | blue3 |
| Rplp2 | blue4 |
| Pnpla2 | saddlebrown |
| Cracr2b | blue4 |
| Cd151 | blue4 |
| Polr2l | blue4 |
| Tspan4 | blue4 |
| Chid1 | green4 |
| Ap2a2 | green4 |
| Tollip | coral1 |
| Mob2 | blue4 |
| Dusp8 | darkgrey |
| Ctsd | blue4 |
| Syt8 | blue4 |
| Lsp1 | blue4 |
| Mrpl23 | blue4 |
| Cd81 | coral1 |
| Tssc4 | deeppink |
| Kcnq1 | green4 |
| Cdkn1c | deeppink |
| Slc22a18 | blue4 |
| Phlda2 | blue4 |
| Nap1l4 | blue4 |
| Gm23297 | deeppink |
| Cars | blue4 |
| Tnfrsf26 | brown1 |
| Osbpl5 | blue4 |
| Nadsyn1 | green4 |
| Dhcr7 | green4 |
| Shank2 | green4 |
| Cttn | blue4 |
| Ppfia1 | blue4 |
| Fadd | blue3 |
| Ano1 | blue4 |
| Oraov1 | blue4 |
| Ccnd1 | blue3 |
| Tpcn2 | blue4 |
| Mrgprf | blue4 |
| Insr | firebrick2 |
| Arhgef18 | blue3 |
| Pex11g | blue4 |
| Zfp358 | brown1 |
| Mcoln1 | green4 |
| Pnpla6 | green4 |
| C330021F23Rik | darkolivegreen |
| Camsap3 | mistyrose |
| Xab2 | blue4 |
| Gm22204 | coral1 |
| Pet100 | blue4 |
| Stxbp2 | blue3 |
| Fcor | lightcoral |
| Trappc5 | blue4 |
| Cd209a | blue4 |
| Evi5l | blue3 |
| Map2k7 | blue3 |
| Tgfbr3l | darkolivegreen |
| Snapc2 | blue4 |
| Ctxn1 | blue4 |
| Timm44 | coral1 |
| Elavl1 | blue4 |
| Ccl25 | blue4 |
| Cers4 | blue4 |
| Zfp958 | coral1 |
| Shcbp1 | blue4 |
| Slc10a2 | darkolivegreen |
| Gm1840 | blue4 |
| Efnb2 | blue3 |
| Arglu1 | blue3 |
| Lig4 | coral1 |
| Abhd13 | green4 |
| Tnfsf13b | blue4 |
| Irs2 | blue4 |
| Gm15418 | darkgrey |
| Col4a1 | blue4 |
| Col4a2 | blue4 |
| Rab20 | blue3 |
| E230013L22Rik | blue4 |
| Carkd | blue4 |
| Cars2 | coral1 |
| Ing1 | coral1 |
| Ankrd10 | blue4 |
| Arhgef7 | mistyrose |
| Tubgcp3 | blue3 |
| Gm15348 | blue4 |
| Atp11a | blue4 |
| Gm15347 | blue3 |
| Mcf2l | blue3 |
| Proz | blue4 |
| Pcid2 | blue3 |
| Gm17022 | blue3 |
| Cul4a | coral1 |
| Lamp1 | blue4 |
| Grtp1 | blue4 |
| Mir1968 | blue4 |
| 2810030D12Rik | darkolivegreen |
| Dcun1d2 | blue3 |
| Gm25014 | blue3 |
| Tmco3 | blue4 |
| Tfdp1 | blue4 |
| Gas6 | mistyrose |
| Rasa3 | blue4 |
| Cdc16 | coral1 |
| Upf3a | blue4 |
| Champ1 | blue4 |
| Gm24698 | coral1 |
| Coprs | blue4 |
| Fbxo25 | blue3 |
| Tdrp | blue4 |
| Erich1 | coral1 |
| Cln8 | coral1 |
| Arhgef10 | blue4 |
| Kbtbd11 | blue4 |
| Myom2 | blue3 |
| Mcph1 | blue4 |
| Angpt2 | blue3 |
| Agpat5 | blue4 |
| Rpl19-ps11 | blue4 |
| Gm6483 | blue4 |
| Gm21811 | blue4 |
| 6820431F20Rik | deeppink1 |
| 2610005L07Rik | deeppink1 |
| Gm21092 | deeppink1 |
| Defb1 | blue3 |
| Defb2 | blue4 |
| Defb11 | blue4 |
| Alg11 | coral1 |
| Nek3 | blue4 |
| Ckap2 | blue4 |
| Vps36 | blue4 |
| Slc25a15 | lightsteelblue |
| 1810012K16Rik | blue3 |
| Mrps31 | mistyrose |
| Smim19 | blue4 |
| Slc20a2 | blue4 |
| Gm17491 | blue3 |
| Vdac3 | blue4 |
| Polb | blue4 |
| Ikbkb | blue3 |
| Plat | darkgrey |
| Ap3m2 | blue3 |
| Kat6a | blue4 |
| Agpat6 | deeppink |
| Gins4 | blue4 |
| Golga7 | blue4 |
| Sfrp1 | darkolivegreen |
| 1810011O10Rik | darkolivegreen |
| Ido2 | darkolivegreen |
| Adam9 | blue4 |
| Tm2d2 | blue4 |
| Plekha2 | blue4 |
| Tacc1 | blue4 |
| Fgfr1 | mistyrose |
| Letm2 | darkolivegreen |
| Whsc1l1 | blue4 |
| Ppapdc1b | blue4 |
| Ddhd2 | coral1 |
| Bag4 | mistyrose |
| Lsm1 | blue4 |
| Star | blue4 |
| Ash2l | blue4 |
| Hgsnat | blue4 |
| Pomk | blue3 |
| Fnta | blue4 |
| Hook3 | blue4 |
| Rnf170 | blue4 |
| Thap1 | blue4 |
| Zfp703 | darkgrey |
| Erlin2 | blue3 |
| Proscos | blue4 |
| Prosc | mistyrose |
| Gpr124 | blue3 |
| Brf2 | blue3 |
| Rab11fip1 | mistyrose |
| Eif4ebp1 | blue4 |
| Rnf122 | mistyrose |
| Tti2 | blue4 |
| Mak16 | blue4 |
| Fut10 | blue4 |
| Nrg1 | blue4 |
| Gm26578 | blue4 |
| Wrn | firebrick2 |
| Purg | blue3 |
| Ppp2cb | blue4 |
| Ubxn8 | green4 |
| Gsr | blue4 |
| Gm24727 | blue4 |
| Gtf2e2 | blue4 |
| Rbpms | blue4 |
| Gm9951 | blue3 |
| Dctn6 | blue4 |
| Gm10131 | blue4 |
| Leprotl1 | blue4 |
| Saraf | blue4 |
| Mir6395 | blue3 |
| Dusp4 | darkgrey |
| Tnks | indianred2 |
| Eri1 | blue4 |
| Mfhas1 | blue4 |
| Cldn23 | darkgrey |
| D8Ertd82e | darkgrey |
| Lonrf1 | darkgrey |
| Dlc1 | mistyrose |
| AI429214 | coral1 |
| Tusc3 | mistyrose |
| Micu3 | coral1 |
| Zdhhc2 | coral1 |
| Cnot7 | blue4 |
| Vps37a | coral1 |
| Mtmr7 | coral1 |
| Mtus1 | lightsteelblue |
| Fgl1 | lightcoral |
| Pcm1 | blue4 |
| Asah1 | coral1 |
| Frg1 | blue4 |
| Gm6180 | blue4 |
| Fat1 | blue4 |
| Cyp4v3 | blue4 |
| Fam149a | blue3 |
| Tlr3 | blue4 |
| Sorbs2 | blue4 |
| Pdlim3 | blue4 |
| 1700029J07Rik | darkolivegreen |
| Ufsp2 | coral1 |
| Ankrd37 | blue4 |
| Snx25 | blue4 |
| Cfap97 | coral1 |
| Gm15634 | blue4 |
| Slc25a4 | coral1 |
| Acsl1 | blue3 |
| Primpol | blue3 |
| Casp3 | blue4 |
| Irf2 | blue3 |
| Enpp6 | mistyrose |
| Stox2 | blue4 |
| Trappc11 | coral1 |
| Rwdd4a | blue4 |
| Ing2 | firebrick2 |
| Cdkn2aip | blue3 |
| Wwc2 | blue3 |
| Dctd | blue4 |
| Gm9892 | blue4 |
| Aga | darkolivegreen |
| Vegfc | blue4 |
| Spcs3 | blue4 |
| Gpm6a | blue4 |
| Hpgd | mistyrose |
| Cep44 | mistyrose |
| Fbxo8 | mistyrose |
| Gm25992 | darkolivegreen |
| Sap30 | blue4 |
| 2500002B13Rik | darkolivegreen |
| Hmgb2 | blue4 |
| Galnt7 | blue4 |
| Aadat | mistyrose |
| Mfap3l | firebrick2 |
| 2700029M09Rik | blue4 |
| Clcn3 | deeppink |
| Nek1 | blue4 |
| Sh3rf1 | darkgrey |
| 1700001D01Rik | antiquewhite2 |
| Cbr4 | mistyrose |
| Palld | darkgrey |
| Tll1 | blue4 |
| Cpe | blue4 |
| Msmo1 | blue4 |
| Klhl2 | blue4 |
| Tmem192 | blue4 |
| Trim61 | darkolivegreen |
| 1-Mar | coral1 |
| Tma16 | blue4 |
| Npy1r | darkolivegreen |
| Naf1 | darkgrey |
| Nat2 | mistyrose |
| Gm9755 | blue4 |
| Psd3 | blue4 |
| Sh2d4a | blue3 |
| Csgalnact1 | deeppink |
| Ints10 | blue4 |
| Lpl | blue4 |
| Slc18a1 | blue3 |
| Atp6v1b2 | darkolivegreen |
| Gm15717 | blue4 |
| Zfp930 | coral1 |
| Gm10033 | blue4 |
| Gm10311 | coral1 |
| Zfp868 | darkgrey |
| Zfp964 | blue3 |
| Zfp869 | darkgrey |
| Zfp866 | coral1 |
| Atp13a1 | deeppink |
| Gmip | blue4 |
| Lpar2 | blue4 |
| Yjefn3 | green4 |
| Ndufa13 | blue4 |
| Tssk6 | blue4 |
| Gatad2a | darkgrey |
| Mau2 | blue3 |
| Sugp1 | green4 |
| Tm6sf2 | blue3 |
| Rfxank | green4 |
| Nr2c2ap | deeppink |
| 2310045N01Rik | blue3 |
| Tmem161a | deeppink |
| Slc25a42 | blue4 |
| Armc6 | blue4 |
| Sugp2 | blue3 |
| Homer3 | blue4 |
| Ddx49 | firebrick2 |
| Cope | blue3 |
| Upf1 | blue3 |
| Crtc1 | blue3 |
| Klhl26 | blue4 |
| Crlf1 | blue4 |
| 2810428I15Rik | deeppink |
| Uba52 | blue4 |
| Kxd1 | darkolivegreen |
| Fkbp8 | deeppink |
| Ell | darkgrey |
| Isyna1 | blue4 |
| Ssbp4 | blue4 |
| Lrrc25 | honeydew |
| Gdf15 | mistyrose |
| Pgpep1 | blue4 |
| Lsm4 | firebrick2 |
| Jund | darkgrey |
| Gm3336 | blue3 |
| Pde4c | darkolivegreen |
| Rab3a | green4 |
| Mpv17l2 | deeppink |
| Ifi30 | blue3 |
| Pik3r2 | green4 |
| 2010320M18Rik | blue3 |
| Mast3 | blue4 |
| Mir7240 | darkgrey |
| Arrdc2 | mistyrose |
| Kcnn1 | green4 |
| Ccdc124 | blue4 |
| Rpl18a | blue4 |
| Snora68 | blue4 |
| Map1s | blue4 |
| Haus8 | blue4 |
| Myo9b | blue4 |
| Use1 | firebrick2 |
| Ocel1 | blue3 |
| Nr2f6 | deeppink |
| Ushbp1 | green4 |
| Babam1 | coral1 |
| Abhd8 | deeppink |
| Mrpl34 | blue3 |
| Dda1 | mistyrose |
| Ano8 | green4 |
| Gtpbp3 | blue3 |
| Plvap | mistyrose |
| Bst2 | saddlebrown |
| Mvb12a | deeppink |
| Tmem221 | green4 |
| Slc27a1 | green4 |
| Pgls | blue4 |
| Fam129c | blue3 |
| Colgalt1 | blue4 |
| Jak3 | blue4 |
| Insl3 | blue3 |
| B3gnt3 | blue4 |
| Zfp709 | coral1 |
| Zfp882 | coral1 |
| Zfp617 | coral1 |
| Ccnb2-ps | blue3 |
| Zfp961 | coral1 |
| Cyp4f18 | blue4 |
| Tpm4 | blue4 |
| Gm16091 | blue4 |
| Rab8a | darkgrey |
| Fam32a | mistyrose |
| Ap1m1 | blue4 |
| Gm10282 | blue4 |
| Klf2 | coral1 |
| Eps15l1 | blue3 |
| 1700030K09Rik | blue3 |
| Cherp | blue3 |
| Slc35e1 | green4 |
| Med26 | darkgrey |
| Smim7 | blue3 |
| Tmem38a | coral1 |
| Sin3b | blue4 |
| Large | blue4 |
| Hmgxb4 | blue4 |
| Tom1 | green4 |
| Hmox1 | darkolivegreen |
| Mcm5 | blue4 |
| Rasd2 | blue4 |
| Gm7984 | blue4 |
| Nr3c2 | blue3 |
| Arhgap10 | orangered |
| 0610038B21Rik | darkolivegreen |
| Prmt10 | coral1 |
| Gm23468 | blue3 |
| Tmem184c | blue4 |
| Rbmxl1 | blue4 |
| Slc10a7 | blue4 |
| Lsm6 | blue4 |
| Zfp827 | blue3 |
| Mmaa | darkolivegreen |
| Smad1 | darkgrey |
| Otud4 | coral1 |
| Abce1 | blue4 |
| Anapc10 | blue4 |
| Smarca5 | blue4 |
| Gab1 | blue4 |
| Usp38 | brown1 |
| Il15 | blue4 |
| Zfp330 | blue4 |
| Tbc1d9 | darkgrey |
| Ucp1 | blue4 |
| Elmod2 | blue4 |
| Scoc | mistyrose |
| Ndufb7 | indianred2 |
| Tecr | green4 |
| Dnajb1 | darkgrey |
| Gipc1 | blue4 |
| Ptger1 | green4 |
| Pkn1 | deeppink |
| Ddx39 | blue4 |
| Cd97 | blue4 |
| Lphn1 | green4 |
| Asf1b | blue4 |
| Prkaca | blue3 |
| Samd1 | blue3 |
| 1700067K01Rik | blue4 |
| 2210011C24Rik | blue4 |
| Palm3 | blue4 |
| Il27ra | blue4 |
| Rfx1 | mistyrose |
| Mir709 | blue3 |
| Dcaf15 | blue4 |
| Cc2d1a | blue3 |
| Mir23a | coral1 |
| Gm26532 | mistyrose |
| Zswim4 | darkgrey |
| D8Ertd738e | firebrick2 |
| Mri1 | blue4 |
| Ccdc130 | blue3 |
| Ier2 | coral1 |
| Gm26664 | blue3 |
| Nacc1 | blue4 |
| Trmt1 | blue4 |
| Nfix | green4 |
| Lyl1 | blue4 |
| Dand5 | blue3 |
| Gadd45gip1 | blue4 |
| Rad23a | green4 |
| Calr | mistyrose |
| Farsa | blue4 |
| Syce2 | mistyrose |
| Gcdh | blue4 |
| Klf1 | darkgrey |
| Dnase2a | darkgrey |
| Rnaseh2a | blue4 |
| Prdx2 | blue4 |
| Junb | coral1 |
| Hook2 | darkolivegreen |
| Asna1 | blue4 |
| 2310036O22Rik | deeppink |
| Tnpo2 | blue4 |
| Fbxw9 | blue4 |
| Gm5741 | blue3 |
| Dhps | blue4 |
| Wdr83 | blue3 |
| Wdr83os | blue4 |
| Man2b1 | green4 |
| Zfp791 | mistyrose |
| Vps35 | coral1 |
| Orc6 | blue4 |
| Mylk3 | blue4 |
| 4921524J17Rik | deeppink |
| Gpt2 | blue4 |
| Dnaja2 | blue4 |
| Itfg1 | mistyrose |
| Phkb | firebrick2 |
| Lonp2 | deeppink |
| Siah1a | blue3 |
| Gm10638 | blue4 |
| N4bp1 | blue3 |
| Zfp423 | deeppink |
| Cnep1r1 | coral1 |
| Heatr3 | blue4 |
| Papd5 | coral1 |
| Adcy7 | blue4 |
| Brd7 | blue4 |
| Nkd1 | blue4 |
| Nod2 | coral1 |
| Cyld | blue4 |
| Sall1 | blue3 |
| Tox3 | darkgrey |
| Chd9 | blue4 |
| Gm6658 | blue3 |
| Rbl2 | mistyrose |
| Aktip | blue3 |
| Rpgrip1l | firebrick2 |
| Fto | deeppink |
| Irx3 | coral1 |
| Irx3os | blue3 |
| Crnde | blue4 |
| Mmp2 | deeppink |
| Ces1d | blue4 |
| Ces1e | blue4 |
| Ces1f | blue4 |
| Ces1g | blue4 |
| Gnao1 | blue4 |
| Amfr | blue4 |
| Nudt21 | blue4 |
| Ogfod1 | blue4 |
| Bbs2 | darkolivegreen |
| Mt3 | blue4 |
| Mt2 | darkgrey |
| Mt1 | darkgrey |
| Nup93 | blue4 |
| Slc12a3 | green4 |
| Gm15889 | blue4 |
| Gm15890 | blue4 |
| Herpud1 | blue3 |
| Cpne2 | blue4 |
| Fam192a | blue4 |
| Rspry1 | coral1 |
| Arl2bp | blue4 |
| Pllp | mistyrose |
| Cx3cl1 | blue4 |
| Ccl17 | blue3 |
| Ciapin1 | darkolivegreen |
| Coq9 | blue4 |
| Polr2c | deeppink |
| Dok4 | blue4 |
| Ccdc102a | blue4 |
| Gpr56 | mistyrose |
| Gpr97 | blue4 |
| Katnb1 | coral1 |
| Kifc3 | blue3 |
| Tepp | blue3 |
| Zfp319 | blue3 |
| Usb1 | blue4 |
| Mmp15 | deeppink |
| Cfap20 | blue4 |
| Csnk2a2 | blue4 |
| Gins3 | blue4 |
| Ndrg4 | green4 |
| Setd6 | blue4 |
| Cnot1 | blue4 |
| Gm26493 | deeppink1 |
| Gm10094 | blue4 |
| Slc38a7 | brown1 |
| Got2 | deeppink |
| Cdh11 | blue4 |
| Gm8730 | blue4 |
| Cdh5 | darkgrey |
| Tk2 | blue4 |
| Cklf | blue4 |
| Cmtm3 | blue4 |
| Cmtm4 | blue4 |
| Dync1li2 | blue4 |
| Nae1 | coral1 |
| Car7 | green4 |
| Pdp2 | darkgrey |
| Cdh16 | coral1 |
| Rrad | mistyrose |
| Fam96b | blue4 |
| Ces2b | blue4 |
| Ces2c | blue4 |
| Ces2e | blue3 |
| Ces2g | coral1 |
| Ces2h | blue4 |
| Cbfb | blue4 |
| D230025D16Rik | blue4 |
| Tradd | blue3 |
| Fbxl8 | blue3 |
| Hsf4 | darkolivegreen |
| Nol3 | blue3 |
| 4931428F04Rik | blue4 |
| Exoc3l | blue3 |
| E2f4 | blue4 |
| Elmo3 | green4 |
| Lrrc29 | blue4 |
| Tmem208 | lightsteelblue |
| Fhod1 | blue3 |
| Tppp3 | darkgrey |
| Zdhhc1 | green4 |
| Hsd11b2 | green4 |
| Atp6v0d1 | coral1 |
| RP23-410B24.2 | blue3 |
| Agrp | blue3 |
| Fam65a | mistyrose |
| Ctcf | blue4 |
| Gm5915 | blue4 |
| Acd | deeppink |
| Pard6a | brown1 |
| Enkd1 | blue3 |
| Gfod2 | blue4 |
| Ranbp10 | blue3 |
| Cenpt | blue4 |
| Thap11 | darkgrey |
| Nutf2 | blue4 |
| Edc4 | mistyrose |
| Pskh1 | blue4 |
| Psmb10 | saddlebrown |
| Lcat | blue4 |
| Slc12a4 | blue4 |
| Dus2 | blue4 |
| Ddx28 | darkolivegreen |
| Nfatc3 | blue4 |
| Esrp2 | blue3 |
| 1810019D21Rik | darkolivegreen |
| Pla2g15 | blue3 |
| Slc7a6 | blue4 |
| Slc7a6os | darkgrey |
| Prmt7 | blue4 |
| Cdh3 | blue4 |
| Gm10073 | blue4 |
| Cdh1 | darkgrey |
| Tango6 | green4 |
| Chtf8 | deeppink |
| Cirh1a | blue4 |
| Sntb2 | blue4 |
| Vps4a | blue4 |
| Gm16209 | blue3 |
| Cog8 | deeppink |
| Nip7 | blue4 |
| Tmed6 | blue4 |
| Terf2 | lightsteelblue |
| Cyb5b | darkolivegreen |
| Rps18-ps3 | blue4 |
| Nfat5 | blue4 |
| Nqo1 | blue4 |
| Nob1 | blue4 |
| Wwp2 | blue4 |
| Rps26-ps1 | blue4 |
| Psmd7 | blue4 |
| Zfhx3 | blue3 |
| Dhx38 | darkgrey |
| Txnl4b | darkgrey |
| Hp | lightcoral |
| Dhodh | blue3 |
| Ist1 | blue4 |
| Zfp821 | mistyrose |
| Atxn1l | lightsteelblue |
| Ap1g1 | firebrick2 |
| Snord71 | darkgrey |
| Gm25321 | deeppink1 |
| Phlpp2 | blue3 |
| Marveld3 | blue3 |
| Zfp612 | darkgrey |
| Gm17720 | darkolivegreen |
| Cmtr2 | blue4 |
| Vac14 | blue3 |
| Mtss1l | blue3 |
| Il34 | blue4 |
| Gm15895 | blue4 |
| Sf3b3 | blue4 |
| Cog4 | coral1 |
| Fuk | green4 |
| St3gal2 | blue4 |
| Ddx19a | blue4 |
| Ddx19b | blue4 |
| Aars | blue4 |
| Pdpr | mistyrose |
| 9430091E24Rik | mistyrose |
| Glg1 | firebrick2 |
| Rfwd3 | darkgrey |
| Mlkl | blue4 |
| Fa2h | green4 |
| Wdr59 | blue4 |
| Znrf1 | blue4 |
| Ldhd | blue4 |
| Zfp1 | darkgrey |
| Bcar1 | darkgrey |
| Cfdp1 | blue4 |
| Tmem170 | blue4 |
| Tmem231 | blue3 |
| Gabarapl2 | green4 |
| Adat1 | blue3 |
| Kars | blue4 |
| Terf2ip | coral1 |
| Gm6793 | blue4 |
| Mon1b | blue3 |
| Nudt7 | orangered |
| Wwox | blue4 |
| Maf | mistyrose |
| Cdyl2 | blue4 |
| Cmc2 | green4 |
| Cenpn | blue4 |
| Atmin | blue4 |
| 1700030J22Rik | darkgrey |
| Gcsh | blue4 |
| Bco1 | blue4 |
| Gan | blue4 |
| Gm20204 | blue3 |
| Cmip | mistyrose |
| Plcg2 | blue3 |
| Sdr42e1 | darkgrey |
| Hsd17b2 | mistyrose |
| Mphosph6 | blue4 |
| Cdh13 | coral1 |
| Hsbp1 | blue4 |
| Mlycd | blue4 |
| Osgin1 | darkgrey |
| Mbtps1 | blue3 |
| Hsdl1 | blue4 |
| Taf1c | blue3 |
| Rps13-ps4 | blue3 |
| Wfdc1 | green4 |
| Tldc1 | darkgrey |
| Cotl1 | mistyrose |
| Klhl36 | green4 |
| Usp10 | green4 |
| Crispld2 | mistyrose |
| Zdhhc7 | deeppink |
| A130014A01Rik | blue3 |
| 6430548M08Rik | blue3 |
| Gse1 | blue4 |
| Gins2 | blue4 |
| Emc8 | blue4 |
| Cox4i1 | indianred2 |
| Irf8 | honeydew |
| Foxf1 | darkgrey |
| Gm26572 | blue3 |
| Mthfsd | blue4 |
| Foxc2 | deeppink |
| Fbxo31 | darkgrey |
| Map1lc3b | blue4 |
| Zcchc14 | firebrick2 |
| Gm27030 | blue4 |
| Klhdc4 | blue4 |
| Slc7a5 | blue4 |
| Car5a | darkolivegreen |
| Banp | deeppink |
| Gm17709 | darkolivegreen |
| Mir7237 | blue3 |
| Zfpm1 | blue4 |
| Gm20681 | blue3 |
| Zc3h18 | blue4 |
| Il17c | antiquewhite2 |
| Cyba | indianred2 |
| Mvd | green4 |
| AL591003.1 | blue3 |
| Snai3 | blue4 |
| Rnf166 | blue4 |
| Gm24366 | blue4 |
| Ctu2 | blue4 |
| Piezo1 | blue4 |
| Cdt1 | blue4 |
| Aprt | blue4 |
| Galns | blue4 |
| Trappc2l | blue4 |
| Cbfa2t3 | deeppink |
| Acsf3 | blue4 |
| Gm16378 | deeppink |
| Ankrd11 | blue4 |
| 2810013P06Rik | mistyrose |
| Spg7 | blue4 |
| Rpl13 | blue4 |
| Cpne7 | blue3 |
| Sult5a1 | blue4 |
| Dpep1 | blue3 |
| Chmp1a | deeppink |
| Rps12-ps9 | darkgrey |
| Spata33 | darkolivegreen |
| Cdk10 | blue3 |
| Spata2l | coral1 |
| Vps9d1 | green4 |
| Zfp276 | blue3 |
| Fanca | blue4 |
| Spire2 | blue4 |
| Tcf25 | mistyrose |
| Def8 | deeppink1 |
| Afg3l1 | darkgrey |
| Gas8 | blue4 |
| Gm22314 | blue4 |
| Rhou | blue4 |
| Rab4a | green4 |
| Nup133 | blue4 |
| Abcb10 | blue3 |
| Taf5l | darkgrey |
| Cog2 | blue4 |
| Agt | darkolivegreen |
| 2310022B05Rik | mistyrose |
| Ttc13 | blue3 |
| Arv1 | blue4 |
| Fam89a | blue3 |
| 2810004N23Rik | blue4 |
| Gnpat | blue3 |
| Exoc8 | darkgrey |
| Sprtn | blue4 |
| Egln1 | blue4 |
| Tsnax | blue4 |
| Sipa1l2 | darkgrey |
| Map10 | lightsteelblue |
| Ntpcr | blue3 |
| BC021891 | blue4 |
| Kcnk1 | blue4 |
| Slc35f3 | coral1 |
| Coa6 | mistyrose |
| Gm17296 | blue4 |
| Irf2bp2 | darkgrey |
| Tomm20 | blue3 |
| Gm26397 | darkgrey |
| Rbm34 | brown1 |
| Pard3 | orangered |
| Nrp1 | blue4 |
| Itgb1 | blue4 |
| 2610044O15Rik8 | darkgrey |
| Gm21399 | blue4 |
| Alkbh8 | coral1 |
| Cwf19l2 | coral1 |
| Gucy1a2 | blue4 |
| Aasdhppt | coral1 |
| Kbtbd3 | firebrick2 |
| Msantd4 | blue4 |
| Casp1 | blue4 |
| Casp4 | darkgrey |
| Casp12 | blue4 |
| Pdgfd | coral1 |
| Dync2h1 | firebrick2 |
| Dcun1d5 | blue4 |
| Mmp8 | darkgrey |
| Gm10709 | blue4 |
| Tmem123 | blue4 |
| Birc2 | blue4 |
| Birc3 | darkgrey |
| Yap1 | blue4 |
| 9230110C19Rik | blue3 |
| AK129341 | firebrick2 |
| Arhgap42 | lightsteelblue |
| Jrkl | blue4 |
| Ccdc82 | blue4 |
| Maml2 | blue4 |
| Mtmr2 | green4 |
| Cep57 | brown1 |
| Fam76b | blue4 |
| Sesn3 | blue4 |
| Endod1 | blue4 |
| Cwc15 | blue4 |
| Amotl1 | blue4 |
| Ankrd49 | blue4 |
| Mre11a | blue4 |
| Vstm5 | blue3 |
| Med17 | coral1 |
| 4931406C07Rik | blue3 |
| Taf1d | blue4 |
| Gm25500 | blue4 |
| Gm24455 | blue4 |
| Gm24299 | blue4 |
| Gm25791 | blue4 |
| Gm22620 | blue4 |
| Gm24357 | blue4 |
| Gm23455 | blue4 |
| Gm22579 | blue4 |
| Cep295 | blue4 |
| Smco4 | green4 |
| Slc36a4 | blue4 |
| Chordc1 | blue4 |
| Zfp317 | blue4 |
| Gm7808 | lightsteelblue |
| Zfp560 | darkgrey |
| Zfp26 | blue4 |
| Zfp426 | blue4 |
| Zfp266 | blue4 |
| Zfp846 | blue3 |
| Fbxl12 | antiquewhite2 |
| Ubl5 | blue4 |
| Pin1 | deeppink |
| Col5a3 | blue4 |
| A230050P20Rik | blue4 |
| Angptl6 | blue3 |
| Ppan | blue4 |
| Eif3g | blue4 |
| Dnmt1 | blue4 |
| S1pr2 | blue4 |
| Mrpl4 | green4 |
| Icam1 | darkgrey |
| Fdx1l | blue4 |
| Raver1 | blue3 |
| Tyk2 | blue3 |
| Cdc37 | blue4 |
| Pde4a | blue3 |
| Keap1 | blue3 |
| Atg4d | brown1 |
| Kri1 | blue3 |
| Cdkn2d | blue4 |
| Ap1m2 | green4 |
| Slc44a2 | blue4 |
| Ilf3 | blue4 |
| Qtrt1 | blue4 |
| Dnm2 | blue3 |
| Mir199a-1 | green4 |
| Tmed1 | blue3 |
| AB124611 | blue4 |
| Carm1 | blue4 |
| Yipf2 | green4 |
| 1810026J23Rik | blue4 |
| Smarca4 | blue4 |
| Ldlr | deeppink |
| Spc24 | blue4 |
| Kank2 | blue4 |
| Dock6 | blue3 |
| Gm6484 | blue4 |
| Rab3d | blue4 |
| Tmem205 | blue4 |
| Ccdc159 | darkolivegreen |
| Lppr2 | blue3 |
| Swsap1 | blue3 |
| Epor | blue4 |
| Rgl3 | green4 |
| Ccdc151 | blue4 |
| Prkcsh | deeppink |
| Zfp653 | blue3 |
| Ecsit | green4 |
| Cnn1 | lightcoral |
| Elof1 | deeppink |
| Acp5 | green4 |
| Pigyl | blue3 |
| Zfp809 | blue4 |
| Zfp810 | coral1 |
| Anln | blue4 |
| 9530077C05Rik | darkgrey |
| Rp9 | green4 |
| Gm17545 | blue4 |
| Bbs9 | firebrick2 |
| Dpy19l1 | blue4 |
| Gm10180 | blue4 |
| Herpud2 | blue4 |
| Gm29642 | darkolivegreen |
| 7-Sep | blue4 |
| Eepd1 | blue4 |
| Glb1l2 | darkgrey |
| Acad8 | mistyrose |
| Thyn1 | blue4 |
| Vps26b | blue3 |
| Ncapd3 | blue3 |
| Jam3 | green4 |
| Snx19 | blue3 |
| Adamts15 | blue4 |
| Adamts8 | green4 |
| Zbtb44 | mistyrose |
| St14 | mistyrose |
| Gm16071 | blue4 |
| Aplp2 | blue4 |
| Prdm10 | blue4 |
| Nfrkb | blue3 |
| Tmem45b | mistyrose |
| Arhgap32 | lightsteelblue |
| Kcnj1 | darkolivegreen |
| Fli1 | blue4 |
| Ets1 | blue4 |
| Gm10698 | blue4 |
| St3gal4 | blue4 |
| 4930581F22Rik | blue3 |
| Dcps | blue3 |
| Tirap | blue4 |
| Srpr | darkolivegreen |
| Foxred1 | blue4 |
| Fam118b | blue4 |
| Rpusd4 | blue3 |
| Cdon | blue4 |
| Chek1 | blue4 |
| Stt3a | blue4 |
| Ei24 | deeppink1 |
| Pknox2 | blue4 |
| Tmem218 | blue3 |
| Gm10177 | blue4 |
| Robo4 | darkgrey |
| Robo3 | green4 |
| Msantd2 | blue3 |
| Esam | darkgrey |
| Vsig2 | blue4 |
| Nrgn | coral1 |
| Spa17 | blue4 |
| Siae | darkolivegreen |
| Tbrg1 | blue4 |
| Vwa5a | blue4 |
| Zfp202 | blue4 |
| Gramd1b | blue4 |
| Clmp | blue4 |
| Hspa8 | darkgrey |
| Ubash3b | blue4 |
| 2610203C20Rik | blue4 |
| Gm28119 | blue3 |
| Sorl1 | blue3 |
| Sc5d | mistyrose |
| Tbcel | blue4 |
| Arhgef12 | blue4 |
| Tmem136 | coral1 |
| Oaf | darkgrey |
| Pvrl1 | blue4 |
| Thy1 | blue4 |
| Rnf26 | blue3 |
| Usp2 | blue4 |
| Mfrp | blue4 |
| Gm10687 | blue4 |
| Mcam | blue4 |
| Cbl | blue4 |
| Pdzd3 | green4 |
| Nlrx1 | blue4 |
| Hinfp | blue4 |
| C2cd2l | coral1 |
| Dpagt1 | blue3 |
| H2afx | blue4 |
| Hmbs | blue4 |
| Vps11 | brown1 |
| Gm10080 | blue4 |
| Hyou1 | mistyrose |
| Slc37a4 | blue4 |
| Trappc4 | blue4 |
| Rps25 | blue4 |
| Ccdc84 | blue3 |
| Upk2 | lightcoral |
| Gm9830 | blue4 |
| C030014I23Rik | blue3 |
| Bcl9l | deeppink |
| Ddx6 | firebrick2 |
| Treh | blue4 |
| Phldb1 | blue4 |
| Gm24166 | darkgrey |
| Arcn1 | coral1 |
| Ift46 | darkgrey |
| Tmem25 | darkolivegreen |
| Ttc36 | blue4 |
| Kmt2a | blue4 |
| Atp5l | blue4 |
| Ube4a | firebrick2 |
| Mpzl2 | blue4 |
| Mpzl3 | blue4 |
| Scn4b | darkolivegreen |
| Fxyd6 | deeppink |
| Fxyd2 | indianred2 |
| Cep164 | blue3 |
| Bace1 | blue3 |
| Gm16536 | blue3 |
| Rnf214 | blue3 |
| Pcsk7 | green4 |
| Tagln | blue4 |
| Sidt2 | blue3 |
| Mir7087 | darkgrey |
| Pafah1b2 | blue4 |
| Sik3 | darkgrey |
| Apoa1 | blue4 |
| Apoc3 | darkolivegreen |
| Apoa4 | blue4 |
| Zpr1 | blue4 |
| Bud13 | blue4 |
| Cadm1 | blue4 |
| Nxpe4 | blue4 |
| Rexo2 | blue4 |
| Rbm7 | green4 |
| Gm5617 | blue4 |
| Nnmt | lightcoral |
| Zbtb16 | blue3 |
| Usp28 | blue4 |
| Zw10 | blue3 |
| Ttc12 | blue4 |
| Ncam1 | blue4 |
| Rpl10-ps3 | blue4 |
| Plet1os | darkgrey |
| Plet1 | darkgrey |
| Pts | mistyrose |
| Bco2 | blue4 |
| Il18 | saddlebrown |
| Sdhd | blue4 |
| Timm8b | blue4 |
| AU019823 | blue4 |
| Pih1d2 | coral1 |
| Dlat | mistyrose |
| Dixdc1 | firebrick2 |
| 2310030G06Rik | blue4 |
| Hspb2 | blue3 |
| Cryab | darkgrey |
| 1110032A03Rik | mistyrose |
| Fdxacb1 | blue3 |
| Alg9 | blue4 |
| Ppp2r1b | blue4 |
| Sik2 | blue4 |
| Layn | blue4 |
| Fdx1 | blue4 |
| Gm6981 | blue4 |
| Rdx | firebrick2 |
| Zc3h12c | deeppink |
| Ddx10 | blue4 |
| Exph5 | firebrick2 |
| Kdelc2 | blue4 |
| 4930550C14Rik | coral1 |
| Atm | blue3 |
| Npat | coral1 |
| Acat1 | mistyrose |
| Cul5 | indianred2 |
| Gm16124 | blue4 |
| Slc35f2 | mistyrose |
| Dmxl2 | blue4 |
| Cib2 | lightcoral |
| Idh3a | blue3 |
| Dnaja4 | blue4 |
| Wdr61 | coral1 |
| Ireb2 | coral1 |
| Hykk | darkolivegreen |
| Psma4 | coral1 |
| Ube2q2 | coral1 |
| Fbxo22 | blue3 |
| Nrg4 | coral1 |
| Etfa | mistyrose |
| Scaper | blue4 |
| Rcn2 | blue4 |
| Tspan3 | blue4 |
| Peak1 | blue4 |
| Gm24270 | blue4 |
| Hmg20a | blue3 |
| Cspg4 | green4 |
| Snx33 | blue4 |
| Imp3 | saddlebrown |
| Snupn | blue4 |
| Ptpn9 | blue4 |
| Gm10658 | blue4 |
| Sin3a | blue4 |
| 2700012I20Rik | blue3 |
| Man2c1 | green4 |
| Neil1 | blue3 |
| Commd4 | blue4 |
| 1700017B05Rik | darkgrey |
| Ppcdc | blue3 |
| Scamp5 | coral1 |
| Cox5a | green4 |
| Fam219b | blue3 |
| Mpi | blue4 |
| Scamp2 | mistyrose |
| Ulk3 | blue3 |
| Csk | blue4 |
| Edc3 | blue4 |
| Clk3 | blue4 |
| Arid3b | blue4 |
| Ubl7 | green4 |
| Sema7a | blue4 |
| Stra6 | blue4 |
| Islr | deeppink |
| Islr2 | blue3 |
| Pml | blue3 |
| Stoml1 | blue3 |
| Loxl1 | blue4 |
| 4930461G14Rik | darkolivegreen |
| 6030419C18Rik | blue4 |
| Nptn | coral1 |
| Rec114 | blue4 |
| Neo1 | blue4 |
| Adpgk | blue4 |
| Bbs4 | coral1 |
| Arih1 | coral1 |
| Hexa | coral1 |
| Parp6 | blue3 |
| Pkm | blue4 |
| Senp8 | coral1 |
| Myo9a | blue4 |
| Larp6 | coral1 |
| Uaca | deeppink1 |
| Gm9869 | darkgrey |
| Tle3 | coral1 |
| Rplp1 | blue4 |
| Kif23 | darkgrey |
| Paqr5 | blue4 |
| Glce | blue4 |
| Anp32a | coral1 |
| Coro2b | deeppink |
| Fem1b | blue4 |
| Cln6 | green4 |
| Calml4 | blue3 |
| Pias1 | deeppink |
| Map2k5 | deeppink |
| 2300009A05Rik | green4 |
| Aagab | blue3 |
| Smad3 | blue4 |
| Smad6 | darkgrey |
| Lctl | blue4 |
| Zwilch | blue4 |
| Rpl4 | blue4 |
| Snord16a | darkgrey |
| Gm22571 | blue4 |
| Gm23136 | darkgrey |
| Gm22455 | darkgrey |
| Snapc5 | blue4 |
| Map2k1 | blue4 |
| Tipin | blue4 |
| Dis3l | blue3 |
| Rab11a | blue4 |
| Dennd4a | blue4 |
| Vwa9 | blue4 |
| Ptplad1 | blue4 |
| Dpp8 | coral1 |
| Parp16 | blue3 |
| Clpx | mistyrose |
| Pdcd7 | blue4 |
| Rasl12 | blue4 |
| Slc51b | blue3 |
| Mtfmt | blue3 |
| Spg21 | blue4 |
| Plekho2 | darkgrey |
| Rbpms2 | blue3 |
| Oaz2 | blue4 |
| Zfp609 | mistyrose |
| Trip4 | coral1 |
| Csnk1g1 | blue4 |
| Ppib | blue4 |
| Snx22 | blue3 |
| Snx1 | blue3 |
| Fam96a | mistyrose |
| Dapk2 | blue4 |
| Herc1 | blue4 |
| Usp3 | blue4 |
| Gm15563 | blue3 |
| Car12 | blue4 |
| Aph1b | blue4 |
| Aph1c | darkolivegreen |
| Rab8b | blue4 |
| Rps27l | blue4 |
| Lactb | deeppink |
| Tpm1 | blue4 |
| Tln2 | blue4 |
| Vps13c | blue4 |
| Rora | brown1 |
| Ice2 | blue3 |
| Anxa2 | blue4 |
| Bnip2 | blue4 |
| Gtf2a2 | blue4 |
| Fam81a | blue4 |
| Myo1e | darkgrey |
| Ccnb2 | blue3 |
| Rnf111 | blue4 |
| Sltm | blue4 |
| Fam63b | mistyrose |
| Adam10 | blue4 |
| Aldh1a2 | blue4 |
| Polr2m | blue4 |
| Cgnl1 | blue4 |
| Tcf12 | blue4 |
| Zfp280d | coral1 |
| RP24-282D16.9 | blue3 |
| RP24-282D16.8 | blue4 |
| Tex9 | blue3 |
| Rfx7 | blue4 |
| Nedd4 | blue4 |
| RP23-296I6.8 | darkgrey |
| RP23-461P14.1 | blue3 |
| Ccpg1os | blue4 |
| Gm27353 | firebrick2 |
| Pigb | blue3 |
| 5031420N21Rik | green4 |
| Rab27a | mistyrose |
| Rsl24d1 | blue4 |
| Wdr72 | blue4 |
| Fam214a | mistyrose |
| Arpp19 | mistyrose |
| Myo5a | blue4 |
| Myo5c | blue3 |
| Gnb5 | blue3 |
| Mapk6 | darkgrey |
| Leo1 | blue4 |
| Tmod3 | blue4 |
| Lysmd2 | blue4 |
| Hmgcll1 | blue3 |
| Tinag | deeppink |
| Gm19541 | darkolivegreen |
| Lrrc1 | blue3 |
| Gclc | blue4 |
| Elovl5 | blue4 |
| Gcm1 | darkolivegreen |
| Fbxo9 | mistyrose |
| Ick | blue4 |
| Rn7sk | blue3 |
| C920006O11Rik | darkolivegreen |
| Gsta4 | blue4 |
| Gsta1 | blue4 |
| Gm10639 | blue4 |
| Gsta2 | blue4 |
| Mto1 | green4 |
| Eef1a1 | blue4 |
| Slc17a5 | mistyrose |
| Col12a1 | blue4 |
| Cox7a2 | blue4 |
| Tmem30a | mistyrose |
| Senp6 | coral1 |
| Myo6 | firebrick2 |
| Htr1b | darkgrey |
| Gm9531 | blue4 |
| Irak1bp1 | blue4 |
| Phip | blue4 |
| Hmgn3 | blue4 |
| Gm27216 | blue3 |
| Sh3bgrl2 | blue4 |
| Bckdhb | blue4 |
| Fam46a | deeppink1 |
| Ibtk | blue4 |
| Ube2cbp | blue4 |
| Dopey1 | blue4 |
| RP23-27L18.4 | blue3 |
| Pgm3 | coral1 |
| Rwdd2a | darkgrey |
| Me1 | blue4 |
| Cyb5r4 | blue4 |
| Cep162 | blue4 |
| Nt5e | deeppink |
| Snx14 | blue3 |
| Gm28229 | blue4 |
| Syncrip | blue4 |
| Snhg5 | blue4 |
| Zfp949 | deeppink |
| Mthfsl | coral1 |
| Tmed3 | deeppink |
| Ctsh | blue4 |
| Morf4l1 | green4 |
| Adamts7 | blue4 |
| Tbc1d2b | blue4 |
| Plscr1 | blue4 |
| Mir6386 | blue4 |
| Plscr2 | coral1 |
| Plscr4 | blue3 |
| Plod2 | blue4 |
| 1190002N15Rik | blue3 |
| Chst2 | blue4 |
| U2surp | blue4 |
| Gm28424 | blue3 |
| Paqr9 | blue3 |
| Trpc1 | firebrick2 |
| Pls1 | blue4 |
| Atr | coral1 |
| Xrn1 | blue4 |
| Gk5 | coral1 |
| Tfdp2 | mistyrose |
| Atp1b3 | blue4 |
| Rnf7 | lightsteelblue |
| Rasa2 | blue4 |
| n-R5s87 | deeppink1 |
| Zbtb38 | blue4 |
| Pxylp1 | blue3 |
| Gm10123 | blue4 |
| Spsb4 | mistyrose |
| Gm16010 | blue3 |
| Gm26767 | antiquewhite2 |
| Slc25a36 | firebrick2 |
| Gm16185 | blue3 |
| Clstn2 | blue4 |
| Nmnat3 | blue4 |
| Rbp1 | blue4 |
| 4930579K19Rik | darkgrey |
| Copb2 | blue4 |
| Mrps22 | blue4 |
| RP24-116C1.9 | darkgrey |
| Faim | blue4 |
| Gm1123 | mistyrose |
| Pik3cb | blue4 |
| Cep70 | blue3 |
| Mras | blue3 |
| Armc8 | coral1 |
| Dbr1 | blue4 |
| A4gnt | blue4 |
| Dzip1l | blue3 |
| Gm16004 | blue4 |
| Il20rb | darkgrey |
| Nck1 | coral1 |
| Slc35g2 | blue4 |
| Stag1 | blue4 |
| Pccb | blue4 |
| Msl2 | blue4 |
| Ppp2r3a | coral1 |
| Cep63 | firebrick2 |
| Anapc13 | blue4 |
| Amotl2 | darkgrey |
| Ryk | blue4 |
| Slco2a1 | blue4 |
| Rab6b | green4 |
| Srprb | blue4 |
| Topbp1 | blue4 |
| Cdv3 | blue4 |
| Tmem108 | mistyrose |
| Nphp3 | blue3 |
| Uba5 | blue4 |
| RP23-380A10.6 | darkgrey |
| Acad11 | blue4 |
| Dnajc13 | firebrick2 |
| RP23-353G11.6 | blue4 |
| Acpp | blue4 |
| Cpne4 | blue4 |
| Mrpl3 | blue4 |
| Nudt16 | blue4 |
| Aste1 | deeppink1 |
| Atp2c1 | firebrick2 |
| Pik3r4 | blue3 |
| Gm22720 | lightcoral |
| Glyctk | coral1 |
| Wdr82 | blue4 |
| Ppm1m | blue4 |
| Twf2 | blue4 |
| Alas1 | mistyrose |
| Poc1a | blue4 |
| Dusp7 | blue4 |
| Rpl29 | blue4 |
| Acy1 | blue4 |
| Abhd14a | blue4 |
| Abhd14b | darkolivegreen |
| Pcbp4 | green4 |
| Parp3 | blue4 |
| Rrp9 | blue4 |
| Tex264 | blue4 |
| Rad54l2 | blue3 |
| Vprbp | blue4 |
| Manf | darkgrey |
| Rbm15b | mistyrose |
| Mapkapk3 | blue4 |
| Gm17040 | green4 |
| Cish | coral1 |
| Hemk1 | darkolivegreen |
| 6430571L13Rik | blue4 |
| Tmem115 | deeppink |
| Cyb561d2 | blue4 |
| Nprl2 | blue4 |
| Zmynd10 | blue4 |
| Rassf1 | darkgrey |
| Gm9917 | blue3 |
| Tusc2 | deeppink1 |
| Hyal2 | deeppink1 |
| Hyal1 | darkgrey |
| Nat6 | darkgrey |
| Hyal3 | blue4 |
| Ifrd2 | green4 |
| RP23-456B9.23 | blue3 |
| RP23-456B9.24 | blue3 |
| Sema3b | green4 |
| Gm29510 | blue3 |
| Gnai2 | blue4 |
| Slc38a3 | blue3 |
| Gnat1 | blue4 |
| Sema3f | blue4 |
| Rbm5 | blue3 |
| Rbm6 | blue4 |
| Mon1a | green4 |
| Uba7 | blue4 |
| Fam212a | blue4 |
| Ip6k1 | brown1 |
| Gmppb | mistyrose |
| Rnf123 | green4 |
| Amigo3 | mistyrose |
| Apeh | blue4 |
| Dag1 | blue3 |
| Nicn1 | blue3 |
| Amt | darkolivegreen |
| Tcta | darkgrey |
| Rhoa | blue4 |
| Gpx1 | blue4 |
| Usp4 | darkolivegreen |
| RP23-448A11.16 | blue4 |
| Klhdc8b | blue3 |
| Ccdc71 | blue4 |
| Lamb2 | green4 |
| Usp19 | mistyrose |
| Qars | deeppink |
| Qrich1 | blue4 |
| Impdh2 | blue4 |
| Ndufaf3 | blue4 |
| Dalrd3 | green4 |
| Wdr6 | blue3 |
| Arih2 | brown1 |
| Slc25a20 | orangered |
| Prkar2a | blue4 |
| Ip6k2 | blue3 |
| Nckipsd | blue3 |
| Slc26a6 | green4 |
| Uqcrc1 | blue4 |
| Col7a1 | blue4 |
| Pfkfb4 | blue3 |
| Shisa5 | deeppink |
| Atrip | blue4 |
| Tma7 | blue4 |
| Ccdc51 | darkgrey |
| Plxnb1 | green4 |
| Fbxw15 | blue4 |
| Spink8 | deeppink |
| 3000002C10Rik | blue3 |
| Nme6 | blue3 |
| Cdc25a | blue4 |
| Map4 | blue4 |
| Gm10615 | blue3 |
| Smarcc1 | blue4 |
| Mir6236 | coral1 |
| Elp6 | blue4 |
| Scap | green4 |
| Ptpn23 | darkgrey |
| Klhl18 | blue4 |
| Kif9 | blue4 |
| Setd2 | coral1 |
| Nradd | blue4 |
| Nbeal2 | blue4 |
| Ccdc12 | blue4 |
| Pth1r | green4 |
| Tmie | blue3 |
| Als2cl | blue3 |
| Ccrl2 | mistyrose |
| Lrrfip2 | blue4 |
| Mlh1 | blue4 |
| Epm2aip1 | blue3 |
| Dclk3 | blue3 |
| Pdcd6ip | blue4 |
| Clasp2 | blue4 |
| Ubp1 | blue4 |
| Fbxl2 | blue4 |
| Crtap | blue4 |
| Glb1 | blue4 |
| Tmppe | darkgrey |
| Cnot10 | blue3 |
| Dync1li1 | blue4 |
| Cmtm6 | blue3 |
| Cmtm7 | blue4 |
| Cmtm8 | darkolivegreen |
| Gpd1l | darkgrey |
| Osbpl10 | blue4 |
| Rps27rt | blue4 |
| Gm27002 | blue4 |
| Stt3b | firebrick2 |
| Gm9385 | blue4 |
| Tgfbr2 | blue4 |
| Rbms3 | blue4 |
| Gm17396 | blue3 |
| Azi2 | mistyrose |
| Cmc1 | darkgrey |
| Golga4 | blue4 |
| Itga9 | blue4 |
| Gm10157 | blue4 |
| Gm2415 | green4 |
| Ctdspl | blue4 |
| Mir26a-1 | darkgrey |
| Vill | blue4 |
| Plcd1 | darkgrey |
| Dlec1 | green4 |
| Acaa1b | blue3 |
| Slc22a13 | darkolivegreen |
| Oxsr1 | blue4 |
| Myd88 | blue4 |
| Acaa1a | blue4 |
| Xylb | coral1 |
| Acvr2b | blue4 |
| 4930516B21Rik | blue3 |
| Exog | blue4 |
| Wdr48 | coral1 |
| Cx3cr1 | blue4 |
| Gorasp1 | blue4 |
| Csrnp1 | coral1 |
| Slc25a38 | mistyrose |
| Rpsa | blue4 |
| Gm24044 | blue4 |
| Gm26448 | blue3 |
| Snora62 | blue4 |
| Eif1b | blue4 |
| Rpl14 | blue4 |
| 5830454E08Rik | blue3 |
| Ctnnb1 | blue4 |
| Trak1 | blue4 |
| Cck | darkolivegreen |
| Vipr1 | blue4 |
| Sec22c | blue4 |
| Deb1 | mistyrose |
| Nktr | deeppink1 |
| Zfp651 | blue3 |
| Hhatl | green4 |
| Higd1a | green4 |
| Pomgnt2 | green4 |
| Snrk | blue3 |
| Ano10 | blue4 |
| Abhd5 | coral1 |
| Tcaim | darkolivegreen |
| Zfp445 | blue4 |
| Zkscan7 | darkolivegreen |
| Zfp105 | blue4 |
| 1110059G10Rik | antiquewhite2 |
| Tmem42 | blue4 |
| Zdhhc3 | blue4 |
| Exosc7 | blue4 |
| Clec3b | green4 |
| Cdcp1 | darkgrey |
| Tmem158 | blue4 |
| Scp2-ps2 | mistyrose |
| Lars2 | deeppink1 |
| Limd1 | blue3 |
| Sacm1l | coral1 |
| Slc6a20b | darkolivegreen |
| Slc6a20a | mistyrose |
| Gm10052 | blue4 |
| Lztfl1 | firebrick2 |
| Fyco1 | blue3 |
| Ccr1 | blue4 |
| Ccr2 | blue4 |
| Ccr5 | honeydew |
| 2010315B03Rik | coral1 |
| Ppp2r3d | blue4 |
| Iyd | blue4 |
| Plekhg1 | blue4 |
| Mthfd1l | blue4 |
| Akap12 | darkgrey |
| Zbtb2 | darkgrey |
| Gm21781 | blue3 |
| Rmnd1 | darkolivegreen |
| 1700052N19Rik | blue3 |
| Ccdc170 | darkolivegreen |
| Esr1 | darkolivegreen |
| Syne1 | blue4 |
| Myct1 | blue3 |
| Fbxo5 | blue4 |
| Mtrf1l | blue3 |
| Ipcef1 | darkolivegreen |
| Gm15536 | blue4 |
| Cnksr3 | darkgrey |
| Lrp11 | blue4 |
| Pcmt1 | deeppink |
| Nup43 | blue4 |
| Lats1 | blue4 |
| Katna1 | blue4 |
| Ginm1 | coral1 |
| Ppil4 | blue4 |
| Zc3h12d | mistyrose |
| Tab2 | blue4 |
| Ust | blue4 |
| Sash1 | blue4 |
| Stxbp5 | firebrick2 |
| Gm25682 | blue4 |
| Rab32 | blue4 |
| Shprh | coral1 |
| Fbxo30 | darkgrey |
| Epm2a | blue4 |
| Utrn | blue4 |
| Stx11 | darkgrey |
| Sf3b5 | blue4 |
| Plagl1 | darkgrey |
| Ltv1 | blue4 |
| Phactr2 | blue4 |
| Fuca2 | mistyrose |
| Pex3 | mistyrose |
| Adat2 | blue4 |
| Aig1 | blue4 |
| Hivep2 | darkgrey |
| Gpr126 | blue4 |
| Vta1 | blue4 |
| Gm8355 | darkgrey |
| Cited2 | blue4 |
| Heca | deeppink |
| Abracl | blue4 |
| Reps1 | blue4 |
| Ccdc28a | darkolivegreen |
| Nhsl1 | blue4 |
| D10Bwg1379e | blue4 |
| Perp | blue4 |
| Tnfaip3 | darkgrey |
| Ifngr1 | blue4 |
| Pex7 | blue4 |
| Map3k5 | deeppink |
| Map7 | orangered |
| Bclaf1 | coral1 |
| Pde7b | blue4 |
| Ahi1 | blue3 |
| Hbs1l | coral1 |
| Aldh8a1 | blue4 |
| Sgk1 | blue3 |
| E030030I06Rik | blue3 |
| Gm4895 | blue4 |
| Slc2a12 | blue4 |
| Tbpl1 | blue4 |
| Tcf21 | coral1 |
| Rps12 | blue4 |
| Snora33 | blue4 |
| Snord100 | blue4 |
| Gm23130 | darkgrey |
| Slc18b1 | blue3 |
| Vnn1 | coral1 |
| Gm15137 | blue4 |
| Stx7 | blue4 |
| Ctgf | darkolivegreen |
| Enpp1 | darkolivegreen |
| Enpp3 | mistyrose |
| Med23 | blue4 |
| Mir6905 | blue4 |
| Akap7 | coral1 |
| Epb4.1l2 | blue4 |
| Smlr1 | blue4 |
| L3mbtl3 | blue4 |
| Arhgap18 | firebrick2 |
| Lama2 | blue4 |
| Ptprk | green4 |
| Gm9824 | blue4 |
| Echdc1 | coral1 |
| Rnf146 | coral1 |
| Gm25596 | coral1 |
| Gm10275 | blue4 |
| Cenpw | blue4 |
| Trmt11 | blue4 |
| Hint3 | blue4 |
| Ncoa7 | firebrick2 |
| Hddc2 | blue4 |
| Tpd52l1 | blue3 |
| Rnf217 | brown1 |
| Trdn | blue4 |
| Gm10327 | blue4 |
| Zufsp | blue4 |
| Gm17567 | blue3 |
| Rwdd1 | blue4 |
| Fam26e | blue4 |
| Dse | blue4 |
| Tspyl1 | coral1 |
| Nt5dc1 | mistyrose |
| Tspyl4 | blue3 |
| Frk | blue4 |
| Hdac2 | coral1 |
| Marcks | blue4 |
| Lama4 | blue4 |
| Fyn | darkgrey |
| Traf3ip2 | blue4 |
| Rev3l | blue4 |
| AA474331 | blue3 |
| G630090E17Rik | blue4 |
| BC021785 | blue3 |
| AI317395 | darkgrey |
| Slc16a10 | coral1 |
| Rpf2 | blue4 |
| Gtf3c6 | blue4 |
| Amd1 | blue4 |
| Cdk19 | firebrick2 |
| Ddo | darkolivegreen |
| Mettl24 | blue3 |
| Cdc40 | coral1 |
| Wasf1 | blue4 |
| Fig4 | coral1 |
| Gm25526 | darkgrey |
| Zbtb24 | blue3 |
| Mical1 | blue4 |
| Smpd2 | blue4 |
| Ppil6 | blue4 |
| Cd164 | firebrick2 |
| Ccdc162 | blue4 |
| Cep57l1 | blue3 |
| Sesn1 | blue3 |
| Foxo3 | blue3 |
| Lace1 | mistyrose |
| Snx3 | deeppink |
| Ostm1 | coral1 |
| Sec63 | blue4 |
| Pdss2 | blue3 |
| Gm3699 | blue4 |
| Bend3 | blue4 |
| Gm9803 | blue4 |
| 1700021F05Rik | blue3 |
| Cd24a | blue4 |
| Qrsl1 | blue4 |
| Rtn4ip1 | darkolivegreen |
| Aim1 | blue4 |
| Atg5 | coral1 |
| Prep | blue4 |
| Ascc3 | blue4 |
| Sim1 | firebrick2 |
| Gp49a | darkgrey |
| Lilrb4 | darkgrey |
| Dcbld1 | blue4 |
| Gopc | firebrick2 |
| Nepn | darkolivegreen |
| Nus1 | firebrick2 |
| Slc35f1 | darkolivegreen |
| Cep85l | darkgrey |
| Pln | darkgrey |
| Mcm9 | blue4 |
| Asf1a | green4 |
| Fam184a | lightsteelblue |
| Man1a | blue4 |
| Tbc1d32 | firebrick2 |
| Msl3l2 | blue4 |
| Gja1 | blue4 |
| Hsf2 | blue4 |
| Serinc1 | mistyrose |
| Fabp7 | saddlebrown |
| Smpdl3a | blue4 |
| Gcc2 | blue4 |
| Lims1 | blue4 |
| Ranbp2 | coral1 |
| Ccdc138 | blue3 |
| 10-Sep | blue4 |
| P4ha1 | blue4 |
| Mcu | blue4 |
| Micu1 | deeppink |
| Dnajb12 | green4 |
| Ddit4 | blue3 |
| Anapc16 | mistyrose |
| Ascc1 | blue4 |
| Chst3 | blue4 |
| Psap | darkolivegreen |
| 4632428N05Rik | blue4 |
| Slc29a3 | green4 |
| Unc5b | blue4 |
| Pcbd1 | blue4 |
| Sgpl1 | blue4 |
| Adamts14 | blue4 |
| Pald1 | blue4 |
| Eif4ebp2 | green4 |
| Lrrc20 | blue4 |
| Ppa1 | blue4 |
| Sar1a | green4 |
| Tysnd1 | green4 |
| Aifm2 | blue4 |
| H2afy2 | blue4 |
| Col13a1 | blue4 |
| Gm5424 | blue4 |
| 2010107G23Rik | blue3 |
| Tspan15 | deeppink |
| Mir7662 | blue3 |
| Hk1 | green4 |
| Hkdc1 | blue4 |
| Supv3l1 | blue3 |
| Vps26a | coral1 |
| Srgn | darkolivegreen |
| 2510003E04Rik | darkolivegreen |
| Ddx21 | blue4 |
| Ddx50 | coral1 |
| Ccar1 | blue4 |
| Snord98 | darkgrey |
| Slc25a16 | blue4 |
| Dna2 | blue4 |
| Rufy2 | blue4 |
| Hnrnph3 | blue3 |
| Pbld1 | blue4 |
| Herc4 | firebrick2 |
| Sirt1 | brown1 |
| Dnajc12 | blue3 |
| Reep3 | blue4 |
| Jmjd1c | darkgrey |
| Gm26359 | darkgrey |
| Nrbf2 | blue3 |
| Egr2 | mistyrose |
| Ado | blue4 |
| Arid5b | darkgrey |
| 1700040L02Rik | darkolivegreen |
| Tmem26 | blue4 |
| Rhobtb1 | blue4 |
| A930033H14Rik | blue3 |
| Cdk1 | blue4 |
| Ank3 | lightsteelblue |
| Ccdc6 | blue3 |
| Slc16a9 | darkgrey |
| Fam13c | blue3 |
| Phyhipl | blue3 |
| Bicc1 | blue4 |
| Tfam | mistyrose |
| Ube2d1 | blue4 |
| Cisd1 | blue4 |
| Ipmk | blue3 |
| Zwint | blue4 |
| Gm6419 | blue4 |
| Gnaz | blue3 |
| Rab36 | green4 |
| Bcr | deeppink |
| Specc1l | blue4 |
| Adora2a | mistyrose |
| Gucd1 | darkgrey |
| Upb1 | blue4 |
| Snrpd3 | coral1 |
| Lrrc75b | blue4 |
| Ggt1 | blue4 |
| Ggt5 | blue4 |
| Susd2 | coral1 |
| Cabin1 | blue3 |
| Ddt | blue4 |
| Gstt3 | blue4 |
| Gstt1 | blue4 |
| Gstt2 | blue4 |
| Mif | blue4 |
| Derl3 | antiquewhite2 |
| Smarcb1 | blue4 |
| Mmp11 | blue3 |
| Chchd10 | green4 |
| Zfp280b | coral1 |
| Slc5a4a | darkolivegreen |
| Prmt2 | blue3 |
| Dip2a | blue3 |
| Pcnt | blue4 |
| Ybey | darkgrey |
| Mcm3ap | blue3 |
| Lss | blue3 |
| Gm15343 | darkolivegreen |
| Ftcd | blue4 |
| Col6a2 | blue4 |
| Col6a1 | blue4 |
| Pcbp3 | lightsteelblue |
| Gm10787 | darkolivegreen |
| Slc19a1 | blue4 |
| Col18a1 | blue4 |
| Gm10941 | darkgrey |
| Pofut2 | mistyrose |
| Adarb1 | blue3 |
| Fam207a | blue3 |
| Itgb2 | honeydew |
| Pttg1ip | darkgrey |
| Sumo3 | mistyrose |
| Ube2g2 | darkgrey |
| 1700009J07Rik | blue3 |
| Lrrc3 | blue4 |
| 1810043G02Rik | green4 |
| Pfkl | green4 |
| Icosl | darkgrey |
| D10Jhu81e | blue4 |
| Pwp2 | blue4 |
| Trappc10 | blue4 |
| Agpat3 | blue4 |
| Gm10146 | blue4 |
| Rrp1 | blue4 |
| Cstb | blue4 |
| Pdxk | darkgrey |
| Mir6908 | coral1 |
| Ilvbl | deeppink |
| Syde1 | blue4 |
| 2610008E11Rik | blue4 |
| Ppap2c | blue4 |
| Mir6909 | darkgrey |
| Mier2 | blue3 |
| Tpgs1 | deeppink |
| Cdc34 | firebrick2 |
| Bsg | blue4 |
| Hcn2 | blue3 |
| Polrmt | green4 |
| Rnf126 | blue4 |
| Fstl3 | blue4 |
| Palm | blue3 |
| Misp | brown1 |
| Ptbp1 | mistyrose |
| Lppr3 | blue3 |
| Cfd | lightcoral |
| Med16 | coral1 |
| R3hdm4 | deeppink |
| Kiss1r | green4 |
| Arid3a | blue4 |
| Wdr18 | deeppink |
| Tmem259 | coral1 |
| Cnn2 | blue4 |
| Abca7 | blue3 |
| Hmha1 | blue4 |
| Polr2e | blue4 |
| Gpx4 | blue4 |
| Sbno2 | darkgrey |
| Stk11 | deeppink |
| Dos | darkgrey |
| Atp5d | indianred2 |
| Midn | darkgrey |
| Cirbp | blue3 |
| 1600002K03Rik | blue4 |
| Mum1 | blue3 |
| Ndufs7 | green4 |
| Gamt | green4 |
| Dazap1 | blue4 |
| Gm15122 | blue3 |
| Rps15 | blue4 |
| 2310011J03Rik | blue4 |
| Pcsk4 | blue3 |
| Reep6 | darkolivegreen |
| Plk5 | blue4 |
| Mex3d | blue4 |
| Mbd3 | deeppink |
| Uqcr11 | blue4 |
| Tcf3 | blue3 |
| Rexo1 | blue4 |
| Klf16 | mistyrose |
| Abhd17a | deeppink |
| Gm29093 | blue4 |
| Adat3 | blue3 |
| Csnk1g2 | deeppink |
| Btbd2 | green4 |
| Mknk2 | blue3 |
| Mob3a | blue4 |
| Izumo4 | darkolivegreen |
| Ap3d1 | green4 |
| Dot1l | darkgrey |
| Plekhj1 | blue4 |
| Sf3a2 | mistyrose |
| Oaz1 | coral1 |
| Lsm7 | blue4 |
| Sppl2b | green4 |
| Tmprss9 | blue4 |
| Timm13 | deeppink |
| Lmnb2 | blue4 |
| Gadd45b | darkgrey |
| Slc39a3 | brown1 |
| Sgta | deeppink |
| Thop1 | blue4 |
| Creb3l3 | mistyrose |
| Map2k2 | deeppink |
| Zbtb7a | blue3 |
| Pias4 | green4 |
| Eef2 | coral1 |
| Snord37 | deeppink |
| Dapk3 | blue4 |
| Zfr2 | blue3 |
| Mrpl54 | firebrick2 |
| Apba3 | green4 |
| Tjp3 | blue3 |
| Pip5k1c | blue3 |
| Cactin | blue4 |
| Tbxa2r | brown1 |
| Gipc3 | blue4 |
| Hmg20b | blue3 |
| Mfsd12 | blue4 |
| Fzr1 | blue3 |
| Dohh | deeppink |
| Mir6913 | orangered |
| Smim24 | blue4 |
| Nfic | blue3 |
| Ncln | deeppink |
| Gna11 | blue3 |
| Aes | indianred2 |
| Tle2 | blue3 |
| Tle6 | blue3 |
| Sirt6 | blue3 |
| Ankrd24 | darkolivegreen |
| Zfp433 | green4 |
| Zfp873 | blue4 |
| AU041133 | coral1 |
| Zfp938 | coral1 |
| 1190007I07Rik | blue3 |
| Tdg | darkgrey |
| Hcfc2 | blue4 |
| Nfyb | blue4 |
| Txnrd1 | blue4 |
| Gm4799 | blue4 |
| Chst11 | lightsteelblue |
| Slc41a2 | green4 |
| D10Wsu102e | deeppink1 |
| Aldh1l2 | blue4 |
| A230046K03Rik | blue4 |
| Appl2 | darkolivegreen |
| 1500009L16Rik | lightsteelblue |
| Nuak1 | blue3 |
| Ckap4 | blue4 |
| Tcp11l2 | blue3 |
| Polr3b | blue4 |
| Ric8b | blue4 |
| Tmem263 | blue4 |
| Mterf2 | firebrick2 |
| Cry1 | green4 |
| Gm8394 | blue4 |
| Btbd11 | antiquewhite2 |
| Pwp1 | blue4 |
| Prdm4 | blue4 |
| Rtcb | antiquewhite2 |
| Fbxo7 | blue4 |
| Gm24430 | blue3 |
| Syn3 | blue4 |
| Timp3 | blue4 |
| 1810014B01Rik | darkolivegreen |
| Hsp90b1 | deeppink1 |
| Gm15344 | mistyrose |
| BC030307 | darkolivegreen |
| Fabp3-ps1 | mistyrose |
| Nt5dc3 | darkgrey |
| Stab2 | coral1 |
| Pah | blue4 |
| Igf1 | blue3 |
| Nup37 | blue4 |
| Ccdc53 | mistyrose |
| Dram1 | blue4 |
| Gnptab | blue4 |
| Gm16235 | blue4 |
| Chpt1 | mistyrose |
| Sycp3 | firebrick2 |
| Arl1 | coral1 |
| Utp20 | mistyrose |
| Slc5a8 | blue4 |
| Gas2l3 | darkgrey |
| Nr1h4 | blue3 |
| Scyl2 | green4 |
| 1500026H17Rik | blue4 |
| Actr6 | mistyrose |
| Uhrf1bp1l | coral1 |
| Gm26180 | blue4 |
| Anks1b | blue4 |
| Apaf1 | blue4 |
| Ikbip | blue4 |
| Slc25a3 | antiquewhite2 |
| Gm24119 | orangered |
| Tmpo | blue4 |
| Nedd1 | antiquewhite2 |
| Gm24241 | blue4 |
| Cdk17 | blue4 |
| Mir1931 | coral1 |
| Elk3 | blue4 |
| Mir7688 | blue4 |
| Lta4h | blue3 |
| Ntn4 | blue4 |
| Metap2 | coral1 |
| Vezt | blue4 |
| Fgd6 | blue4 |
| Nr2c1 | blue4 |
| Ndufa12 | blue4 |
| Tmcc3 | brown1 |
| Mir7211 | darkgrey |
| Cep83os | mistyrose |
| Cep83 | blue4 |
| Cradd | darkolivegreen |
| Socs2 | darkgrey |
| Mrpl42 | darkgrey |
| Ube2n | blue4 |
| Nudt4 | mistyrose |
| Mir3058 | blue4 |
| 4732465J04Rik | darkolivegreen |
| Eea1 | blue4 |
| Btg1 | darkgrey |
| Dcn | darkgrey |
| Lum | blue4 |
| Atp2b1 | blue4 |
| Poc1b | blue4 |
| Galnt4 | blue4 |
| Dusp6 | coral1 |
| Kitl | blue4 |
| Gm22918 | blue4 |
| Tmtc3 | blue4 |
| Cep290 | blue4 |
| 4930430F08Rik | coral1 |
| Rassf9 | blue4 |
| Alx1 | blue4 |
| Gm17028 | blue3 |
| Slc6a15 | firebrick2 |
| Gm15662 | blue4 |
| Mettl25 | coral1 |
| Ccdc59 | blue4 |
| Acss3 | mistyrose |
| Lin7a | deeppink |
| Ppp1r12a | blue4 |
| Pawr | blue3 |
| Csrp2 | blue4 |
| Zdhhc17 | blue4 |
| Gm5428 | blue4 |
| Osbpl8 | firebrick2 |
| Bbs10 | blue3 |
| Nap1l1 | blue4 |
| Gm25117 | blue4 |
| Phlda1 | coral1 |
| Krr1 | coral1 |
| Glipr1 | honeydew |
| Atxn7l3b | blue4 |
| Tbc1d15 | green4 |
| Rab21 | blue3 |
| Tmem19 | blue4 |
| Thap2 | blue3 |
| Zfc3h1 | blue3 |
| Tspan8 | blue4 |
| Ptprb | blue4 |
| Cnot2 | coral1 |
| 5330438D12Rik | blue3 |
| Rab3ip | green4 |
| Gm10271 | blue3 |
| D630029K05Rik | mistyrose |
| Cct2 | blue4 |
| Frs2 | blue4 |
| Yeats4 | blue4 |
| Lyz2 | blue4 |
| Cpsf6 | blue4 |
| Cpm | blue4 |
| Mdm2 | darkgrey |
| Slc35e3 | blue4 |
| Nup107 | blue4 |
| Rap1b | green4 |
| Mdm1 | blue4 |
| Dyrk2 | deeppink |
| Cand1 | blue4 |
| Grip1 | blue4 |
| Helb | coral1 |
| Irak3 | honeydew |
| Tmbim4 | blue4 |
| Llph | blue4 |
| Msrb3 | blue4 |
| Lemd3 | darkgrey |
| Gns | coral1 |
| Rassf3 | mistyrose |
| Tbk1 | coral1 |
| Xpot | coral1 |
| BC048403 | blue4 |
| Srgap1 | blue4 |
| Tmem5 | coral1 |
| Avpr1a | orangered |
| Ppm1h | green4 |
| Mirlet7i | blue3 |
| Mon2 | blue3 |
| Usp15 | coral1 |
| Gm27920 | darkgrey |
| Slc16a7 | darkolivegreen |
| Lrig3 | orangered |
| Xrcc6bp1 | coral1 |
| Ctdsp2 | blue4 |
| Tsfm | blue3 |
| Mettl21b | mistyrose |
| Mettl1 | blue4 |
| Cyp27b1 | blue4 |
| 9-Mar | green4 |
| Cdk4 | blue4 |
| Tspan31 | deeppink |
| Os9 | blue4 |
| B4galnt1 | blue4 |
| Slc26a10 | darkolivegreen |
| Arhgef25 | blue4 |
| Dtx3 | blue3 |
| F420014N23Rik | blue3 |
| Pip4k2c | blue3 |
| Kif5a | blue3 |
| Dctn2 | blue4 |
| Mbd6 | blue3 |
| Gm20492 | blue3 |
| Gm4189 | blue4 |
| Ddit3 | darkgrey |
| Mars | blue4 |
| Arhgap9 | saddlebrown |
| R3hdm2 | green4 |
| Ndufa4l2 | green4 |
| Shmt2 | green4 |
| Nxph4 | blue4 |
| Lrp1 | blue4 |
| Stat6 | blue3 |
| Nab2 | mistyrose |
| Tmem194 | blue4 |
| Zbtb39 | blue4 |
| Gpr182 | blue4 |
| Rdh16 | blue4 |
| BC089597 | darkolivegreen |
| Prim1 | blue4 |
| Naca | coral1 |
| Ptges3 | blue4 |
| Atp5b | blue4 |
| Gm25206 | coral1 |
| Mir677 | darkolivegreen |
| Baz2a | blue3 |
| Rbms2 | blue4 |
| Spryd4 | blue4 |
| Timeless | blue4 |
| Stat2 | blue4 |
| Pan2 | blue3 |
| Cnpy2 | blue4 |
| Cs | blue3 |
| Coq10a | blue4 |
| Ankrd52 | blue4 |
| Gm17201 | blue3 |
| Slc39a5 | green4 |
| Nabp2 | blue3 |
| Gm25361 | blue4 |
| Rnf41 | firebrick2 |
| Gm26347 | blue3 |
| Smarcc2 | blue3 |
| Myl6 | blue4 |
| Myl6b | coral1 |
| A430046D13Rik | darkolivegreen |
| Esyt1 | blue4 |
| Gm29585 | blue4 |
| Zc3h10 | deeppink |
| Rpl41 | firebrick2 |
| Pa2g4 | blue4 |
| Erbb3 | mistyrose |
| Rps26 | blue4 |
| Ikzf4 | blue4 |
| Suox | blue4 |
| Rab5b | darkgrey |
| Cdk2 | blue4 |
| Dgka | blue3 |
| Wibg | darkgrey |
| Rpsa-ps2 | blue4 |
| Mmp19 | blue4 |
| Tmem198b | blue3 |
| Dnajc14 | blue3 |
| Ormdl2 | blue3 |
| Sarnp | blue4 |
| Gdf11 | blue3 |
| Cd63 | blue4 |
| Rdh5 | blue4 |
| Bloc1s1 | deeppink |
| Itga7 | blue4 |
| Mettl7b | blue3 |
| Gm9770 | antiquewhite2 |
| Pisd-ps1 | blue3 |
| Sfi1 | blue3 |
| Drg1 | blue4 |
| Eif4enif1 | blue4 |
| Patz1 | blue3 |
| Gm11944 | blue3 |
| Pik3ip1 | mistyrose |
| Limk2 | blue3 |
| Rnf185 | deeppink1 |
| 8430429K09Rik | blue4 |
| Gm11945 | mistyrose |
| Pla2g3 | antiquewhite2 |
| Inpp5j | green4 |
| Selm | blue4 |
| Smtn | blue4 |
| Tug1 | blue3 |
| Morc2a | blue4 |
| Osbp2 | blue4 |
| Dusp18 | blue3 |
| Slc35e4 | darkgrey |
| Tcn2 | blue4 |
| 4930556J24Rik | blue4 |
| Pes1 | blue4 |
| Gal3st1 | green4 |
| Sec14l3 | blue4 |
| Mtfp1 | green4 |
| Sec14l2 | blue4 |
| Rnf215 | blue3 |
| Mir3060 | blue3 |
| Ccdc157 | darkolivegreen |
| Sf3a1 | deeppink |
| Tbc1d10a | darkgrey |
| Gatsl3 | blue4 |
| Lif | darkgrey |
| Mtmr3 | orangered |
| Ascc2 | blue3 |
| Uqcr10 | blue4 |
| Zmat5 | brown1 |
| Nf2 | blue3 |
| Nipsnap1 | blue4 |
| Thoc5 | blue4 |
| Ap1b1 | deeppink |
| Gm24958 | blue4 |
| Gas2l1 | blue4 |
| Rasl10a | mistyrose |
| Ewsr1 | blue3 |
| Rhbdd3 | green4 |
| Emid1 | deeppink |
| Kremen1 | mistyrose |
| Znrf3 | blue4 |
| Gm11962 | blue3 |
| Gm11963 | mistyrose |
| Xbp1 | mistyrose |
| Ccdc117 | mistyrose |
| Gm11964 | blue4 |
| Mrps24 | deeppink |
| Urgcp | darkgrey |
| 2210015D19Rik | blue4 |
| Dbnl | blue4 |
| Pgam2 | blue4 |
| Gm11966 | blue4 |
| Polm | blue3 |
| Aebp1 | blue4 |
| Pold2 | blue4 |
| Myl7 | lightsteelblue |
| Ykt6 | blue4 |
| Camk2b | blue4 |
| Nudcd3 | deeppink |
| Rps15a-ps6 | blue4 |
| Ddx56 | blue4 |
| Tmed4 | blue4 |
| n-R5s67 | blue3 |
| Ogdh | coral1 |
| Zmiz2 | deeppink |
| Mir7651 | blue3 |
| Ppia | blue4 |
| H2afv | blue4 |
| Purb | blue4 |
| Myo1g | honeydew |
| Gm11974 | darkgrey |
| Ccm2 | blue4 |
| Nacad | antiquewhite2 |
| Tbrg4 | blue3 |
| Ramp3 | blue3 |
| Adcy1 | darkolivegreen |
| Igfbp1 | blue3 |
| Igfbp3 | blue3 |
| Tns3 | blue4 |
| Hus1 | coral1 |
| Sun3 | blue4 |
| Gm11992 | blue4 |
| Upp1 | blue4 |
| Abca13 | darkolivegreen |
| Ikzf1 | blue3 |
| Fignl1 | blue4 |
| Ddc | blue4 |
| Grb10 | blue4 |
| Gm24036 | darkolivegreen |
| Cobl | blue4 |
| Gm12002 | blue4 |
| Gm12009 | deeppink |
| Vstm2a | darkolivegreen |
| Sec61g | darkolivegreen |
| Egfr | darkgrey |
| Plek | honeydew |
| Ppp3r1 | blue4 |
| Wdr92 | coral1 |
| Pno1 | blue4 |
| C1d | mistyrose |
| Etaa1 | coral1 |
| Etaa1os | green4 |
| Meis1 | blue4 |
| Glns-ps1 | mistyrose |
| Spred2 | blue4 |
| Actr2 | coral1 |
| Rab1 | coral1 |
| Cep68 | blue3 |
| Slc1a4 | blue4 |
| Sertad2 | darkgrey |
| Aftph | deeppink |
| Lgalsl | blue4 |
| Peli1 | blue3 |
| Vps54 | firebrick2 |
| Ugp2 | deeppink |
| Mdh1 | mistyrose |
| Gm22990 | blue4 |
| Wdpcp | mistyrose |
| Ehbp1 | blue4 |
| Tmem17 | blue3 |
| 9130230N09Rik | darkolivegreen |
| Commd1 | blue4 |
| Zrsr1 | blue4 |
| Cct4 | blue4 |
| Fam161a | blue3 |
| Xpo1 | blue4 |
| Usp34 | blue4 |
| Gm22753 | darkgrey |
| Ahsa2 | blue3 |
| 0610010F05Rik | blue4 |
| Pex13 | green4 |
| Pus10 | coral1 |
| Gm20645 | darkgrey |
| Rel | darkgrey |
| Papolg | coral1 |
| Fancl | blue4 |
| Vrk2 | coral1 |
| Gm12070 | blue4 |
| Efemp1 | blue4 |
| Pnpt1 | blue4 |
| A630052C17Rik | coral1 |
| Smek2 | coral1 |
| Cfap36 | green4 |
| Ccdc88a | blue4 |
| Prorsd1 | green4 |
| Mtif2 | blue4 |
| Rps27a | blue4 |
| Rtn4 | blue4 |
| Eml6 | blue3 |
| Sptbn1 | blue4 |
| Gm29237 | blue4 |
| Acyp2 | blue4 |
| Psme4 | blue4 |
| Erlec1 | mistyrose |
| Asb3 | coral1 |
| Chac2 | blue4 |
| Stc2 | blue3 |
| Bod1 | blue4 |
| D630024D03Rik | blue4 |
| Cpeb4 | deeppink |
| Snrnp25 | deeppink |
| Rhbdf1 | darkgrey |
| Nprl3 | blue3 |
| Mpg | blue3 |
| Hba-a1 | blue4 |
| Hba-a2 | coral1 |
| Sh3pxd2b | darkgrey |
| Ubtd2 | blue4 |
| Fbxw11 | blue4 |
| Gm12112 | blue4 |
| Fgf18 | blue4 |
| Npm1 | blue4 |
| 4930469K13Rik | blue4 |
| Gm12121 | blue3 |
| Foxi1 | mistyrose |
| Dock2 | honeydew |
| Spdl1 | blue4 |
| Slit3 | blue4 |
| Pank3 | mistyrose |
| Rars | blue4 |
| Wwc1 | blue3 |
| Mat2b | mistyrose |
| Hmmr | darkolivegreen |
| Nudcd2 | blue4 |
| Ccng1 | coral1 |
| Gm12141 | blue4 |
| Atp10b | blue4 |
| Pttg1 | blue4 |
| Slu7 | coral1 |
| C1qtnf2 | coral1 |
| Pwwp2a | blue3 |
| Ttc1 | green4 |
| Adra1b | blue4 |
| Ublcp1 | coral1 |
| Gm22121 | blue3 |
| Rnf145 | mistyrose |
| Ebf1 | blue4 |
| Gm12164 | blue4 |
| Clint1 | blue4 |
| Lsm11 | blue4 |
| Thg1l | blue4 |
| Gm12166 | blue3 |
| Adam19 | blue4 |
| Cyfip2 | darkolivegreen |
| Fndc9 | blue4 |
| Med7 | blue4 |
| Gm12168 | blue4 |
| Havcr2 | darkgrey |
| Timd2 | blue4 |
| Gm12174 | blue4 |
| Havcr1 | blue4 |
| Gm12183 | blue4 |
| Gnb2l1 | blue4 |
| Trim41 | blue4 |
| Trim7 | blue4 |
| Gm16170 | blue4 |
| Irgm1 | saddlebrown |
| Psme2b | saddlebrown |
| Tgtp1 | saddlebrown |
| 9930111J21Rik2 | coral1 |
| Tgtp2 | saddlebrown |
| Ifi47 | saddlebrown |
| Btnl9 | lightsteelblue |
| Zfp62 | blue4 |
| Mgat1 | coral1 |
| Olfr1393 | blue3 |
| Flt4 | blue4 |
| Cnot6 | blue4 |
| Gm12191 | blue4 |
| Gfpt2 | darkgrey |
| Mapk9 | blue4 |
| Rnf130 | deeppink |
| Tbc1d9b | green4 |
| Gm12195 | blue4 |
| 3010026O09Rik | mistyrose |
| Sqstm1 | blue3 |
| Mgat4b | blue4 |
| Ltc4s | blue4 |
| Gm26542 | blue4 |
| Maml1 | darkgrey |
| Canx | blue4 |
| Hnrnph1 | deeppink1 |
| Rufy1 | blue3 |
| Adamts2 | blue4 |
| Zfp354c | coral1 |
| Zfp454 | blue3 |
| Zfp2 | coral1 |
| Zfp354b | firebrick2 |
| Zfp354a | blue3 |
| BC049762 | blue4 |
| Clk4 | deeppink1 |
| Gm25082 | deeppink1 |
| Col23a1 | blue4 |
| Phykpl | deeppink1 |
| Hnrnpab | blue4 |
| Nhp2 | blue4 |
| Rmnd5b | blue3 |
| N4bp3 | deeppink |
| D930048N14Rik | blue3 |
| 0610009B22Rik | darkgrey |
| Sec24a | darkgrey |
| Gm25291 | darkgrey |
| Sar1b | mistyrose |
| Jade2 | darkgrey |
| Cdkn2aipnl | blue4 |
| Ube2b | blue3 |
| Cdkl3 | blue3 |
| Ppp2ca | brown1 |
| Olfr1372-ps1 | blue4 |
| Skp1a | mistyrose |
| Gm12207 | darkgrey |
| Tcf7 | blue4 |
| Vdac1 | blue4 |
| 9530068E07Rik | blue4 |
| Gm12208 | blue3 |
| Hspa4 | blue4 |
| Zcchc10 | blue4 |
| Aff4 | brown1 |
| Uqcrq | blue4 |
| Shroom1 | blue4 |
| Sowaha | darkolivegreen |
| 8-Sep | blue4 |
| Kif3a | blue3 |
| Il4 | blue3 |
| Rad50 | blue4 |
| Irf1 | saddlebrown |
| Gm12216 | saddlebrown |
| Slc22a5 | blue4 |
| Slc22a21 | darkolivegreen |
| Slc22a4 | blue4 |
| Pdlim4 | blue4 |
| P4ha2 | mistyrose |
| Fnip1 | deeppink |
| Rapgef6 | blue4 |
| Cdc42se2 | blue4 |
| Lyrm7 | blue3 |
| Hint1 | blue4 |
| Gpx3 | indianred2 |
| Tnip1 | mistyrose |
| Anxa6 | blue4 |
| Ccdc69 | lightcoral |
| Gm2a | darkolivegreen |
| Gm12231 | orangered |
| Slc36a1 | green4 |
| Sparc | blue4 |
| Atox1 | blue4 |
| G3bp1 | blue4 |
| Fam114a2 | blue3 |
| Mfap3 | blue4 |
| Galnt10 | blue4 |
| 2010001A14Rik | blue4 |
| Sap30l | blue4 |
| Larp1 | blue4 |
| Cnot8 | coral1 |
| Gemin5 | blue4 |
| Mrpl22 | blue4 |
| Igtp | saddlebrown |
| Zfp692 | blue3 |
| Zfp672 | blue3 |
| Sh3bp5l | darkgrey |
| Gm12251 | blue4 |
| Gm12254 | blue4 |
| Gm12258 | blue3 |
| 2810021J22Rik | blue3 |
| Zfp39 | firebrick2 |
| Rnf187 | blue4 |
| Hist3h2ba | coral1 |
| Hist3h2a | green4 |
| Trim11 | blue3 |
| Iba57 | blue4 |
| Gjc2 | blue3 |
| Guk1 | blue4 |
| 2610507I01Rik | blue3 |
| Mrpl55 | blue3 |
| 2310033P09Rik | mistyrose |
| Arf1 | blue4 |
| Wnt9a | darkolivegreen |
| Snap47 | coral1 |
| Jmjd4 | darkgrey |
| Zfp867 | blue4 |
| Zkscan17 | blue4 |
| 4933439C10Rik | blue3 |
| Mprip | blue3 |
| Gm12264 | blue3 |
| Flcn | darkolivegreen |
| Cops3 | blue4 |
| Nt5m | blue3 |
| 1810063I02Rik | blue3 |
| Med9 | blue4 |
| Rasd1 | coral1 |
| Pemt | blue4 |
| Rai1 | blue3 |
| 4930412M03Rik | blue4 |
| Srebf1 | blue3 |
| Tom1l2 | coral1 |
| Atpaf2 | deeppink |
| Gid4 | firebrick2 |
| Drg2 | deeppink |
| Alkbh5 | blue3 |
| Llgl1 | blue4 |
| Flii | blue3 |
| Mief2 | green4 |
| Mir5100 | blue3 |
| Gm23341 | coral1 |
| Top3a | blue4 |
| Smcr8 | darkgrey |
| Shmt1 | blue3 |
| Dhrs7b | coral1 |
| Tmem11 | darkgrey |
| Natd1 | darkgrey |
| Map2k3os | blue4 |
| Map2k3 | darkgrey |
| Kcnj12 | blue4 |
| Usp22 | darkgrey |
| Aldh3a2 | mistyrose |
| Slc47a1 | blue4 |
| Mfap4 | mistyrose |
| Mapk7 | blue4 |
| B9d1 | deeppink |
| Epn2 | blue4 |
| Grap | darkgrey |
| Slc5a10 | blue4 |
| Fam83g | darkgrey |
| Prpsap2 | firebrick2 |
| Ulk2 | blue3 |
| A530017D24Rik | firebrick2 |
| Akap10 | blue4 |
| Specc1 | blue4 |
| Adora2b | blue4 |
| Zswim7 | blue3 |
| Ttc19 | blue4 |
| Ncor1 | blue4 |
| Gm12276 | deeppink1 |
| Pigl | blue3 |
| Cenpv | blue4 |
| Gm12279 | firebrick2 |
| Ubb | darkgrey |
| Trpv2 | blue4 |
| 2410006H16Rik | darkgrey |
| Snord49a | blue4 |
| Snord65 | mistyrose |
| Lrrc75a | blue4 |
| Mmgt2 | blue3 |
| Zfp287 | blue4 |
| Trim16 | darkgrey |
| Tvp23b | mistyrose |
| Pmp22 | blue4 |
| Hs3st3b1 | green4 |
| Cox10 | blue4 |
| 9630013K17Rik | blue3 |
| 2810001G20Rik | blue3 |
| Elac2 | blue3 |
| Arhgap44 | blue3 |
| Map2k4 | coral1 |
| Zkscan6 | darkgrey |
| 9130409J20Rik | blue3 |
| Tmem220 | mistyrose |
| Adprm | mistyrose |
| Sco1 | blue3 |
| Gas7 | blue4 |
| Usp43 | blue4 |
| Stx8 | blue4 |
| Ntn1 | coral1 |
| Pik3r5 | honeydew |
| Pik3r6 | blue3 |
| Myh10 | blue4 |
| Ndel1 | blue4 |
| Rpl26 | blue4 |
| Odf4 | darkolivegreen |
| Arhgef15 | blue3 |
| Slc25a35 | green4 |
| Pfas | blue3 |
| Ctc1 | blue3 |
| Aurkb | blue4 |
| 2310047M10Rik | coral1 |
| Tmem107 | blue3 |
| Snord118 | darkgrey |
| Vamp2 | blue3 |
| Per1 | darkgrey |
| Cntrob | blue3 |
| Trappc1 | blue4 |
| Chd3os | blue4 |
| Chd3 | blue4 |
| Gm22442 | blue4 |
| Cyb5d1 | blue3 |
| Naa38 | coral1 |
| Tmem88 | blue4 |
| Kdm6b | darkgrey |
| Kdm6bos | darkgrey |
| Wrap53 | blue3 |
| Trp53 | blue4 |
| Atp1b2 | deeppink |
| Sat2 | mistyrose |
| Fxr2 | blue3 |
| Mir467f | blue3 |
| Mpdu1 | firebrick2 |
| Mir1934 | blue3 |
| Cd68 | blue4 |
| Eif4a1 | blue4 |
| Gm25835 | darkgrey |
| Senp3 | blue4 |
| Tnfsf12 | blue4 |
| Polr2a | deeppink |
| Zbtb4 | blue4 |
| Chrnb1 | blue4 |
| Fgf11 | blue3 |
| Tmem102 | deeppink |
| Nlgn2 | blue4 |
| Tmem256 | blue4 |
| Plscr3 | blue4 |
| Tnk1 | blue3 |
| Kctd11 | blue4 |
| Neurl4 | green4 |
| Gps2 | blue4 |
| Eif5a | blue4 |
| Ybx2 | darkolivegreen |
| Slc2a4 | blue4 |
| Cldn7 | blue4 |
| Elp5 | blue4 |
| Ctdnep1 | blue3 |
| Gabarap | blue4 |
| Phf23 | deeppink |
| Dvl2 | blue3 |
| Acadvl | mistyrose |
| Dlg4 | blue3 |
| Mgl2 | darkgrey |
| Slc16a11 | blue4 |
| Slc16a13 | brown1 |
| Bcl6b | darkgrey |
| Alox12 | antiquewhite2 |
| Pelp1 | blue4 |
| Arrb2 | blue4 |
| Med11 | blue4 |
| Cxcl16 | saddlebrown |
| Zmynd15 | blue3 |
| Psmb6 | blue4 |
| Pld2 | blue4 |
| Mink1 | blue3 |
| Slc25a11 | blue4 |
| Rnf167 | blue4 |
| Pfn1 | blue4 |
| Gm12319 | green4 |
| Eno3 | darkolivegreen |
| Spag7 | blue4 |
| Camta2 | green4 |
| Inca1 | blue4 |
| Kif1c | deeppink |
| Rabep1 | blue4 |
| Nup88 | blue4 |
| Rpain | blue3 |
| C1qbp | blue3 |
| Dhx33 | blue4 |
| Derl2 | deeppink |
| Mis12 | blue4 |
| Gm12326 | darkolivegreen |
| Wscd1 | green4 |
| Pitpnm3 | green4 |
| 4933427D14Rik | blue3 |
| Txndc17 | coral1 |
| Med31 | darkgrey |
| Slc13a5 | blue4 |
| Xaf1 | saddlebrown |
| Mir6338 | blue4 |
| Smtnl2 | blue4 |
| Ggt6 | blue3 |
| Mybbp1a | blue4 |
| Spns2 | blue4 |
| Spns3 | blue3 |
| Ube2g1 | blue4 |
| Ankfy1 | blue4 |
| Cyb5d2 | darkolivegreen |
| Zzef1 | blue4 |
| Atp2a3 | coral1 |
| P2rx1 | green4 |
| 1200014J11Rik | blue4 |
| Gsg2 | blue4 |
| P2rx5 | blue4 |
| Emc6 | darkgrey |
| Tax1bp3 | blue4 |
| Ctns | blue3 |
| Shpk | blue4 |
| Aspa | blue3 |
| Rap1gap2 | blue4 |
| Cluh | green4 |
| Pafah1b1 | coral1 |
| Mettl16 | blue4 |
| Mnt | darkgrey |
| Sgsm2 | deeppink |
| Tsr1 | blue4 |
| Snord91a | darkolivegreen |
| Gm22771 | darkolivegreen |
| Srr | darkolivegreen |
| Smg6 | blue3 |
| Hic1 | blue4 |
| Mir212 | coral1 |
| Mir132 | darkgrey |
| Ovca2 | green4 |
| Dph1 | blue4 |
| Rtn4rl1 | blue3 |
| Rpa1 | blue4 |
| Smyd4 | blue3 |
| Serpinf1 | lightcoral |
| Serpinf2 | blue4 |
| Wdr81 | green4 |
| Mir22hg | darkgrey |
| Tlcd2 | blue3 |
| Prpf8 | blue3 |
| Rilp | green4 |
| Scarf1 | blue3 |
| Slc43a2 | green4 |
| Pitpna | blue4 |
| Gm12338 | antiquewhite2 |
| Inpp5k | mistyrose |
| Myo1c | blue4 |
| Crk | coral1 |
| Ywhae | blue4 |
| Rph3al | deeppink |
| Fam101b | blue4 |
| Vps53 | blue3 |
| Glod4 | coral1 |
| Fam57a | blue4 |
| Rnmtl1 | blue4 |
| Nxn | blue4 |
| Timm22 | deeppink1 |
| Abr | blue3 |
| Rpl36-ps2 | blue4 |
| Tusc5 | lightcoral |
| Gosr1 | blue4 |
| Cpd | blue4 |
| Tmigd1 | mistyrose |
| Blmh | blue3 |
| Slc6a4 | blue3 |
| Gm12343 | blue3 |
| Ccdc55 | brown1 |
| Gm22772 | blue4 |
| Ssh2 | blue4 |
| Gm12346 | darkgrey |
| Coro6 | blue4 |
| Ankrd13b | mistyrose |
| Git1 | mistyrose |
| Trp53i13 | blue3 |
| Abhd15 | mistyrose |
| Taok1 | blue4 |
| Nufip2 | blue4 |
| Myo18a | blue3 |
| Pipox | mistyrose |
| Phf12 | blue3 |
| Dhrs13 | blue4 |
| Flot2 | blue4 |
| Eral1 | brown1 |
| Fam222b | mistyrose |
| Gm12571 | blue4 |
| Traf4 | coral1 |
| Nek8 | blue3 |
| Tlcd1 | darkolivegreen |
| Rpl23a | blue4 |
| Snord42b | green4 |
| Rab34 | blue4 |
| Proca1 | antiquewhite2 |
| Supt6 | darkgrey |
| Sdf2 | lightsteelblue |
| 2610507B11Rik | blue4 |
| Spag5 | blue4 |
| Aldoc | blue4 |
| Pigs | deeppink |
| Unc119 | blue4 |
| Slc13a2 | green4 |
| Slc46a1 | blue4 |
| Tmem199 | blue4 |
| Poldip2 | blue4 |
| Tnfaip1 | blue4 |
| Ift20 | blue4 |
| Tmem97 | blue4 |
| Nlk | blue4 |
| Fam58b | blue3 |
| Lyrm9 | blue3 |
| Nos2 | coral1 |
| Lgals9 | blue4 |
| Ksr1 | blue4 |
| Wsb1 | blue4 |
| Nf1 | blue4 |
| Rab11fip4 | darkolivegreen |
| Mir365-2 | blue3 |
| 9130204K15Rik | blue4 |
| Utp6 | blue4 |
| Suz12 | blue4 |
| Crlf3 | blue4 |
| Atad5 | blue4 |
| Tefm | coral1 |
| Adap2 | blue4 |
| Adap2os | blue4 |
| Rnf135 | blue3 |
| Rhot1 | coral1 |
| Rhbdl3 | blue4 |
| 5730455P16Rik | coral1 |
| Zfp207 | blue4 |
| Psmd11 | blue4 |
| Myo1d | blue3 |
| Tmem98 | blue4 |
| Ccl2 | darkgrey |
| Ccl7 | blue4 |
| Ccl11 | darkgrey |
| Cct6b | blue4 |
| Zfp830 | blue3 |
| Gm11423 | darkolivegreen |
| Lig3 | blue4 |
| Rffl | mistyrose |
| Rad51d | darkgrey |
| Nle1 | blue4 |
| Unc45bos | blue4 |
| Slfn5 | blue4 |
| Slfn8 | blue4 |
| Slfn2 | honeydew |
| Slfn3 | blue4 |
| AA465934 | blue3 |
| Snord7 | blue3 |
| Pex12 | mistyrose |
| Ap2b1 | blue4 |
| 1700020L24Rik | blue3 |
| Mmp28 | blue4 |
| Taf15 | blue4 |
| Ccl5 | saddlebrown |
| Ccl9 | blue4 |
| E230016K23Rik | blue3 |
| Ccl6 | blue4 |
| Ccl3 | mistyrose |
| Ccl4 | coral1 |
| Wfdc17 | darkgrey |
| Heatr6 | blue3 |
| Gm11434 | darkolivegreen |
| Gm12576 | blue3 |
| Hnf1b | green4 |
| Ddx52 | coral1 |
| Synrg | blue4 |
| Dusp14 | darkgrey |
| Tada2a | blue4 |
| Acaca | lightcoral |
| Aatf | blue4 |
| Lhx1 | darkolivegreen |
| Lhx1os | blue4 |
| Mrm1 | blue4 |
| Dhrs11 | green4 |
| Ggnbp2 | brown1 |
| Pigw | blue4 |
| Myo19 | blue3 |
| Gm23564 | blue3 |
| Znhit3 | green4 |
| Car4 | blue4 |
| Usp32 | blue4 |
| Rpl13-ps1 | blue3 |
| Appbp2 | blue4 |
| Appbp2os | blue3 |
| Ppm1d | mistyrose |
| Bcas3 | blue4 |
| Tbx2 | blue3 |
| Brip1os | blue3 |
| Ints2 | mistyrose |
| Med13 | deeppink1 |
| Rnft1 | mistyrose |
| Rps6kb1 | blue4 |
| Tubd1 | blue3 |
| Vmp1 | blue4 |
| Gm11478 | blue4 |
| Ptrh2 | blue4 |
| Cltc | blue4 |
| Dhx40 | deeppink |
| Trim37 | blue4 |
| 4-Sep | blue3 |
| Mtmr4 | green4 |
| Supt4a | blue3 |
| Mir142hg | blue4 |
| Mks1 | blue3 |
| Dynll2 | darkolivegreen |
| Srsf1 | blue3 |
| Vezf1 | brown1 |
| Cuedc1 | blue3 |
| Mrps23 | blue4 |
| Msi2 | coral1 |
| Mir378b | lightsteelblue |
| C030037D09Rik | green4 |
| Akap1 | deeppink1 |
| Scpep1 | coral1 |
| Trim25 | coral1 |
| Pctp | darkolivegreen |
| Tmem100 | blue3 |
| Mmd | blue4 |
| Hlf | darkolivegreen |
| Stxbp4 | darkolivegreen |
| Cox11 | brown1 |
| Tom1l1 | blue4 |
| Utp18 | blue4 |
| Mbtd1 | green4 |
| Nme2 | mistyrose |
| Nme1 | darkolivegreen |
| Spag9 | deeppink |
| B230206L02Rik | darkolivegreen |
| Tob1 | coral1 |
| Luc7l3 | blue3 |
| Ankrd40 | green4 |
| Epn3 | deeppink |
| Mycbpap | green4 |
| Rsad1 | blue3 |
| Acsf2 | blue4 |
| Lrrc59 | blue4 |
| Mrpl27 | blue3 |
| Xylt2 | green4 |
| Col1a1 | blue4 |
| Ppp1r9b | blue4 |
| Samd14 | blue4 |
| Pdk2 | blue4 |
| Itga3 | blue4 |
| Kat7 | darkgrey |
| Gm11520 | blue4 |
| Fam117a | blue3 |
| Slc35b1 | darkgrey |
| Spop | coral1 |
| Nxph3 | deeppink |
| Phb | blue4 |
| Zfp652 | blue4 |
| Gngt2 | blue4 |
| Igf2bp1 | blue3 |
| Snf8 | deeppink |
| Ube2z | blue4 |
| Atp5g1 | blue4 |
| Gm11539 | blue4 |
| Gm53 | blue3 |
| Mir196a-1 | blue3 |
| Hoxb9 | blue3 |
| Hoxb8 | blue3 |
| Hoxb7 | blue4 |
| Hoxb5os | blue3 |
| Hoxb6 | blue3 |
| Hoxb5 | blue3 |
| Mir10a | blue3 |
| Hoxb4 | blue3 |
| Hoxb3 | blue4 |
| Gm11536 | blue3 |
| Hoxb3os | blue4 |
| Hoxb2 | blue3 |
| Snx11 | blue4 |
| Cbx1 | blue3 |
| Gm11517 | blue3 |
| Nfe2l1 | coral1 |
| Copz2 | green4 |
| Cdk5rap3 | blue4 |
| D030028A08Rik | blue3 |
| Prr15l | brown1 |
| Pnpo | blue4 |
| Sp2 | darkgrey |
| Scrn2 | coral1 |
| Lrrc46 | blue3 |
| Mrpl10 | darkgrey |
| Osbpl7 | blue4 |
| Tbkbp1 | blue4 |
| Kpnb1 | blue4 |
| Npepps | blue3 |
| Mrpl45 | blue4 |
| Socs7 | blue4 |
| Arhgap23 | blue4 |
| Srcin1 | green4 |
| Gm11611 | green4 |
| Gm11612 | green4 |
| 2410003L11Rik | darkolivegreen |
| E130012A19Rik | blue3 |
| Mllt6 | blue3 |
| Cisd3 | green4 |
| Pcgf2 | blue3 |
| Psmb3 | blue4 |
| Pip4k2b | blue4 |
| Mir8102 | blue4 |
| Cwc25 | darkgrey |
| Atp5l2-ps | blue4 |
| Rpl23 | blue4 |
| Gm27326 | blue4 |
| Snora21 | darkgrey |
| Lasp1 | blue4 |
| Cacnb1 | green4 |
| Rpl19 | blue4 |
| Fbxl20 | mistyrose |
| Med1 | blue4 |
| Cdk12 | blue4 |
| Mir5119 | deeppink1 |
| Ppp1r1b | green4 |
| Stard3 | green4 |
| Pgap3 | green4 |
| Erbb2 | blue3 |
| Gm25048 | darkolivegreen |
| Mien1 | coral1 |
| Grb7 | blue4 |
| Ormdl3 | blue4 |
| Psmd3 | mistyrose |
| Gm12356 | blue4 |
| Med24 | blue3 |
| Thra | blue4 |
| Nr1d1 | darkgrey |
| Msl1 | blue3 |
| Casc3 | blue4 |
| Wipf2 | mistyrose |
| Rara | mistyrose |
| Top2a | blue4 |
| Igfbp4 | blue4 |
| Smarce1 | blue4 |
| Krt10 | lightcoral |
| Krt20 | blue4 |
| Krt23 | darkgrey |
| Gm11560 | blue4 |
| Krt15 | blue4 |
| Krt19 | blue4 |
| Krt14 | blue4 |
| Gm11598 | blue4 |
| Eif1 | darkgrey |
| Hap1 | blue4 |
| Gm10039 | blue4 |
| Jup | blue4 |
| Leprel4 | blue4 |
| Fkbp10 | blue4 |
| Nt5c3b | blue3 |
| Klhl11 | deeppink |
| Acly | antiquewhite2 |
| Ttc25 | mistyrose |
| Cnp | blue4 |
| Dnajc7 | blue4 |
| Nkiras2 | deeppink |
| Dhx58 | honeydew |
| Kat2a | blue3 |
| Rab5c | blue4 |
| Ghdc | green4 |
| Stat5b | darkgrey |
| Stat5a | blue4 |
| Stat3 | darkgrey |
| Ptrf | blue4 |
| Atp6v0a1 | mistyrose |
| Naglu | green4 |
| Coasy | blue4 |
| Mlx | blue4 |
| Psmc3ip | blue4 |
| Fam134c | deeppink1 |
| Tubg1 | blue3 |
| Tubg2 | blue4 |
| Plekhh3 | blue3 |
| Cntnap1 | blue3 |
| Ezh1 | blue3 |
| Ramp2 | blue4 |
| Wnk4 | green4 |
| Gm11615 | blue3 |
| Coa3 | blue4 |
| Becn1 | blue4 |
| Psme3 | blue4 |
| Aoc2 | blue4 |
| Aoc3 | lightcoral |
| Gm11618 | blue4 |
| G6pc | mistyrose |
| Rundc1 | green4 |
| Rpl27 | blue4 |
| Ifi35 | blue4 |
| Vat1 | blue4 |
| Rnd2 | blue4 |
| Nbr1 | mistyrose |
| Tmem106a | blue4 |
| Rdm1 | blue4 |
| Gm23849 | mistyrose |
| Arl4d | coral1 |
| Dhx8 | blue4 |
| Etv4 | blue4 |
| Meox1 | deeppink1 |
| Dusp3 | blue4 |
| Mpp3 | darkgrey |
| Cd300lg | blue3 |
| Mpp2 | deeppink |
| Gm11585 | blue4 |
| Tmem101 | blue3 |
| Lsm12 | mistyrose |
| G6pc3 | deeppink |
| Hdac5 | blue4 |
| Tmub2 | green4 |
| Atxn7l3 | blue3 |
| Ubtf | blue3 |
| Slc4a1 | darkolivegreen |
| Rundc3a | blue4 |
| Slc25a39 | indianred2 |
| Grn | blue4 |
| Fam171a2 | blue4 |
| Itga2b | blue4 |
| Gpatch8 | blue4 |
| Fzd2 | blue4 |
| Ccdc43 | blue4 |
| Adam11 | blue4 |
| Gjc1 | blue4 |
| Higd1b | blue4 |
| Eftud2 | blue4 |
| Dcakd | blue3 |
| Nmt1 | coral1 |
| Plcd3 | blue3 |
| Acbd4 | blue4 |
| Hexim1 | blue4 |
| Hexim2 | mistyrose |
| Fmnl1 | green4 |
| Gm20511 | darkolivegreen |
| Map3k14 | darkgrey |
| Arhgap27 | blue3 |
| Plekhm1 | blue4 |
| Gosr2 | darkolivegreen |
| C130046K22Rik | green4 |
| Gm11642 | blue3 |
| Wnt9b | darkolivegreen |
| Nsf | blue4 |
| Arf2 | blue4 |
| Mapt | green4 |
| Kansl1 | deeppink1 |
| Cdc27 | blue4 |
| Itgb3 | blue4 |
| Mettl2 | blue4 |
| Tlk2 | brown1 |
| Mrc2 | blue4 |
| 10-Mar | blue4 |
| Tanc2 | blue4 |
| Cyb561 | blue4 |
| RP23-186E14.5 | blue4 |
| Ace | blue4 |
| Dcaf7 | blue3 |
| Taco1 | darkgrey |
| Map3k3 | darkolivegreen |
| Limd2 | deeppink1 |
| Strada | blue3 |
| Ccdc47 | coral1 |
| Ddx42 | coral1 |
| Ftsj3 | blue4 |
| Psmc5 | blue4 |
| Gm23645 | blue3 |
| Smarcd2 | blue4 |
| Icam2 | deeppink |
| Ern1 | blue3 |
| Snord104 | blue4 |
| Gm22711 | blue4 |
| Tex2 | darkolivegreen |
| Pecam1 | blue4 |
| Polg2 | blue4 |
| Ddx5 | deeppink1 |
| Cep95 | blue3 |
| Gm11706 | blue3 |
| Smurf2 | indianred2 |
| Gm11707 | darkgrey |
| Kpna2 | blue4 |
| Gm11705 | blue4 |
| 1810010H24Rik | blue3 |
| Bptf | blue4 |
| Nol11 | blue4 |
| Pitpnc1 | blue3 |
| Gm11714 | blue4 |
| Psmd12 | coral1 |
| Helz | blue3 |
| Prkca | blue4 |
| Apoh | mistyrose |
| Gna13 | blue4 |
| Amz2 | blue3 |
| Slc16a6 | blue4 |
| Gm25540 | blue4 |
| Arsg | blue3 |
| Gm22378 | blue4 |
| Wipi1 | mistyrose |
| Prkar1a | blue4 |
| Fam20a | blue4 |
| Abca8a | deeppink |
| Abca9 | firebrick2 |
| Abca5 | blue4 |
| Map2k6 | blue4 |
| Kcnj16 | mistyrose |
| Kcnj2 | blue4 |
| Sox9 | blue4 |
| 2610035D17Rik | blue4 |
| Slc39a11 | blue4 |
| Cog1 | blue3 |
| Fam104a | blue4 |
| D11Wsu47e | blue4 |
| Cpsf4l | blue3 |
| Cdc42ep4 | darkolivegreen |
| Rpl38 | blue4 |
| Ttyh2 | mistyrose |
| Gprc5c | blue4 |
| AF251705 | honeydew |
| Cd300lf | honeydew |
| Slc9a3r1 | coral1 |
| Nat9 | darkolivegreen |
| Tmem104 | green4 |
| Fdxr | green4 |
| Fads6 | darkolivegreen |
| Hid1 | blue4 |
| Cdr2l | blue4 |
| Ict1 | firebrick2 |
| Ict1os | blue4 |
| Atp5h | blue4 |
| Kctd2 | darkolivegreen |
| Gm11695 | green4 |
| Slc16a5 | blue4 |
| Armc7 | blue4 |
| Nt5c | blue4 |
| Hn1 | blue4 |
| Sumo2 | blue4 |
| Nup85 | blue4 |
| Gga3 | blue3 |
| Gm25364 | blue3 |
| Mrps7 | indianred2 |
| Mif4gd | green4 |
| Slc25a19 | green4 |
| Grb2 | blue4 |
| Gm11702 | blue3 |
| 2310067B10Rik | green4 |
| Caskin2 | blue3 |
| Tsen54 | blue3 |
| Llgl2 | blue4 |
| Myo15b | green4 |
| Recql5 | blue3 |
| Smim5 | blue4 |
| Smim6 | blue3 |
| Sap30bp | blue3 |
| Itgb4 | blue4 |
| Galk1 | blue4 |
| H3f3b | darkgrey |
| Unk | mistyrose |
| Unc13d | blue4 |
| Wbp2 | green4 |
| Trim47 | darkgrey |
| Trim65 | blue4 |
| Mrpl38 | blue4 |
| Fbf1 | blue3 |
| Gm26413 | darkgrey |
| Acox1 | blue4 |
| Ten1 | blue4 |
| Evpl | green4 |
| Srp68 | blue4 |
| Exoc7 | blue3 |
| Foxj1 | darkgrey |
| Ubald2 | blue3 |
| Gm11739 | blue3 |
| Prpsap1 | green4 |
| Gm29292 | darkgrey |
| Sphk1 | darkgrey |
| Ube2o | green4 |
| Rhbdf2 | darkgrey |
| Cygb | blue4 |
| 1810032O08Rik | darkgrey |
| St6galnac2 | darkolivegreen |
| Mxra7 | blue4 |
| Jmjd6 | darkgrey |
| Mettl23 | blue4 |
| Srsf2 | blue3 |
| Mfsd11 | green4 |
| 2810008D09Rik | blue3 |
| Sec14l1 | darkolivegreen |
| 9-Sep | mistyrose |
| Gm11734 | darkolivegreen |
| Tnrc6c | blue3 |
| Tmc6 | deeppink |
| Syngr2 | blue4 |
| Tk1 | blue3 |
| Afmid | blue4 |
| Birc5 | blue4 |
| Tha1 | blue4 |
| Socs3 | darkgrey |
| Pgs1 | blue4 |
| Cyth1 | darkgrey |
| Gm24060 | deeppink1 |
| Usp36 | darkgrey |
| Timp2 | mistyrose |
| Lgals3bp | saddlebrown |
| Cant1 | green4 |
| C1qtnf1 | darkolivegreen |
| Engase | darkgrey |
| Cbx2 | blue3 |
| Cbx8 | coral1 |
| Cbx4 | coral1 |
| Gm11754 | darkolivegreen |
| Tbc1d16 | blue4 |
| Gm11752 | blue4 |
| Gaa | green4 |
| Eif4a3 | darkgrey |
| Card14 | blue4 |
| Sgsh | green4 |
| Slc26a11 | green4 |
| Mir1932 | blue4 |
| Rnf213 | blue4 |
| Endov | blue4 |
| Rptor | blue3 |
| Chmp6 | blue3 |
| Gm11767 | blue3 |
| Gm11766 | darkolivegreen |
| Baiap2 | darkgrey |
| Cep131 | blue3 |
| Enthd2 | blue3 |
| 1810043H04Rik | deeppink |
| Slc38a10 | blue4 |
| 2810410L24Rik | blue3 |
| 2900052L18Rik | blue3 |
| Bahcc1 | blue3 |
| Gm11772 | darkgrey |
| Actg1 | mistyrose |
| 0610009L18Rik | green4 |
| 2310003H01Rik | mistyrose |
| Nploc4 | firebrick2 |
| Oxld1 | darkolivegreen |
| Ccdc137 | blue3 |
| Arl16 | coral1 |
| Hgs | mistyrose |
| Mrpl12 | blue4 |
| Gm11788 | blue4 |
| Slc25a10 | blue4 |
| Gm11789 | darkolivegreen |
| Gcgr | darkolivegreen |
| Fam195b | deeppink |
| P4hb | coral1 |
| Arhgdia | blue4 |
| Alyref | blue4 |
| Anapc11 | blue4 |
| Npb | green4 |
| Pcyt2 | darkolivegreen |
| Sirt7 | green4 |
| Mafg | mistyrose |
| Gm17586 | blue4 |
| Notum | blue4 |
| Aspscr1 | deeppink |
| Stra13 | blue4 |
| Lrrc45 | blue3 |
| Rac3 | blue3 |
| Dcxr | blue4 |
| Cbr2 | lightcoral |
| Rfng | green4 |
| Gps1 | deeppink |
| Dus1l | darkgrey |
| Fasn | blue3 |
| Ccdc57 | blue3 |
| Csnk1d | darkgrey |
| Gm28192 | deeppink1 |
| Gm11775 | blue4 |
| Sectm1b | blue4 |
| Sectm1a | green4 |
| Ogfod3 | blue4 |
| Hexdc | blue3 |
| BC017643 | blue4 |
| Narf | darkgrey |
| Foxk2 | blue4 |
| Wdr45b | blue4 |
| Rab40b | blue3 |
| Fn3krp | blue3 |
| Fn3k | darkolivegreen |
| Tbcd | blue4 |
| Zfp750 | darkolivegreen |
| B3gntl1 | blue4 |
| Metrnl | darkgrey |
| Rab10os | blue3 |
| Rab10 | blue4 |
| Kif3c | blue3 |
| 1110002L01Rik | blue3 |
| Asxl2 | blue4 |
| Dtnb | blue4 |
| Dnmt3a | blue3 |
| Pomc | blue4 |
| Dnajc27 | blue4 |
| Cenpo | blue4 |
| Ncoa1 | firebrick2 |
| Gm3625 | blue4 |
| Itsn2 | blue4 |
| Fam228b | antiquewhite2 |
| Pfn4 | darkolivegreen |
| Gm6682 | blue4 |
| Sf3b6 | blue3 |
| Fkbp1b | blue4 |
| BC068281 | blue3 |
| Mfsd2b | deeppink1 |
| Ubxn2a | coral1 |
| Atad2b | blue4 |
| Apob | mistyrose |
| 1110057K04Rik | blue3 |
| Gdf7 | blue3 |
| Hs1bp3 | blue3 |
| Rhob | coral1 |
| Pum2 | coral1 |
| Sdc1 | blue4 |
| Laptm4a | lightsteelblue |
| Wdr35 | blue4 |
| Ttc32 | blue3 |
| Osr1 | blue3 |
| Rdh14 | blue4 |
| Kcns3 | blue4 |
| Smc6 | blue4 |
| Gm4294 | blue4 |
| Fam49a | blue4 |
| Rpl36-ps3 | blue4 |
| Mycn | mistyrose |
| Ddx1 | coral1 |
| Nbas | blue3 |
| Fam84a | darkgrey |
| Trib2 | green4 |
| Mir6387 | blue3 |
| Lpin1 | blue4 |
| E2f6 | blue4 |
| Rock2 | blue4 |
| Pqlc3 | blue4 |
| Pdia6 | blue4 |
| Atp6v1c2 | blue4 |
| Nol10 | darkgrey |
| Odc1 | blue4 |
| Gm22748 | blue3 |
| Hpcal1 | blue4 |
| Gm10479 | mistyrose |
| 3110053B16Rik | blue4 |
| Gm10478 | blue3 |
| Zfp125 | blue4 |
| Asap2 | mistyrose |
| Itgb1bp1 | blue3 |
| Cpsf3 | blue4 |
| Gm25821 | blue3 |
| Iah1 | blue4 |
| Adam17 | blue4 |
| Gm22766 | blue3 |
| Ywhaq | blue4 |
| Taf1b | blue4 |
| Grhl1 | darkgrey |
| Gm6969 | coral1 |
| Klf11 | darkgrey |
| Cys1 | green4 |
| Rrm2 | blue4 |
| Mboat2 | blue3 |
| Kidins220 | firebrick2 |
| Id2 | blue4 |
| Rnf144a | blue4 |
| Rsad2 | blue3 |
| Cmpk2 | saddlebrown |
| Colec11 | blue3 |
| Rps7 | blue4 |
| Rnaseh1 | blue4 |
| Adi1 | blue3 |
| Trappc12 | blue3 |
| Tssc1 | blue4 |
| Pxdn | blue4 |
| Sntg2 | blue4 |
| Tmem18 | blue4 |
| Acp1 | coral1 |
| Sh3yl1 | blue3 |
| Fam110c | darkgrey |
| Gm24613 | darkolivegreen |
| Lamb1 | blue4 |
| Dld | mistyrose |
| Cbll1 | blue3 |
| Slc26a4 | blue4 |
| Bcap29 | blue4 |
| Dus4l | coral1 |
| Cog5 | blue4 |
| Hbp1 | mistyrose |
| Prkar2b | lightcoral |
| Ccdc71l | blue4 |
| Nampt | coral1 |
| Gdap10 | blue4 |
| Sypl | mistyrose |
| Atxn7l1 | blue3 |
| Twistnb | green4 |
| Twist1 | blue4 |
| Snx13 | coral1 |
| Ahr | blue4 |
| Tspan13 | blue4 |
| Bzw2 | blue4 |
| Ankmy2 | blue3 |
| Sostdc1 | blue3 |
| Ispd | coral1 |
| Agmo | blue3 |
| Etv1 | coral1 |
| Arl4a | blue3 |
| Scin | blue4 |
| Ifrd1 | darkgrey |
| Gm17024 | darkgrey |
| Zfp277 | blue4 |
| Dock4 | blue4 |
| Immp2l | blue4 |
| Dnajb9 | blue3 |
| Pnpla8 | mistyrose |
| Stxbp6 | firebrick2 |
| Prkd1 | blue3 |
| G2e3 | blue4 |
| Scfd1 | coral1 |
| Coch | blue4 |
| Strn3 | blue4 |
| Ap4s1 | deeppink |
| Hectd1 | deeppink |
| Heatr5a | blue4 |
| Dtd2 | coral1 |
| Nubpl | blue4 |
| Arhgap5 | coral1 |
| Egln3 | lightcoral |
| Sptssa | mistyrose |
| Eapp | coral1 |
| Gm22513 | blue3 |
| Snx6 | firebrick2 |
| Cfl2 | blue4 |
| Baz1a | blue4 |
| 2700097O09Rik | blue3 |
| Srp54b | green4 |
| Ppp2r3c | coral1 |
| 1110008L16Rik | firebrick2 |
| Psma6 | coral1 |
| Nfkbia | blue3 |
| Ralgapa1 | firebrick2 |
| Brms1l | blue3 |
| Mbip | blue4 |
| Gm6265 | blue4 |
| Slc25a21 | blue4 |
| Prps1l3 | blue3 |
| Mipol1 | blue4 |
| Clec14a | blue4 |
| Sec23a | deeppink |
| Gemin2 | blue4 |
| Trappc6b | deeppink |
| Pnn | blue3 |
| Gm5786 | blue4 |
| Mia2 | coral1 |
| Ctage5 | coral1 |
| Gm24233 | blue3 |
| Fbxo33 | blue3 |
| Gm527 | blue4 |
| Klhl28 | coral1 |
| Fam179b | coral1 |
| Prpf39 | blue4 |
| Fkbp3 | coral1 |
| Fancm | blue4 |
| Rps29 | firebrick2 |
| Rpl36al | green4 |
| Mgat2 | blue4 |
| Dnaaf2 | blue4 |
| 9330151L19Rik | blue3 |
| Pole2 | blue4 |
| Klhdc1 | darkgrey |
| Klhdc2 | coral1 |
| Nemf | coral1 |
| Gm9887 | blue4 |
| Arf6 | blue4 |
| Vcpkmt | blue4 |
| Sos2 | firebrick2 |
| L2hgdh | mistyrose |
| Atp5s | darkolivegreen |
| Cdkl1 | blue4 |
| Mir681 | green4 |
| 4930512B01Rik | green4 |
| Map4k5 | blue4 |
| Atl1 | blue4 |
| Gm3086 | darkgrey |
| Sav1 | blue4 |
| Nin | blue4 |
| Pygl | darkolivegreen |
| Tmx1 | green4 |
| Frmd6 | blue4 |
| Actr10 | blue3 |
| Psma3 | blue4 |
| 3110056K07Rik | blue3 |
| Arid4a | coral1 |
| Timm9 | blue4 |
| 2700049A03Rik | coral1 |
| Dact1 | darkgrey |
| Daam1 | blue4 |
| Gpr135 | darkolivegreen |
| L3hypdh | darkolivegreen |
| Jkamp | mistyrose |
| Pcnxl4 | blue4 |
| Dhrs7 | blue4 |
| Ppm1a | green4 |
| Six4 | blue3 |
| Mnat1 | blue4 |
| Trmt5 | blue4 |
| Slc38a6 | blue3 |
| Tmem30b | green4 |
| Prkch | darkgrey |
| Hif1a | blue4 |
| Snapc1 | blue4 |
| Gm11042 | blue4 |
| Rhoj | blue4 |
| Ppp2r5e | blue4 |
| Sgpp1 | blue4 |
| Syne2 | blue4 |
| Gm7862 | blue4 |
| Mthfd1 | blue4 |
| Zbtb25 | blue4 |
| Zbtb1 | blue4 |
| Hspa2 | orangered |
| Gm10451 | blue3 |
| Plekhg3 | mistyrose |
| Sptb | coral1 |
| Churc1 | blue4 |
| Fntb | blue4 |
| Rab15 | blue4 |
| Max | antiquewhite2 |
| Fut8 | blue3 |
| Gphn | firebrick2 |
| Mpp5 | brown1 |
| Atp6v1d | mistyrose |
| Eif2s1 | blue4 |
| Plek2 | blue3 |
| Tmem229b | blue4 |
| Plekhh1 | blue3 |
| Pigh | blue3 |
| Arg2 | lightcoral |
| Vti1b | blue4 |
| Gm17195 | blue3 |
| Gm17194 | blue4 |
| Rdh11 | blue4 |
| Zfyve26 | blue3 |
| Zfp36l1 | mistyrose |
| 2310015A10Rik | blue3 |
| Actn1 | blue4 |
| Dcaf5 | coral1 |
| Exd2 | blue3 |
| Erh | blue4 |
| Slc39a9 | blue3 |
| Plekhd1os | blue3 |
| Plekhd1 | blue3 |
| 4933426M11Rik | blue4 |
| Srsf5 | darkgrey |
| Smoc1 | lightcoral |
| Cox16 | coral1 |
| Gm28370 | blue3 |
| Synj2bp | coral1 |
| Med6 | green4 |
| Map3k9 | blue4 |
| Pcnx | blue3 |
| Sipa1l1 | green4 |
| Rgs6 | blue3 |
| Dpf3 | green4 |
| Dcaf4 | blue3 |
| Zfyve1 | blue4 |
| Rbm25 | blue4 |
| Psen1 | green4 |
| Numb | blue3 |
| 2410016O06Rik | blue4 |
| Acot2 | green4 |
| Acot1 | orangered |
| Acot4 | blue4 |
| 4732463B04Rik | blue4 |
| Acot3 | darkolivegreen |
| Acot6 | blue4 |
| Dnal1 | blue3 |
| Pnma1 | darkolivegreen |
| Elmsan1 | darkgrey |
| Gm5436 | blue4 |
| Ptgr2 | firebrick2 |
| Zfp410 | blue4 |
| Fam161b | blue3 |
| Coq6 | green4 |
| Entpd5 | coral1 |
| Ccdc176 | blue3 |
| Aldh6a1 | mistyrose |
| Lin52 | blue4 |
| Abcd4 | blue4 |
| Npc2 | firebrick2 |
| Isca2 | brown1 |
| Arel1 | darkgrey |
| Gm17193 | blue3 |
| Fcf1 | green4 |
| Ylpm1 | blue3 |
| Dlst | blue4 |
| Pgf | blue4 |
| Eif2b2 | darkolivegreen |
| Mlh3 | blue3 |
| Acyp1 | mistyrose |
| Zc2hc1c | blue4 |
| Nek9 | blue4 |
| Tmed10 | blue4 |
| Fos | coral1 |
| Jdp2 | darkgrey |
| Batf | honeydew |
| Mfsd7c | blue4 |
| 0610007P14Rik | deeppink |
| Ttll5 | blue3 |
| Tgfb3 | blue4 |
| Ift43 | blue4 |
| Gpatch2l | coral1 |
| 4732487G21Rik | blue3 |
| Esrrb | blue4 |
| Vash1 | blue4 |
| Angel1 | blue3 |
| Irf2bpl | deeppink |
| Cipc | blue3 |
| Pomt2 | green4 |
| Gstz1 | blue4 |
| Tmed8 | blue4 |
| Vipas39 | blue3 |
| Ahsa1 | blue4 |
| Sptlc2 | blue4 |
| Alkbh1 | darkgrey |
| Slirp | blue3 |
| Snw1 | deeppink |
| Adck1 | brown1 |
| Tshr | lightcoral |
| Gtf2a1 | coral1 |
| Ston2 | blue3 |
| Sel1l | blue4 |
| Gm6863 | blue4 |
| Galc | blue4 |
| Gpr65 | saddlebrown |
| Spata7 | deeppink |
| Ptpn21 | darkgrey |
| Zc3h14 | coral1 |
| Eml5 | coral1 |
| Ttc8 | darkolivegreen |
| Gm28933 | brown1 |
| Foxn3 | blue3 |
| Tdp1 | blue3 |
| Psmc1 | coral1 |
| Nrde2 | blue4 |
| Calm1 | blue4 |
| Ttc7b | blue4 |
| 9030617O03Rik | darkolivegreen |
| Ccdc88c | blue4 |
| Smek1 | coral1 |
| D130020L05Rik | darkolivegreen |
| Tc2n | blue3 |
| Fbln5 | coral1 |
| Trip11 | blue4 |
| Atxn3 | blue4 |
| Cpsf2 | blue4 |
| Rin3 | blue4 |
| Lgmn | blue4 |
| Golga5 | green4 |
| Itpk1 | deeppink |
| Tmem251 | blue3 |
| AK010878 | blue3 |
| Ubr7 | coral1 |
| Btbd7 | blue4 |
| Prima1 | green4 |
| Asb2 | blue3 |
| Otub2 | green4 |
| Ddx24 | darkgrey |
| Ifi27 | saddlebrown |
| Ifi27l2a | lightcoral |
| Ppp4r4 | blue4 |
| Serpina1f | blue4 |
| Serpina1b | blue4 |
| Serpina1d | blue4 |
| Serpina1a | darkolivegreen |
| Serpina1c | blue4 |
| Serpina3f | darkgrey |
| Serpina3g | darkgrey |
| Serpina3n | lightcoral |
| Dicer1 | blue4 |
| Clmn | blue3 |
| Gm28875 | blue4 |
| Syne3 | blue4 |
| Scarna13 | coral1 |
| Glrx5 | darkgrey |
| Bdkrb2 | mistyrose |
| Atg2b | blue4 |
| Gskip | blue4 |
| Ak7 | firebrick2 |
| Papola | blue4 |
| Vrk1 | blue4 |
| Bcl11b | blue3 |
| Setd3 | blue4 |
| Ccnk | darkgrey |
| Ccdc85c | blue4 |
| Cyp46a1 | blue3 |
| Eml1 | blue4 |
| Evl | blue4 |
| Degs2 | blue4 |
| Yy1 | blue4 |
| Slc25a29 | green4 |
| Mir345 | blue4 |
| Slc25a47 | blue3 |
| Wars | blue4 |
| Gm22079 | blue4 |
| Wdr25 | green4 |
| Dio3os | darkolivegreen |
| Mir1247 | darkgrey |
| Ppp2r5c | blue4 |
| Dync1h1 | blue4 |
| Gm17111 | darkgrey |
| 1700001K19Rik | darkgrey |
| Hsp90aa1 | darkgrey |
| Wdr20 | coral1 |
| Mok | darkolivegreen |
| Zfp839 | firebrick2 |
| Mpc1-ps | blue4 |
| Cinp | blue4 |
| Tecpr2 | blue4 |
| Rps19-ps6 | blue4 |
| Ankrd9 | coral1 |
| Rcor1 | blue4 |
| Traf3 | blue4 |
| Amn | blue4 |
| Cdc42bpb | blue4 |
| Exoc3l4 | blue3 |
| Tnfaip2 | blue4 |
| Gm266 | blue3 |
| Eif5 | brown1 |
| Snora28 | darkgrey |
| 2810029C07Rik | blue4 |
| Mark3 | green4 |
| Ckb | green4 |
| Trmt61a | blue4 |
| Bag5 | green4 |
| Apopt1 | blue3 |
| Gm15996 | blue4 |
| Klc1 | blue4 |
| Xrcc3 | blue4 |
| Zfyve21 | darkolivegreen |
| Ppp1r13b | mistyrose |
| 2010107E04Rik | blue4 |
| Aspg | darkolivegreen |
| Inf2 | blue4 |
| Adssl1 | darkolivegreen |
| Siva1 | blue4 |
| Akt1 | blue4 |
| Zbtb42 | blue4 |
| RP24-562D16.4 | blue3 |
| Cep170b | mistyrose |
| Pld4 | honeydew |
| BC022687 | blue3 |
| Cdca4 | darkgrey |
| Jag2 | blue4 |
| Nudt14 | deeppink |
| Brf1 | blue3 |
| Btbd6 | blue3 |
| Pacs2 | blue4 |
| Mta1 | blue3 |
| Crip2 | blue3 |
| Crip1 | blue4 |
| Igha | blue3 |
| Ighm | blue3 |
| Ighj1 | deeppink |
| Ighv1-26 | green4 |
| Ighv1-81 | blue3 |
| Zfp386 | coral1 |
| Wdr60 | firebrick2 |
| Esyt2 | blue4 |
| Rapgef5 | brown1 |
| Cdca7l | blue3 |
| Sp4 | coral1 |
| Itgb8 | firebrick2 |
| Macc1 | blue4 |
| Gm16505 | blue3 |
| Gdi2 | deeppink |
| Fam208b | blue4 |
| Asb13 | blue4 |
| Gm23084 | blue4 |
| Calml3 | blue4 |
| Net1 | blue4 |
| Akr1c14 | mistyrose |
| Akr1c18 | blue4 |
| Akr1c13 | blue4 |
| Akr1c19 | blue4 |
| Marcksl1-ps4 | blue3 |
| Akr1c12 | blue4 |
| Akr1c20 | blue4 |
| Akr1c21 | blue4 |
| Akr1e1 | firebrick2 |
| Rpl29-ps2 | blue3 |
| Klf6 | darkgrey |
| Pitrm1 | blue4 |
| Pfkp | blue4 |
| Gm10029 | blue4 |
| Wdr37 | deeppink |
| Idi1 | blue3 |
| Gtpbp4 | darkgrey |
| Larp4b | deeppink |
| Dip2c | blue4 |
| Zmynd11 | firebrick2 |
| Gm24187 | blue4 |
| Chrm3 | orangered |
| Mtr | firebrick2 |
| Heatr1 | blue4 |
| Lgals8 | blue4 |
| Ero1lb | darkolivegreen |
| Gpr137b-ps | blue4 |
| Gm2399 | blue4 |
| Gm2423 | blue4 |
| Gpr137b | darkolivegreen |
| Nid1 | blue4 |
| Lyst | blue4 |
| B3galnt2 | blue4 |
| Tbce | blue4 |
| Ggps1 | green4 |
| Arid4b | coral1 |
| Mrpl32 | blue4 |
| Psma2 | blue4 |
| AW209491 | coral1 |
| Sugct | mistyrose |
| Mplkip | blue3 |
| Cdk13 | blue4 |
| Mir466i | blue4 |
| Rala | blue4 |
| Yae1d1 | blue4 |
| Vdac3-ps1 | blue4 |
| Vps41 | coral1 |
| Stard3nl | blue4 |
| Epdr1 | blue4 |
| Sfrp4 | blue3 |
| Elmo1 | darkgrey |
| Aoah | blue4 |
| Trim27 | blue4 |
| Gpx6 | blue4 |
| Zscan12 | blue4 |
| Zkscan3 | blue3 |
| Mir6942 | blue4 |
| Pgbd1 | blue3 |
| Zscan26 | blue3 |
| Zkscan8 | blue3 |
| Zfp184 | deeppink1 |
| Hist1h2bk | darkolivegreen |
| Hist1h4i | blue4 |
| Zfp322a | blue3 |
| Abt1 | darkgrey |
| C230035I16Rik | blue3 |
| Hist1h4h | blue4 |
| Hist1h4d | blue4 |
| Hist1h2be | darkolivegreen |
| Gm24991 | blue4 |
| Hist1h2ac | blue4 |
| Hist1h2bc | blue4 |
| Hist1h4c | blue4 |
| Hfe | blue4 |
| Hist1h1c | blue4 |
| Slc17a3 | blue4 |
| Slc17a1 | mistyrose |
| Slc17a4 | blue3 |
| Lrrc16a | blue4 |
| Cmah | mistyrose |
| Fam65b | darkgrey |
| Gmnn | blue4 |
| BC005537 | deeppink1 |
| Gm22358 | darkgrey |
| Acot13 | darkolivegreen |
| Tdp2 | brown1 |
| D130043K22Rik | antiquewhite2 |
| Aldh5a1 | darkolivegreen |
| Gpld1 | darkolivegreen |
| Mrs2 | blue4 |
| Dcdc2a | blue4 |
| Gm11361 | blue4 |
| Sox4 | blue4 |
| Cdkal1 | blue4 |
| E2f3 | blue4 |
| Mboat1 | blue4 |
| Agtr1a | blue4 |
| Uqcrfs1 | blue4 |
| Dusp22 | blue4 |
| Exoc2 | coral1 |
| Foxq1 | darkgrey |
| Foxc1 | blue4 |
| Gmds | blue4 |
| Wrnip1 | blue3 |
| Serpinb1a | blue4 |
| Serpinb6b | blue4 |
| Serpinb9 | blue4 |
| Serpinb6a | blue4 |
| Nqo2 | blue4 |
| Ripk1 | coral1 |
| Bphl | blue4 |
| Tubb2a | blue4 |
| Tubb2b | blue4 |
| Psmg4 | blue4 |
| Slc22a23 | green4 |
| Pxdc1 | blue4 |
| Prpf4b | blue3 |
| Fam217a | coral1 |
| 4933417A18Rik | blue4 |
| Eci3 | blue4 |
| Gm16984 | blue3 |
| Eci2 | mistyrose |
| Cdyl | blue4 |
| Rpp40 | blue4 |
| Lyrm4 | blue4 |
| Fars2 | blue4 |
| Ly86 | saddlebrown |
| Rreb1 | green4 |
| Ssr1 | blue4 |
| Riok1 | darkgrey |
| Dsp | blue4 |
| Snrnp48 | blue4 |
| Bmp6 | blue4 |
| Txndc5 | blue3 |
| Bloc1s5 | blue3 |
| Eef1e1 | blue4 |
| Slc35b3 | green4 |
| Tfap2a | blue4 |
| Gcnt2 | blue4 |
| Pak1ip1 | blue4 |
| Tmem14c | blue3 |
| Elovl2 | blue4 |
| Smim13 | coral1 |
| Nedd9 | blue3 |
| Tmem170b | coral1 |
| Adtrp | blue4 |
| Hivep1 | blue4 |
| Edn1 | blue4 |
| Phactr1 | blue3 |
| Tbc1d7 | blue3 |
| Gfod1 | darkgrey |
| Sirt5 | blue3 |
| Nol7 | blue4 |
| Ranbp9 | deeppink |
| Mcur1 | blue4 |
| Cd83 | blue4 |
| Jarid2 | darkgrey |
| Dtnbp1 | blue3 |
| Hsp25-ps1 | darkgrey |
| Mylip | darkgrey |
| Gmpr | blue3 |
| Atxn1 | orangered |
| 5033430I15Rik | coral1 |
| C78339 | firebrick2 |
| Nup153 | blue4 |
| Kif13a | firebrick2 |
| Gm24620 | darkgrey |
| Nhlrc1 | green4 |
| Tpmt | mistyrose |
| Kdm1b | firebrick2 |
| Dek | blue4 |
| Rnf144b | blue3 |
| Id4 | darkolivegreen |
| Zfp169 | blue3 |
| 6720427I07Rik | coral1 |
| Mirlet7a-1 | blue3 |
| Ptpdc1 | darkolivegreen |
| Phf2 | mistyrose |
| Phf2os1 | blue3 |
| Fam120a | blue4 |
| Fam120aos | blue4 |
| Wnk2 | coral1 |
| Ninj1 | firebrick2 |
| 1110007C09Rik | blue4 |
| Susd3 | blue4 |
| Fgd3 | darkgrey |
| Bicd2 | blue4 |
| Ippk | blue4 |
| Cenpp | honeydew |
| Ecm2 | blue4 |
| Aspn | blue3 |
| Omd | mistyrose |
| Ogn | blue3 |
| Nol8 | coral1 |
| Iars | blue4 |
| Gm906 | blue4 |
| Gm24017 | darkolivegreen |
| Fbxw17 | blue4 |
| Spin1 | blue4 |
| Nxnl2 | blue4 |
| S1pr3 | coral1 |
| Cks2 | blue3 |
| Gm22806 | blue3 |
| Secisbp2 | blue3 |
| Gm15440 | darkgrey |
| Sema4d | blue4 |
| Gadd45g | coral1 |
| Syk | darkgrey |
| Auh | blue4 |
| Nfil3 | darkgrey |
| Ror2 | blue3 |
| Sptlc1 | blue4 |
| Sfxn1 | coral1 |
| Cplx2 | blue4 |
| Thoc3 | green4 |
| Gm2830 | coral1 |
| Simc1 | blue4 |
| 4833439L19Rik | blue4 |
| Arl10 | blue3 |
| Gm24195 | blue3 |
| Nop16 | blue4 |
| Higd2a | indianred2 |
| Cltb | darkgrey |
| Faf2 | blue4 |
| Rnf44 | blue3 |
| Cdhr2 | blue4 |
| Tspan17 | blue4 |
| Uimc1 | blue3 |
| Zfp346 | blue3 |
| Fgfr4 | green4 |
| Nsd1 | blue4 |
| Rab24 | darkolivegreen |
| Prelid1 | firebrick2 |
| Mxd3 | darkgrey |
| Lman2 | green4 |
| Slc34a1 | coral1 |
| Pfn3 | antiquewhite2 |
| Grk6 | blue4 |
| Prr7 | darkgrey |
| Dbn1 | blue4 |
| Pdlim7 | blue4 |
| Dok3 | blue4 |
| Ddx41 | blue4 |
| Fam193b | blue3 |
| Tmed9 | blue4 |
| B4galt7 | blue4 |
| Caml | blue4 |
| Ddx46 | blue4 |
| B230219D22Rik | blue4 |
| Txndc15 | darkgrey |
| Pcbd2 | blue4 |
| H2afy | blue4 |
| Tifab | blue4 |
| Cxcl14 | blue4 |
| Slc25a48 | mistyrose |
| Fbxl21 | blue4 |
| Tgfbi | honeydew |
| Smad5 | blue4 |
| Klhl3 | blue4 |
| Mir874 | green4 |
| Hnrnpa0 | blue4 |
| Idnk | saddlebrown |
| Ubqln1 | darkgrey |
| Gkap1 | deeppink |
| Kif27 | blue4 |
| 2210016F16Rik | blue4 |
| Hnrnpk | blue4 |
| Mir7-1 | coral1 |
| Rmi1 | firebrick2 |
| Agtpbp1 | firebrick2 |
| Naa35 | blue4 |
| Golm1 | blue4 |
| Isca1 | blue4 |
| Etohd2 | blue3 |
| Zcchc6 | deeppink |
| Gas1 | blue4 |
| Dapk1 | blue4 |
| Ctla2b | darkgrey |
| Ctla2a | darkgrey |
| Gm20563 | blue4 |
| Zfp808 | blue4 |
| Gm3604 | blue4 |
| Zfp935 | coral1 |
| Zfp934 | blue4 |
| 6720489N17Rik | blue4 |
| Gm5141 | blue4 |
| Fbp2 | blue4 |
| Fbp1 | blue4 |
| 2010111I01Rik | darkgrey |
| Gm16907 | darkgrey |
| Mir23b | blue4 |
| Mir27b | blue4 |
| Mir3074-1 | darkgrey |
| Fancc | blue3 |
| Ptch1 | darkgrey |
| Ercc6l2 | coral1 |
| Slc35d2 | coral1 |
| Zfp367 | blue4 |
| Habp4 | blue4 |
| Cdc14b | coral1 |
| 1810034E14Rik | green4 |
| Aaed1 | coral1 |
| Gm25654 | blue4 |
| Ctsl | coral1 |
| Cdk20 | blue4 |
| Hiatl1 | coral1 |
| Zfp369 | blue3 |
| Uqcrb | mistyrose |
| Gm10767 | blue3 |
| Mterf3 | coral1 |
| Ptdss1 | blue4 |
| Zfp759 | darkgrey |
| Rsl1 | blue4 |
| Zfp455 | blue4 |
| Zfp595 | coral1 |
| Zfp953 | darkgrey |
| Zfp429 | blue4 |
| Zfp874a | coral1 |
| Zfp874b | darkgrey |
| Zfp58 | coral1 |
| Zfp87 | coral1 |
| Zfp748 | coral1 |
| AA987161 | coral1 |
| RP23-206J9.6 | blue3 |
| A530054K11Rik | coral1 |
| Gm26587 | blue3 |
| Zfp738 | coral1 |
| Zfp65 | coral1 |
| Zfp85 | coral1 |
| Zfp493 | blue3 |
| Gm9625 | blue4 |
| Zfp273 | darkgrey |
| Gm10037 | blue3 |
| Mtrr | blue3 |
| Fastkd3 | blue4 |
| Papd7 | coral1 |
| Nsun2 | blue4 |
| Srd5a1 | blue4 |
| Gm26819 | deeppink1 |
| Ice1 | blue4 |
| Adamts16 | blue3 |
| Irx1 | blue4 |
| Rpl9-ps4 | blue4 |
| Gm20554 | blue3 |
| Irx2 | darkolivegreen |
| Gm10263 | blue3 |
| Ndufs6 | blue4 |
| Mrpl36 | blue3 |
| Lpcat1 | blue4 |
| Clptm1l | blue4 |
| Tert | blue4 |
| Slc6a18 | blue4 |
| Slc6a19 | blue4 |
| Slc6a19os | blue4 |
| Slc12a7 | green4 |
| Brd9 | blue4 |
| Slc9a3 | blue4 |
| Exoc3 | blue4 |
| Pdcd6 | blue4 |
| Sdha | mistyrose |
| Ccdc127 | blue4 |
| Zfp72 | coral1 |
| Gm10116 | blue4 |
| Zfp825 | coral1 |
| Erap1 | saddlebrown |
| Cast | blue4 |
| Gm4149 | blue4 |
| Ell2 | green4 |
| Gm23127 | darkgrey |
| Glrx | blue3 |
| Rhobtb3 | blue4 |
| Rfesd | mistyrose |
| Arsk | mistyrose |
| Ttc37 | blue4 |
| Ankrd32 | coral1 |
| 2210408I21Rik | blue4 |
| Fam172a | blue4 |
| Nr2f1 | blue4 |
| Arrdc3 | blue3 |
| 1700023H06Rik | blue4 |
| Gm8399 | blue4 |
| Lysmd3 | brown1 |
| Polr3g | blue4 |
| Mblac2 | blue3 |
| Cetn3 | blue4 |
| Mef2c | blue4 |
| Gm17750 | blue3 |
| Tmem161b | mistyrose |
| Gm4076 | blue3 |
| Ccnh | blue4 |
| Rasa1 | blue4 |
| Cox7c | blue4 |
| Gm22574 | blue3 |
| Xrcc4 | coral1 |
| Tmem167 | blue4 |
| Gm24507 | blue4 |
| Rps23 | blue4 |
| Atg10 | firebrick2 |
| Ssbp2 | blue3 |
| Acot12 | mistyrose |
| Gm24597 | blue3 |
| Zcchc9 | blue4 |
| Rasgrf2 | blue4 |
| Msh3 | firebrick2 |
| Dhfr | mistyrose |
| Fam151b | blue4 |
| Zfyve16 | blue4 |
| Serinc5 | blue4 |
| Mtx3 | firebrick2 |
| Papd4 | green4 |
| Homer1 | blue4 |
| Jmy | blue3 |
| Bhmt | blue4 |
| Bhmt2 | blue4 |
| Dmgdh | blue4 |
| Arsb | blue4 |
| Lhfpl2 | blue4 |
| Scamp1 | coral1 |
| Gm9776 | blue3 |
| Ap3b1 | firebrick2 |
| Tbca | antiquewhite2 |
| Wdr41 | blue4 |
| Pde8b | blue4 |
| Zbed3 | blue3 |
| Snora47 | blue4 |
| Gm22661 | blue4 |
| Aggf1 | blue4 |
| F2rl1 | blue4 |
| F2r | blue4 |
| Iqgap2 | blue4 |
| Poc5 | coral1 |
| Polk | blue3 |
| Gm17622 | blue3 |
| Col4a3bp | firebrick2 |
| Hmgcr | blue4 |
| Gcnt4 | blue4 |
| Nsa2 | coral1 |
| Gfm2 | darkolivegreen |
| Hexb | blue4 |
| Enc1 | blue4 |
| Arhgef28 | blue4 |
| Utp15 | blue4 |
| Ankra2 | blue3 |
| Btf3 | blue4 |
| Foxd1 | blue4 |
| Gm10320 | blue4 |
| Tmem174 | darkolivegreen |
| Tmem171 | blue4 |
| Fcho2 | firebrick2 |
| Tnpo1 | coral1 |
| Zfp366 | coral1 |
| Ptcd2 | blue3 |
| Mrps27 | blue4 |
| Map1b | blue4 |
| Mccc2 | blue4 |
| Bdp1 | blue4 |
| Serf1 | deeppink |
| Smn1 | blue4 |
| Gtf2h2 | deeppink |
| Ocln | blue3 |
| Marveld2 | blue4 |
| Gm24261 | blue3 |
| Rad17 | coral1 |
| Ak6 | coral1 |
| Ccdc125 | blue4 |
| Cdk7 | coral1 |
| Mrps36 | green4 |
| Ccnb1 | blue4 |
| Slc30a5 | mistyrose |
| Pik3r1 | blue4 |
| Cd180 | blue4 |
| Mast4 | blue4 |
| Srek1 | blue3 |
| Erbb2ip | deeppink |
| Nln | blue4 |
| Sgtb | green4 |
| Trappc13 | coral1 |
| Trim23 | mistyrose |
| Ppwd1 | coral1 |
| Cenpk | blue4 |
| Adamts6 | brown1 |
| Cwc27 | blue4 |
| Srek1ip1 | coral1 |
| Rnf180 | blue3 |
| Ipo11 | blue4 |
| Dimt1 | blue4 |
| Kif2a | blue4 |
| Apoo-ps | coral1 |
| Zswim6 | darkgrey |
| Smim15 | coral1 |
| Ndufaf2 | mistyrose |
| Ercc8 | blue4 |
| Elovl7 | blue4 |
| Depdc1b | blue4 |
| Pde4d | deeppink |
| Rps3a3 | blue4 |
| Plk2 | darkgrey |
| Gpbp1 | green4 |
| Gm10736 | deeppink |
| Mier3 | coral1 |
| Map3k1 | blue4 |
| Gm15327 | mistyrose |
| Il6st | blue4 |
| Slc38a9 | coral1 |
| Ppap2a | darkolivegreen |
| Skiv2l2 | firebrick2 |
| Dhx29 | firebrick2 |
| Gpx8 | blue4 |
| Esm1 | coral1 |
| BC067074 | darkolivegreen |
| Snx18 | blue4 |
| Arl15 | blue4 |
| Ndufs4 | mistyrose |
| Fst | blue4 |
| Mocs2 | green4 |
| Itga1 | blue4 |
| Pelo | mistyrose |
| Parp8 | blue4 |
| Emb | coral1 |
| Gm6421 | blue4 |
| Mrps30 | mistyrose |
| Nnt | darkolivegreen |
| Paip1 | coral1 |
| 4833420G17Rik | mistyrose |
| Gm7120 | darkolivegreen |
| Gm21967 | darkolivegreen |
| Ccl28 | blue3 |
| Hmgcs1 | firebrick2 |
| Nim1k | darkolivegreen |
| Zfp131 | brown1 |
| Flnb | blue4 |
| Abhd6 | blue4 |
| Rpp14 | blue4 |
| Pxk | blue4 |
| Pdhb | blue4 |
| Kctd6 | darkgrey |
| Acox2 | blue4 |
| Fam107a | darkolivegreen |
| Oit1 | blue4 |
| Gm9800 | blue4 |
| Fhit | blue4 |
| Ptprg | blue4 |
| 3830406C13Rik | blue3 |
| Gm5457 | blue4 |
| Thoc7 | green4 |
| Atxn7 | blue4 |
| Psmd6 | coral1 |
| Il3ra | blue4 |
| Slc4a7 | blue4 |
| Lrrc3b | coral1 |
| Oxsm | mistyrose |
| Ngly1 | deeppink |
| Top2b | blue4 |
| Rarb | blue4 |
| Thrb | firebrick2 |
| Nr1d2 | deeppink |
| Rpl15 | blue4 |
| Nkiras1 | deeppink |
| Ube2e1 | deeppink |
| Ube2e2 | green4 |
| Nid2 | mistyrose |
| 2700060E02Rik | blue4 |
| Gng2 | blue4 |
| Saysd1 | blue4 |
| Kcnk5 | coral1 |
| Nudt13 | darkolivegreen |
| Ecd | blue4 |
| Fam149b | mistyrose |
| Dnajc9 | blue4 |
| Mrps16 | blue3 |
| Anxa7 | blue4 |
| Ppp3cb | blue4 |
| 1810062O18Rik | darkgrey |
| Usp54 | deeppink |
| 2810402E24Rik | blue3 |
| Synpo2l | green4 |
| Sec24c | blue3 |
| 6230400D17Rik | deeppink1 |
| Fut11 | blue4 |
| Gm25328 | blue4 |
| Chchd1 | blue4 |
| Zswim8 | mistyrose |
| Ndst2 | blue4 |
| Camk2g | blue3 |
| Plau | blue4 |
| Vcl | blue4 |
| Ap3m1 | coral1 |
| Adk | blue4 |
| Kat6b | blue4 |
| Samd8 | blue3 |
| Vdac2 | blue4 |
| Comtd1 | blue4 |
| Zfp503 | green4 |
| Gm6158 | blue4 |
| Dlg5 | blue3 |
| Polr3a | blue4 |
| Rps24 | blue4 |
| Zmiz1 | mistyrose |
| Ppif | blue3 |
| Zcchc24 | blue4 |
| Anxa11 | blue4 |
| Slmap | firebrick2 |
| Dennd6a | coral1 |
| Arf4 | darkgrey |
| Pde12 | blue4 |
| Appl1 | blue4 |
| Il17rd | coral1 |
| Arhgef3 | blue3 |
| Fam208a | blue3 |
| Ccdc66 | blue3 |
| Gm22485 | blue4 |
| Erc2 | blue3 |
| Wnt5a | blue4 |
| Selk | blue3 |
| Actr8 | darkgrey |
| Il17rb | blue4 |
| Chdh | darkgrey |
| Dcp1a | blue4 |
| Tkt | blue4 |
| Prkcd | blue4 |
| Rft1 | blue4 |
| Sfmbt1 | coral1 |
| Tmem110 | blue4 |
| Mustn1 | blue3 |
| Itih1 | blue4 |
| Nek4 | blue4 |
| Spcs1 | antiquewhite2 |
| Glt8d1 | blue3 |
| Gnl3 | blue4 |
| Snord69 | darkgrey |
| Gm24916 | blue3 |
| Snord19 | darkgrey |
| Pbrm1 | blue4 |
| Smim4 | blue4 |
| Nt5dc2 | blue4 |
| Stab1 | blue4 |
| Nisch | green4 |
| Tnnc1 | blue4 |
| Sema3g | blue4 |
| Phf7 | blue4 |
| Bap1 | coral1 |
| Capn7 | coral1 |
| Sh3bp5 | green4 |
| Mettl6 | coral1 |
| Eaf1 | blue4 |
| Hacl1 | mistyrose |
| Btd | deeppink |
| Ankrd28 | coral1 |
| Galnt15 | green4 |
| Dph3 | darkolivegreen |
| Oxnad1 | blue4 |
| Ncoa4 | mistyrose |
| Timm23 | green4 |
| Parg | blue4 |
| Gm17210 | blue4 |
| Ogdhl | blue4 |
| Ercc6 | blue4 |
| 1810011H11Rik | blue4 |
| Vstm4 | blue4 |
| Arhgap22 | mistyrose |
| Mapk8 | deeppink1 |
| Gdf10 | blue3 |
| Anxa8 | lightcoral |
| Rpl23a-ps3 | blue4 |
| Fam35a | mistyrose |
| Glud1 | blue4 |
| Fam25c | blue4 |
| Sncg | blue4 |
| Mmrn2 | mistyrose |
| Bmpr1a | coral1 |
| Wapal | coral1 |
| Grid1 | blue4 |
| Ccser2 | firebrick2 |
| Ghitm | deeppink |
| Sh2d4b | blue4 |
| Tspan14 | blue3 |
| Fam213a | mistyrose |
| Mbl1 | blue4 |
| Gm7236 | blue4 |
| Gm16439 | coral1 |
| Txndc16 | blue4 |
| Ero1l | blue4 |
| Psmc6 | coral1 |
| Styx | blue3 |
| Gnpnat1 | mistyrose |
| Fermt2 | blue4 |
| Gm22637 | deeppink1 |
| Ddhd1 | blue4 |
| Gm7206 | blue4 |
| Gm1821 | blue3 |
| Gm15217 | darkolivegreen |
| Bmp4 | coral1 |
| Gm15222 | blue3 |
| Cdkn3 | darkolivegreen |
| Cnih1 | blue4 |
| Gmfb | coral1 |
| Cgrrf1 | blue3 |
| Samd4 | darkgrey |
| Gch1 | blue4 |
| Wdhd1 | blue4 |
| Socs4 | blue4 |
| Mapk1ip1l | blue4 |
| Lgals3 | blue4 |
| Fbxo34 | blue3 |
| Atg14 | mistyrose |
| Ktn1 | blue4 |
| Gm6055 | blue4 |
| Peli2 | blue4 |
| Tmem260 | blue3 |
| Exoc5 | coral1 |
| Ap5m1 | firebrick2 |
| Naa30 | coral1 |
| 1700011H14Rik | blue4 |
| 3632451O06Rik | blue4 |
| Ttc5 | blue3 |
| Rpph1 | blue4 |
| Parp2 | blue4 |
| Tep1 | blue3 |
| Gm26782 | blue4 |
| Osgep | blue3 |
| Apex1 | blue4 |
| Tmem55b | blue4 |
| Pnp | blue3 |
| Rnase4 | blue4 |
| Ang | darkolivegreen |
| Eddm3b | blue4 |
| Rnase6 | saddlebrown |
| Mettl17 | blue3 |
| Ndrg2 | blue4 |
| Arhgef40 | blue4 |
| Zfp219 | blue3 |
| Tmem253 | blue3 |
| Hnrnpc | blue4 |
| Supt16 | blue4 |
| Chd8 | blue4 |
| Snord8 | blue3 |
| Rab2b | blue3 |
| Tox4 | darkgrey |
| Mettl3 | blue3 |
| Sall2 | blue3 |
| Trav3-4 | blue4 |
| Trac | blue4 |
| Dad1 | blue3 |
| Abhd4 | blue4 |
| Oxa1l | brown1 |
| Slc7a7 | blue4 |
| Mrpl52 | blue4 |
| Mmp14 | blue4 |
| Lrp10 | deeppink |
| Rem2 | darkgrey |
| Prmt5 | blue4 |
| Haus4 | blue4 |
| Ajuba | blue4 |
| 4931414P19Rik | blue3 |
| Psmb5 | blue4 |
| Acin1 | blue3 |
| 4930579G18Rik | blue4 |
| 1700123O20Rik | deeppink |
| Slc7a8 | green4 |
| Homez | darkgrey |
| Ppp1r3e | blue3 |
| Bcl2l2 | blue3 |
| Pabpn1 | blue3 |
| Slc22a17 | green4 |
| Ngdn | blue4 |
| Thtpa | darkgrey |
| Zfhx2 | blue3 |
| Ap1g2 | blue3 |
| Gm8894 | blue4 |
| Dhrs4 | mistyrose |
| Lrrc16b | blue3 |
| Pck2 | blue4 |
| Dcaf11 | blue4 |
| Gm15932 | blue3 |
| Fitm1 | blue4 |
| Psme1 | saddlebrown |
| Emc9 | darkolivegreen |
| Psme2 | saddlebrown |
| Rnf31 | blue3 |
| Irf9 | saddlebrown |
| Rec8 | darkolivegreen |
| Ipo4 | blue4 |
| Tm9sf1 | blue4 |
| Mdp1 | blue4 |
| Nedd8 | antiquewhite2 |
| Gmpr2 | blue4 |
| Tinf2 | blue4 |
| Tgm1 | darkgrey |
| Rabggta | blue3 |
| Dhrs1 | blue4 |
| Nop9 | blue4 |
| Cideb | blue4 |
| Adcy4 | blue3 |
| Ripk3 | blue4 |
| Nfatc4 | blue4 |
| Nynrin | blue3 |
| Khnyn | darkolivegreen |
| Sdr39u1 | blue4 |
| Cenpj | blue3 |
| Parp4 | blue3 |
| Mphosph8 | blue4 |
| Gm16973 | blue3 |
| Pspc1 | blue4 |
| Zmym5 | deeppink |
| 2410022M11Rik | blue4 |
| Zmym2 | blue4 |
| Gjb2 | coral1 |
| Gjb6 | darkolivegreen |
| Cryl1 | blue4 |
| Ift88 | firebrick2 |
| Il17d | blue4 |
| N6amt2 | blue4 |
| Xpo4 | blue4 |
| Lats2 | blue4 |
| Gm26440 | darkgrey |
| Gm20430 | darkgrey |
| Sap18 | mistyrose |
| Mrpl57 | blue4 |
| Zdhhc20 | brown1 |
| Micu2 | coral1 |
| 3110083C13Rik | darkolivegreen |
| Fgf9 | mistyrose |
| Rcbtb1 | blue4 |
| Phf11b | saddlebrown |
| Phf11d | blue4 |
| Phf11c | blue3 |
| Setdb2 | blue4 |
| Cab39l | firebrick2 |
| Cdadc1 | deeppink |
| Nupl1 | blue4 |
| Mtmr6 | coral1 |
| Spata13 | blue3 |
| Mipep | blue4 |
| Ebpl | blue4 |
| Kpna3 | blue4 |
| Spryd7 | coral1 |
| Trim13 | blue4 |
| Dleu2 | blue3 |
| Gm27010 | blue4 |
| Dleu7 | green4 |
| Rnaseh2b | blue4 |
| Gm4131 | blue4 |
| Fam124a | blue4 |
| Ints6 | blue4 |
| Wdfy2 | blue4 |
| Defb42 | darkgrey |
| Ctsb | blue4 |
| Fdft1 | blue4 |
| Neil2 | darkolivegreen |
| Fam167a | darkgrey |
| Mtmr9 | blue4 |
| Xkr6 | darkolivegreen |
| Pinx1 | blue4 |
| Sox7 | darkgrey |
| Msra | blue4 |
| Kif13b | blue4 |
| Hmbox1 | blue4 |
| Ints9 | blue3 |
| Extl3 | blue3 |
| Fbxo16 | blue3 |
| Zfp395 | blue3 |
| Elp3 | blue4 |
| Pbk | blue4 |
| Ccdc25 | coral1 |
| Clu | blue4 |
| Ephx2 | blue4 |
| Ptk2b | darkgrey |
| Trim35 | blue4 |
| Adra1a | blue3 |
| Dpysl2 | blue4 |
| Bnip3l | mistyrose |
| Ppp2r2a | darkgrey |
| Kctd9 | blue4 |
| Gnrh1 | blue3 |
| Dock5 | blue4 |
| Stc1 | blue3 |
| Nkx3-1 | darkgrey |
| Slc25a37 | blue3 |
| Loxl2 | blue4 |
| R3hcc1 | blue4 |
| Chmp7 | green4 |
| 4930480K23Rik | darkolivegreen |
| Tnfrsf10b | blue4 |
| Rhobtb2 | blue3 |
| Egr3 | coral1 |
| Bin3 | blue4 |
| Ccar2 | blue3 |
| 9930012K11Rik | blue3 |
| Pdlim2 | green4 |
| Sorbs3 | deeppink |
| Ppp3cc | mistyrose |
| Slc39a14 | darkgrey |
| Polr3d | darkgrey |
| Phyhip | green4 |
| Bmp1 | blue4 |
| Reep4 | blue4 |
| Hr | mistyrose |
| Nudt18 | blue4 |
| Fam160b2 | blue3 |
| Dmtn | green4 |
| Xpo7 | coral1 |
| Dok2 | green4 |
| Fndc3a | blue3 |
| Rcbtb2 | mistyrose |
| Rb1 | blue4 |
| Lpar6 | mistyrose |
| Itm2b | blue4 |
| Med4 | blue4 |
| Sucla2 | deeppink |
| Gm6984 | blue4 |
| Esd | blue4 |
| Lrch1 | blue4 |
| Lcp1 | blue4 |
| Cpb2 | blue4 |
| Zc3h13 | blue4 |
| 4930564B18Rik | darkolivegreen |
| Cog3 | coral1 |
| Slc25a30 | green4 |
| Gm4285 | blue4 |
| Tpt1 | darkolivegreen |
| Snora31 | darkgrey |
| Gtf2f2 | blue4 |
| Gpalpp1 | coral1 |
| Nufip1 | blue4 |
| Tsc22d1 | darkolivegreen |
| Lacc1 | blue4 |
| Ccdc122 | blue3 |
| Gm23823 | blue4 |
| Dnajc15 | coral1 |
| Epsti1 | saddlebrown |
| Akap11 | blue4 |
| Dgkh | brown1 |
| Gm26251 | darkgrey |
| Vwa8 | blue4 |
| Rgcc | green4 |
| Naa16 | blue4 |
| Mtrf1 | coral1 |
| Kbtbd7 | firebrick2 |
| Wbp4 | coral1 |
| Elf1 | blue4 |
| Sugt1 | blue4 |
| Pcdh8 | blue4 |
| Gm6999 | firebrick2 |
| Gm10845 | blue3 |
| Olfm4 | firebrick2 |
| Pcdh17 | lightsteelblue |
| Tdrd3 | firebrick2 |
| Rps3a2 | blue4 |
| Dach1 | blue3 |
| Rpl36a-ps1 | blue4 |
| Mzt1 | coral1 |
| Bora | blue4 |
| Dis3 | blue4 |
| Pibf1 | blue4 |
| Klf5 | darkgrey |
| Klf12 | firebrick2 |
| Tbc1d4 | blue3 |
| Commd6 | coral1 |
| Uchl3 | blue4 |
| Gm9922 | blue3 |
| Lmo7 | deeppink1 |
| Kctd12 | blue3 |
| Cln5 | blue4 |
| Fbxl3 | deeppink |
| Mycbp2 | blue4 |
| Scel | darkolivegreen |
| Slain1 | blue3 |
| Ednrb | blue3 |
| Rnf219 | blue4 |
| Rbm26 | blue4 |
| Gm17066 | blue4 |
| Ndfip2 | blue4 |
| Gm10076 | blue4 |
| Spry2 | darkgrey |
| Slitrk6 | darkolivegreen |
| Mir17hg | blue4 |
| Gm20713 | blue4 |
| Gpc6 | lightsteelblue |
| Tgds | mistyrose |
| Gpr180 | blue4 |
| Abcc4 | blue4 |
| Cldn10 | blue4 |
| Dzip1 | blue4 |
| Dnajc3 | firebrick2 |
| Uggt2 | blue4 |
| Oxgr1 | mistyrose |
| Mbnl2 | green4 |
| Rap2a | blue4 |
| Ipo5 | blue4 |
| Farp1 | blue3 |
| B930095G15Rik | antiquewhite2 |
| Stk24 | blue4 |
| Dock9 | firebrick2 |
| 1810041H14Rik | blue4 |
| Ubac2 | coral1 |
| A330035P11Rik | firebrick2 |
| Tm9sf2 | blue3 |
| Clybl | blue4 |
| Pcca | blue4 |
| Ggact | blue4 |
| Tmtc4 | blue3 |
| Sepp1 | mistyrose |
| Ccdc152 | mistyrose |
| Ghr | mistyrose |
| Fbxo4 | blue4 |
| AW549877 | firebrick2 |
| Oxct1 | blue4 |
| C7 | blue4 |
| Card6 | blue3 |
| Rpl37 | blue4 |
| Snord72 | blue4 |
| Gm10250 | blue4 |
| Prkaa1 | blue4 |
| Ttc33 | mistyrose |
| Ptger4 | blue4 |
| Dab2 | blue3 |
| Rictor | coral1 |
| Osmr | blue4 |
| Lifr | deeppink |
| Egflam | deeppink |
| Wdr70 | blue4 |
| Gm27529 | blue4 |
| Nup155 | blue4 |
| 2410089E03Rik | blue4 |
| Nipbl | blue4 |
| Slc1a3 | lightcoral |
| Ranbp3l | darkolivegreen |
| Nadk2 | mistyrose |
| Skp2 | blue4 |
| Lmbrd2 | firebrick2 |
| Ugt3a1 | blue4 |
| Ugt3a2 | blue4 |
| Prlr | blue4 |
| Agxt2 | blue4 |
| Dnajc21 | coral1 |
| Brix1 | blue4 |
| Rad1 | blue4 |
| Rai14 | blue4 |
| C1qtnf3 | blue4 |
| Amacr | blue4 |
| Tars | blue4 |
| Npr3 | blue3 |
| Sub1 | blue3 |
| Zfr | coral1 |
| Mir1898 | blue3 |
| Mtmr12 | darkgrey |
| Golph3 | blue3 |
| Pdzd2 | darkolivegreen |
| 6030458C11Rik | blue4 |
| Drosha | blue3 |
| Cdh6 | blue4 |
| Basp1 | blue4 |
| Myo10 | darkgrey |
| Fam134b | blue3 |
| Zfp622 | darkgrey |
| Fbxl7 | blue3 |
| Ank | blue4 |
| Otulin | darkolivegreen |
| Fam105a | blue4 |
| Trio | blue4 |
| Dap | blue4 |
| Ankrd33b | darkgrey |
| Ropn1l | blue4 |
| 6-Mar | blue4 |
| Cmbl | blue4 |
| Cct5 | blue4 |
| Fam173b | green4 |
| Snhg18 | green4 |
| Snord123 | blue4 |
| Sema5a | blue4 |
| Sdc2 | blue4 |
| Cpq | blue4 |
| Tspyl5 | mistyrose |
| Mtdh | blue4 |
| Gm24500 | blue4 |
| Laptm4b | darkgrey |
| Gm25809 | darkgrey |
| Matn2 | indianred2 |
| Rpl30 | blue4 |
| Hrsp12 | mistyrose |
| Pop1 | blue3 |
| Gm22208 | darkolivegreen |
| Stk3 | blue4 |
| Osr2 | darkgrey |
| Vps13b | coral1 |
| Cox6c | blue3 |
| Polr2k | blue4 |
| Spag1 | blue3 |
| Rnf19a | blue4 |
| Ankrd46 | blue3 |
| Snx31 | blue4 |
| Pabpc1 | blue4 |
| Ywhaz | green4 |
| Zfp706 | blue4 |
| Gm16136 | blue4 |
| Grhl2 | blue4 |
| Ncald | blue4 |
| Rrm2b | mistyrose |
| Gm3362 | blue4 |
| Ubr5 | blue4 |
| Klf10 | darkgrey |
| Azin1 | blue4 |
| Atp6v1c1 | blue4 |
| Fzd6 | coral1 |
| Gm24789 | darkgrey |
| Slc25a32 | mistyrose |
| Dcaf13 | coral1 |
| n-R5s39 | blue4 |
| Gm9522 | blue3 |
| Dpys | blue4 |
| Lrp12 | firebrick2 |
| Oxr1 | brown1 |
| Angpt1 | firebrick2 |
| Eif3e | green4 |
| Emc2 | coral1 |
| Nudcd1 | blue4 |
| Eny2 | blue4 |
| Gm22392 | darkgrey |
| Ebag9 | mistyrose |
| Trps1 | lightsteelblue |
| Mir1907 | darkolivegreen |
| Eif3h | lightsteelblue |
| Utp23 | blue4 |
| Rad21 | mistyrose |
| Med30 | blue4 |
| Ext1 | blue4 |
| Samd12 | blue4 |
| Mal2 | blue4 |
| Enpp2 | blue4 |
| Taf2 | blue4 |
| Deptor | firebrick2 |
| Col14a1 | coral1 |
| Mrpl13 | coral1 |
| Mtbp | blue4 |
| Sntb1 | blue4 |
| Slc22a22 | blue4 |
| Zhx2 | blue3 |
| Gm16006 | blue4 |
| Derl1 | blue3 |
| Tbc1d31 | blue3 |
| 9130401M01Rik | blue4 |
| Zhx1 | coral1 |
| Atad2 | blue4 |
| Wdyhv1 | blue3 |
| Fbxo32 | blue3 |
| Anxa13 | blue3 |
| D15Ertd621e | coral1 |
| Tmem65 | green4 |
| Trmt12 | blue3 |
| Rnf139 | coral1 |
| Tatdn1 | blue4 |
| Ndufb9 | blue4 |
| Mtss1 | blue4 |
| Sqle | blue4 |
| E430025E21Rik | blue4 |
| Nsmce2 | blue4 |
| Trib1 | darkgrey |
| Fam84b | blue4 |
| 9930014A18Rik | blue4 |
| Myc | darkgrey |
| Gsdmc2 | lightcoral |
| Gsdmc3 | lightcoral |
| Fam49b | blue4 |
| Asap1 | blue4 |
| Efr3a | blue4 |
| Gm20405 | darkgrey |
| Phf20l1 | coral1 |
| Sla | honeydew |
| Wisp1 | blue4 |
| Ndrg1 | blue3 |
| St3gal1 | blue4 |
| Zfat | green4 |
| Gm26621 | blue3 |
| Khdrbs3 | green4 |
| Gm23217 | blue4 |
| Trappc9 | coral1 |
| Gm3150 | blue4 |
| Chrac1 | saddlebrown |
| Ago2 | mistyrose |
| Ptk2 | blue4 |
| Dennd3 | antiquewhite2 |
| Slc45a4 | green4 |
| Ptp4a3 | blue4 |
| Gm3244 | blue4 |
| Arc | darkgrey |
| Jrk | blue4 |
| Psca | lightcoral |
| Them6 | blue4 |
| Lypd2 | coral1 |
| Lynx1 | blue3 |
| Ly6d | lightcoral |
| D730001G18Rik | green4 |
| Ly6e | blue4 |
| Ly6a | saddlebrown |
| Ly6c1 | blue4 |
| BC025446 | blue4 |
| Ly6f | darkolivegreen |
| 9030619P08Rik | blue4 |
| Gpihbp1 | blue3 |
| Zfp41 | blue4 |
| 2810039B14Rik | blue3 |
| Top1mt | coral1 |
| Rhpn1 | green4 |
| Zc3h3 | deeppink |
| Gm23747 | blue3 |
| Gsdmd | blue4 |
| Naprt | green4 |
| Eef1d | blue4 |
| Pycrl | green4 |
| Tsta3 | blue4 |
| Zfp623 | darkgrey |
| Zfp707 | coral1 |
| Ccdc166 | blue3 |
| Mapk15 | green4 |
| Fam83h | darkgrey |
| K230010J24Rik | blue3 |
| Gm23027 | blue3 |
| Scrib | blue3 |
| Puf60 | blue4 |
| Nrbp2 | blue3 |
| Plec | blue4 |
| Parp10 | saddlebrown |
| Grina | green4 |
| Gm10872 | blue4 |
| Spatc1 | blue4 |
| Oplah | blue4 |
| Exosc4 | blue3 |
| Gpaa1 | deeppink |
| Cyc1 | blue4 |
| Sharpin | blue4 |
| Maf1 | blue4 |
| Hgh1 | blue4 |
| Mroh1 | green4 |
| Bop1 | blue4 |
| Scx | blue3 |
| Hsf1 | blue3 |
| Dgat1 | coral1 |
| Fbxl6 | blue3 |
| Slc52a2 | coral1 |
| Adck5 | green4 |
| Cpsf1 | blue3 |
| Tonsl | blue4 |
| Cyhr1 | blue4 |
| Kifc2 | darkolivegreen |
| Ppp1r16a | blue4 |
| Gpt | blue4 |
| Mfsd3 | blue4 |
| Lrrc14 | blue3 |
| Lrrc24 | green4 |
| Arhgap39 | green4 |
| Zfp251 | blue3 |
| Zfp7 | blue3 |
| Commd5 | blue3 |
| Rpl8 | blue4 |
| Zfp647 | blue3 |
| 1110038F14Rik | blue3 |
| Rbfox2 | blue4 |
| Apol9b | saddlebrown |
| Myh9 | blue4 |
| Gm22107 | darkgrey |
| Txn2 | deeppink |
| Foxred2 | lightsteelblue |
| Eif3d | blue4 |
| Ift27 | deeppink |
| Pvalb | blue4 |
| Ncf4 | honeydew |
| Tst | blue4 |
| Mpst | green4 |
| Kctd17 | deeppink |
| Il2rb | blue4 |
| C1qtnf6 | blue4 |
| Gm6723 | blue3 |
| Rac2 | honeydew |
| Cyth4 | blue4 |
| Mfng | deeppink |
| Card10 | blue4 |
| Cdc42ep1 | blue4 |
| Gga1 | deeppink |
| Mir6955 | blue3 |
| Sh3bp1 | blue3 |
| Lgals1 | blue4 |
| Nol12 | blue4 |
| Triobp | blue4 |
| H1f0 | blue4 |
| Gcat | blue4 |
| Ankrd54 | deeppink |
| Eif3l | blue4 |
| Micall1 | blue4 |
| Polr2f | blue4 |
| Pick1 | blue3 |
| Baiap2l2 | blue3 |
| Pla2g6 | deeppink |
| Maff | darkgrey |
| Tmem184b | blue4 |
| Mir1943 | blue4 |
| Csnk1e | darkgrey |
| Kdelr3 | blue4 |
| Ddx17 | blue3 |
| Cby1 | brown1 |
| Tomm22 | blue4 |
| Josd1 | coral1 |
| Gtpbp1 | deeppink |
| Sun2 | blue4 |
| Dnal4 | green4 |
| Nptxr | blue4 |
| Cbx6 | green4 |
| Apobec3 | blue4 |
| Cbx7 | darkolivegreen |
| Pdgfb | blue4 |
| Mir7213 | blue4 |
| Rpl3 | blue4 |
| Snord83b | blue4 |
| Syngr1 | blue4 |
| Tab1 | darkgrey |
| Mgat3 | darkolivegreen |
| Mief1 | blue4 |
| Atf4 | darkgrey |
| Rps19bp1 | blue4 |
| Fam83f | darkgrey |
| Tnrc6b | firebrick2 |
| Adsl | blue4 |
| Mkl1 | mistyrose |
| Mchr1 | darkgrey |
| Slc25a17 | coral1 |
| St13 | blue3 |
| Xpnpep3 | blue4 |
| Rbx1 | blue4 |
| RP23-310L8.1 | blue3 |
| Ep300 | blue4 |
| L3mbtl2 | blue4 |
| Chadl | blue4 |
| Rangap1 | blue4 |
| Zc3h7b | green4 |
| Tef | blue4 |
| Tob2 | coral1 |
| Phf5a | blue4 |
| Aco2 | blue4 |
| Polr3h | blue4 |
| Csdc2 | mistyrose |
| Pmm1 | blue4 |
| Gm5805 | blue4 |
| Xrcc6 | blue4 |
| Desi1 | blue4 |
| Nhp2l1 | blue4 |
| Ccdc134 | blue3 |
| Srebf2 | coral1 |
| Cenpm | blue4 |
| Naga | green4 |
| Smdt1 | indianred2 |
| Ndufa6 | blue4 |
| Cyp2d22 | lightcoral |
| Cyp2d9 | blue4 |
| Cyp2d12 | blue4 |
| Cyp2d26 | blue4 |
| Tcf20 | coral1 |
| Gm20324 | blue4 |
| Nfam1 | blue4 |
| Serhl | darkolivegreen |
| Rrp7a | blue4 |
| Poldip3 | darkgrey |
| Rnu12 | mistyrose |
| Cyb5r3 | blue4 |
| A4galt | blue4 |
| Arfgap3 | darkgrey |
| Pacsin2 | coral1 |
| Gm5417 | blue4 |
| Ttll1 | blue3 |
| Bik | green4 |
| Mcat | blue4 |
| Tspo | blue4 |
| Ttll12 | blue4 |
| Mpped1 | blue4 |
| Samm50 | deeppink1 |
| Parvb | blue4 |
| Parvg | honeydew |
| Ldoc1l | blue3 |
| Prr5 | coral1 |
| Phf21b | blue3 |
| Nup50 | blue4 |
| 5031439G07Rik | mistyrose |
| Upk3a | lightcoral |
| Fam118a | blue4 |
| Fbln1 | mistyrose |
| Atxn10 | blue4 |
| Wnt7b | blue3 |
| Mirlet7c-2 | blue4 |
| Mirlet7b | blue4 |
| Ppara | mistyrose |
| Cdpf1 | green4 |
| Ttc38 | darkolivegreen |
| Trmu | blue3 |
| Celsr1 | blue3 |
| Gramd4 | mistyrose |
| Cerk | blue4 |
| Tbc1d22a | coral1 |
| Fam19a5 | blue4 |
| Brd1 | coral1 |
| Zbed4 | blue4 |
| Alg12 | blue4 |
| Creld2 | mistyrose |
| Pim3 | coral1 |
| 1810021B22Rik | darkolivegreen |
| Trabd | firebrick2 |
| Selo | blue4 |
| Tubgcp6 | blue3 |
| Hdac10 | blue3 |
| Mapk12 | blue3 |
| Mapk11 | blue4 |
| Plxnb2 | blue3 |
| Dennd6b | blue3 |
| Gm26798 | blue3 |
| Ppp6r2 | blue3 |
| Sbf1 | blue4 |
| Adm2 | deeppink |
| Miox | blue4 |
| Lmf2 | deeppink |
| Ncaph2 | deeppink |
| Chkb | darkgrey |
| Arsa | mistyrose |
| Shank3 | darkgrey |
| Rabl2 | blue3 |
| Alg10b | blue4 |
| Cpne8 | blue4 |
| Rpl31-ps8 | blue4 |
| Tcea1-ps1 | blue4 |
| Kif21a | firebrick2 |
| Slc2a13 | blue4 |
| Lrrk2 | mistyrose |
| Gm15382 | darkolivegreen |
| Gxylt1 | blue4 |
| Yaf2 | blue3 |
| Zcrb1 | blue4 |
| Pphln1 | blue4 |
| Prickle1 | blue4 |
| Pus7l | blue4 |
| Irak4 | blue4 |
| Twf1 | blue4 |
| Tmem117 | darkolivegreen |
| Ano6 | blue4 |
| Gm17546 | darkolivegreen |
| 2610037D02Rik | blue3 |
| Arid2 | blue4 |
| Scaf11 | coral1 |
| Slc38a2 | darkgrey |
| Amigo2 | blue4 |
| Pced1b | blue3 |
| Rpap3 | coral1 |
| Endou | blue4 |
| Rapgef3 | blue3 |
| Rapgef3os2 | blue4 |
| Slc48a1 | blue4 |
| Hdac7 | blue4 |
| Vdr | blue4 |
| Tmem106c | brown1 |
| Senp1 | blue4 |
| Pfkm | darkolivegreen |
| Asb8 | blue4 |
| Zfp641 | darkolivegreen |
| Kansl2 | blue4 |
| Snora34 | blue3 |
| Snora2b | blue4 |
| Ccnt1 | blue4 |
| 4930415O20Rik | green4 |
| Adcy6 | blue3 |
| Cacnb3 | blue3 |
| Ddx23 | blue4 |
| Rnd1 | darkgrey |
| Fkbp11 | blue4 |
| Arf3 | blue3 |
| Ddn | blue3 |
| Prkag1 | deeppink |
| Kmt2d | blue3 |
| Rhebl1 | coral1 |
| Lmbr1l | blue4 |
| Tuba1b | blue4 |
| Tuba1a | blue4 |
| Tuba1c | darkgrey |
| C1ql4 | blue4 |
| Dnajc22 | blue4 |
| Spats2 | blue4 |
| Mcrs1 | blue4 |
| Prpf40b | blue3 |
| Fmnl3 | blue4 |
| Tmbim6 | blue4 |
| Nckap5l | mistyrose |
| Bcdin3d | blue3 |
| Aqp2 | green4 |
| Aqp6 | green4 |
| Racgap1 | blue4 |
| Asic1 | blue3 |
| Gm17349 | blue4 |
| Smarcd1 | blue4 |
| Gpd1 | blue4 |
| Cox14 | green4 |
| Cers5 | blue4 |
| Lima1 | blue4 |
| Gm25897 | blue4 |
| Larp4 | deeppink |
| Dip2b | blue4 |
| Atf1 | blue4 |
| Mettl7a1 | blue4 |
| Mettl7a2 | blue4 |
| Higd1c | darkolivegreen |
| Slc11a2 | green4 |
| Gm5475 | darkolivegreen |
| Letmd1 | blue3 |
| Csrnp2 | mistyrose |
| Tfcp2 | blue3 |
| Pou6f1 | mistyrose |
| Gm27209 | blue3 |
| C330013E15Rik | darkolivegreen |
| Dazap2 | darkolivegreen |
| Smagp | blue4 |
| Cela1 | blue3 |
| Slc4a8 | blue3 |
| Fignl2 | blue3 |
| Acvrl1 | deeppink |
| Acvr1b | darkgrey |
| Grasp | blue3 |
| Nr4a1 | coral1 |
| Atg101 | blue4 |
| Krt80 | coral1 |
| Krt7 | blue4 |
| Krt5 | blue4 |
| Krt8 | darkgrey |
| Krt18 | blue4 |
| Eif4b | mistyrose |
| Tenc1 | darkgrey |
| Spryd3 | blue4 |
| Igfbp6 | lightcoral |
| Csad | blue4 |
| Zfp740 | blue3 |
| Rarg | blue4 |
| Mfsd5 | blue4 |
| Pfdn5 | blue4 |
| Myg1 | blue3 |
| Aaas | blue4 |
| Sp1 | blue4 |
| Prr13 | blue4 |
| Pcbp2 | coral1 |
| Map3k12 | blue3 |
| Tarbp2 | blue4 |
| Npff | blue4 |
| Atf7 | blue4 |
| Rpl39-ps | blue4 |
| Atp5g2 | coral1 |
| Calcoco1 | blue4 |
| Hoxc11 | blue4 |
| Hoxc10 | blue3 |
| Hoxc5 | blue3 |
| Hoxc9 | blue4 |
| Hoxc8 | blue4 |
| Hoxc6 | blue3 |
| Hoxc4 | blue3 |
| Smug1 | blue4 |
| Cbx5 | blue4 |
| Hnrnpa1 | blue4 |
| Copz1 | deeppink |
| Zfp385a | blue4 |
| Itga5 | blue4 |
| Nckap1l | honeydew |
| Ppp1r1a | green4 |
| Zfp263 | blue4 |
| Zfp174 | blue3 |
| Naa60 | green4 |
| 1700037C18Rik | blue3 |
| Cluap1 | blue3 |
| Gm15538 | blue3 |
| Gm23493 | blue4 |
| Slx4 | blue4 |
| Gm15879 | darkolivegreen |
| Dnase1 | blue4 |
| Trap1 | mistyrose |
| Crebbp | orangered |
| Adcy9 | blue3 |
| Gm6142 | green4 |
| Tfap4 | darkgrey |
| Glis2 | green4 |
| Pam16 | blue3 |
| Coro7 | deeppink |
| Vasn | darkgrey |
| Dnaja3 | blue3 |
| Nmral1 | blue4 |
| Hmox2 | blue3 |
| Cdip1 | blue4 |
| Gm15835 | darkgrey |
| Ubald1 | coral1 |
| Mgrn1 | green4 |
| Gm16861 | green4 |
| Nudt16l1 | green4 |
| Anks3 | green4 |
| Smim22 | blue4 |
| Rogdi | green4 |
| Glyr1 | blue4 |
| Ubn1 | green4 |
| Ppl | blue4 |
| Nagpa | blue3 |
| AU021092 | blue3 |
| Mir6364 | blue4 |
| Gm23689 | blue4 |
| Alg1 | green4 |
| Fam86 | blue4 |
| Rbfox1 | blue4 |
| Mettl22 | blue4 |
| Abat | blue4 |
| Tmem186 | blue3 |
| Pmm2 | blue3 |
| Carhsp1 | blue4 |
| Usp7 | blue4 |
| 1810013L24Rik | darkgrey |
| Gm22224 | blue3 |
| Emp2 | blue4 |
| Nubp1 | blue4 |
| Dexi | green4 |
| Clec16a | green4 |
| Socs1 | saddlebrown |
| Litaf | blue4 |
| Gm26268 | darkgrey |
| Snn | blue4 |
| Txndc11 | darkgrey |
| Zc3h7a | blue4 |
| Gm23935 | blue4 |
| Rsl1d1 | blue4 |
| Gspt1 | blue4 |
| Mir1945 | blue3 |
| Snx29 | blue4 |
| Cpped1 | blue4 |
| Ercc4 | blue3 |
| Mkl2 | blue4 |
| Parn | blue4 |
| Bfar | coral1 |
| 3110001I22Rik | green4 |
| Rrn3 | blue4 |
| Gm27734 | coral1 |
| Ntan1 | blue4 |
| Pdxdc1 | coral1 |
| Mpv17l | blue4 |
| Marf1 | blue3 |
| Nde1 | blue4 |
| Myh11 | lightcoral |
| Fopnl | blue4 |
| Abcc1 | blue4 |
| Snai2 | blue4 |
| Ube2v2 | coral1 |
| Mcm4 | blue4 |
| Prkdc | blue4 |
| Mzt2 | lightsteelblue |
| Cebpd | darkgrey |
| Spidr | blue4 |
| Pkp2 | blue4 |
| Yars2 | blue4 |
| Dnm1l | blue4 |
| Fgd4 | firebrick2 |
| Vpreb1 | darkolivegreen |
| Top3b | blue3 |
| Ppm1f | blue3 |
| Mapk1 | coral1 |
| 1700056N10Rik | blue4 |
| Ppil2 | blue3 |
| Sdf2l1 | blue4 |
| Ydjc | blue4 |
| Ube2l3 | blue3 |
| Hic2 | darkgrey |
| Tmem191c | darkolivegreen |
| Pi4ka | blue4 |
| Snap29 | coral1 |
| Crkl | blue4 |
| Gm22770 | blue4 |
| Aifm3 | green4 |
| Lztr1 | blue4 |
| Thap7 | deeppink |
| Slc7a4 | deeppink |
| Smpd4 | blue4 |
| Ccdc74a | blue4 |
| Med15 | blue4 |
| Klhl22 | blue4 |
| Scarf2 | blue4 |
| Dgcr14 | blue4 |
| Slc25a1 | firebrick2 |
| Dgcr6 | indianred2 |
| Prodh | blue4 |
| Zdhhc8 | blue4 |
| Ranbp1 | blue4 |
| Trmt2a | blue4 |
| Dgcr8 | blue4 |
| Tango2 | green4 |
| Arvcf | deeppink |
| Comt | blue4 |
| Txnrd2 | deeppink |
| 5-Sep | blue4 |
| Cdc45 | blue4 |
| Ufd1l | coral1 |
| Mrpl40 | blue4 |
| Iglv1 | saddlebrown |
| Klhl6 | green4 |
| Klhl24 | mistyrose |
| Yeats2 | blue4 |
| Gm10241 | blue4 |
| Parl | blue4 |
| Abcc5 | blue3 |
| Gm23332 | darkolivegreen |
| Eif2b5 | blue4 |
| Dvl3 | blue3 |
| Ap2m1 | blue4 |
| Gm15760 | blue4 |
| Abcf3 | green4 |
| Alg3 | blue4 |
| Ece2 | blue4 |
| Camk2n2 | blue4 |
| Psmd2 | blue4 |
| Eif4g1 | blue4 |
| Clcn2 | darkolivegreen |
| Polr2h | blue4 |
| Thpo | blue3 |
| Chrd | green4 |
| Rps10-ps2 | blue4 |
| Ephb3 | blue3 |
| Vps8 | blue4 |
| 2510009E07Rik | blue4 |
| Ehhadh | mistyrose |
| Gm24898 | mistyrose |
| 1300002E11Rik | blue4 |
| Map3k13 | blue3 |
| Gm6467 | blue4 |
| Tmem41a | deeppink |
| Senp2 | darkgrey |
| Igf2bp2 | blue4 |
| Tra2b | darkgrey |
| Gm15776 | blue4 |
| Etv5 | darkolivegreen |
| Dgkg | blue4 |
| Tbccd1 | blue3 |
| Dnajb11 | mistyrose |
| Hrg | blue4 |
| Kng2 | darkolivegreen |
| Eif4a2 | firebrick2 |
| Rfc4 | blue4 |
| Adipoq | lightcoral |
| St6gal1 | blue4 |
| Masp1 | blue4 |
| Rtp4 | saddlebrown |
| Bcl6 | deeppink1 |
| RP23-274H19.2 | blue3 |
| 1110054M08Rik | green4 |
| Lpp | blue4 |
| Gm4524 | darkolivegreen |
| Leprel1 | darkolivegreen |
| Cldn1 | blue4 |
| Cldn16 | blue4 |
| Tmem207 | blue4 |
| Il1rap | blue4 |
| Ccdc50 | blue4 |
| Mb21d2 | mistyrose |
| Opa1 | firebrick2 |
| 4632428C04Rik | darkolivegreen |
| Hes1 | deeppink |
| Atp13a3 | blue4 |
| Tmem44 | blue3 |
| Lsg1 | blue4 |
| Fam43a | darkgrey |
| Xxylt1 | blue4 |
| Acap2 | indianred2 |
| Ppp1r2 | darkgrey |
| Gm27415 | blue3 |
| Bdh1 | mistyrose |
| Dlg1 | blue4 |
| 0610012G03Rik | blue3 |
| Ncbp2 | blue3 |
| Senp5 | blue4 |
| Gm15696 | blue4 |
| Gm15694 | darkgrey |
| Pak2 | blue4 |
| Pigx | darkolivegreen |
| Cep19 | firebrick2 |
| Nrros | darkgrey |
| Fbxo45 | blue4 |
| Wdr53 | mistyrose |
| Rnf168 | blue4 |
| Ubxn7 | mistyrose |
| RP23-123L9.5 | blue4 |
| Tctex1d2 | mistyrose |
| Pcyt1a | green4 |
| Slc51a | blue4 |
| Tfrc | blue4 |
| Tnk2 | blue3 |
| Muc20 | blue3 |
| 1700021K19Rik | blue3 |
| Fyttd1 | coral1 |
| Lrch3 | blue4 |
| Iqcg | darkolivegreen |
| Rpl35a | blue4 |
| Lmln | blue3 |
| Osbpl11 | deeppink |
| Snx4 | coral1 |
| 1700007L15Rik | blue4 |
| Zfp148 | firebrick2 |
| Heg1 | blue4 |
| Itgb5 | coral1 |
| Umps | blue4 |
| Kalrn | darkgrey |
| Mylk | blue4 |
| 1700119H24Rik | blue4 |
| Ptplb | mistyrose |
| Sec22a | antiquewhite2 |
| Pdia5 | blue4 |
| Gm5963 | blue4 |
| Dirc2 | blue4 |
| Hspbap1 | blue4 |
| Parp14 | saddlebrown |
| Dtx3l | blue4 |
| Parp9 | saddlebrown |
| Gm15564 | coral1 |
| Kpna1 | blue4 |
| Wdr5b | coral1 |
| Fam162a | blue4 |
| Ccdc58 | blue4 |
| 2010005H15Rik | blue4 |
| Casr | blue3 |
| Cd86 | blue3 |
| Ildr1 | green4 |
| Slc15a2 | blue4 |
| Eaf2 | blue4 |
| Iqcb1 | brown1 |
| Golgb1 | blue4 |
| Hcls1 | honeydew |
| Fbxo40 | blue4 |
| Stxbp5l | firebrick2 |
| Gtf2e1 | blue4 |
| Rabl3 | blue4 |
| Hgd | blue4 |
| Ndufb4 | blue4 |
| Fstl1 | blue4 |
| Lrrc58 | blue3 |
| Gsk3b | blue4 |
| Nr1i2 | blue4 |
| Cox17 | blue3 |
| Pla1a | blue3 |
| Adprh | blue4 |
| Timmdc1 | blue4 |
| Poglut1 | blue4 |
| Tmem39a | darkgrey |
| Arhgap31 | blue4 |
| B4galt4 | blue4 |
| Upk1b | lightcoral |
| Igsf11 | blue4 |
| Zbtb20 | blue4 |
| BC002163 | lightsteelblue |
| Tigit | blue3 |
| Qtrtd1 | blue4 |
| 2610015P09Rik | firebrick2 |
| Zdhhc23 | blue3 |
| Gramd1c | blue4 |
| Atp6v1a | mistyrose |
| Naa50 | blue4 |
| Gm608 | firebrick2 |
| Spice1 | blue3 |
| Boc | blue3 |
| BC027231 | blue4 |
| Gtpbp8 | blue3 |
| Ccdc80 | lightcoral |
| Slc35a5 | darkgrey |
| Atg3 | deeppink |
| Gm6030 | blue3 |
| Cd200 | blue3 |
| Abhd10 | lightsteelblue |
| Phldb2 | blue3 |
| Gm15638 | blue4 |
| Plcxd2 | darkgrey |
| Gm4737 | mistyrose |
| Pvrl3 | blue4 |
| Gm7204 | blue4 |
| Retnlg | darkgrey |
| Dzip3 | blue3 |
| Ift57 | green4 |
| Cd47 | green4 |
| Bbx | blue4 |
| 5330426P16Rik | blue4 |
| Cblb | darkgrey |
| Alcam | blue4 |
| Zpld1 | mistyrose |
| Mir5118 | blue3 |
| Nfkbiz | darkgrey |
| Nxpe3 | blue4 |
| Cep97 | blue3 |
| Rpl24 | darkgrey |
| 2310061J03Rik | blue4 |
| Zbtb11 | darkgrey |
| Pcnp | coral1 |
| Trmt10c | coral1 |
| Gm22422 | firebrick2 |
| Senp7 | blue3 |
| Abi3bp | darkgrey |
| Tfg | blue4 |
| Tmem45a | blue4 |
| Tomm70a | coral1 |
| Nit2 | blue4 |
| Tbc1d23 | coral1 |
| Cmss1 | blue4 |
| Filip1l | blue4 |
| Col8a1 | blue4 |
| Dcbld2 | blue4 |
| St3gal6 | darkgrey |
| Cpox | blue4 |
| Cldn25 | blue4 |
| Mina | blue4 |
| Crybg3 | blue4 |
| Gm9581 | blue4 |
| Arl6 | mistyrose |
| Gm9816 | blue4 |
| Nsun3 | mistyrose |
| Arl13b | brown1 |
| Pros1 | blue4 |
| 4930453N24Rik | blue4 |
| Zfp654 | coral1 |
| Cggbp1 | blue4 |
| Gm23374 | blue4 |
| Chmp2b | blue4 |
| Vgll3 | blue4 |
| Gbe1 | blue4 |
| Robo1 | blue4 |
| Robo2 | brown1 |
| Hspa13 | coral1 |
| Samsn1 | darkgrey |
| Nrip1 | darkgrey |
| Gm9843 | blue4 |
| Usp25 | coral1 |
| Mir99ahg | lightsteelblue |
| Mir99a | blue3 |
| Cxadr | blue4 |
| Btg3 | darkgrey |
| D16Ertd472e | blue4 |
| Gm26667 | blue4 |
| Mrpl39 | coral1 |
| Jam2 | blue4 |
| Gm25908 | brown1 |
| Atp5j | mistyrose |
| Gabpa | firebrick2 |
| App | blue4 |
| Cyyr1 | blue4 |
| Adamts1 | darkgrey |
| Adamts5 | blue4 |
| N6amt1 | coral1 |
| Ltn1 | firebrick2 |
| Rwdd2b | blue3 |
| Usp16 | coral1 |
| Cct8 | blue4 |
| Map3k7cl | darkolivegreen |
| Bach1 | darkgrey |
| Cldn8 | darkolivegreen |
| Tiam1 | blue4 |
| Sod1 | blue4 |
| Scaf4 | blue3 |
| Hunk | green4 |
| Mis18a | blue4 |
| Mrap | lightcoral |
| Urb1 | blue4 |
| Eva1c | darkgrey |
| 1110004E09Rik | blue4 |
| Synj1 | blue4 |
| Gm22933 | blue3 |
| Paxbp1 | deeppink1 |
| H3f3a-ps2 | blue4 |
| Ifnar2 | blue4 |
| Il10rb | blue3 |
| A930006K02Rik | blue3 |
| Ifnar1 | blue4 |
| Ifngr2 | blue4 |
| Tmem50b | blue3 |
| Dnajc28 | mistyrose |
| Gart | blue4 |
| Son | blue4 |
| Donson | blue4 |
| Atp5o | blue4 |
| Cryzl1 | blue3 |
| Itsn1 | blue4 |
| Mrps6 | blue4 |
| Slc5a3 | blue4 |
| Gm16310 | blue4 |
| Smim11 | blue3 |
| 1700048M11Rik | blue3 |
| Kcne1 | green4 |
| Rcan1 | darkgrey |
| Runx1 | blue4 |
| Setd4 | blue4 |
| Cbr1 | blue4 |
| Cbr3 | blue4 |
| Dopey2 | blue3 |
| Morc3 | blue4 |
| Chaf1b | blue4 |
| Sim2 | darkgrey |
| Hlcs | blue4 |
| Ripply3 | blue4 |
| Pigp | blue4 |
| Ttc3 | blue3 |
| Dscr3 | blue4 |
| Dyrk1a | darkgrey |
| Kcnj15 | blue3 |
| Erg | blue4 |
| Ets2 | darkgrey |
| Psmg1 | blue3 |
| Brwd1 | blue4 |
| Hmgn1 | blue4 |
| Wrb | blue4 |
| B3galt5 | darkolivegreen |
| Igsf5 | darkolivegreen |
| Itgb2l | blue3 |
| Bace2 | blue4 |
| Tmprss2 | blue3 |
| Ripk4 | darkgrey |
| Prdm15 | blue3 |
| C2cd2 | blue4 |
| Zbtb21 | darkgrey |
| Gm10232 | blue4 |
| Pisd-ps2 | blue3 |
| Scaf8 | darkgrey |
| Tiam2 | blue4 |
| Tfb1m | blue3 |
| Arid1b | blue3 |
| Tmem242 | blue4 |
| Zdhhc14 | darkgrey |
| 3300005D01Rik | blue4 |
| Gm26622 | darkolivegreen |
| Snx9 | blue4 |
| Synj2 | blue4 |
| Serac1 | blue3 |
| Gtf2h5 | antiquewhite2 |
| Tulp4 | firebrick2 |
| Gm15590 | blue4 |
| Tmem181a | blue4 |
| Dynlt1a | blue4 |
| Dynlt1b | antiquewhite2 |
| Tmem181b-ps | blue3 |
| Dynlt1f | blue4 |
| Ezr | darkgrey |
| Mir692-1 | blue4 |
| Rsph3b | blue4 |
| Tagap1 | darkolivegreen |
| Rnaset2b | saddlebrown |
| Rps6ka2 | mistyrose |
| Rsph3a | green4 |
| Rnaset2a | coral1 |
| Fgfr1op | green4 |
| Mpc1 | blue3 |
| 4930506C21Rik | antiquewhite2 |
| Sft2d1 | blue4 |
| Pde10a | brown1 |
| Qk | blue4 |
| Pacrg | blue4 |
| Park2 | mistyrose |
| Agpat4 | blue4 |
| Map3k4 | blue3 |
| 4732491K20Rik | blue3 |
| Slc22a2 | blue4 |
| Slc22a1 | blue4 |
| Igf2r | lightsteelblue |
| Airn | blue3 |
| Gm16574 | blue3 |
| Mrpl18 | blue3 |
| Tcp1 | blue4 |
| Snora20 | blue4 |
| Acat3 | blue4 |
| Acat2 | blue4 |
| Wtap | coral1 |
| Sod2 | blue4 |
| Mllt4 | blue4 |
| Dact2 | coral1 |
| Smoc2 | blue3 |
| Thbs2 | blue4 |
| Gm3222 | lightsteelblue |
| 1600012H06Rik | blue4 |
| Phf10 | blue3 |
| Ermard | blue3 |
| Dll1 | coral1 |
| Fam120b | blue3 |
| Psmb1 | orangered |
| Prdm9 | blue3 |
| Chd1 | blue4 |
| Rgmb | blue4 |
| BC002059 | blue4 |
| Zfp960 | coral1 |
| Zfp97 | mistyrose |
| Gm6712 | coral1 |
| Oaz1-ps | blue4 |
| Riok2 | green4 |
| Gm26873 | darkgrey |
| Lix1 | blue4 |
| Lnpep | blue4 |
| Gm25927 | blue4 |
| Spaca6 | blue3 |
| Has1 | darkgrey |
| Fpr2 | blue4 |
| Ppp2r1a | mistyrose |
| Zfp160 | coral1 |
| Gm26384 | coral1 |
| Zfp677 | blue4 |
| Zfp54 | blue4 |
| Zfp51 | blue4 |
| Zfp53 | blue4 |
| 9330136K24Rik | blue4 |
| Zfp52 | blue4 |
| Zfp948 | blue4 |
| 3110052M02Rik | blue3 |
| Gm10509 | blue4 |
| Gm10226 | saddlebrown |
| Zfp760 | blue3 |
| Zfp229 | coral1 |
| 2210404O09Rik | blue4 |
| Zfp942 | coral1 |
| Zfp943 | coral1 |
| Zfp947 | blue4 |
| Gm4944 | coral1 |
| Zfp944 | coral1 |
| Zfp758 | coral1 |
| Zfp946 | coral1 |
| Gm24606 | darkolivegreen |
| Gm5224 | lightsteelblue |
| Zfp945 | coral1 |
| Zfp40 | coral1 |
| Zfp13 | blue4 |
| Ccdc64b | blue4 |
| Thoc6 | darkgrey |
| Hcfc1r1 | coral1 |
| Tnfrsf12a | darkgrey |
| Cldn6 | coral1 |
| Pkmyt1 | blue4 |
| Paqr4 | blue4 |
| 9530082P21Rik | green4 |
| Flywch1 | green4 |
| Flywch2 | blue4 |
| Srrm2 | blue3 |
| Mir5125 | blue3 |
| Tceb2 | blue4 |
| Prss22 | blue4 |
| Kctd5 | blue4 |
| Pdpk1 | blue4 |
| Amdhd2 | blue4 |
| Atp6v0c | coral1 |
| Tbc1d24 | blue3 |
| 1600002H07Rik | blue4 |
| Abca3 | coral1 |
| Gm25618 | blue4 |
| D330041H03Rik | blue3 |
| Rnps1 | blue4 |
| Eci1 | mistyrose |
| Dnase1l2 | blue3 |
| E4f1 | blue3 |
| Pgp | blue4 |
| Mlst8 | blue4 |
| Traf7 | blue4 |
| Rab26os | blue4 |
| Pkd1 | blue3 |
| Tsc2 | green4 |
| Nthl1 | blue3 |
| Slc9a3r2 | blue3 |
| Zfp598 | blue3 |
| Gfer | blue4 |
| Tbl3 | blue4 |
| Rps2 | blue4 |
| Snora78 | blue4 |
| Ndufb10 | green4 |
| Rpl3l | blue4 |
| Msrb1 | blue3 |
| Hs3st6 | blue4 |
| Hagh | coral1 |
| Fahd1 | firebrick2 |
| Igfals | green4 |
| Nubp2 | blue4 |
| Spsb3 | coral1 |
| Eme2 | blue3 |
| Mapk8ip3 | blue3 |
| Mrps34 | coral1 |
| Hn1l | blue4 |
| Cramp1l | darkgrey |
| Ift140 | green4 |
| Tmem204 | deeppink1 |
| Telo2 | blue3 |
| Clcn7 | green4 |
| BC003965 | blue3 |
| Unkl | darkolivegreen |
| Gnptg | blue4 |
| Tsr3 | blue3 |
| Baiap3 | blue4 |
| Ube2i | blue4 |
| Lmf1 | green4 |
| Rpusd1 | blue4 |
| Msln | blue4 |
| Narfl | brown1 |
| Haghl | green4 |
| Fam173a | blue4 |
| Fbxl16 | blue3 |
| Wdr24 | green4 |
| Jmjd8 | coral1 |
| Stub1 | blue3 |
| Rhbdl1 | blue3 |
| Rhot2 | green4 |
| Wdr90 | blue3 |
| Fam195a | blue4 |
| 0610011F06Rik | blue4 |
| Rab40c | blue3 |
| Pigq | blue4 |
| Nhlrc4 | blue3 |
| A930017K11Rik | blue3 |
| D630044L22Rik | blue3 |
| Capn15 | blue3 |
| 1700022N22Rik | blue3 |
| Rab11fip3 | green4 |
| Decr2 | mistyrose |
| Nme4 | deeppink |
| Gm8186 | blue4 |
| Tmem8 | blue4 |
| Mrpl28 | blue4 |
| Axin1 | deeppink |
| Arhgdig | blue4 |
| Rgs11 | darkolivegreen |
| Itfg3 | deeppink |
| Gm23123 | lightsteelblue |
| Luc7l | blue4 |
| Neurl1b | blue4 |
| Dusp1 | coral1 |
| Ergic1 | blue4 |
| Atp6v0e | lightsteelblue |
| Crebrf | mistyrose |
| Bnip1 | blue3 |
| Phf1 | green4 |
| Cuta | deeppink |
| Syngap1 | blue3 |
| Ggnbp1 | darkolivegreen |
| Bak1 | blue4 |
| Itpr3 | blue4 |
| Uqcc2 | blue4 |
| Lemd2 | deeppink |
| Gm26724 | blue3 |
| Hmga1 | blue4 |
| AI413582 | blue4 |
| Nudt3 | blue3 |
| Rps10 | blue4 |
| D17Wsu92e | darkgrey |
| Snrpc | blue4 |
| Uhrf1bp1 | blue4 |
| Taf11 | blue4 |
| Anks1 | green4 |
| Scube3 | green4 |
| Zfp523 | green4 |
| Def6 | blue4 |
| Ppard | coral1 |
| Fance | blue4 |
| Rpl10a | blue4 |
| Tead3 | blue3 |
| Fkbp5 | blue3 |
| Srpk1 | blue3 |
| Mapk14 | blue4 |
| Mapk13 | blue4 |
| Brpf3 | green4 |
| Kctd20 | blue4 |
| Stk38 | blue3 |
| Srsf3 | green4 |
| Cdkn1a | darkgrey |
| Rpl35a-ps3 | orangered |
| Ppil1 | blue4 |
| BC004004 | blue4 |
| Pi16 | blue3 |
| Mtch1 | coral1 |
| Fgd2 | saddlebrown |
| Pim1 | darkgrey |
| Tbc1d22b | green4 |
| Ccdc167 | blue3 |
| Zfand3 | mistyrose |
| Btbd9 | blue4 |
| Glo1 | coral1 |
| Abcg1 | blue3 |
| Gm15318 | blue4 |
| Tff1 | blue3 |
| Rsph1 | blue4 |
| Pde9a | darkolivegreen |
| Wdr4 | blue3 |
| Ndufv3 | blue4 |
| Pknox1 | blue3 |
| Gm24970 | blue3 |
| Cbs | blue4 |
| U2af1 | blue4 |
| Cryaa | blue4 |
| Sik1 | coral1 |
| Hsf2bp | blue4 |
| Rrp1b | blue4 |
| Notch3 | blue4 |
| Brd4 | mistyrose |
| Gm26549 | blue3 |
| Akap8 | blue3 |
| Akap8l | blue3 |
| Wiz | blue3 |
| Cyp4f17 | blue4 |
| Cyp4f16 | blue4 |
| Zfp871 | firebrick2 |
| Gm17115 | blue4 |
| Zfp799 | firebrick2 |
| Zfp870 | coral1 |
| Cyp4f14 | blue4 |
| Cyp4f13 | green4 |
| Zfp472 | coral1 |
| Zfp952 | coral1 |
| Zfp763 | coral1 |
| Zfp563 | coral1 |
| Zfp955a | coral1 |
| Zfp955b | coral1 |
| Zfp422-rs1 | mistyrose |
| Zfp81 | blue4 |
| Zfp101 | firebrick2 |
| Adamts10 | blue3 |
| Myo1f | blue4 |
| Zfp414 | blue4 |
| Hnrnpm | blue4 |
| 2-Mar | blue4 |
| Rab11b | mistyrose |
| Gm17251 | blue4 |
| Angptl4 | blue3 |
| Kank3 | blue3 |
| Rps28 | blue4 |
| Ndufa7 | indianred2 |
| Cd320 | deeppink |
| Kifc1 | blue4 |
| BC051226 | blue3 |
| Daxx | blue4 |
| Tapbp | saddlebrown |
| Rgl2 | blue3 |
| Pfdn6 | blue4 |
| Wdr46 | blue4 |
| Rps18 | blue4 |
| Vps52 | green4 |
| H2-K2 | blue4 |
| H2-K1 | saddlebrown |
| Ring1 | green4 |
| Rxrb | brown1 |
| Brd2 | darkgrey |
| H2-DMa | saddlebrown |
| H2-DMb1 | saddlebrown |
| Psmb9 | saddlebrown |
| Tap1 | saddlebrown |
| Psmb8 | saddlebrown |
| Tap2 | saddlebrown |
| H2-Ab1 | saddlebrown |
| H2-Aa | saddlebrown |
| H2-Eb1 | saddlebrown |
| Notch4 | blue4 |
| Gpsm3 | blue4 |
| Pbx2 | blue3 |
| Rnf5 | blue4 |
| Agpat1 | blue3 |
| Atf6b | blue3 |
| Tnxb | blue4 |
| C4b | saddlebrown |
| Stk19 | darkgrey |
| Dxo | mistyrose |
| Skiv2l | green4 |
| Nelfe | blue4 |
| C2 | blue4 |
| Ehmt2 | green4 |
| Slc44a4 | blue4 |
| Neu1 | green4 |
| 1110038B12Rik | mistyrose |
| Lsm2 | blue4 |
| Vars | blue4 |
| Clic1 | blue4 |
| Ddah2 | blue4 |
| Ly6g6d | blue3 |
| Abhd16a | blue4 |
| Csnk2b | deeppink |
| Apom | blue4 |
| Bag6 | deeppink |
| Prrc2a | blue3 |
| Aif1 | saddlebrown |
| Lst1 | saddlebrown |
| Ddx39b | blue4 |
| H2-D1 | saddlebrown |
| H2-Q4 | saddlebrown |
| H2-Q5 | saddlebrown |
| H2-Q6 | saddlebrown |
| H2-Q7 | saddlebrown |
| Tcf19 | blue4 |
| Cchcr1 | blue3 |
| Vars2 | green4 |
| Gtf2h4 | blue3 |
| Ddr1 | mistyrose |
| Ier3 | darkgrey |
| Flot1 | blue4 |
| Tubb5 | blue4 |
| Mdc1 | blue4 |
| Nrm | blue4 |
| Ppp1r18 | blue4 |
| Dhx16 | blue3 |
| 2310061I04Rik | blue3 |
| Atat1 | green4 |
| Mrps18b | blue4 |
| Ppp1r10 | green4 |
| Gm9840 | blue4 |
| Abcf1 | blue3 |
| Prr3 | blue3 |
| Gnl1 | darkgrey |
| H2-T24 | blue3 |
| H2-T23 | saddlebrown |
| H2-T22 | saddlebrown |
| Gm11127 | blue4 |
| H2-Bl | blue4 |
| C920025E04Rik | saddlebrown |
| Rpp21 | blue4 |
| Trim39 | deeppink1 |
| Trim26 | blue3 |
| Ppp1r11 | deeppink |
| Znrd1 | lightsteelblue |
| Gabbr1 | darkolivegreen |
| Gm26917 | coral1 |
| Yam1 | blue4 |
| 9130008F23Rik | darkgrey |
| Cenpq | coral1 |
| Mut | mistyrose |
| Cd2ap | blue4 |
| Tnfrsf21 | blue3 |
| Gpr116 | brown1 |
| Gm25135 | darkgrey |
| Mep1a | blue4 |
| Pla2g7 | blue3 |
| Slc25a27 | blue3 |
| Cyp39a1 | mistyrose |
| Rcan2 | firebrick2 |
| Enpp5 | blue4 |
| Enpp4 | coral1 |
| Clic5 | blue3 |
| Supt3 | blue3 |
| Cdc5l | blue4 |
| B230354K17Rik | coral1 |
| Aars2 | green4 |
| Nfkbie | blue4 |
| Slc35b2 | deeppink1 |
| Hsp90ab1 | darkgrey |
| Slc29a1 | deeppink |
| Gm7325 | coral1 |
| Tmem63b | blue4 |
| Gm16172 | blue3 |
| Mrpl14 | deeppink |
| Vegfa | green4 |
| Mrps18a | deeppink |
| Rsph9 | blue4 |
| Mad2l1bp | blue4 |
| Gtpbp2 | blue3 |
| Polh | blue4 |
| Xpo5 | blue4 |
| Polr1c | firebrick2 |
| Yipf3 | deeppink |
| Tjap1 | mistyrose |
| Abcc10 | blue3 |
| Zfp318 | blue3 |
| Crip3 | darkolivegreen |
| Slc22a7 | blue4 |
| Gm5093 | blue4 |
| Ttbk1 | coral1 |
| Dnph1 | blue4 |
| Cul9 | blue3 |
| Srf | coral1 |
| Ptk7 | blue4 |
| Klc4 | coral1 |
| Mrpl2 | blue4 |
| Cul7 | blue3 |
| Rrp36 | blue3 |
| Klhdc3 | coral1 |
| Mea1 | deeppink |
| Ppp2r5d | blue3 |
| Pex6 | green4 |
| Gnmt | blue4 |
| Cnpy3 | blue3 |
| 2310039H08Rik | blue4 |
| Rpl7l1 | blue4 |
| Gltscr1l | blue3 |
| Tbcc | darkgrey |
| Ubr2 | lightsteelblue |
| Trerf1 | blue3 |
| Mrps10 | blue4 |
| Guca1b | blue3 |
| Gm5814 | blue4 |
| AI661453 | coral1 |
| Taf8 | darkolivegreen |
| Ccnd3 | deeppink |
| Bysl | blue4 |
| Med20 | blue3 |
| Usp49 | blue4 |
| Frs3 | darkolivegreen |
| Frs3os | blue3 |
| Tfeb | blue4 |
| Mdfi | darkgrey |
| Foxp4 | mistyrose |
| Nfya | blue4 |
| Oard1 | coral1 |
| Apobec2 | blue4 |
| Mocs1 | blue4 |
| Daam2 | coral1 |
| Rftn1 | blue4 |
| Plcl2 | blue4 |
| Gm7334 | blue4 |
| Tbc1d5 | blue3 |
| Rab5a | brown1 |
| Kat2b | mistyrose |
| Sult1c2 | darkolivegreen |
| Mrps36-ps1 | blue4 |
| Pot1b | blue3 |
| Zfp119a | coral1 |
| Zfp959 | coral1 |
| Zfp119b | deeppink |
| Ccdc94 | blue4 |
| Shd | darkolivegreen |
| Stap2 | blue4 |
| Mpnd | green4 |
| Gm16276 | blue4 |
| Sh3gl1 | blue4 |
| Chaf1a | blue4 |
| Ubxn6 | coral1 |
| Hdgfrp2 | blue3 |
| Plin4 | lightcoral |
| Plin5 | mistyrose |
| Lrg1 | lightcoral |
| Sema6b | darkgrey |
| Tnfaip8l1 | mistyrose |
| D17Wsu104e | firebrick2 |
| Dpp9 | blue3 |
| Fem1a | brown1 |
| Ticam1 | coral1 |
| Plin3 | blue3 |
| Uhrf1 | blue4 |
| Kdm4b | green4 |
| Ptprs | blue3 |
| Safb2 | blue3 |
| Safb | blue3 |
| 2410015M20Rik | blue4 |
| Rpl36 | blue4 |
| Lonp1 | blue4 |
| Ranbp3 | blue4 |
| Vmac | green4 |
| Ndufa11 | blue4 |
| Nrtn | blue4 |
| Dus3l | blue3 |
| Rfx2 | mistyrose |
| Mllt1 | blue3 |
| Clpp | coral1 |
| Alkbh7 | green4 |
| Gtf2f1 | blue4 |
| Khsrp | blue3 |
| Gm17168 | blue3 |
| Slc25a23 | green4 |
| Crb3 | darkgrey |
| Dennd1c | darkgrey |
| Tubb4a | mistyrose |
| C3 | blue4 |
| Gpr108 | deeppink |
| Trip10 | blue4 |
| Emr1 | blue4 |
| Nudt12 | mistyrose |
| Efna5 | blue4 |
| Fbxl17 | mistyrose |
| Fer | blue4 |
| Pja2 | coral1 |
| Man2a1 | blue4 |
| Gm17133 | blue4 |
| Vapa | blue4 |
| Rab31 | blue4 |
| Ppp4r1 | blue4 |
| Ralbp1 | blue4 |
| Twsg1 | blue4 |
| Ankrd12 | blue4 |
| Ndufv2 | blue4 |
| Gm23264 | blue3 |
| Wash | green4 |
| Ddx11 | blue4 |
| Mtcl1 | blue3 |
| Rab12 | blue4 |
| Ptprm | blue4 |
| Lama1 | green4 |
| Arhgap28 | blue4 |
| Epb4.1l3 | blue4 |
| Zbtb14 | coral1 |
| Gm20703 | blue3 |
| Tgif1 | darkgrey |
| Gm9320 | blue4 |
| Myl12b | blue3 |
| Myl12a | blue4 |
| Lpin2 | brown1 |
| Emilin2 | darkgrey |
| Smchd1 | blue4 |
| Ndc80 | blue4 |
| Trmt61b | darkgrey |
| Wdr43 | blue4 |
| Snord53 | deeppink1 |
| Gm22858 | darkgrey |
| Clip4 | blue4 |
| Ypel5 | green4 |
| Lbh | blue4 |
| Lclat1 | coral1 |
| Galnt14 | blue4 |
| Ehd3 | coral1 |
| Xdh | blue4 |
| Srd5a2 | blue3 |
| Memo1 | blue4 |
| Dpy30 | blue4 |
| Spast | blue4 |
| Slc30a6 | blue3 |
| Nlrc4 | deeppink |
| Yipf4 | mistyrose |
| Birc6 | firebrick2 |
| Ttc27 | blue4 |
| Ltbp1 | mistyrose |
| Rasgrp3 | lightsteelblue |
| Fam98a | blue4 |
| Gm17538 | blue4 |
| Crim1 | blue4 |
| Fez2 | blue4 |
| Strn | blue4 |
| Heatr5b | blue4 |
| Gpatch11 | blue3 |
| Gm6548 | blue4 |
| Eif2ak2 | blue4 |
| n-R5s29 | blue4 |
| Cebpzos | blue4 |
| Cebpz | blue4 |
| Ndufaf7 | coral1 |
| Prkd3 | firebrick2 |
| Cdc42ep3 | blue3 |
| Rmdn2 | blue4 |
| Cyp1b1 | blue4 |
| Atl2 | green4 |
| Hnrnpll | blue4 |
| Galm | darkolivegreen |
| Srsf7 | blue4 |
| Gemin6 | blue4 |
| Dhx57 | blue3 |
| Morn2 | blue4 |
| Sos1 | firebrick2 |
| Map4k3 | blue4 |
| Gm9959 | blue3 |
| Tmem178 | blue4 |
| Thumpd2 | blue4 |
| Slc8a1 | darkolivegreen |
| Pkdcc | blue4 |
| Eml4 | blue4 |
| Cox7a2l | blue4 |
| Mta3 | blue3 |
| Haao | blue4 |
| Zfp36l2 | blue4 |
| Thada | blue4 |
| Plekhh2 | blue4 |
| Dync2li1 | coral1 |
| Lrpprc | blue4 |
| 1110020A21Rik | blue4 |
| Ppm1b | firebrick2 |
| Slc3a1 | blue4 |
| Prepl | darkolivegreen |
| Camkmt | blue4 |
| Rpl31-ps16 | blue4 |
| Srbd1 | blue3 |
| Prkce | lightsteelblue |
| Epas1 | mistyrose |
| Rhoq | blue4 |
| Pigf | blue4 |
| Cript | mistyrose |
| Socs5 | blue3 |
| Mcfd2 | blue4 |
| 4833418N02Rik | darkolivegreen |
| Ttc7 | green4 |
| Calm2 | darkolivegreen |
| Epcam | blue4 |
| Msh2 | blue4 |
| Msh6 | blue4 |
| Fbxo11 | blue4 |
| Foxn2 | blue4 |
| Ppp1r21 | lightsteelblue |
| Ston1 | blue4 |
| Mettl4 | blue3 |
| Gm1976 | blue4 |
| Gm20939 | blue3 |
| Crem | blue4 |
| Cul2 | blue4 |
| Bambi | blue4 |
| Gm10557 | blue4 |
| Map3k8 | darkgrey |
| Mtpap | blue4 |
| 9430020K01Rik | blue4 |
| Svil | blue3 |
| Zeb1 | blue4 |
| Arhgap12 | blue4 |
| Kif5b | blue4 |
| Epc1 | darkgrey |
| Gm28529 | darkgrey |
| Rab18 | coral1 |
| Mpp7 | coral1 |
| Wac | brown1 |
| Fzd8 | mistyrose |
| Ccny | green4 |
| Colec12 | blue4 |
| Thoc1 | blue4 |
| Usp14 | blue4 |
| Rock1 | blue4 |
| Greb1l | blue3 |
| Esco1 | coral1 |
| Snrpd1 | blue4 |
| Abhd3 | blue4 |
| Mib1 | blue4 |
| Gata6 | mistyrose |
| Rbbp8 | blue4 |
| Cables1 | blue4 |
| Tmem241 | blue3 |
| Riok3 | blue4 |
| 3110002H16Rik | blue4 |
| Npc1 | blue4 |
| Ankrd29 | coral1 |
| Lama3 | blue4 |
| Gm29201 | blue4 |
| Ttc39c | mistyrose |
| Osbpl1a | darkolivegreen |
| Impact | brown1 |
| Zfp521 | blue3 |
| Ss18 | blue4 |
| Taf4b | blue4 |
| Kctd1 | blue4 |
| Gm6304 | blue4 |
| Aqp4 | blue3 |
| Chst9 | darkolivegreen |
| Gm10036 | blue4 |
| Gm7665 | blue4 |
| Cdh2 | blue4 |
| Dsc2 | blue3 |
| Dsg2 | blue4 |
| Ttr | blue4 |
| Gm10269 | blue4 |
| B4galt6 | darkgrey |
| Trappc8 | blue4 |
| Gm24959 | darkolivegreen |
| Rnf125 | darkgrey |
| Rnf138 | brown1 |
| Mep1b | blue4 |
| Garem | blue3 |
| Klhl14 | blue3 |
| 4930426D05Rik | darkolivegreen |
| Dtna | blue4 |
| Gm15972 | blue4 |
| Mapre2 | darkgrey |
| Zfp397 | blue3 |
| Zscan30 | blue3 |
| Zfp35 | coral1 |
| Zfp191 | mistyrose |
| Ino80c | blue4 |
| Galnt1 | blue3 |
| 2700062C07Rik | blue4 |
| Rprd1a | blue3 |
| Slc39a6 | blue4 |
| Elp2 | coral1 |
| Mocos | saddlebrown |
| Fhod3 | orangered |
| Tpgs2 | blue3 |
| AW554918 | blue4 |
| Pik3c3 | darkgrey |
| Gm7936 | orangered |
| Slc25a46 | coral1 |
| Sap130 | blue3 |
| Ammecr1l | darkgrey |
| Polr2d | blue4 |
| Wdr33 | coral1 |
| Gm6665 | blue4 |
| Sft2d3 | coral1 |
| Lims2 | blue4 |
| Myo7b | deeppink |
| Iws1 | blue4 |
| Proc | blue4 |
| Map3k2 | darkgrey |
| Ercc3 | blue4 |
| Bin1 | blue4 |
| Gypc | blue4 |
| Wdr36 | blue4 |
| Stard4 | blue3 |
| Nrep | blue3 |
| 2410004N09Rik | brown1 |
| Gm23639 | blue4 |
| Epb4.1l4a | blue3 |
| Apc | blue4 |
| Srp19 | blue4 |
| Reep5 | blue4 |
| Pkd2l2 | blue3 |
| Fam13b | blue4 |
| Nme5 | blue4 |
| Brd8 | blue3 |
| Cdc23 | blue4 |
| Fam53c | coral1 |
| Kdm3b | blue4 |
| Egr1 | coral1 |
| Etf1 | brown1 |
| Hspa9 | coral1 |
| Gm22200 | blue3 |
| Gm26109 | darkgrey |
| Ctnna1 | blue4 |
| Sil1 | coral1 |
| Snhg4 | blue4 |
| Matr3 | coral1 |
| Paip2 | mistyrose |
| Slc23a1 | darkolivegreen |
| Mzb1 | darkgrey |
| Prob1 | blue4 |
| Spata24 | coral1 |
| Dnajc18 | darkgrey |
| Ecscr | blue4 |
| Tmem173 | blue4 |
| Ube2d2a | blue4 |
| Cxxc5 | blue3 |
| Gm29417 | green4 |
| Pura | blue3 |
| Cystm1 | firebrick2 |
| Pfdn1 | coral1 |
| Hbegf | darkgrey |
| Slc4a9 | darkolivegreen |
| Ankhd1 | blue4 |
| Gm6542 | blue4 |
| Eif4ebp3 | mistyrose |
| Sra1 | blue3 |
| Slc35a4 | blue4 |
| Cd14 | blue4 |
| Tmco6 | blue3 |
| Ndufa2 | blue3 |
| Ik | coral1 |
| Wdr55 | blue4 |
| Hars | blue4 |
| Hars2 | blue3 |
| Zmat2 | green4 |
| Vaultrc5 | coral1 |
| Taf7 | darkgrey |
| Diap1 | darkgrey |
| Hdac3 | blue3 |
| Fchsd1 | coral1 |
| Arap3 | blue3 |
| Pcdh1 | deeppink |
| 0610009O20Rik | green4 |
| Pcdh12 | brown1 |
| Rnf14 | coral1 |
| Gm23205 | blue4 |
| Gnpda1 | blue3 |
| Ndfip1 | blue4 |
| Spry4 | darkgrey |
| Arhgap26 | blue4 |
| Gm19774 | blue4 |
| Fgf1 | darkolivegreen |
| Nr3c1 | firebrick2 |
| Yipf5 | blue4 |
| Prelid2 | blue4 |
| Lars | blue4 |
| Rbm27 | blue4 |
| Tcerg1 | blue4 |
| Ppp2r2b | darkolivegreen |
| Gm8181 | blue4 |
| Stk32a | coral1 |
| Dpysl3 | blue4 |
| Spink3 | blue4 |
| Npy6r | coral1 |
| Dcp2 | blue4 |
| Ythdc2 | blue4 |
| Pggt1b | coral1 |
| Ccdc112 | blue4 |
| Mospd4 | blue4 |
| Fem1c | blue4 |
| Tmed7 | green4 |
| Eif1a | blue4 |
| Cdo1 | blue4 |
| Atg12 | green4 |
| Ap3s1 | blue4 |
| Commd10 | blue4 |
| Sema6a | darkgrey |
| Eno1b | blue4 |
| Gm16283 | darkolivegreen |
| Dmxl1 | firebrick2 |
| Tnfaip8 | darkolivegreen |
| C030005K06Rik | darkolivegreen |
| Hsd17b4 | coral1 |
| Cd63-ps | blue4 |
| Gm4950 | blue4 |
| Srfbp1 | blue4 |
| Lox | blue4 |
| Snx2 | coral1 |
| Snx24 | blue4 |
| Ppic | mistyrose |
| Cep120 | brown1 |
| Csnk1g3 | deeppink |
| Zfp608 | blue4 |
| Gramd3 | mistyrose |
| Aldh7a1 | blue4 |
| Mir1258 | blue3 |
| Phax | deeppink |
| Lmnb1 | blue4 |
| 3-Mar | blue4 |
| C330018D20Rik | mistyrose |
| Prrc1 | blue4 |
| 4930511M06Rik | blue4 |
| Ctxn3 | lightcoral |
| Slc12a2 | indianred2 |
| Isoc1 | coral1 |
| Iigp1 | saddlebrown |
| Smim3 | darkgrey |
| Dctn4 | blue4 |
| Rbm22 | blue4 |
| Synpo | blue4 |
| Ndst1 | blue3 |
| Rps14 | blue4 |
| Cd74 | saddlebrown |
| Mir5107 | blue4 |
| Tcof1 | blue4 |
| Pdgfrb | blue4 |
| Csf1r | blue4 |
| Hmgxb3 | brown1 |
| Gm26403 | blue3 |
| Slc26a2 | firebrick2 |
| Pde6a | blue4 |
| Rps2-ps10 | blue4 |
| Ppargc1b | orangered |
| Gm25301 | darkgrey |
| Arhgef37 | green4 |
| Csnk1a1 | blue4 |
| Gm20748 | coral1 |
| Mir145a | blue4 |
| Pcyox1l | blue3 |
| Grpel2 | deeppink |
| 1500015A07Rik | darkgrey |
| Afap1l1 | blue4 |
| Ablim3 | blue4 |
| Sh3tc2 | blue4 |
| Adrb2 | coral1 |
| Gm9949 | blue4 |
| Fbxo38 | green4 |
| Apcdd1 | blue4 |
| Napg | blue4 |
| Txnl1 | green4 |
| Wdr7 | firebrick2 |
| Fech | mistyrose |
| Nars | blue4 |
| Atp8b1 | blue4 |
| Nedd4l | blue4 |
| Malt1 | deeppink |
| Zfp532 | blue3 |
| Sec11c | blue4 |
| Lman1 | blue4 |
| Ccbe1 | blue4 |
| Pmaip1 | darkgrey |
| Mppe1 | darkolivegreen |
| Impa2 | blue3 |
| Cidea | lightcoral |
| Tubb6 | darkgrey |
| Afg3l2 | coral1 |
| Spire1 | coral1 |
| Cep76 | blue4 |
| Psmg2 | blue4 |
| Ptpn2 | blue4 |
| Gm26910 | blue4 |
| Seh1l | blue4 |
| Cep192 | blue4 |
| Ldlrad4 | blue4 |
| Fam210a | mistyrose |
| Rnmt | green4 |
| Tcf4 | blue4 |
| Ccdc68 | blue4 |
| Rab27b | blue4 |
| 4930503L19Rik | blue4 |
| Poli | darkolivegreen |
| Mbd2 | blue4 |
| Mex3c | blue4 |
| Smad4 | blue4 |
| Elac1 | coral1 |
| Me2 | blue4 |
| Mapk4 | blue4 |
| Cxxc1 | blue3 |
| Mbd1 | blue3 |
| Myo5b | blue4 |
| Scarna17 | blue3 |
| Gm23119 | green4 |
| Acaa2 | blue4 |
| Lipg | coral1 |
| Rpl17 | blue4 |
| Snord58b | darkgrey |
| Gm23301 | darkgrey |
| Gm26202 | darkgrey |
| BC031181 | blue4 |
| Dym | deeppink |
| Gm20544 | darkolivegreen |
| Smad7 | coral1 |
| Ctif | green4 |
| Smad2 | blue4 |
| Ier3ip1 | blue4 |
| Hdhd2 | darkolivegreen |
| Pias2 | firebrick2 |
| 8030462N17Rik | green4 |
| Haus1 | blue4 |
| Atp5a1 | blue4 |
| Pstpip2 | deeppink |
| Epg5 | blue3 |
| Slc14a1 | blue3 |
| Slc14a2 | green4 |
| Setbp1 | blue4 |
| Pard6g | blue4 |
| Adnp2 | blue4 |
| Rbfa | blue4 |
| Gm16286 | blue4 |
| Txnl4a | blue4 |
| Hsbp1l1 | blue4 |
| Pqlc1 | darkgrey |
| Ctdp1 | darkgrey |
| Nfatc1 | blue4 |
| Atp9b | blue3 |
| Sall3 | blue3 |
| Gm27239 | blue3 |
| Mbp | blue4 |
| Rpl21-ps8 | blue4 |
| Zfp236 | blue3 |
| Gm10524 | lightsteelblue |
| Zfp516 | mistyrose |
| Tshz1 | blue3 |
| Zadh2 | blue3 |
| Zfp407 | blue4 |
| Cndp1 | blue4 |
| Cndp2 | blue3 |
| Cyb5a | blue3 |
| Gm16146 | blue4 |
| Timm21 | coral1 |
| Socs6 | coral1 |
| Rttn | blue4 |
| Eif3s6-ps2 | blue4 |
| Tmx3 | coral1 |
| Ighmbp2 | blue3 |
| Mrpl21 | blue4 |
| Cpt1a | mistyrose |
| Ppp6r3 | coral1 |
| Lrp5 | deeppink |
| 1810055G02Rik | blue4 |
| Suv420h1 | blue4 |
| Chka | darkgrey |
| Tcirg1 | blue3 |
| Ndufs8 | blue4 |
| Aldh3b1 | blue4 |
| Unc93b1 | blue4 |
| 1700055N04Rik | darkolivegreen |
| Acy3 | blue4 |
| Tbx10 | blue4 |
| Nudt8 | deeppink |
| Doc2g | blue3 |
| Ndufv1 | blue4 |
| Gstp1 | blue4 |
| Gstp2 | blue3 |
| BC021614 | blue4 |
| Cdk2ap2 | blue3 |
| Pitpnm1 | blue4 |
| Aip | blue3 |
| Tmem134 | deeppink |
| Coro1b | blue4 |
| Rps6kb2 | green4 |
| Ppp1ca | deeppink |
| Rad9a | blue4 |
| Pold4 | blue4 |
| Ssh3 | blue3 |
| Ankrd13d | blue3 |
| Adrbk1 | blue4 |
| Kdm2a | blue4 |
| Rhod | blue4 |
| A930001C03Rik | blue4 |
| Syt12 | blue4 |
| 2010003K11Rik | blue4 |
| Pcx | blue4 |
| Lrfn4 | blue4 |
| Rce1 | mistyrose |
| Sptbn2 | green4 |
| Rbm4b | blue3 |
| Rbm14 | blue4 |
| Ccs | blue4 |
| Ctsf | blue4 |
| Zdhhc24 | blue3 |
| Bbs1 | blue3 |
| Dpp3 | blue4 |
| Mrpl11 | lightsteelblue |
| Slc29a2 | blue3 |
| B3gnt1 | green4 |
| Brms1 | firebrick2 |
| Rin1 | blue4 |
| Cd248 | blue4 |
| Tmem151a | coral1 |
| Yif1a | deeppink |
| Cnih2 | darkgrey |
| Rab1b | coral1 |
| Klc2 | blue3 |
| Pacs1 | blue4 |
| Sf3b2 | blue3 |
| Banf1 | blue4 |
| Eif1ad | blue4 |
| Sart1 | blue4 |
| 4930481A15Rik | darkolivegreen |
| Drap1 | firebrick2 |
| AI837181 | blue4 |
| Fosl1 | darkgrey |
| Ccdc85b | blue4 |
| Fibp | blue4 |
| Efemp2 | blue4 |
| Mus81 | blue3 |
| Cfl1 | blue4 |
| Snx32 | blue3 |
| Ovol1 | blue4 |
| Ap5b1 | blue4 |
| Rnaseh2c | coral1 |
| Kat5 | darkgrey |
| Rela | darkgrey |
| Sipa1 | blue4 |
| Pcnxl3 | green4 |
| Map3k11 | deeppink |
| Ehbp1l1 | blue4 |
| Sssca1 | blue4 |
| Ltbp3 | deeppink |
| Scyl1 | blue3 |
| Malat1 | mistyrose |
| Neat1 | blue3 |
| Frmd8 | blue4 |
| Slc25a45 | coral1 |
| Dpf2 | deeppink |
| Cdc42ep2 | blue4 |
| Pola2 | blue4 |
| Capn1 | blue3 |
| Gm8034 | blue3 |
| Syvn1 | coral1 |
| Mrpl49 | blue3 |
| Fau | firebrick2 |
| Znhit2 | coral1 |
| Tm7sf2 | blue4 |
| Vps51 | deeppink |
| Zfpl1 | deeppink |
| Sac3d1 | blue4 |
| Snx15 | blue4 |
| Arl2 | blue3 |
| Ppp2r5b | mistyrose |
| Atg2a | green4 |
| Mir192 | firebrick2 |
| Ehd1 | mistyrose |
| Cdc42bpg | green4 |
| Men1 | green4 |
| Map4k2 | green4 |
| Sf1 | darkgrey |
| Pygm | deeppink |
| Rasgrp2 | blue3 |
| Gm14964 | blue4 |
| Slc22a12 | blue4 |
| Gm14967 | darkgrey |
| Gm26460 | blue3 |
| Rps6ka4 | blue4 |
| Ccdc88b | blue4 |
| Prdx5 | blue4 |
| Trmt112 | blue4 |
| Esrra | green4 |
| Tex40 | darkolivegreen |
| Gpr137 | green4 |
| Bad | deeppink |
| Plcb3 | blue3 |
| Ppp1r14b | blue4 |
| Fkbp2 | blue4 |
| Vegfb | green4 |
| Dnajc4 | blue3 |
| Nudt22 | blue3 |
| Trpt1 | blue3 |
| Fermt3 | blue4 |
| Stip1 | blue4 |
| Macrod1 | blue4 |
| Flrt1 | green4 |
| Otub1 | mistyrose |
| Cox8a | blue4 |
| Naa40 | blue3 |
| Rcor2 | blue3 |
| Mark2 | deeppink |
| AI846148 | mistyrose |
| Rab11b-ps2 | blue4 |
| 2700081O15Rik | blue3 |
| Rtn3 | blue4 |
| Atl3 | blue4 |
| Pla2g16 | blue4 |
| Gm16437 | antiquewhite2 |
| Slc22a19 | blue4 |
| Slc22a26 | darkolivegreen |
| Slc22a28 | blue4 |
| Slc22a29 | blue4 |
| Slc22a30 | blue4 |
| Slc22a8 | green4 |
| Slc22a6 | blue4 |
| Slc3a2 | coral1 |
| Snhg1 | darkolivegreen |
| Wdr74 | blue4 |
| Stx5a | blue4 |
| Nxf1 | blue3 |
| Taf6l | green4 |
| Polr2g | blue4 |
| Zbtb3 | blue4 |
| Ttc9c | blue4 |
| Hnrnpul2 | blue3 |
| Bscl2 | deeppink |
| Ubxn1 | blue4 |
| Uqcc3 | mistyrose |
| Gm25822 | blue4 |
| Ints5 | blue4 |
| Ganab | green4 |
| B3gat3 | deeppink |
| Rom1 | deeppink |
| Eml3 | blue3 |
| Mta2 | blue4 |
| Tut1 | mistyrose |
| Eef1g | blue4 |
| Ahnak | blue4 |
| Asrgl1 | blue4 |
| Incenp | blue4 |
| Fth1 | blue4 |
| Rab3il1 | green4 |
| Fads3 | green4 |
| Fads2 | blue4 |
| Fads1 | green4 |
| Tmem258 | blue4 |
| Myrf | deeppink |
| Dagla | blue3 |
| Syt7 | green4 |
| Sdhaf2 | blue4 |
| Cpsf7 | blue4 |
| Tmem216 | blue4 |
| Tmem138 | blue4 |
| Cyb561a3 | blue3 |
| Dak | blue4 |
| Ddb1 | blue4 |
| Vwce | green4 |
| Vps37c | darkgrey |
| Slc15a3 | honeydew |
| Tmem132a | green4 |
| Tmem109 | blue3 |
| Prpf19 | blue4 |
| Ccdc86 | blue4 |
| AW112010 | saddlebrown |
| Ms4a4a | honeydew |
| Ms4a6c | saddlebrown |
| Ms4a6b | saddlebrown |
| Mrpl16 | blue3 |
| Stx3 | darkgrey |
| Patl1 | blue4 |
| Osbp | blue4 |
| Mpeg1 | saddlebrown |
| Dtx4 | blue4 |
| Fam111a | blue4 |
| A330040F15Rik | blue3 |
| Gm4952 | blue4 |
| Glyat | mistyrose |
| Olfr1442 | green4 |
| Keg1 | darkolivegreen |
| Gm15962 | darkolivegreen |
| Zfp91 | indianred2 |
| Lpxn | darkgrey |
| Tle4 | darkgrey |
| Psat1 | blue4 |
| Cep78 | blue4 |
| Gnaq | blue4 |
| Gm10819 | blue4 |
| Gna14 | blue4 |
| Vps13a | blue4 |
| Gcnt1 | darkolivegreen |
| Rfk | blue4 |
| Pcsk5 | orangered |
| Ostf1 | blue4 |
| Gm8250 | blue3 |
| Nmrk1 | blue3 |
| 2410127L17Rik | blue4 |
| D030056L22Rik | blue4 |
| Trpm6 | darkgrey |
| Anxa1 | blue4 |
| Aldh1a1 | blue4 |
| Aldh1a7 | blue4 |
| Zfand5 | darkgrey |
| Gda | blue4 |
| 1110059E24Rik | antiquewhite2 |
| Abhd17b | blue3 |
| Tmem2 | darkgrey |
| Gm5514 | antiquewhite2 |
| C330002G04Rik | darkolivegreen |
| Klf9 | blue3 |
| Mir1192 | blue4 |
| Smc5 | blue4 |
| Gm9493 | blue4 |
| Gm6563 | blue4 |
| Ptar1 | blue4 |
| Gm9938 | blue4 |
| Fam189a2 | green4 |
| Tjp2 | blue4 |
| Fxn | indianred2 |
| Pip5k1b | blue3 |
| Fam122a | blue4 |
| Tmem252 | blue3 |
| Pgm5 | blue4 |
| Gm10053 | antiquewhite2 |
| Cbwd1 | coral1 |
| Dock8 | green4 |
| Kank1 | blue4 |
| 2610016A17Rik | blue4 |
| Dmrt2 | blue4 |
| Smarca2 | blue4 |
| 4931403E22Rik | green4 |
| Vldlr | firebrick2 |
| D19Bwg1357e | blue4 |
| Rfx3 | blue3 |
| Glis3 | blue4 |
| Slc1a1 | blue4 |
| 4430402I18Rik | blue4 |
| Ppapdc2 | green4 |
| Cdc37l1 | mistyrose |
| Gm10136 | darkgrey |
| Ak3 | blue4 |
| 1700018L02Rik | darkgrey |
| Rcl1 | coral1 |
| Jak2 | coral1 |
| Insl6 | green4 |
| Plgrkt | blue3 |
| Ric1 | deeppink |
| Ermp1 | blue4 |
| 9930021J03Rik | darkgrey |
| Ranbp6 | coral1 |
| Il33 | blue4 |
| Uhrf2 | blue3 |
| Gldc | green4 |
| Cstf2t | firebrick2 |
| A1cf | firebrick2 |
| Asah2 | mistyrose |
| Sgms1 | blue4 |
| 2700046G09Rik | darkolivegreen |
| Rpl9-ps6 | blue4 |
| Minpp1 | blue4 |
| Papss2 | darkolivegreen |
| Atad1 | blue4 |
| Pten | indianred2 |
| Rnls | blue4 |
| Lipo1 | blue3 |
| Lipo2 | blue3 |
| Acta2 | blue4 |
| Fas | green4 |
| Ch25h | darkgrey |
| Lipa | blue3 |
| Ifit2 | saddlebrown |
| Ifit3 | saddlebrown |
| Gm14446 | mistyrose |
| I830012O16Rik | saddlebrown |
| 2010002M12Rik | mistyrose |
| Ifit1 | saddlebrown |
| Slc16a12 | blue4 |
| Pank1 | blue4 |
| Kif20b | blue3 |
| Rpp30 | blue4 |
| Ankrd1 | blue4 |
| Pcgf5 | brown1 |
| Ppp1r3c | lightcoral |
| Tnks2 | blue4 |
| Fgfbp3 | blue4 |
| Btaf1 | blue4 |
| Cpeb3 | blue4 |
| Gm23918 | blue3 |
| 5-Mar | green4 |
| Ide | blue4 |
| Kif11 | blue4 |
| Hhex | blue4 |
| Exoc6 | coral1 |
| Myof | blue4 |
| Ffar4 | darkgrey |
| Rbp4 | lightcoral |
| Fra10ac1 | blue4 |
| Slc35g1 | blue3 |
| Plce1 | blue4 |
| Noc3l | blue4 |
| Tbc1d12 | blue4 |
| Hells | blue4 |
| Pdlim1 | blue4 |
| Sorbs1 | blue4 |
| Aldh18a1 | blue4 |
| Gm27042 | blue3 |
| Tctn3 | green4 |
| Entpd1 | darkolivegreen |
| Ccnj | blue4 |
| Zfp518a | coral1 |
| Blnk | darkgrey |
| Tm9sf3 | coral1 |
| Pik3ap1 | honeydew |
| Lcor | blue4 |
| Gm340 | darkgrey |
| Mir8091 | blue4 |
| AI606181 | blue4 |
| Frat1 | blue4 |
| Frat2 | darkgrey |
| Rrp12 | blue4 |
| Pgam1 | blue3 |
| Exosc1 | blue4 |
| Zdhhc16 | blue4 |
| Mms19 | blue3 |
| Ubtd1 | firebrick2 |
| Hoga1 | blue3 |
| 4933411K16Rik | blue4 |
| Morn4 | mistyrose |
| Pi4k2a | darkgrey |
| Avpi1 | firebrick2 |
| Marveld1 | blue4 |
| Zfyve27 | mistyrose |
| R3hcc1l | brown1 |
| Loxl4 | blue4 |
| Pyroxd2 | blue4 |
| Hps1 | mistyrose |
| Cnnm1 | green4 |
| Got1 | blue3 |
| Slc25a28 | blue4 |
| Entpd7 | blue3 |
| Cox15 | mistyrose |
| Cutc | deeppink |
| Abcc2 | blue4 |
| Dnmbp | blue4 |
| Gm24400 | blue3 |
| Cpn1 | blue4 |
| Cyp2c44 | blue4 |
| Erlin1 | blue3 |
| Chuk | mistyrose |
| Cwf19l1 | blue3 |
| Gm24336 | blue3 |
| Bloc1s2 | blue4 |
| Scd2 | blue3 |
| Scd1 | saddlebrown |
| Sec31b | darkolivegreen |
| Ndufb8 | blue4 |
| Hif1an | blue4 |
| Pax2 | blue3 |
| 1700039E22Rik | blue3 |
| Fam178a | blue4 |
| Sema4g | green4 |
| Mrpl43 | blue4 |
| Peo1 | blue3 |
| Lzts2 | blue4 |
| Sfxn3 | blue3 |
| Kazald1 | blue3 |
| Btrc | blue3 |
| Gm6807 | blue4 |
| Poll | green4 |
| Dpcd | lightsteelblue |
| Fbxw4 | green4 |
| Npm3 | coral1 |
| Mgea5 | blue4 |
| Kcnip2 | blue4 |
| 9130011E15Rik | blue3 |
| Hps6 | blue4 |
| Ldb1 | darkgrey |
| Pprc1 | blue4 |
| Nolc1 | blue4 |
| Gbf1 | green4 |
| Nfkb2 | blue4 |
| Psd | blue4 |
| Fbxl15 | green4 |
| Cuedc2 | deeppink |
| Gm26792 | blue3 |
| Tmem180 | blue3 |
| 2310034G01Rik | blue3 |
| Actr1a | blue4 |
| Sufu | green4 |
| Trim8 | blue4 |
| Arl3 | blue4 |
| Sfxn2 | blue4 |
| Wbp1l | blue3 |
| 2010012O05Rik | blue4 |
| As3mt | firebrick2 |
| Cnnm2 | green4 |
| Nt5c2 | brown1 |
| Pcgf6 | blue4 |
| Taf5 | coral1 |
| Usmg5 | blue4 |
| Pdcd11 | blue3 |
| Calhm2 | blue4 |
| Sh3pxd2a | blue4 |
| Slk | blue4 |
| Sfr1 | blue4 |
| Cfap43 | coral1 |
| Gsto1 | blue4 |
| Gsto2 | green4 |
| Xpnpep1 | coral1 |
| Add3 | blue4 |
| Mxi1 | blue4 |
| Smndc1 | mistyrose |
| Dusp5 | darkgrey |
| Smc3 | blue4 |
| Rbm20 | coral1 |
| Pdcd4 | deeppink |
| Bbip1 | blue4 |
| Shoc2 | coral1 |
| Adra2a | blue3 |
| Gpam | mistyrose |
| Acsl5 | blue4 |
| Zdhhc6 | firebrick2 |
| Vti1a | darkolivegreen |
| Tcf7l2 | blue3 |
| Habp2 | blue4 |
| Casp7 | blue4 |
| Dclre1a | blue3 |
| Nhlrc2 | blue4 |
| Adrb1 | blue4 |
| Ccdc186 | coral1 |
| Vwa2 | blue4 |
| Afap1l2 | blue3 |
| Ablim1 | blue3 |
| Fam160b1 | brown1 |
| Trub1 | blue4 |
| Atrnl1 | blue4 |
| Gfra1 | mistyrose |
| Pnliprp1 | blue3 |
| Hspa12a | lightsteelblue |
| 4930506M07Rik | blue4 |
| Pdzd8 | blue4 |
| Rps12-ps3 | blue4 |
| Emx2os | blue3 |
| Emx2 | blue3 |
| Rab11fip2 | firebrick2 |
| Fam204a | green4 |
| Cacul1 | darkgrey |
| Eif3a | blue4 |
| Snora19 | blue4 |
| Fam45a | coral1 |
| Sfxn4 | darkolivegreen |
| Prdx3 | mistyrose |
| Grk5 | blue4 |
| 5830428H23Rik | blue3 |
| Gm6020 | coral1 |
| Csf2ra | blue4 |
| Nudt10 | blue4 |
| Shroom4 | blue4 |
| Clcn5 | blue4 |
| 2010204K13Rik | blue4 |
| Syp | coral1 |
| Prickle3 | blue3 |
| Plp2 | blue4 |
| Magix | darkolivegreen |
| Gpkow | blue4 |
| Wdr45 | blue4 |
| Praf2 | blue4 |
| Ccdc120 | darkgrey |
| Tfe3 | mistyrose |
| Gripap1 | blue3 |
| Mir1198 | deeppink |
| Otud5 | blue3 |
| Pim2 | mistyrose |
| Slc35a2 | brown1 |
| Pqbp1 | mistyrose |
| Timm17b | deeppink |
| Hdac6 | mistyrose |
| Glod5 | mistyrose |
| Suv39h1 | blue4 |
| Was | honeydew |
| Wdr13 | firebrick2 |
| Rbm3 | blue4 |
| Tbc1d25 | mistyrose |
| Ebp | green4 |
| Porcn | green4 |
| Ftsj1 | blue4 |
| Gm14502 | blue3 |
| B630019K06Rik | blue3 |
| Lancl3 | blue3 |
| Gm6829 | blue4 |
| Cybb | saddlebrown |
| Dynlt3 | coral1 |
| Srpx | blue4 |
| Rpgr | blue4 |
| Tspan7 | mistyrose |
| Mid1ip1 | mistyrose |
| Bcor | blue4 |
| Atp6ap2 | deeppink |
| 1810030O07Rik | blue4 |
| Med14 | firebrick2 |
| AA414768 | darkolivegreen |
| 5730405O15Rik | blue3 |
| Usp9x | blue4 |
| Rpl3-ps1 | blue4 |
| Llph-ps2 | blue4 |
| 2010308F09Rik | coral1 |
| Ddx3x | brown1 |
| Cask | blue4 |
| Maoa | blue4 |
| Maob | lightcoral |
| Fundc1 | coral1 |
| Kdm6a | deeppink |
| Mir221 | blue3 |
| Chst7 | blue4 |
| Slc9a7 | coral1 |
| Rp2h | blue4 |
| Jade3 | blue4 |
| Rgn | coral1 |
| Gm14539 | antiquewhite2 |
| Gm23628 | darkolivegreen |
| Ndufb11 | blue4 |
| Rbm10 | blue3 |
| Uba1 | blue4 |
| Cdk16 | blue3 |
| Usp11 | mistyrose |
| Araf | blue3 |
| Timp1 | blue4 |
| Cfp | blue4 |
| Elk1 | deeppink |
| Uxt | blue3 |
| A230072C01Rik | blue3 |
| Zfp182 | blue3 |
| Klhl13 | blue4 |
| Wdr44 | blue4 |
| Gm15432 | blue4 |
| Gm2223 | blue4 |
| Il13ra1 | blue4 |
| Lonrf3 | darkgrey |
| Gm25047 | darkgrey |
| Pgrmc1 | mistyrose |
| Akap17b | blue3 |
| Gm6274 | blue4 |
| Slc25a43 | blue4 |
| Slc25a5 | mistyrose |
| C330007P06Rik | coral1 |
| Gm14541 | darkgrey |
| Ube2a | blue4 |
| Nkrf | blue4 |
| 6-Sep | blue3 |
| Rpl39 | blue4 |
| Snora69 | blue4 |
| Upf3b | blue3 |
| Nkap | coral1 |
| Ndufa1 | blue3 |
| Rnf113a1 | blue4 |
| Rhox6 | blue3 |
| Zbtb33 | indianred2 |
| Lamp2 | blue4 |
| Cul4b | blue4 |
| Mcts1 | blue4 |
| C1galt1c1 | blue4 |
| Gm14567 | antiquewhite2 |
| Gria3 | blue3 |
| Thoc2 | blue4 |
| Xiap | blue4 |
| Stag2 | coral1 |
| Dcaf12l1 | blue4 |
| Ocrl | blue4 |
| Apln | blue4 |
| Xpnpep2 | blue4 |
| Zdhhc9 | green4 |
| Utp14a | blue4 |
| Bcorl1 | blue3 |
| Elf4 | blue4 |
| Aifm1 | deeppink |
| Zfp280c | blue4 |
| Slc25a14 | blue4 |
| Rbmx2 | blue4 |
| Enox2 | firebrick2 |
| Gm14719 | blue4 |
| Firre | blue3 |
| Stk26 | blue4 |
| Rap2c | blue4 |
| Hs6st2 | mistyrose |
| Gpc4 | blue4 |
| Gpc3 | blue4 |
| Gm14586 | blue4 |
| Ccdc160 | coral1 |
| Phf6 | blue4 |
| Hprt | blue4 |
| Mir351 | coral1 |
| C430049B03Rik | blue3 |
| Fam122b | blue3 |
| Mospd1 | green4 |
| Gm14584 | deeppink |
| Gm14597 | blue4 |
| Cxx1c | blue4 |
| Cxx1a | blue3 |
| Cxx1b | firebrick2 |
| AW822252 | blue4 |
| 4930502E18Rik | blue3 |
| Zfp449 | coral1 |
| 6330419J24Rik | blue4 |
| Ddx26b | blue3 |
| Mmgt1 | coral1 |
| Slc9a6 | mistyrose |
| Fhl1 | blue4 |
| Htatsf1 | blue4 |
| Arhgef6 | blue4 |
| Rbmx | blue3 |
| Atp11c | coral1 |
| Gm7327 | blue4 |
| Gm14681 | blue4 |
| Fmr1 | blue4 |
| Fmr1nb | blue4 |
| Ids | blue3 |
| 1110012L19Rik | mistyrose |
| BC023829 | blue4 |
| Mamld1 | darkgrey |
| Mtm1 | coral1 |
| Mtmr1 | coral1 |
| Cd99l2 | brown1 |
| Hmgb3 | blue4 |
| Vma21 | firebrick2 |
| Gabra3 | green4 |
| Cetn2 | mistyrose |
| Nsdhl | blue3 |
| Zfp185 | blue4 |
| Xlr3b | blue4 |
| Zfp275 | blue4 |
| Haus7 | blue4 |
| Bgn | blue4 |
| Dusp9 | green4 |
| Slc6a8 | mistyrose |
| Bcap31 | blue4 |
| Abcd1 | green4 |
| Idh3g | blue4 |
| Ssr4 | firebrick2 |
| L1cam | green4 |
| Arhgap4 | blue3 |
| Naa10 | blue3 |
| Renbp | green4 |
| Hcfc1 | blue4 |
| Irak1 | blue4 |
| Mecp2 | blue4 |
| Flna | blue4 |
| Emd | mistyrose |
| Rpl10 | blue4 |
| Dnase1l1 | blue4 |
| Taz | blue3 |
| Atp6ap1 | green4 |
| Gdi1 | blue3 |
| Fam50a | blue4 |
| Plxna3 | blue3 |
| Lage3 | blue4 |
| Ubl4 | blue4 |
| Slc10a3 | blue4 |
| Fam3a | green4 |
| Ikbkg | blue4 |
| G6pdx | blue4 |
| Gm15361 | blue4 |
| Dkc1 | blue4 |
| Mpp1 | blue4 |
| F8 | firebrick2 |
| Fundc2 | mistyrose |
| Mtcp1 | blue3 |
| Brcc3 | coral1 |
| Gm8666 | blue4 |
| Vbp1 | blue4 |
| 4933407K13Rik | blue3 |
| Pls3 | blue4 |
| Tbl1x | blue4 |
| Prkx | darkgrey |
| Prrg1 | blue4 |
| Rps24-ps3 | blue4 |
| Gm14760 | blue4 |
| Tmem47 | blue4 |
| Dmd | blue4 |
| Tab3 | coral1 |
| Gyk | mistyrose |
| Gm6977 | blue4 |
| Pola1 | blue4 |
| Pdk3 | blue4 |
| Zfx | coral1 |
| Eif2s3x | green4 |
| Apoo | mistyrose |
| Maged1 | coral1 |
| Gspt2 | blue4 |
| Arhgef9 | firebrick2 |
| Amer1 | blue3 |
| Zc4h2 | blue4 |
| Las1l | blue4 |
| Msn | blue4 |
| Heph | coral1 |
| Ar | firebrick2 |
| Ophn1 | blue4 |
| Yipf6 | coral1 |
| Stard8 | green4 |
| Efnb1 | mistyrose |
| Pja1 | blue3 |
| Tmem28 | blue4 |
| Igbp1 | mistyrose |
| Pdzd11 | blue4 |
| Dlg3 | firebrick2 |
| Snx12 | blue4 |
| Foxo4 | darkgrey |
| Il2rg | darkgrey |
| Med12 | blue4 |
| Gjb1 | brown1 |
| Zmym3 | darkolivegreen |
| Nono | blue4 |
| Taf1 | blue4 |
| Ogt | blue3 |
| Gm14845 | blue4 |
| Tpt1-ps6 | antiquewhite2 |
| H3f3a-ps1 | blue4 |
| Nhsl2 | blue4 |
| Rgag4 | blue3 |
| Pin4 | blue4 |
| Rps4x | blue4 |
| Hdac8 | darkgrey |
| Phka1 | firebrick2 |
| Gm9115 | green4 |
| Gm6206 | blue4 |
| Chic1 | blue4 |
| Gm9157 | mistyrose |
| Ftx | blue3 |
| Mir421 | blue3 |
| Mir374b | blue3 |
| Slc16a2 | blue4 |
| Rlim | brown1 |
| Abcb7 | blue4 |
| Vcp-rs | blue4 |
| Uprt | blue4 |
| Zdhhc15 | blue4 |
| 5530601H04Rik | mistyrose |
| Pbdc1 | blue4 |
| Magee1 | blue3 |
| Atrx | blue4 |
| Gm26020 | blue3 |
| Magt1 | firebrick2 |
| Cox7b | mistyrose |
| Atp7a | blue4 |
| Tlr13 | honeydew |
| Pgk1 | deeppink |
| Taf9b | coral1 |
| Itm2a | blue4 |
| 2610002M06Rik | blue4 |
| Brwd3 | blue4 |
| Hmgn5 | coral1 |
| Sh3bgrl | blue4 |
| Rps6ka6 | blue4 |
| Apool | mistyrose |
| Chm | coral1 |
| Gm1866 | blue4 |
| Gm14957 | blue4 |
| Diap2 | blue4 |
| Tspan6 | blue4 |
| Srpx2 | blue4 |
| Sytl4 | blue3 |
| Cstf2 | blue4 |
| Trmt2b | darkolivegreen |
| Tmem35 | green4 |
| Timm8a1 | green4 |
| Rpl36a | blue4 |
| Gla | darkgrey |
| Hnrnph2 | coral1 |
| Armcx4 | blue4 |
| Armcx1 | blue4 |
| Armcx6 | blue4 |
| Armcx3 | blue4 |
| Armcx2 | blue4 |
| Zmat1 | mistyrose |
| Armcx5 | darkgrey |
| Gprasp1 | lightsteelblue |
| Bhlhb9 | blue4 |
| Arxes2 | lightcoral |
| Bex2 | blue4 |
| Bex4 | blue4 |
| Tceal8 | blue4 |
| Bex1 | darkgrey |
| Wbp5 | blue4 |
| Ngfrap1 | blue3 |
| Tceal1 | blue3 |
| Morf4l2 | blue4 |
| BC065397 | blue3 |
| Plp1 | blue3 |
| Fam199x | blue4 |
| Mum1l1 | blue4 |
| Rnf128 | firebrick2 |
| Tbc1d8b | blue4 |
| Cldn2 | mistyrose |
| Morc4 | blue4 |
| Rbm41 | coral1 |
| Prps1 | blue4 |
| Tsc22d3 | blue3 |
| Mid2 | coral1 |
| Psmd10 | blue4 |
| Atg4a | green4 |
| Col4a6 | blue4 |
| Col4a5 | blue3 |
| Nxt2 | mistyrose |
| Acsl4 | blue4 |
| Tmem164 | blue3 |
| Ammecr1 | blue4 |
| Chrdl1 | blue4 |
| Alg13 | blue4 |
| Amot | blue4 |
| Gm5644 | blue4 |
| Apex2 | blue4 |
| Alas2 | coral1 |
| Pfkfb1 | lightcoral |
| Maged2 | blue4 |
| Gnl3l | coral1 |
| Fgd1 | blue3 |
| Tsr2 | blue4 |
| Fam120c | blue3 |
| Gm6451 | blue4 |
| Phf8 | blue3 |
| Gm15151 | lightsteelblue |
| Huwe1 | blue4 |
| Hsd17b10 | blue4 |
| Ribc1 | blue3 |
| Smc1a | blue4 |
| Iqsec2 | green4 |
| Kdm5c | mistyrose |
| RP23-330G24.3 | blue3 |
| Kantr | firebrick2 |
| Tspyl2 | darkgrey |
| Shroom2 | blue4 |
| Gm6472 | blue4 |
| Mageh1 | blue4 |
| Rragb | blue3 |
| Ubqln2 | blue4 |
| Kctd12b | blue4 |
| 2210013O21Rik | blue4 |
| 4930524N10Rik | mistyrose |
| Sat1 | darkgrey |
| Acot9 | green4 |
| Prdx4 | darkolivegreen |
| Gm15163 | coral1 |
| Sms | lightsteelblue |
| Mbtps2 | coral1 |
| Rps6ka3 | blue4 |
| Eif1ax | blue4 |
| A830080D01Rik | blue4 |
| Gm15190 | blue4 |
| Sh3kbp1 | blue4 |
| Map3k15 | firebrick2 |
| Pdha1 | deeppink |
| Phka2 | blue3 |
| Cdkl5 | blue4 |
| Rai2 | darkolivegreen |
| Nhs | blue4 |
| Reps2 | firebrick2 |
| Gm7331 | blue4 |
| Rbbp7 | blue4 |
| Txlng | blue4 |
| Syap1 | coral1 |
| Mir3473a | blue4 |
| Ctps2 | blue4 |
| S100g | mistyrose |
| Ap1s2 | blue4 |
| Zrsr2 | blue4 |
| Car5b | blue4 |
| Siah1b | darkgrey |
| Tmem27 | blue4 |
| Ace2 | deeppink |
| Pir | blue4 |
| Figf | lightcoral |
| Piga | blue4 |
| Asb11 | darkolivegreen |
| Asb9 | blue3 |
| Mospd2 | blue4 |
| Gemin8 | blue3 |
| Gpm6b | blue4 |
| Ofd1 | blue4 |
| Trappc2 | coral1 |
| Rab9 | mistyrose |
| Tceanc | coral1 |
| Egfl6 | darkolivegreen |
| Tmsb4x | blue4 |
| Prps2 | firebrick2 |
| Msl3 | coral1 |
| Arhgap6 | blue3 |
| Hccs | blue4 |
| Gm15246 | blue3 |
| Mid1 | blue4 |
| Gm15726 | coral1 |
| Gm21887 | darkolivegreen |
| Kdm5d | blue3 |
| Eif2s3y | blue3 |
| Gm29650 | blue3 |
| Uty | coral1 |
| Ddx3y | green4 |
| Gm4017 | blue4 |
| Gm21857 | blue3 |
| Erdr1 | blue3 |
