# Supplementary material for: Sodium aescinate protects renal ischemia-reperfusion and pyroptosis through AKT/NLRP3 signaling pathway
Source: Ren Fail. 2025 Apr 22;47(1):2488140. doi: 10.1080/0886022X.2025.2488140 (PMC12016278; doi:10.1080/0886022X.2025.2488140)
Supplement: Supplementary data 2 KEGG enrichment analysis.docx [file IRNF_A_2488140_SM1629.docx]

| ID | Description | GeneRatio | BgRatio | pvalue | p.adjust | qvalue | geneID | Count | one_type | two_type |  |
| --- | --- | --- | --- | --- | --- | --- | --- | --- | --- | --- | --- |
| hsa03010 | Ribosome | 95/2309 | 153/7914 | 1.53E-17 | 5.03E-15 | 3.72E-15 | MRPL15/RPL7/RPL31/RPL37A/RPL7A/RPL12/RPL35/RPS27/MRPS21/RPL34/RPS20/RPS6/RPS8/MRPS15/RPL11/RPL22/MRPL20/MRPL33/RPL9/RPL5/RPLP0/RPL6/MRPS17/RPL21/MRPL35/MRPL19/RPL32/RPS9/RPL28/RPS5/RPS19/RPS16/RPS11/RPL13A/RPL18/RPS17/RPS3/MRPL17/RPL27A/RPS13/RPS15A/RPLP2/MRPL23/UBA52/RPL18A/RPL13/RPS25/RPLP1/RPL4/RPS27L/RSL24D1/MRPL3/RPL29/RPSA/RPL14/RPS12/RPS15/RPS26/RPS27A/MRPL22/RPL26/RPL23A/RPL23/RPL19/RPL27/RPL38/MRPL12/RPS7/MRPL32/RPS23/RPL15/RPS24/RPL37/RPL30/RPL8/RPL3/RPL35A/MRPS6/RPS2/RPL3L/MRPL28/RPS10/RPL10A/RPS28/RPS18/MRPL2/MRPS10/RPL36/RPS14/RPL17/MRPL21/RPL39/RPL10/RPS4X/RPL36A | 95 | Genetic Information Processing | Translation | |
| hsa03030 | DNA replication | 32/2309 | 36/7914 | 1.03E-13 | 1.69E-11 | 1.25E-11 | MCM3/PRIM2/MCM6/PCNA/POLE3/RPA2/RFC1/POLE/RFC5/MCM7/RFC3/RPA3/SSBP1/POLE4/MCM2/LIG1/POLD1/POLD3/MCM5/RNASEH2A/DNA2/PRIM1/POLD2/RPA1/RNASEH1/POLE2/RNASEH2B/MCM4/RFC4/POLD4/POLA2/POLA1 | 32 | Genetic Information Processing | Replication and repair | |
| hsa03013 | RNA transport | 86/2309 | 165/7914 | 3.64E-10 | 3.98E-08 | 2.94E-08 | EIF5B/EIF4E2/TPR/RPP38/UPF2/NUP188/NUP35/NUP160/NXT1/EIF2S2/RAE1/RBM8A/DDX20/EIF4E/TGS1/NDC1/EIF2B3/PABPC4/EIF3I/TACC3/NUP54/EIF2B1/RAN/POM121/POP7/EIF3B/NUP205/NUP210/SEC13/MAGOHB/STRAP/POP4/NUP62/CYFIP1/CLNS1A/NUP98/EIF3C/UPF3A/EIF4EBP1/NUP93/NUP133/EIF3G/SNUPN/EEF1A1/EIF1B/NUP43/NUP37/NUP107/THOC5/XPO1/GEMIN5/EIF4A1/NUP88/KPNB1/CASC3/SUMO2/NUP85/ALYREF/GEMIN2/EIF2S1/RPP40/NUP153/SMN1/RPP14/PRMT5/NUP155/PABPC1/EIF3D/RANGAP1/NUP50/AAAS/EIF2B5/EIF4G1/RNPS1/UBE2I/DDX39B/RPP21/XPO5/GEMIN6/THOC1/SEH1L/RPP30/EIF3A/THOC2/FMR1/EIF1AX | 86 | Genetic Information Processing | Translation | |
| hsa04110 | Cell cycle | 67/2309 | 124/7914 | 4.77E-09 | 3.91E-07 | 2.88E-07 | MCM3/MCM6/ANAPC1/PCNA/E2F1/RBL1/YWHAB/CDC14A/CCNE2/ORC3/CDC26/CDKN2C/HDAC1/SFN/E2F2/MAD2L2/CDK6/DBF4/YWHAH/ANAPC4/CHEK2/YWHAG/MCM7/MAD1L1/MAD2L1/MCM2/CCND2/TGFB1/PLK1/BUB3/TFDP1/MCM5/ANAPC10/ORC6/E2F4/CDKN2D/CHEK1/SMAD3/STAG1/ANAPC13/CDC25A/CDK1/CDK4/CDK2/PTTG1/YWHAE/CDC27/ANAPC11/YWHAQ/TGFB3/E2F3/CCNH/CCNB1/RB1/SKP2/RBX1/EP300/MCM4/PRKDC/CDC45/GSK3B/PKMYT1/CDC23/SMAD4/SMAD2/SMC3/SMC1A | 67 | Cellular Processes | Cell growth and death | |
| hsa04510 | Focal adhesion | 93/2309 | 199/7914 | 8.67E-08 | 5.69E-06 | 4.20E-06 | COL4A4/COL6A3/LAMC2/LAMC1/TNN/AKT3/CAPN2/LAMB3/ITGA8/VAV2/RAPGEF1/ITGB6/ITGA6/ITGAV/MYL9/SRC/LAMA5/SHC1/EGF/TNC/PIK3R3/CDC42/HGF/PDGFRA/SPP1/RAC1/FLT1/COL1A2/CAV2/CAV1/MET/FLNC/CCND2/VASP/ACTN4/PAK1/PARVA/MAPK3/DOCK1/COL4A1/COL4A2/VEGFC/MYLK3/ITGB1/BIRC2/MAP2K1/TLN2/PIK3CB/RHOA/ITGA9/CTNNB1/LAMA2/LAMA4/COL6A2/COL6A1/PPP1R12A/ITGA7/FLT4/MAPK9/COL1A1/ITGA3/ITGA2B/ITGB3/PRKCA/GRB2/ITGB4/ROCK2/LAMB1/ACTN1/PGF/AKT1/PIK3R1/ITGA1/FLNB/VCL/PTK2/PDGFB/PARVB/ITGA5/CRKL/PAK2/MYLK/GSK3B/THBS2/PDPK1/TNXB/MYL12A/ROCK1/LAMA3/PDGFRB/XIAP/FLNA/COL4A6 | 93 | Cellular Processes | Cellular community - eukaryotes | |
| hsa00480 | Glutathione metabolism | 35/2309 | 56/7914 | 2.14E-07 | 1.17E-05 | 8.62E-06 | IDH1/GSS/GSTM5/GCLM/GPX7/TXNDC12/SRM/PGD/LAP3/GSTK1/GGCT/HPGDS/NAT8/MGST1/IDH2/RRM1/GSR/GCLC/GSTA4/GSTA1/GSTA2/GPX1/GGT1/GGT5/GSTT1/GSTT2/GPX4/CHAC2/ODC1/RRM2/GPX6/GPX8/OPLAH/GSTP1/GSTO1 | 35 | Metabolism | Metabolism of other amino acids | |
| hsa01100 | Metabolic pathways | 497/2309 | 1439/7914 | 5.98E-07 | 2.80E-05 | 2.07E-05 | RDH10/COX5B/MGAT4A/UXS1/GLS/IDH1/PIKFYVE/ATIC/CYP27A1/NDUFA10/DTYMK/PIGN/KDSR/ACMSD/PLA2G4A/NPL/PRDX6/UCK2/ALDH9A1/SDHC/NDUFS2/B4GALT3/PIGM/KMO/PYCR2/DEGS1/BPNT1/EPRS/NUDT5/DHTKD1/PIP4K2A/ENTPD8/UAP1L1/ENTPD2/AGPAT2/SARDH/ASS1/UCK1/DPM2/ST6GALNAC6/FPGS/PTGS1/NDUFS3/EXT2/HSD17B12/LPCAT4/IVD/SORD/GATM/DUT/BLVRA/ITPA/PANK2/SMOX/CDS2/PLCB4/ACSS1/AHCY/PIGU/ACSS2/GSS/NFS1/LPIN3/ADA/CYP24A1/NDUFB5/NDUFC1/ALG5/PLCH1/SLC33A1/B3GALNT1/GATB/PKLR/GBA/SELENBP1/PIP5K1A/CERS2/PHGDH/HSD3B2/HAO2/MAN1A2/GSTM5/ALG14/GCLM/PDE5A/ELOVL6/PLA2G12A/HADH/PAPSS1/BDH2/ADH5/AK5/ACO1/NUDT2/GNE/GRHPR/GALNT12/FKTN/MTAP/ALG6/PGM2/GPX7/TXNDC12/CMPK1/UQCRH/AKR1A1/UROD/HYI/ELOVL1/NT5C1A/NDUFS5/AZIN2/MECR/MAN1C1/HMGCL/GALE/DDOST/CDA/ALDH4A1/SDHB/AGMAT/DHRS3/SRM/PGD/H6PD/NADK/B3GALT6/NOS3/CHPF2/GALNT11/TYMS/KHK/CAD/RBKS/PISD/ACOX3/BST1/CD38/QDPR/LAP3/PGM1/UGDH/DCK/MTHFD2L/ENOPH1/COQ2/CDS1/DAO/ACADS/GATC/SDSL/ALDH2/AACS/PSPH/VKORC1L1/ASL/KL/ASNS/IMPDH1/ATP6V1F/AHCYL2/AKR1B10/AGK/GSTK1/AOC1/GGCT/HPGDS/RPIA/MTHFD2/NAGK/SPR/NAT8/GFPT1/MTMR14/ATP6V1E1/ENO2/TPI1/NDUFA9/MGST1/PIK3C2G/PDE3A/GYS2/LDHB/BCAT1/NDUFA3/ETHE1/PAFAH1B3/BCKDHA/CYP2S1/BLVRB/COX7A1/PRODH2/COX6B1/ATP4A/ASPDH/MCEE/IDH2/FAH/ME3/ALG8/NDUFC2/DGAT2/NEU3/PGM2L1/PDE2A/RRM1/AMPD3/PDE3B/CYP2R1/XYLT1/ACSM5/NDUFAB1/QPRT/DCTPP1/HSD3B7/COX6A2/UROS/ECHS1/CYP2E1/SIRT3/TALDO1/CERS4/AGPAT5/HGSNAT/GSR/DCTD/GALNT7/MSMO1/NDUFA13/ISYNA1/PGLS/B3GNT3/MRI1/GCDH/GPT2/ADCY7/TK2/NQO1/ST3GAL2/LDHD/GCSH/BCO1/MLYCD/APRT/GALNS/ACSF3/GUCY1A2/DNMT1/ST3GAL4/STT3A/HMBS/TREH/PAFAH1B2/SDHD/ALG9/MPI/ADPGK/PKM/GLCE/ALDH1A2/GCLC/ELOVL5/GSTA4/GSTA1/GSTA2/COX7A2/BCKDHB/ME1/NMNAT3/PIK3CB/PCCB/NUDT16/ACY1/HYAL3/GPX1/IMPDH2/UQCRC1/GLB1/MTHFD1L/PDE7B/ALDH8A1/DSE/AMD1/SMPD2/QRSL1/P4HA1/PCBD1/SGPL1/HKDC1/ADO/UPB1/GGT1/GGT5/GSTT1/GSTT2/MIF/FTCD/AGPAT3/GPX4/UQCR11/PAH/NDUFA12/GALNT4/CYP27B1/B4GALNT1/MARS/RDH16/PTGES3/SUOX/UQCR10/PGAM2/UPP1/DDC/ACYP2/CHAC2/MGAT4B/LTC4S/UQCRQ/GALNT10/GUK1/PEMT/COX10/PLD2/SHPK/ALDOC/PIGW/PNPO/PIP4K2B/COASY/AOC2/NT5C/GALK1/ACOX1/AFMID/PGS1/DCXR/LPIN1/ATP6V1C2/ODC1/RRM2/MGAT2/MTHFD1/RDH11/ACOT4/DLST/GSTZ1/SPTLC2/GALC/DEGS2/PFKP/B3GALNT2/GPX6/MBOAT1/UQCRFS1/GMDS/GCNT2/ELOVL2/IPPK/AUH/SPTLC1/B4GALT7/PCBD2/FBP2/FBP1/PTDSS1/NDUFS6/LPCAT1/COX7C/BHMT/BHMT2/DMGDH/ARSB/PDE8B/HMGCR/GCNT4/HEXB/MCCC2/ELOVL7/GPX8/PDHB/ACOX2/FHIT/NDST2/ADK/TKT/OGDHL/GLUD1/GCH1/PCK2/GMPR2/CRYL1/FDFT1/EPHX2/ESD/PCCA/OXCT1/AGXT2/AMACR/CMBL/ATP6V1C1/DPYS/EXT1/NDUFB9/SQLE/ST3GAL1/TSTA3/OPLAH/CYC1/GPT/TST/GCAT/ADSL/ACO2/PMM1/NDUFA6/A4GALT/MCAT/ALG12/MIOX/ALG10B/CSAD/ABAT/PI4KA/SMPD4/PRODH/COMT/ALG3/DGKG/ST6GAL1/UMPS/HGD/NDUFB4/B4GALT4/NIT2/CPOX/GBE1/SYNJ1/GART/CBR1/CBR3/HLCS/PIGP/SYNJ2/AGPAT4/ACAT2/AMDHD2/PGP/PIGQ/NDUFV3/CBS/GNMT/MOCS1/NDUFA11/MAN2A1/NDUFV2/GALNT14/XDH/COX7A2L/HAAO/PIGF/CDO1/ALDH7A1/PDE6A/ACAA2/CNDP1/NDUFS8/ALDH3B1/NDUFV1/GSTP1/TM7SF2/COX8A/ASRGL1/FADS2/PSAT1/RFK/ALDH1A1/GDA/SGMS1/MINPP1/PANK1/PLCE1/ALDH18A1/NDUFB8/GSTO1/ACSL5/MAOA/NDUFB11/C1GALT1C1/OCRL/IDH3G/UPRT/PRPS1/ACSL4/ALG13/HSD17B10/CTPS2/PIGA | 497 | Metabolism | Global and overview maps | |
| hsa03040 | Spliceosome | 66/2309 | 135/7914 | 8.97E-07 | 3.68E-05 | 2.71E-05 | SNRPE/HNRNPU/RBM17/HNRNPA3/AQR/SNRPB/SNRPB2/PRPF3/SF3B4/RBM8A/BCAS2/PRPF38B/PRPF4/PPIH/PPIE/SF3A3/SNRNP40/SRSF10/DHX15/SRSF9/LSM8/TRA2A/LSM5/USP39/SNRPG/LSM3/MAGOHB/PRPF31/SNRPD2/SNRPA1/XAB2/RBMXL1/LSM6/SF3B3/CWC15/U2SURP/CCDC12/SF3B5/LSM7/DHX8/EFTUD2/ALYREF/RBM25/DDX46/HNRNPK/HNRNPC/PUF60/PHF5A/DDX23/HNRNPA1/SNRPC/PPIL1/U2AF1/HNRNPM/LSM2/DDX39B/CDC5L/SRSF7/THOC1/SNRPD1/TCERG1/RBM22/TXNL4A/SART1/PRPF19/THOC2 | 66 | Genetic Information Processing | Transcription | |
| hsa03430 | Mismatch repair | 18/2309 | 23/7914 | 1.52E-06 | 5.52E-05 | 4.08E-05 | PCNA/RPA2/RFC1/RFC5/PMS2/RFC3/RPA3/SSBP1/LIG1/POLD1/POLD3/MLH1/POLD2/RPA1/RFC4/MSH2/MSH6/POLD4 | 18 | Genetic Information Processing | Replication and repair | |
| hsa04932 | Non-alcoholic fatty liver disease (NAFLD) | 70/2309 | 149/7914 | 2.69E-06 | 8.22E-05 | 6.07E-05 | COX5B/CASP8/NDUFA10/SDHC/NDUFS2/AKT3/TRAF2/RXRA/NDUFS3/IL1B/NDUFB5/NDUFC1/PKLR/PRKAB2/NFKB1/NDUFB6/UQCRH/PIK3R3/NDUFS5/CDC42/SDHB/MLXIP/RAC1/TNFRSF1A/NDUFA9/NDUFA3/TGFB1/COX7A1/COX6B1/CEBPA/BAX/NDUFC2/NDUFAB1/COX6A2/CYP2E1/IRS2/CASP3/NDUFA13/SDHD/COX7A2/PIK3CB/UQCRC1/UQCR11/NDUFA12/UQCR10/MAPK9/UQCRQ/MLX/EIF2S1/AKT1/UQCRFS1/NDUFS6/COX7C/PIK3R1/PRKAA1/NDUFB9/CYC1/NDUFA6/NDUFB4/GSK3B/NDUFV3/NDUFA11/NDUFV2/COX7A2L/NDUFS8/NDUFV1/COX8A/NDUFB8/CASP7/NDUFB11 | 70 | Human Diseases | Endocrine and metabolic disease | |
| hsa05222 | Small cell lung cancer | 48/2309 | 92/7914 | 2.76E-06 | 8.22E-05 | 6.07E-05 | COL4A4/LAMC2/LAMC1/AKT3/TRAF5/LAMB3/TRAF2/RXRA/TRAF1/ITGA6/ITGAV/E2F1/LAMA5/CKS1B/NFKB1/CCNE2/PIK3R3/E2F2/CDK6/BAX/COL4A1/COL4A2/CASP3/ITGB1/BIRC2/PIK3CB/LAMA2/LAMA4/APAF1/CDK4/CDK2/ITGA3/ITGA2B/LAMB1/TRAF3/AKT1/E2F3/PIK3R1/FHIT/RARB/RB1/SKP2/PTK2/BAK1/LAMA3/XIAP/IKBKG/COL4A6 | 48 | Human Diseases | Cancer: specific types | |
| hsa05131 | Shigellosis | 37/2309 | 67/7914 | 6.86E-06 | 0.000188 | 0.000138 | ARPC2/ARPC5/ARPC5L/CD44/SRC/ELMO2/PFN2/NFKB1/RIPK2/WASF2/CDC42/MAD2L2/ARPC3/RAC1/ARPC1A/ARPC1B/WASL/RHOG/MAPK3/DOCK1/CTTN/ITGB1/WASF1/UBE2D1/FBXW11/MAPK9/PFN1/ROCK2/VCL/MAPK11/ITGA5/CRKL/MAPK14/MAPK13/U2AF1/ROCK1/IKBKG | 37 | Human Diseases | Infectious disease: bacterial | |
| hsa05132 | Salmonella infection | 42/2309 | 80/7914 | 9.35E-06 | 0.000236 | 0.000174 | ARPC2/ARPC5/ARPC5L/DYNC1I2/IL1B/LBP/PFN2/NFKB1/TLR4/WASF2/CDC42/PLEKHM2/ARPC3/RAC1/ARPC1A/ARPC1B/WASL/FLNC/KLC3/TJP1/RHOG/MAPK3/PYCARD/DYNC1LI2/CASP1/DYNC1LI1/MYD88/IFNGR1/WASF1/MAPK9/PFN1/ROCK2/DYNC1H1/KLC1/FLNB/MAPK11/IFNGR2/MAPK14/MAPK13/ROCK1/CD14/FLNA | 42 | Human Diseases | Infectious disease: bacterial | |
| hsa05130 | Pathogenic Escherichia coli infection | 87/2309 | 201/7914 | 1.15E-05 | 0.000268 | 0.000198 | IL1R1/NCK2/MYO1B/CASP8/ARPC2/NCL/ARPC5/ABI1/TRAF2/ARPC5L/WIPF1/NCKAP1/F2/IL1B/SEC24B/NFKB1/LYN/MAP3K7/LPAR1/TLR4/WASF2/CDC42/PTPN11/ARPC3/CLDN15/RAC1/ARPC1A/ARPC1B/WASL/WIPF3/BRK1/TNFRSF1A/MYH14/BAX/CYFIP1/TJP1/PAK1/RPS3/MAPK3/PYCARD/CTTN/CASP3/LPAR2/ITGB1/CASP1/TIRAP/ARHGEF12/MYO5A/RHOA/MYD88/TAB2/WASF1/ARHGEF25/MAPK9/ARF1/MYH10/CLDN7/MYO1C/GNA13/ROCK2/ARF6/TMED10/TUBB2A/TUBB2B/F2R/TNFRSF10B/CLDN10/MYH9/CYTH4/MAPK11/IRAK4/TUBA1B/TUBA1A/TMBIM6/CLDN1/CLDN16/PAK2/CLDN25/BAK1/MAPK14/MAPK13/MYO1F/ROCK1/MYO5B/CASP7/IRAK1/IKBKG | 87 | Human Diseases | Infectious disease: bacterial | |
| hsa05016 | Huntington disease | 84/2309 | 193/7914 | 1.25E-05 | 0.000274 | 0.000202 | COX5B/CASP8/CREB1/NDUFA10/SDHC/NDUFS2/NDUFS3/CREB3L1/BDNF/PLCB4/TGM2/NDUFB5/NDUFC1/NDUFB6/UQCRH/NDUFS5/HDAC1/SDHB/HTT/HIP1/POLR2J/NDUFA9/NDUFA3/AP2S1/COX7A1/COX6B1/AP2A1/BAX/NDUFC2/NDUFAB1/COX6A2/POLR2L/VDAC3/CASP3/NDUFA13/UCP1/SDHD/SIN3A/COX7A2/GPX1/UQCRC1/TBPL1/POLR2E/UQCR11/APAF1/NDUFA12/DCTN2/UQCR10/VDAC1/UQCRQ/AP2B1/CLTC/HAP1/RCOR1/UQCRFS1/NDUFS6/COX7C/VDAC2/POLR2K/NDUFB9/CYC1/POLR2F/EP300/NDUFA6/SP1/AP2M1/POLR2H/NDUFB4/SOD1/SOD2/NDUFV3/NDUFA11/NDUFV2/COX7A2L/TAF4B/POLR2D/DCTN4/NDUFS8/NDUFV1/COX8A/POLR2G/GNAQ/NDUFB8/NDUFB11 | 84 | Human Diseases | Neurodegenerative disease | |
| hsa04810 | Regulation of actin cytoskeleton | 90/2309 | 213/7914 | 2.55E-05 | 0.000522 | 0.000386 | ABI2/PIKFYVE/ARPC2/ARPC5/ENAH/ITGA8/PIP4K2A/VAV2/ARPC5L/ITGB6/ITGA6/NCKAP1/ITGAV/F2/MYL9/SRC/PFN2/PIP5K1A/NRAS/EGF/LPAR1/PIK3R3/WASF2/CDC42/PDGFRA/SSH1/ARPC3/LIMK1/RAC1/ARPC1A/ARPC1B/WASL/BRK1/KRAS/ACTN4/MYH14/RRAS/CYFIP1/IQGAP1/PAK1/RRAS2/MAPK3/ITGAL/DOCK1/LPAR2/MYLK3/ITGB1/ARHGEF12/MAP2K1/PIK3CB/RHOA/ITGA9/WASF1/PPP1R12A/ITGA7/FGF18/MYH10/PFN1/SSH2/ITGA3/PIP4K2B/ITGA2B/ITGB3/GNA13/ITGB4/ROCK2/SCIN/CFL2/ACTN1/F2R/IQGAP2/PIK3R1/ITGA1/VCL/PTK2/MYH9/PDGFB/ITGA5/CRKL/PAK2/MYLK/TIAM1/MYL12A/ROCK1/APC/PDGFRB/CFL1/ARHGEF6/MSN/TMSB4X | 90 | Cellular Processes | Cell motility | |
| hsa03420 | Nucleotide excision repair | 27/2309 | 47/7914 | 4.70E-05 | 0.000906 | 0.000669 | ERCC5/PCNA/RAD23B/POLE3/RPA2/RFC1/POLE/RFC5/RFC3/RPA3/POLE4/LIG1/POLD1/POLD3/POLD2/RPA1/POLE2/MNAT1/CCNH/ERCC8/ERCC6/RBX1/RFC4/ERCC3/POLD4/DDB1/CUL4B | 27 | Genetic Information Processing | Replication and repair | |
| hsa01200 | Carbon metabolism | 54/2309 | 117/7914 | 6.68E-05 | 0.001188 | 0.000877 | IDH1/SDHC/ACSS1/ACSS2/PKLR/PHGDH/HAO2/ADH5/ACO1/SDHB/PGD/H6PD/ACOX3/ACADS/SDSL/PSPH/RPIA/ENO2/TPI1/MCEE/IDH2/ME3/ECHS1/TALDO1/PGLS/GPT2/GCSH/SDHD/ADPGK/PKM/ME1/PCCB/HKDC1/PGAM2/ALDOC/ACOX1/DLST/PFKP/FBP2/FBP1/PDHB/TKT/OGDHL/GLUD1/ESD/PCCA/GPT/ACO2/ACAT2/PGP/ME2/PSAT1/IDH3G/PRPS1 | 54 | Metabolism | Global and overview maps | |
| hsa05212 | Pancreatic cancer | 38/2309 | 75/7914 | 6.88E-05 | 0.001188 | 0.000877 | RALB/AKT3/RAD51/E2F1/EGF/NFKB1/TGFBR1/JAK1/PIK3R3/E2F2/CDC42/MTOR/CDK6/RAC1/TGFA/KRAS/TGFB1/BAX/MAPK3/SMAD3/MAP2K1/PIK3CB/TGFBR2/CDK4/MAPK9/RPS6KB1/TGFB3/AKT1/RALA/E2F3/PIK3R1/RB1/BAK1/RALBP1/SMAD4/SMAD2/ARHGEF6/IKBKG | 38 | Human Diseases | Cancer: specific types | |
| hsa05135 | Yersinia infection | 55/2309 | 120/7914 | 7.27E-05 | 0.001192 | 0.00088 | ACTR3/AKT3/TRAF2/VAV2/WIPF1/IL1B/SRC/PIP5K1A/NFKB1/MAP3K7/TLR4/PIK3R3/WASF2/RPS6KA1/CDC42/GIT2/LIMK1/RAC1/WASL/SKAP2/WIPF3/LAT/MAPK3/PYCARD/DOCK1/NFATC3/ITGB1/CASP1/ARHGEF12/MAP2K1/PIK3CB/RHOA/MYD88/TAB2/MAPK9/MAP2K6/ROCK2/ARF6/AKT1/ARHGEF28/PIK3R1/PTK2/MAPK11/IRAK4/ITGA5/CRKL/GSK3B/MAPK14/MAPK13/ROCK1/NFATC1/IRAK1/IKBKG/RPS6KA6/RPS6KA3 | 55 | Human Diseases | Infectious disease: bacterial | |
| hsa04218 | Cellular senescence | 69/2309 | 160/7914 | 0.000103 | 0.001615 | 0.001192 | AKT3/CAPN2/E2F1/RBL1/PPID/NRAS/NFKB1/CCNE2/NBN/TGFBR1/PIK3R3/RBBP4/E2F2/MTOR/CDK6/LIN54/CHEK2/SERPINE1/HIPK2/CCND2/FOXM1/KRAS/TGFB1/LIN37/RRAS/RRAS2/MAPK3/VDAC3/EIF4EBP1/E2F4/NFATC3/ETS1/CHEK1/SMAD3/MAP2K1/PIK3CB/CDC25A/TGFBR2/TRAF3IP2/MCU/CDK1/CDK4/CDK2/PPP3R1/FBXW11/VDAC1/RAD50/MAP2K6/LIN52/TGFB3/CALM1/AKT1/CALML3/E2F3/CCNB1/PIK3R1/PPP3CB/VDAC2/NFATC4/RB1/RAD1/MAPK11/ITPR3/MAPK14/MAPK13/ZFP36L2/SMAD2/NFATC1/RAD9A | 69 | Cellular Processes | Cell growth and death | |
| hsa03410 | Base excision repair | 20/2309 | 33/7914 | 0.000166 | 0.002474 | 0.001826 | PCNA/POLE3/MUTYH/POLE/UNG/POLE4/OGG1/LIG1/POLD1/POLD3/POLB/PARP3/POLD2/LIG3/POLE2/PARP2/APEX1/SMUG1/POLD4/APEX2 | 20 | Genetic Information Processing | Replication and repair | |
| hsa05100 | Bacterial invasion of epithelial cells | 36/2309 | 73/7914 | 0.000214 | 0.003054 | 0.002255 | ARPC2/ARPC5/ARPC5L/SRC/ELMO2/SHC1/PIK3R3/WASF2/CDC42/MAD2L2/ARPC3/RAC1/ARPC1A/ARPC1B/CAV2/CAV1/MET/WASL/RHOG/DOCK1/CTTN/GAB1/ITGB1/CBL/PIK3CB/RHOA/CTNNB1/WASF1/CLTC/PIK3R1/VCL/PTK2/ITGA5/CRKL/CD2AP/CTNNA1 | 36 | Human Diseases | Infectious disease: bacterial | |
| hsa05010 | Alzheimer disease | 71/2309 | 171/7914 | 0.000331 | 0.00453 | 0.003344 | COX5B/CASP8/NDUFA10/SDHC/NDUFS2/NCSTN/CAPN2/NDUFS3/IL1B/PLCB4/NDUFB5/NDUFC1/NDUFB6/UQCRH/NDUFS5/SDHB/TNFRSF1A/NDUFA9/NDUFA3/COX7A1/COX6B1/NDUFC2/NDUFAB1/MAPK3/COX6A2/CASP3/LPL/NDUFA13/CASP12/SDHD/APH1B/ADAM10/COX7A2/UQCRC1/UQCR11/APAF1/NDUFA12/LRP1/UQCR10/PPP3R1/RTN4/UQCRQ/ADAM17/CALM1/CALML3/UQCRFS1/NDUFS6/COX7C/PPP3CB/NDUFB9/CYC1/NDUFA6/NDUFB4/GSK3B/APP/BACE2/ITPR3/NDUFV3/NDUFA11/NDUFV2/COX7A2L/NDUFS8/NDUFV1/COX8A/RTN3/GNAQ/IDE/NDUFB8/CASP7/NDUFB11/HSD17B10 | 71 | Human Diseases | Neurodegenerative disease | |
| hsa05203 | Viral carcinogenesis | 81/2309 | 201/7914 | 0.000411 | 0.005398 | 0.003984 | CASP8/CREB1/SP100/HDAC4/TRAF5/TRAF2/TRAF1/CREB3L1/RBL1/SRC/YWHAB/NRAS/NFKB1/PRKACB/LYN/CCNE2/JAK1/PIK3R3/HDAC1/CDC42/CDK6/YWHAH/RBPJ/YWHAG/MAD1L1/RAC1/SND1/CHD4/LTBR/CCND2/KRAS/ACTN4/HPN/BAX/MAPK3/VDAC3/POLB/GTF2E2/CASP3/JAK3/CHEK1/PKM/GTF2A2/RASA2/PIK3CB/RHOA/TBPL1/CDK1/CDK4/CDK2/YWHAE/STAT5A/HDAC5/GRB2/YWHAQ/SCIN/ACTN1/TRAF3/HIST1H4I/HIST1H4H/HIST1H4D/HIST1H2BC/HIST1H4C/HNRNPK/PIK3R1/IL6ST/RB1/SKP2/EP300/HDAC7/USP7/RANBP1/DLG1/GTF2E1/BAK1/MRPS18B/C3/EIF2AK2/DDB1/NFKB2/IKBKG | 81 | Human Diseases | Cancer: overview | |
| hsa05200 | Pathways in cancer | 189/2309 | 530/7914 | 0.000489 | 0.006167 | 0.004552 | CASP8/COL4A4/RALB/TPR/LAMC2/LAMC1/AKT3/TRAF5/LAMB3/TRAF2/RXRA/TRAF1/ITGA6/NFE2L2/ITGAV/F2/RASGRP1/RAD51/PLCB4/E2F1/STK4/MMP9/LAMA5/HEY1/GNB4/NTRK1/CKS1B/TPM3/ARNT/NRAS/GSTM5/GNAI3/CAMK2D/EGF/NFKB1/GNG5/PRKACB/CCNE2/TGFBR1/LPAR1/JAK1/PIK3R3/PTCH2/HEYL/CSF3R/HDAC1/E2F2/CDC42/MTOR/GNB1/CDK6/HGF/PDGFRA/GNB2/RAC1/GNG11/MET/TGFA/TXNRD3/CCND2/MGST1/KRAS/TGFB1/CEBPA/BAX/ARNT2/MAPK3/CTBP2/COL4A1/COL4A2/CASP3/VEGFC/LPAR2/JAK3/IL15/ADCY7/NQO1/EGLN1/ITGB1/BIRC2/EPOR/ETS1/ARHGEF12/CBL/SMAD3/MAP2K1/DAPK2/GSTA4/GSTA1/GSTA2/PIK3CB/GNAI2/RHOA/MLH1/TGFBR2/CTNNB1/IFNGR1/LAMA2/LAMA4/GSTT1/GSTT2/DAPK3/TXNRD1/APAF1/CDK4/STAT2/CDK2/CAMK2B/FGF18/FLT4/MAPK9/TCF7/RPS6KB1/ITGA3/GNGT2/JUP/STAT5A/ITGA2B/FZD2/PRKCA/GNA13/GRB2/BIRC5/ROCK2/LAMB1/HIF1A/PGF/TGFB3/CALM1/TRAF3/AKT1/JAG2/CALML3/RALA/E2F3/EDN1/DAPK1/TERT/F2R/PIK3R1/IL6ST/IL3RA/RARB/GNG2/APPL1/WNT5A/RB1/PTGER4/SKP2/PTK2/IL2RB/PDGFB/RBX1/EP300/SP1/CRKL/GSK3B/TFG/IFNAR2/IFNAR1/IFNGR2/RUNX1/BAK1/NOTCH3/NOTCH4/RALBP1/EML4/MSH2/MSH6/CUL2/ROCK1/LAMA3/APC/CTNNA1/PDGFRB/CSF1R/SMAD4/SMAD2/GSTP1/GNAQ/FRAT1/NFKB2/GSTO1/CASP7/CSF2RA/IL13RA1/XIAP/IKBKG/COL4A6 | 189 | Human Diseases | Cancer: overview | |
| hsa04933 | AGE-RAGE signaling pathway in diabetic complications | 45/2309 | 100/7914 | 0.000527 | 0.006402 | 0.004725 | COL3A1/COL4A4/AKT3/IL1B/PLCB4/THBD/NRAS/NFKB1/TGFBR1/PIK3R3/CDC42/PRKCZ/NOS3/SERPINE1/RAC1/COL1A2/KRAS/TGFB1/BAX/MAPK3/COL4A1/COL4A2/CASP3/VEGFC/SMAD3/PIK3CB/TGFBR2/CDK4/MAPK9/COL1A1/STAT5A/PRKCA/TGFB3/AKT1/EDN1/PIK3R1/PRKCD/MAPK11/MAPK14/MAPK13/SMAD4/SMAD2/NFATC1/PLCE1/COL4A6 | 45 | Human Diseases | Endocrine and metabolic disease | |
| hsa05161 | Hepatitis B | 67/2309 | 162/7914 | 0.000548 | 0.006425 | 0.004742 | CASP8/CREB1/AKT3/IFIH1/CREB3L1/PCNA/E2F1/SRC/YWHAB/MMP9/TLR2/NRAS/NFKB1/CCNE2/MAP3K7/DDX58/TGFBR1/TLR4/JAK1/PIK3R3/E2F2/KRAS/TGFB1/BAX/MAPK3/VDAC3/TLR3/CASP3/JAK3/NFATC3/CASP12/TIRAP/SMAD3/MAP2K1/PIK3CB/TGFBR2/MYD88/TAB2/APAF1/STAT2/CDK2/MAPK9/STAT5A/PRKCA/MAP2K6/GRB2/BIRC5/YWHAQ/TGFB3/TRAF3/AKT1/E2F3/PIK3R1/MAP3K1/NFATC4/RB1/EP300/MAPK11/IRAK4/IFNAR1/MAPK14/MAPK13/SMAD4/NFATC1/DDB1/IRAK1/IKBKG | 67 | Human Diseases | Infectious disease: viral | |
| hsa03020 | RNA polymerase | 18/2309 | 31/7914 | 0.000731 | 0.008264 | 0.0061 | POLR1B/POLR3K/POLR3C/POLR1E/POLR2J/POLR1D/POLR1A/POLR2L/POLR2E/POLR3B/POLR3G/POLR3A/POLR2K/POLR2F/POLR3H/POLR2H/POLR2D/POLR2G | 18 | Genetic Information Processing | Transcription | |
| hsa04144 | Endocytosis | 94/2309 | 243/7914 | 0.000773 | 0.008446 | 0.006234 | ARFGEF1/SMAP1/CXCR2/ARPC2/ARPC5/ARPC5L/STAM2/WIPF1/EHD4/CHMP4B/SRC/ARFGEF2/RAB22A/CHMP4C/PRKCI/PIP5K1A/VPS45/CAPZA1/SH3GLB1/TGFBR1/SH3GL2/DNAJC6/EPS15/LDLRAP1/CDC42/CAPZB/PRKCZ/ACAP3/ARAP2/PDGFRA/GIT2/RAB35/ARPC3/VPS37D/SMURF1/ARPC1A/ARPC1B/CAV2/CAV1/WASL/WIPF3/CHMP3/STAMBP/CHMP2A/EHD2/AP2S1/CBLC/AP2A1/TSG101/ARRB1/FOLR1/VPS36/PSD3/VPS4A/IST1/CBL/SMAD3/RAB11A/SPG21/NEDD4/RHOA/PDCD6IP/TGFBR2/VTA1/EEA1/ARF1/EPN2/ARRB2/PLD2/RABEP1/AP2B1/CLTC/RAB5C/RAB10/ARF6/GRK6/ZFYVE16/ASAP1/IL2RB/CYTH4/AP2M1/TFRC/CHMP2B/SH3GL1/RAB31/KIF5B/BIN1/NEDD4L/SMAD2/PARD6G/PSD/GRK5/SNX12/SH3KBP1 | 94 | Cellular Processes | Transport and catabolism | |
| hsa04611 | Platelet activation | 53/2309 | 124/7914 | 0.000813 | 0.008605 | 0.006352 | COL3A1/PLA2G4A/AKT3/APBB1IP/PTGS1/F2/RASGRP1/SNAP23/PLCB4/SRC/PRKCI/FGG/FGA/FGB/GNAI3/PRKACB/LYN/PIK3R3/PRKCZ/NOS3/ORAI1/COL1A2/VAMP8/PTGIR/VASP/MAPK3/MYLK3/ADCY7/ITGB1/GUCY1A2/ARHGEF12/TLN2/PIK3CB/GNAI2/RHOA/PPP1R12A/COL1A1/ITGA2B/ITGB3/GNA13/ROCK2/AKT1/F2R/PIK3R1/MAPK11/MYLK/ITPR3/MAPK14/MAPK13/MYL12A/ROCK1/FERMT3/GNAQ | 53 | Organismal Systems | Immune system | |
| hsa01524 | Platinum drug resistance | 34/2309 | 73/7914 | 0.001175 | 0.012042 | 0.008889 | CASP8/AKT3/GSTM5/SLC31A1/PIK3R3/MGST1/BAX/MAPK3/CASP3/BIRC2/GSTA4/GSTA1/GSTA2/PIK3CB/MLH1/REV3L/GSTT1/GSTT2/APAF1/TOP2A/BIRC5/AKT1/PIK3R1/TOP2B/PDPK1/BAK1/POLH/MSH2/MSH6/GSTP1/ABCC2/GSTO1/XIAP/ATP7A | 34 | Human Diseases | Drug resistance: antineoplastic | |
| hsa04530 | Tight junction | 68/2309 | 169/7914 | 0.001214 | 0.012065 | 0.008905 | ACTR3/F11R/PCNA/MYL9/SRC/PRKCI/RAB13/CGN/PRKAB2/PRKACB/MPDZ/CDC42/PRKCZ/CLDN15/MICALL2/RAC1/CFTR/MAGI1/YBX3/VASP/ACTN4/MYH14/TJP1/WHAMM/ARHGAP17/CTTN/PPP2CB/ITGB1/AMOTL1/PPP2R1B/RAB8B/CGNL1/NEDD4/RHOA/CDK4/MYL6/MAPK9/HSPA4/RAPGEF6/LLGL1/MYH10/CLDN7/LLGL2/ROCK2/ACTN1/MARVELD2/MAP3K1/CLDN10/PRKAA1/MYH9/TUBA1B/TUBA1A/CLDN1/CLDN16/DLG1/CLDN25/JAM2/TIAM1/RUNX1/MYL12A/ROCK1/SYNPO/NEDD4L/PARD6G/TJP2/RAP2C/MSN/AMOT | 68 | Cellular Processes | Cellular community - eukaryotes | |
| hsa04360 | Axon guidance | 72/2309 | 181/7914 | 0.001283 | 0.012376 | 0.009135 | NCK2/BMPR2/ENAH/PLXNA2/MYL9/SRC/EFNA4/NRAS/GNAI3/CAMK2D/BMPR1B/PIK3R3/EPHB2/CDC42/PRKCZ/SEMA3D/SEMA3C/SSH1/PTPN11/LIMK1/RAC1/MET/EPHA1/PLXNA1/SRGAP3/KRAS/RRAS/RGMA/FES/PAK1/MAPK3/NFATC3/NRP1/ITGB1/ARHGEF12/SEMA7A/NEO1/PIK3CB/RYK/GNAI2/SEMA3F/RHOA/UNC5B/NTN4/SRGAP1/CAMK2B/PPP3R1/SLIT3/SSH2/PRKCA/ROCK2/CFL2/SEMA4D/RASA1/PIK3R1/PPP3CB/WNT5A/SEMA3G/NFATC4/DPYSL2/SEMA5A/PTK2/PAK2/GSK3B/ROBO1/EFNA5/MYL12A/ROCK1/ABLIM3/PARD6G/RHOD/CFL1 | 72 | Organismal Systems | Development and regeneration | |
| hsa04520 | Adherens junction | 33/2309 | 71/7914 | 0.001441 | 0.013506 | 0.009969 | PTPRJ/SRC/SNAI1/MAP3K7/TGFBR1/WASF2/CDC42/YES1/RAC1/MET/WASL/ACTN4/TJP1/IQGAP1/MAPK3/CSNK2A2/SMAD3/RHOA/TGFBR2/CTNNB1/WASF1/PTPRB/TCF7/NLK/ACTN1/VCL/EP300/SNAI2/FER/PTPRM/CTNNA1/SMAD4/SORBS1 | 33 | Cellular Processes | Cellular community - eukaryotes | |
| hsa04666 | Fc gamma R-mediated phagocytosis | 41/2309 | 93/7914 | 0.001496 | 0.01356 | 0.010009 | ARPC2/PTPRC/PLA2G4A/ARPC5/FCGR2B/AKT3/VAV2/ARPC5L/PIP5K1A/LYN/PIK3R3/MARCKSL1/WASF2/CDC42/ARPC3/NCF1/LIMK1/RAC1/ARPC1A/ARPC1B/VASP/PAK1/LAT/MAPK3/MAP2K1/PIK3CB/MARCKS/WASF1/PLD2/RPS6KB1/PRKCA/SCIN/CFL2/ARF6/AKT1/PIK3R1/PRKCD/ASAP1/CRKL/BIN1/CFL1 | 41 | Organismal Systems | Immune system | |
| hsa04114 | Oocyte meiosis | 53/2309 | 127/7914 | 0.001568 | 0.01356 | 0.010009 | ANAPC1/YWHAB/AURKA/CAMK2D/PRKACB/CCNE2/CDC26/RPS6KA1/MAD2L2/YWHAH/ANAPC4/YWHAG/MAD1L1/MAD2L1/CPEB1/PLK1/MAPK3/PPP2CB/ANAPC10/ADCY7/PPP2R1B/MAP2K1/ANAPC13/FBXO5/CDK1/CDK2/CAMK2B/PPP3R1/FBXW11/PTTG1/YWHAE/CDC27/ANAPC11/YWHAQ/PPP2R5E/CALM1/PPP2R5C/CALML3/CCNB1/PPP3CB/RBX1/MAPK11/PKMYT1/ITPR3/MAPK14/MAPK13/CDC23/CPEB3/SLK/SMC3/RPS6KA6/SMC1A/RPS6KA3 | 53 | Cellular Processes | Cell growth and death | |
| hsa03050 | Proteasome | 23/2309 | 45/7914 | 0.001571 | 0.01356 | 0.010009 | PSMD1/PSMD14/PSMC3/PSMF1/PSMA7/PSMB4/PSMA5/PSMB2/PSMC2/POMP/PSMC4/PSMD13/PSMD7/PSME4/PSMB6/PSMD11/PSMB3/PSME3/PSMC5/PSMA3/PSMA2/PSMB5/PSMD2 | 23 | Genetic Information Processing | Folding, sorting and degradation | |
| hsa05418 | Fluid shear stress and atherosclerosis | 57/2309 | 139/7914 | 0.001743 | 0.014145 | 0.010441 | IL1R1/BMPR2/AKT3/ASS1/NFE2L2/ITGAV/IL1B/THBD/SRC/MMP9/GSTM5/NFKB1/BMPR1B/MAP3K7/PIK3R3/PRKCZ/NOS3/NCF1/RAC1/CAV2/CAV1/TNFRSF1A/MGST1/NQO1/GSTA4/GSTA1/GSTA2/PIK3CB/RHOA/ACVR2B/CTNNB1/GSTT1/GSTT2/MAPK9/MAPK7/ITGA2B/ITGB3/PECAM1/MAP2K6/SUMO2/SDC1/CALM1/AKT1/CALML3/EDN1/MEF2C/PIK3R1/PRKAA1/SDC2/PTK2/PDGFB/MAPK11/MAPK14/MAPK13/GSTP1/GSTO1/IKBKG | 57 | Human Diseases | Cardiovascular disease | |
| hsa04722 | Neurotrophin signaling pathway | 50/2309 | 119/7914 | 0.001764 | 0.014145 | 0.010441 | AKT3/RAPGEF1/BDNF/NTRK1/SHC1/NGF/NRAS/CAMK2D/NFKB1/RIPK2/PIK3R3/RPS6KA1/CDC42/PTPN11/SH2B2/RAC1/ARHGDIB/KRAS/BAX/MAPK3/GAB1/MAP2K1/PIK3CB/RHOA/PRDM4/FRS2/CAMK2B/MAPK9/MAPK7/YWHAE/GRB2/ARHGDIA/CALM1/AKT1/CALML3/PIK3R1/MAP3K1/PRKCD/MAPK11/IRAK4/CRKL/GSK3B/PDPK1/ARHGDIG/MAPK14/MAPK13/NFKBIE/IRAK1/RPS6KA6/RPS6KA3 | 50 | Organismal Systems | Nervous system | |
| hsa05211 | Renal cell carcinoma | 32/2309 | 69/7914 | 0.001768 | 0.014145 | 0.010441 | AKT3/RAPGEF1/ARNT/NRAS/PIK3R3/CDC42/HGF/PTPN11/RAC1/MET/TGFA/KRAS/TGFB1/ARNT2/PAK1/MAPK3/GAB1/EGLN1/ETS1/MAP2K1/PIK3CB/GRB2/HIF1A/TGFB3/AKT1/PIK3R1/PDGFB/RBX1/EP300/CRKL/PAK2/CUL2 | 32 | Human Diseases | Cancer: specific types | |
| hsa00280 | Valine, leucine and isoleucine degradation | 24/2309 | 48/7914 | 0.001848 | 0.014436 | 0.010655 | ALDH9A1/IVD/HADH/HMGCL/ACADS/ALDH2/AACS/BCAT1/BCKDHA/MCEE/ECHS1/ACSF3/BCKDHB/PCCB/AUH/MCCC2/PCCA/OXCT1/AGXT2/ABAT/ACAT2/ALDH7A1/ACAA2/HSD17B10 | 24 | Metabolism | Amino acid metabolism | |
| hsa05210 | Colorectal cancer | 38/2309 | 86/7914 | 0.002085 | 0.015905 | 0.01174 | RALB/AKT3/NRAS/EGF/TGFBR1/PIK3R3/MTOR/RAC1/TGFA/KRAS/TGFB1/BAX/MAPK3/CASP3/SMAD3/MAP2K1/PIK3CB/RHOA/MLH1/TGFBR2/CTNNB1/MAPK9/TCF7/RPS6KB1/GRB2/BIRC5/TGFB3/AKT1/RALA/PIK3R1/APPL1/GSK3B/BAK1/MSH2/MSH6/APC/SMAD4/SMAD2 | 38 | Human Diseases | Cancer: specific types | |
| hsa05220 | Chronic myeloid leukemia | 34/2309 | 76/7914 | 0.00273 | 0.020352 | 0.015022 | AKT3/E2F1/SHC1/NRAS/NFKB1/TGFBR1/PIK3R3/HDAC1/E2F2/CDK6/PTPN11/KRAS/TGFB1/BAX/MAPK3/CTBP2/CBL/SMAD3/MAP2K1/PIK3CB/TGFBR2/CDK4/STAT5A/GRB2/TGFB3/AKT1/E2F3/PIK3R1/RB1/CRKL/RUNX1/BAK1/SMAD4/IKBKG | 34 | Human Diseases | Cancer: specific types | |
| hsa05225 | Hepatocellular carcinoma | 66/2309 | 168/7914 | 0.002869 | 0.020912 | 0.015436 | AKT3/NFE2L2/E2F1/ACTL6A/SHC1/NRAS/GSTM5/TGFBR1/PIK3R3/E2F2/MTOR/CDK6/HGF/MET/TGFA/TXNRD3/MGST1/KRAS/TGFB1/BAX/MAPK3/GAB1/BRD7/NQO1/SMARCA4/SMAD3/MAP2K1/GSTA4/GSTA1/GSTA2/PIK3CB/SMARCC1/TGFBR2/CTNNB1/GSTT1/GSTT2/SMARCB1/TXNRD1/CDK4/TCF7/RPS6KB1/SMARCE1/FZD2/SMARCD2/PRKCA/GRB2/TGFB3/AKT1/E2F3/TERT/PIK3R1/WNT5A/PBRM1/RB1/ARID2/SMARCD1/GSK3B/BAK1/APC/CSNK1A1/SMAD4/SMAD2/GSTP1/SMARCA2/FRAT1/GSTO1 | 66 | Human Diseases | Cancer: specific types | |
| hsa04210 | Apoptosis | 55/2309 | 136/7914 | 0.002992 | 0.021332 | 0.015746 | CASP8/AKT3/CAPN2/TRAF2/SPTAN1/ENDOG/TRAF1/DAB2IP/CTSZ/NTRK1/CTSK/NGF/NRAS/NFKB1/PIK3R3/DFFA/DFFB/PTPN13/CASP2/HTRA2/TNFRSF1A/KRAS/BAX/CTSC/MAPK3/CTSD/CASP3/CASP12/BIRC2/MAP2K1/CTSH/PIK3CB/PARP3/LMNB2/APAF1/MAPK9/BIRC5/EIF2S1/AKT1/PIK3R1/IL3RA/PARP2/CTSB/TNFRSF10B/TUBA1B/TUBA1A/PDPK1/BAK1/ITPR3/DAXX/LMNB1/CTSF/CASP7/XIAP/IKBKG | 55 | Cellular Processes | Cell growth and death | |
| hsa04071 | Sphingolipid signaling pathway | 49/2309 | 119/7914 | 0.003207 | 0.022382 | 0.016521 | AKT3/DEGS1/TRAF2/PLCB4/CERS2/NRAS/GNAI3/NFKB1/PIK3R3/PRKCZ/NOS3/RAC1/TNFRSF1A/KRAS/BAX/MAPK3/CTSD/CERS4/PPP2CB/S1PR2/PPP2R1B/MAP2K1/PIK3CB/GNAI2/RHOA/SMPD2/SGPL1/MAPK9/PLD2/PRKCA/GNA13/ROCK2/PPP2R5E/SGPP1/SPTLC2/DEGS2/PPP2R5C/AKT1/SPTLC1/PIK3R1/MAPK11/CERS5/ABCC1/PDPK1/MAPK14/MAPK13/ROCK1/GNAQ/SGMS1 | 49 | Environmental Information Processing | Signal transduction | |
| hsa04512 | ECM-receptor interaction | 38/2309 | 88/7914 | 0.00341 | 0.023305 | 0.017202 | COL4A4/COL6A3/LAMC2/LAMC1/TNN/LAMB3/ITGA8/ITGB6/ITGA6/ITGAV/CD44/LAMA5/TNC/AGRN/SPP1/COL1A2/COL4A1/COL4A2/ITGB1/ITGA9/LAMA2/LAMA4/COL6A2/COL6A1/ITGA7/COL1A1/ITGA3/ITGA2B/ITGB3/ITGB4/SDC1/LAMB1/ITGA1/ITGA5/THBS2/TNXB/LAMA3/COL4A6 | 38 | Environmental Information Processing | Signaling molecules and interaction | |
| hsa04926 | Relaxin signaling pathway | 52/2309 | 129/7914 | 0.004142 | 0.027724 | 0.020464 | COL3A1/CREB1/COL4A4/AKT3/CREB3L1/PLCB4/SRC/MMP9/GNB4/SHC1/NRAS/GNAI3/NFKB1/GNG5/PRKACB/TGFBR1/PIK3R3/PRKCZ/GNB1/NOS3/GNB2/GNG11/COL1A2/KRAS/TGFB1/ARRB1/MAPK3/COL4A1/COL4A2/VEGFC/ADCY7/GNAO1/MAP2K1/PIK3CB/GNAI2/TGFBR2/MAPK9/ARRB2/COL1A1/GNGT2/PRKCA/GRB2/AKT1/EDN1/PIK3R1/GNG2/MAPK11/MAPK14/MAPK13/SMAD2/ACTA2/COL4A6 | 52 | Organismal Systems | Endocrine system | |
| hsa04151 | PI3K-Akt signaling pathway | 126/2309 | 354/7914 | 0.00443 | 0.029055 | 0.021446 | CREB1/ERBB4/COL4A4/EIF4E2/COL6A3/LAMC2/LAMC1/TNN/AKT3/LAMB3/ITGA8/RXRA/PKN3/ITGB6/ITGA6/ITGAV/CREB3L1/BDNF/YWHAB/LAMA5/GNB4/TLR2/NTRK1/EFNA4/NGF/NRAS/CSF1/EGF/NFKB1/EIF4E/GNG5/CCNE2/LPAR1/TNC/TLR4/RPS6/TEK/JAK1/PIK3R3/CSF3R/MTOR/GNB1/CDK6/HGF/NOS3/YWHAH/PDGFRA/SPP1/YWHAG/GNB2/RAC1/FLT1/GNG11/COL1A2/MET/TGFA/MAGI1/CCND2/GYS2/KRAS/MAPK3/COL4A1/COL4A2/EIF4EBP1/PPP2CB/VEGFC/LPAR2/JAK3/ITGB1/CDC37/EPOR/PPP2R1B/MAP2K1/PIK3CB/ITGA9/LAMA2/LAMA4/COL6A2/COL6A1/CDK4/CDK2/ITGA7/FGF18/FLT4/YWHAE/RPS6KB1/COL1A1/ITGA3/GNGT2/ITGA2B/ITGB3/PRKCA/GRB2/ITGB4/YWHAQ/LAMB1/PPP2R5E/PGF/PPP2R5C/AKT1/F2R/PIK3R1/ITGA1/IL3RA/GNG2/PCK2/PRKAA1/OSMR/PRLR/PTK2/IL2RB/PDGFB/ITGA5/GSK3B/IFNAR2/IFNAR1/THBS2/PDPK1/MLST8/TNXB/EFNA5/LAMA3/PDGFRB/CSF1R/IKBKG/COL4A6 | 126 | Environmental Information Processing | Signal transduction | |
| hsa05166 | Human T-cell leukemia virus 1 infection | 82/2309 | 219/7914 | 0.004618 | 0.029055 | 0.021446 | IL1R1/CREB1/AKT3/CREB3L1/ANAPC1/E2F1/NRAS/NFKB1/PRKACB/CCNE2/TGFBR1/CDC26/JAK1/CDKN2C/PIK3R3/E2F2/ANAPC4/CHEK2/RAN/MAD1L1/MAD2L1/LTBR/TNFRSF1A/CCND2/KRAS/TGFB1/BAX/MAPK3/ITGAL/BUB3/VDAC3/POLB/JAK3/ANAPC10/IL15/ADCY7/NFATC3/NRP1/ETS1/CHEK1/SMAD3/MAP2K1/TLN2/PIK3CB/TGFBR2/TBPL1/NFYB/CDK4/CDK2/PPP3R1/XPO1/PTTG1/MAPK9/CANX/VDAC1/GPS2/STAT5A/CDC27/ANAPC11/TGFB3/AKT1/E2F3/TERT/PIK3R1/MAP3K1/PPP3CB/VDAC2/NFATC4/RB1/IL2RB/EP300/TSPO/RANBP1/DLG1/RANBP3/CDC23/SMAD4/SMAD2/NFATC1/NFKB2/XIAP/IKBKG | 82 | Human Diseases | Infectious disease: viral | |
| hsa04015 | Rap1 signaling pathway | 79/2309 | 210/7914 | 0.004669 | 0.029055 | 0.021446 | RALB/AKT3/ENAH/APBB1IP/VAV2/RAPGEF1/RAPGEF4/PLCB4/SRC/PRKCI/PFN2/EFNA4/NGF/NRAS/MAGI3/CSF1/GNAI3/EGF/LPAR1/TEK/PIK3R3/CDC42/PRKCZ/KRIT1/HGF/PDGFRA/RAC1/FLT1/MET/MAGI1/KRAS/PRKD2/VASP/RRAS/LAT/MAPK3/ITGAL/VEGFC/LPAR2/ADCY7/GNAO1/ITGB1/MAP2K1/TLN2/PIK3CB/GNAI2/RHOA/CTNNB1/FGF18/FLT4/RAPGEF6/ADORA2B/PFN1/ITGA2B/ITGB3/PRKCA/MAP2K6/DOCK4/PGF/CALM1/EVL/AKT1/CALML3/RALA/F2R/PIK3R1/PDGFB/MAPK11/CRKL/TIAM1/MAPK14/MAPK13/EFNA5/PDGFRB/CSF1R/PARD6G/SIPA1/GNAQ/PLCE1 | 79 | Environmental Information Processing | Signal transduction | |
| hsa05205 | Proteoglycans in cancer | 77/2309 | 204/7914 | 0.004695 | 0.029055 | 0.021446 | ERBB4/AKT3/VAV2/ITGAV/CD44/SRC/MMP9/TLR2/NRAS/CAMK2D/PRKACB/TLR4/RPS6/PIK3R3/CDC42/MTOR/HGF/PTPN11/RAC1/COL1A2/CAV2/CAV1/MET/FLNC/KRAS/TGFB1/RRAS/IQGAP1/PAK1/RRAS2/MAPK3/CTTN/CASP3/GAB1/ITGB1/ARHGEF12/CBL/MAP2K1/PIK3CB/RHOA/CTNNB1/TIMP3/LUM/PPP1R12A/FRS2/CD63/CAMK2B/RPS6KB1/COL1A1/FZD2/ITGB3/PRKCA/GRB2/SDC1/ROCK2/TWIST1/HIF1A/AKT1/PIK3R1/FLNB/PLAU/WNT5A/SDC2/PTK2/MAPK11/ITGA5/TIAM1/PDPK1/ITPR3/MAPK14/MAPK13/ROCK1/SMAD2/PLCE1/GPC3/FLNA/MSN | 77 | Human Diseases | Cancer: overview | |
| hsa04914 | Progesterone-mediated oocyte maturation | 41/2309 | 98/7914 | 0.004795 | 0.0291 | 0.021479 | AKT3/ANAPC1/AURKA/GNAI3/PRKACB/CDC26/PIK3R3/RPS6KA1/MAD2L2/ANAPC4/MAD1L1/MAD2L1/KRAS/CPEB1/PDE3B/PLK1/MAPK3/KIF22/ANAPC10/ADCY7/MAP2K1/PIK3CB/ANAPC13/GNAI2/CDC25A/CDK1/CDK2/MAPK9/CDC27/ANAPC11/AKT1/CCNB1/PIK3R1/MAPK11/PKMYT1/MAPK14/MAPK13/CDC23/CPEB3/RPS6KA6/RPS6KA3 | 41 | Organismal Systems | Endocrine system | |
| hsa05165 | Human papillomavirus infection | 118/2309 | 330/7914 | 0.00488 | 0.0291 | 0.021479 | CASP8/CREB1/COL4A4/COL6A3/HES6/LAMC2/LAMC1/TNN/AKT3/LAMB3/ITGA8/ITGB6/ITGA6/ITGAV/CREB3L1/E2F1/RBL1/LAMA5/HEY1/PRKCI/MAML3/NRAS/EGF/NFKB1/PRKACB/CCNE2/NFX1/TNC/JAK1/PIK3R3/HEYL/HDAC1/CDC42/MTOR/PRKCZ/CDK6/RBPJ/SPP1/LFNG/COL1A2/ATP6V1F/MAGI1/ATP6V1E1/CHD4/TNFRSF1A/CCND2/KRAS/BAX/MAPK3/COL4A1/COL4A2/EIF4EBP1/PPP2CB/TLR3/CASP3/ITGB1/MAML2/PPP2R1B/PKM/MAP2K1/PIK3CB/ITGA9/CTNNB1/TBPL1/LAMA2/LAMA4/COL6A2/COL6A1/CDK4/STAT2/CDK2/ITGA7/TCF7/LLGL1/RPS6KB1/COL1A1/ITGA3/TUBG2/ITGA2B/FZD2/ITGB3/GRB2/LLGL2/ITGB4/ATP6V1C2/LAMB1/PPP2R5E/PPP2R5C/TRAF3/AKT1/TERT/PIK3R1/ITGA1/WNT5A/RB1/PTGER4/ATP6V1C1/PTK2/EP300/ITGA5/DLG1/GSK3B/IFNAR2/IFNAR1/THBS2/BAK1/NOTCH3/NOTCH4/TNXB/EIF2AK2/LAMA3/APC/PDGFRB/CSNK1A1/PARD6G/BCAP31/IKBKG/COL4A6 | 118 | Human Diseases | Infectious disease: viral | |
| hsa05012 | Parkinson disease | 56/2309 | 142/7914 | 0.005217 | 0.030555 | 0.022553 | COX5B/NDUFA10/SDHC/NDUFS2/NDUFS3/NDUFB5/NDUFC1/GNAI3/PRKACB/NDUFB6/UQCRH/NDUFS5/PINK1/SDHB/PARK7/HTRA2/NDUFA9/NDUFA3/COX7A1/COX6B1/NDUFC2/NDUFAB1/COX6A2/VDAC3/CASP3/NDUFA13/SDHD/COX7A2/GNAI2/UBA7/UQCRC1/UQCR11/APAF1/NDUFA12/UQCR10/VDAC1/UQCRQ/UBE2G1/UQCRFS1/NDUFS6/COX7C/VDAC2/NDUFB9/CYC1/NDUFA6/NDUFB4/NDUFV3/NDUFA11/NDUFV2/COX7A2L/NDUFS8/NDUFV1/COX8A/NDUFB8/NDUFB11/UBA1 | 56 | Human Diseases | Neurodegenerative disease | |
| hsa05223 | Non-small cell lung cancer | 29/2309 | 66/7914 | 0.007366 | 0.042385 | 0.031285 | AKT3/RXRA/E2F1/STK4/NRAS/EGF/PIK3R3/E2F2/CDK6/TGFA/KRAS/BAX/MAPK3/JAK3/MAP2K1/PIK3CB/CDK4/STAT5A/PRKCA/GRB2/AKT1/E2F3/PIK3R1/FHIT/RARB/RB1/PDPK1/BAK1/EML4 | 29 | Human Diseases | Cancer: specific types | |
| hsa04120 | Ubiquitin mediated proteolysis | 53/2309 | 136/7914 | 0.008485 | 0.047985 | 0.035419 | UBE2F/UBE2E3/ANAPC1/FBXW7/CDC26/FBXO2/UBE3C/ANAPC4/UBE2K/UBA6/SMURF1/UBA3/SAE1/CBLC/UBA2/HERC2/ANAPC10/WWP2/BIRC2/CBL/HERC1/NEDD4/ANAPC13/UBA7/RHOBTB1/UBE2D1/UBE2N/FANCL/FBXW11/UBE2G1/TRIM37/UBE2Z/CDC27/ANAPC11/ERCC8/MAP3K1/FBXO4/SKP2/UBR5/RBX1/UBE2I/CUL2/CDC23/NEDD4L/DDB1/PRPF19/UBA1/KLHL13/UBE2A/CUL4B/XIAP/HUWE1/MID1 | 53 | Genetic Information Processing | Folding, sorting and degradation | |
| hsa04392 | Hippo signaling pathway - multiple species | 15/2309 | 29/7914 | 0.008757 | 0.048685 | 0.035936 | WWTR1/RASSF6/MOB1A/TEAD4/WTIP/TEAD2/PAK1/TEAD1/YAP1/LATS1/SAV1/FRMD6/AJUBA/LATS2/STK3 | 15 | Environmental Information Processing | Signal transduction | |
| hsa04625 | C-type lectin receptor signaling pathway | 42/2309 | 104/7914 | 0.009011 | 0.049261 | 0.036361 | CASP8/AKT3/IL1B/SRC/NRAS/NFKB1/PIK3R3/PTPN11/CLEC4D/KRAS/BCL3/RRAS/PAK1/RRAS2/MAPK3/PYCARD/LSP1/CYLD/NFATC3/CASP1/ARHGEF12/PIK3CB/RHOA/STAT2/PPP3R1/MAPK9/KSR1/CALM1/AKT1/CALML3/PIK3R1/PPP3CB/PRKCD/NFATC4/IL17D/MAPK11/ITPR3/MAPK14/MAPK13/NFATC1/NFKB2/IKBKG | 42 | Organismal Systems | Immune system | |
| hsa04140 | Autophagy - animal | 53/2309 | 137/7914 | 0.009997 | 0.053754 | 0.039677 | ATG16L1/AKT3/TANK/ATG13/EIF2AK4/RAB33B/NRAS/SH3GLB1/PRKACB/MAP3K7/RRAGA/PIK3R3/RRAGC/MTOR/ULK1/CAMKK2/WIPI2/VAMP8/MTMR14/ATG7/KRAS/RRAS/UVRAG/RRAS2/MAPK3/CTSD/IRS2/LAMP1/PPP2CB/MAP2K1/DAPK2/PIK3CB/DAPK3/MAPK9/GABARAP/RPS6KB1/VMP1/BECN1/HIF1A/EIF2S1/ZFYVE1/ATG2B/AKT1/DAPK1/WDR41/PIK3R1/PRKCD/CTSB/PRKAA1/ATG101/PDPK1/MLST8/LAMP2 | 53 | Cellular Processes | Transport and catabolism | |
| hsa00260 | Glycine, serine and threonine metabolism | 19/2309 | 40/7914 | 0.010673 | 0.05545 | 0.040929 | SARDH/GATM/PHGDH/GRHPR/DAO/SDSL/PSPH/GCSH/PGAM2/AOC2/BHMT/DMGDH/AGXT2/GCAT/CBS/GNMT/ALDH7A1/PSAT1/MAOA | 19 | Metabolism | Amino acid metabolism | |
| hsa05146 | Amoebiasis | 41/2309 | 102/7914 | 0.010716 | 0.05545 | 0.040929 | IL1R1/COL3A1/COL4A4/LAMC2/LAMC1/LAMB3/C8G/IL1B/PLCB4/LAMA5/TLR2/NFKB1/PRKACB/TLR4/C8A/PIK3R3/PRDX1/COL1A2/TGFB1/ACTN4/COL4A1/COL4A2/CASP3/PIK3CB/LAMA2/LAMA4/COL1A1/RAB5C/PRKCA/LAMB1/ACTN1/TGFB3/SERPINB9/PIK3R1/VCL/PTK2/LAMA3/CD14/GNAQ/GNA14/COL4A6 | 41 | Human Diseases | Infectious disease: parasitic | |
| hsa03008 | Ribosome biogenesis in eukaryotes | 42/2309 | 105/7914 | 0.010872 | 0.05545 | 0.040929 | IMP4/WDR75/NOP58/NVL/RPP38/NAT10/NOP10/NOP56/NXT1/EIF6/WDR3/GAR1/MDN1/GNL2/RAN/POP7/BMS1/FBL/POP4/CSNK2A2/NOB1/REXO2/XRN1/PWP2/REXO1/XPO1/NHP2/UTP6/UTP18/HEATR1/RPP40/UTP15/GNL3/RRP7A/LSG1/TBL3/WDR43/WDR36/TCOF1/RPP30/UTP14A/DKC1 | 42 | Genetic Information Processing | Translation | |
| hsa01521 | EGFR tyrosine kinase inhibitor resistance | 33/2309 | 79/7914 | 0.010989 | 0.05545 | 0.040929 | EIF4E2/AKT3/SRC/SHC1/NRAS/EGF/EIF4E/RPS6/JAK1/PIK3R3/MTOR/HGF/PDGFRA/MET/TGFA/KRAS/AXL/BAX/MAPK3/EIF4EBP1/NRG1/GAB1/MAP2K1/PIK3CB/NF1/RPS6KB1/PRKCA/GRB2/AKT1/PIK3R1/PDGFB/GSK3B/PDGFRB | 33 | Human Diseases | Drug resistance: antineoplastic | |
| hsa00190 | Oxidative phosphorylation | 51/2309 | 133/7914 | 0.013683 | 0.065403 | 0.048276 | COX5B/NDUFA10/SDHC/NDUFS2/NDUFS3/NDUFB5/NDUFC1/PPA2/NDUFB6/UQCRH/NDUFS5/SDHB/ATP6V1F/ATP6V1E1/NDUFA9/NDUFA3/COX7A1/COX6B1/ATP4A/NDUFC2/NDUFAB1/COX6A2/LHPP/NDUFA13/SDHD/COX7A2/UQCRC1/PPA1/UQCR11/NDUFA12/UQCR10/UQCRQ/COX10/ATP6V1C2/UQCRFS1/NDUFS6/COX7C/ATP6V1C1/NDUFB9/CYC1/NDUFA6/NDUFB4/NDUFV3/NDUFA11/NDUFV2/COX7A2L/NDUFS8/NDUFV1/COX8A/NDUFB8/NDUFB11 | 51 | Metabolism | Energy metabolism | |
| hsa00532 | Glycosaminoglycan biosynthesis - chondroitin sulfate / dermatan sulfate | 11/2309 | 20/7914 | 0.013708 | 0.065403 | 0.048276 | CHST14/B3GALT6/CHPF2/CHST12/CHST13/XYLT1/UST/DSE/CHST3/B4GALT7/CHST7 | 11 | Metabolism | Glycan biosynthesis and metabolism | |
| hsa00270 | Cysteine and methionine metabolism | 22/2309 | 49/7914 | 0.013759 | 0.065403 | 0.048276 | AHCY/GSS/PHGDH/GCLM/MTAP/SRM/ENOPH1/SDSL/AHCYL2/LDHB/BCAT1/MRI1/DNMT1/GCLC/AMD1/BHMT/BHMT2/AGXT2/TST/CBS/CDO1/PSAT1 | 22 | Metabolism | Amino acid metabolism | |
| hsa00510 | N-Glycan biosynthesis | 22/2309 | 49/7914 | 0.013759 | 0.065403 | 0.048276 | MGAT4A/B4GALT3/DOLPP1/DPM2/ALG5/MAN1A2/ALG14/ALG6/MAN1C1/DDOST/SRD5A3/ALG8/STT3A/ALG9/MGAT4B/MGAT2/ALG12/ALG10B/ALG3/ST6GAL1/MAN2A1/ALG13 | 22 | Metabolism | Glycan biosynthesis and metabolism | |
| hsa05235 | PD-L1 expression and PD-1 checkpoint pathway in cancer | 36/2309 | 89/7914 | 0.014461 | 0.066214 | 0.048874 | AKT3/RASGRP1/TLR2/NRAS/EGF/NFKB1/TLR4/JAK1/PIK3R3/MTOR/PTPN11/KRAS/LAT/MAPK3/CSNK2A2/NFATC3/TIRAP/MAP2K1/PIK3CB/MYD88/IFNGR1/PPP3R1/RPS6KB1/MAP2K6/HIF1A/AKT1/PIK3R1/PPP3CB/MAPK11/IFNGR2/MAPK14/MAPK13/NFKBIE/EML4/NFATC1/IKBKG | 36 | Human Diseases | Cancer: overview | |
| hsa03440 | Homologous recombination | 19/2309 | 41/7914 | 0.014554 | 0.066214 | 0.048874 | RAD51/NBN/RPA2/XRCC2/RPA3/SSBP1/RAD52/POLD1/BLM/POLD3/PALB2/TOPBP1/POLD2/RAD50/TOP3A/RPA1/XRCC3/RBBP8/POLD4 | 19 | Genetic Information Processing | Replication and repair | |
| hsa00410 | beta-Alanine metabolism | 16/2309 | 33/7914 | 0.01471 | 0.066214 | 0.048874 | ALDH9A1/SMOX/SRM/ACOX3/ACADS/ALDH2/ECHS1/MLYCD/UPB1/AOC2/ACOX1/DPYS/ABAT/ALDH7A1/CNDP1/ALDH3B1 | 16 | Metabolism | Metabolism of other amino acids | |
| hsa05163 | Human cytomegalovirus infection | 81/2309 | 225/7914 | 0.014737 | 0.066214 | 0.048874 | IL1R1/CASP8/CREB1/CXCR2/AKT3/TRAF5/TRAF2/ITGAV/CREB3L1/PDIA3/IL1B/PLCB4/E2F1/SRC/GNB4/NRAS/GNAI3/NFKB1/GNG5/PRKACB/JAK1/PIK3R3/E2F2/MTOR/GNB1/CDK6/PDGFRA/GNB2/RAC1/GNG11/TNFRSF1A/KRAS/BAX/MAPK3/EIF4EBP1/CASP3/ADCY7/GNAO1/CX3CL1/NFATC3/ARHGEF12/MAP2K1/PIK3CB/GNAI2/RHOA/CTNNB1/CCR1/CDK4/PPP3R1/RPS6KB1/GNGT2/ITGB3/PRKCA/GNA13/MAP2K6/GRB2/ROCK2/CALM1/AKT1/CALML3/E2F3/PIK3R1/GNG2/PPP3CB/NFATC4/RB1/PTGER4/PTK2/MAPK11/SP1/CRKL/GSK3B/BAK1/ITPR3/MAPK14/MAPK13/ROCK1/TMEM173/NFATC1/GNAQ/IKBKG | 81 | Human Diseases | Infectious disease: viral | |
| hsa05160 | Hepatitis C | 58/2309 | 155/7914 | 0.015678 | 0.068636 | 0.050662 | CASP8/RNASEL/AKT3/TRAF2/RXRA/EIF2AK4/E2F1/YWHAB/NRAS/EGF/NFKB1/DDX58/JAK1/PIK3R3/E2F2/CDK6/YWHAH/OAS2/SCARB1/YWHAG/CLDN15/EIF2AK1/TNFRSF1A/KRAS/BAX/MAPK3/PPP2CB/TLR3/CASP3/PPP2R1B/MAP2K1/PIK3CB/CTNNB1/APAF1/CDK4/STAT2/CDK2/CLDN7/YWHAE/PSME3/GRB2/YWHAQ/EIF2S1/TRAF3/AKT1/E2F3/PIK3R1/RB1/CLDN10/CLDN1/CLDN16/GSK3B/CLDN25/IFNAR2/IFNAR1/BAK1/EIF2AK2/IKBKG | 58 | Human Diseases | Infectious disease: viral | |
| hsa01230 | Biosynthesis of amino acids | 31/2309 | 75/7914 | 0.015904 | 0.068636 | 0.050662 | IDH1/PYCR2/ASS1/PKLR/PHGDH/ACO1/SDSL/PSPH/ASL/ASNS/RPIA/ENO2/TPI1/BCAT1/IDH2/TALDO1/GPT2/PKM/ACY1/PAH/PGAM2/ALDOC/PFKP/TKT/GPT/ACO2/CBS/PSAT1/ALDH18A1/IDH3G/PRPS1 | 31 | Metabolism | Global and overview maps | |
| hsa05214 | Glioma | 31/2309 | 75/7914 | 0.015904 | 0.068636 | 0.050662 | AKT3/CAMK1G/E2F1/SHC1/NRAS/CAMK2D/EGF/PIK3R3/E2F2/MTOR/CDK6/PDGFRA/TGFA/KRAS/BAX/MAPK3/MAP2K1/PIK3CB/CDK4/CAMK2B/PRKCA/GRB2/CALM1/AKT1/CALML3/E2F3/PIK3R1/RB1/PDGFB/BAK1/PDGFRB | 31 | Human Diseases | Cancer: specific types | |
| hsa00330 | Arginine and proline metabolism | 22/2309 | 50/7914 | 0.017944 | 0.076436 | 0.056419 | ALDH9A1/PYCR2/GATM/SMOX/AZIN2/ALDH4A1/AGMAT/SRM/NOS3/LAP3/DAO/ALDH2/AOC1/PRODH2/AMD1/P4HA1/ODC1/PRODH/ALDH7A1/CNDP1/ALDH18A1/MAOA | 22 | Metabolism | Amino acid metabolism | |
| hsa03018 | RNA degradation | 32/2309 | 79/7914 | 0.019902 | 0.083691 | 0.061775 | EXOSC2/EXOSC8/EXOSC3/PABPC4/EXOSC10/LSM8/CNOT4/LSM5/LSM3/ENO2/LSM1/CNOT7/LSM6/CNOT1/MPHOSPH6/EDC3/XRN1/NUDT16/EXOSC7/LSM7/PNPT1/CNOT6/PFKP/TTC37/DCP1A/DIS3/PABPC1/PARN/LSM2/DCP2/PATL1/EXOSC1 | 32 | Genetic Information Processing | Folding, sorting and degradation | |
| hsa04714 | Thermogenesis | 82/2309 | 231/7914 | 0.020478 | 0.085021 | 0.062756 | COX5B/COA5/CREB1/NDUFA10/SDHC/NDUFS2/NDUFS3/CREB3L1/NDUFAF5/ACTL6A/NDUFB5/NDUFC1/PRKAB2/NRAS/PRKACB/NDUFAF6/NDUFB6/RPS6/UQCRH/NDUFS5/RPS6KA1/KDM1A/SDHB/MTOR/NDUFA9/KRAS/NDUFA3/COX7A1/COX6B1/NDUFC2/COA4/NDUFAB1/COX6A2/NDUFA13/UCP1/ADCY7/SMARCA4/SDHD/COX7A2/NDUFAF3/UQCRC1/SMARCC1/SMARCB1/UQCR11/NDUFA12/FRS2/UQCR10/UQCRQ/COX10/RPS6KB1/SMARCE1/COA3/SMARCD2/GRB2/UQCRFS1/NDUFS6/COX7C/PRKAA1/NDUFB9/CYC1/NDUFA6/MAPK11/SMARCD1/NDUFB4/MLST8/MAPK14/MAPK13/NDUFV3/NDUFA11/NDUFV2/COX7A2L/KDM3B/NDUFS8/NDUFV1/COX8A/SMARCA2/NDUFB8/ACSL5/NDUFB11/RPS6KA6/ACSL4/RPS6KA3 | 82 | Organismal Systems | Environmental adaptation | |
| hsa04350 | TGF-beta signaling pathway | 37/2309 | 94/7914 | 0.021194 | 0.086894 | 0.064139 | BMPR2/GREM2/LEFTY1/FBN1/RBL1/BMPR1B/TGFBR1/ID3/NBL1/SMURF1/TGFB1/RGMA/MAPK3/TFDP1/PPP2CB/E2F4/PPP2R1B/NEO1/SMAD3/RHOA/TGFBR2/ACVR2B/RPS6KB1/ID2/TGFB3/BMP6/SMAD5/ZFYVE16/FST/RBX1/EP300/SP1/RGMB/BAMBI/ROCK1/SMAD4/SMAD2 | 37 | Environmental Information Processing | Signal transduction | |
