# Supplementary material for: Sodium aescinate protects renal ischemia-reperfusion and pyroptosis through AKT/NLRP3 signaling pathway
Source: Ren Fail. 2025 Apr 22;47(1):2488140. doi: 10.1080/0886022X.2025.2488140 (PMC12016278; doi:10.1080/0886022X.2025.2488140)
Supplement: Raw data.docx [file IRNF_A_2488140_SM1628.docx]

Figure 1C
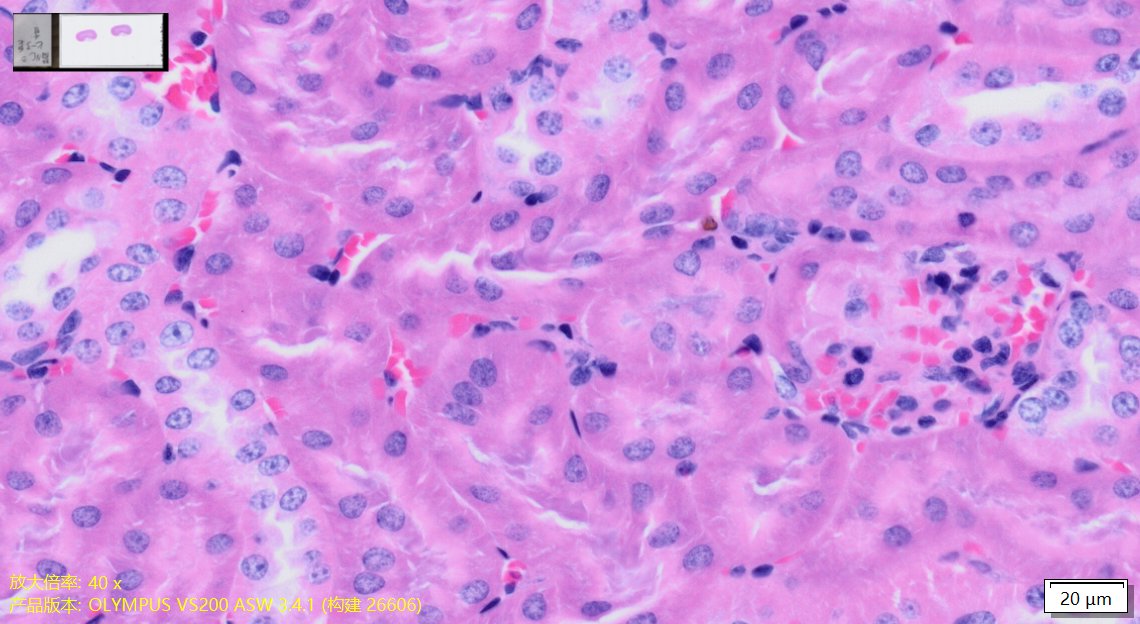


Sham1


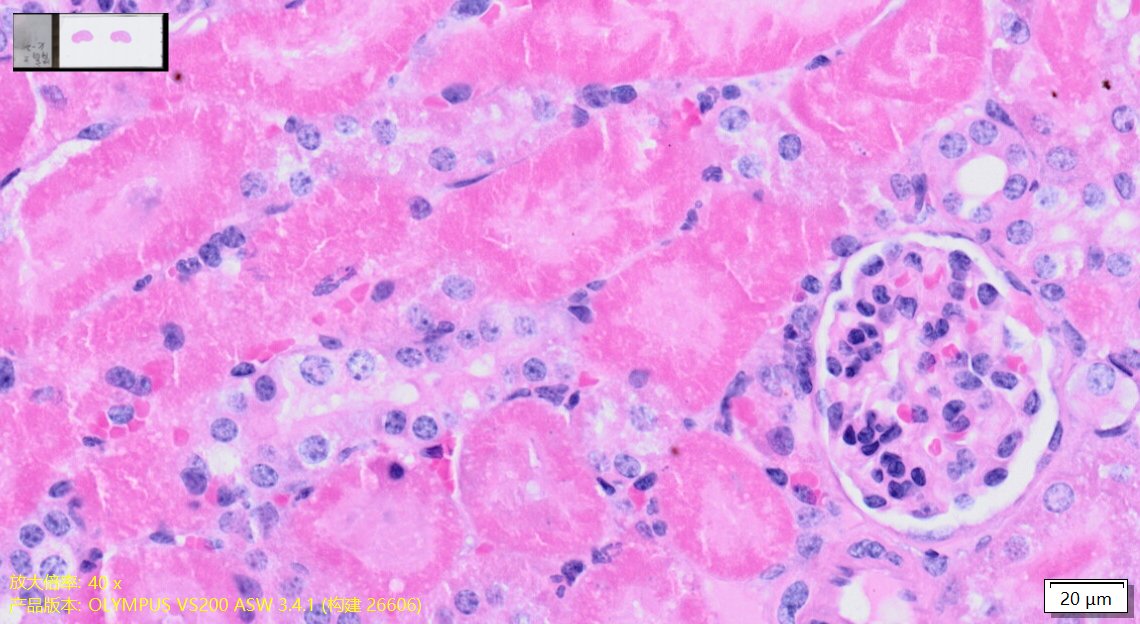


RIRI


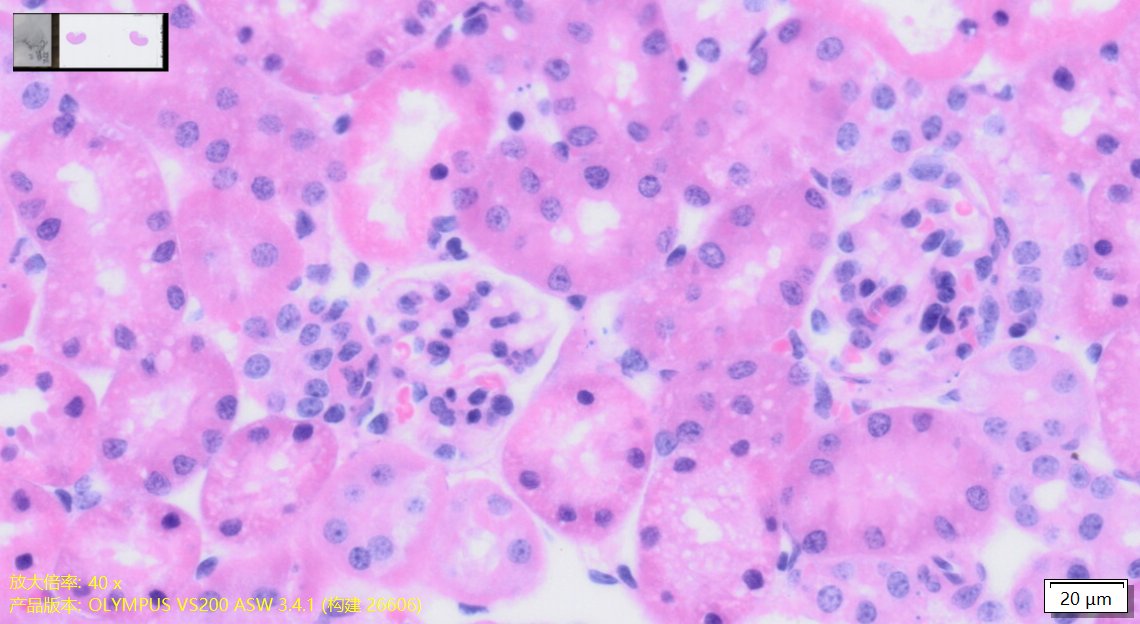


SA-L


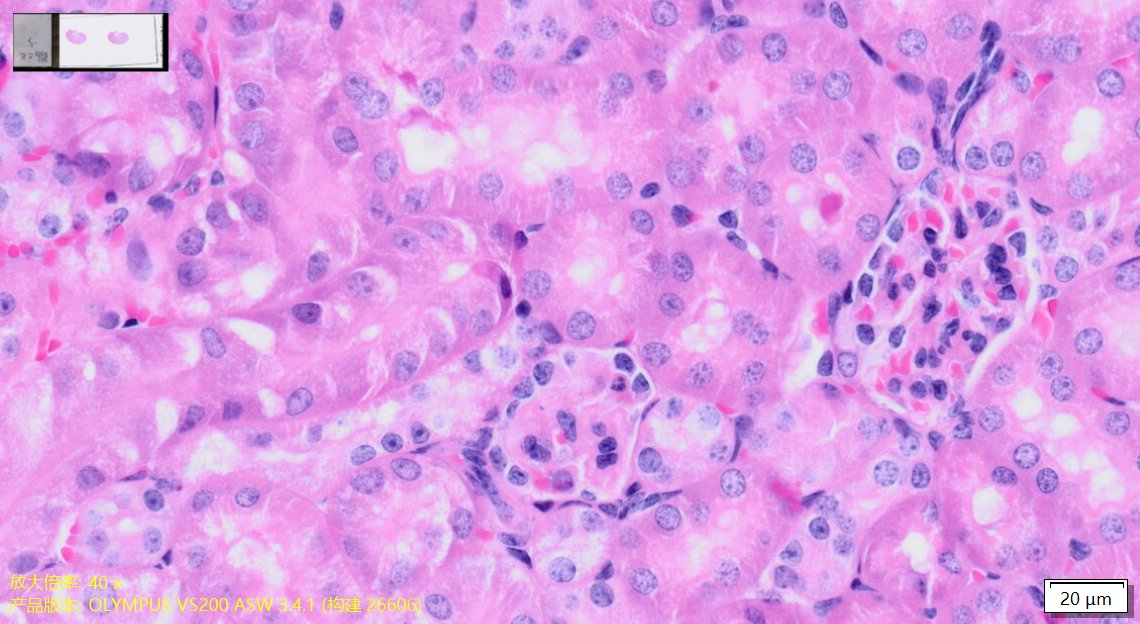


Sa-H

Figure 1D KIM-1
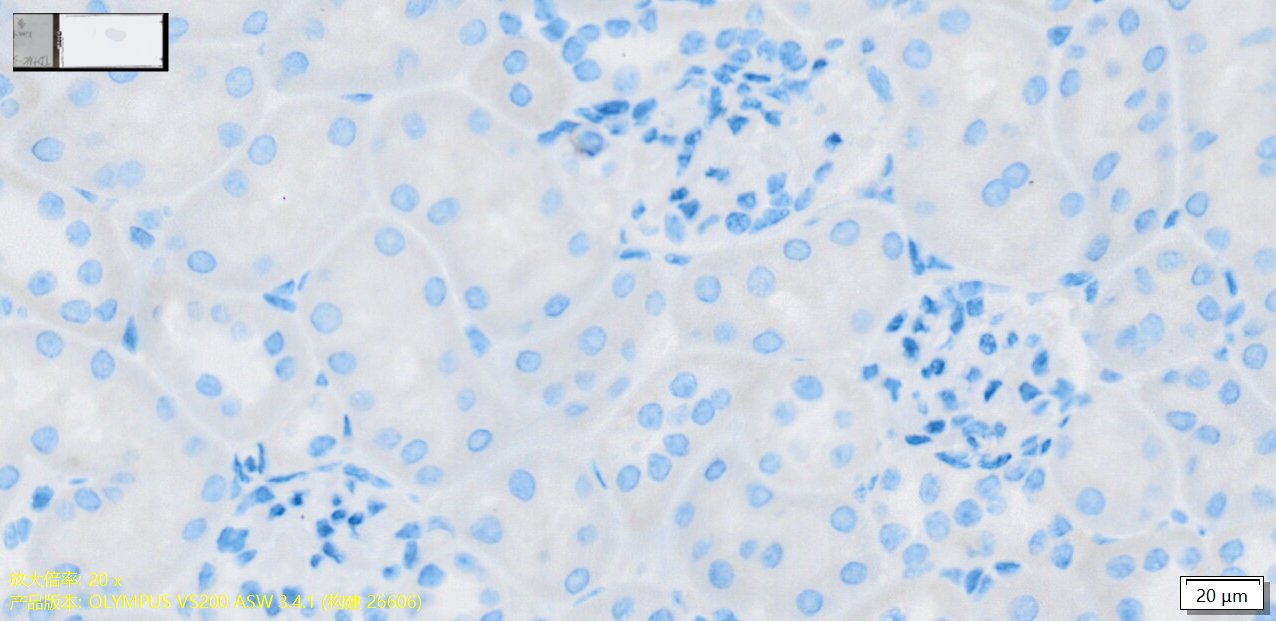


Sham


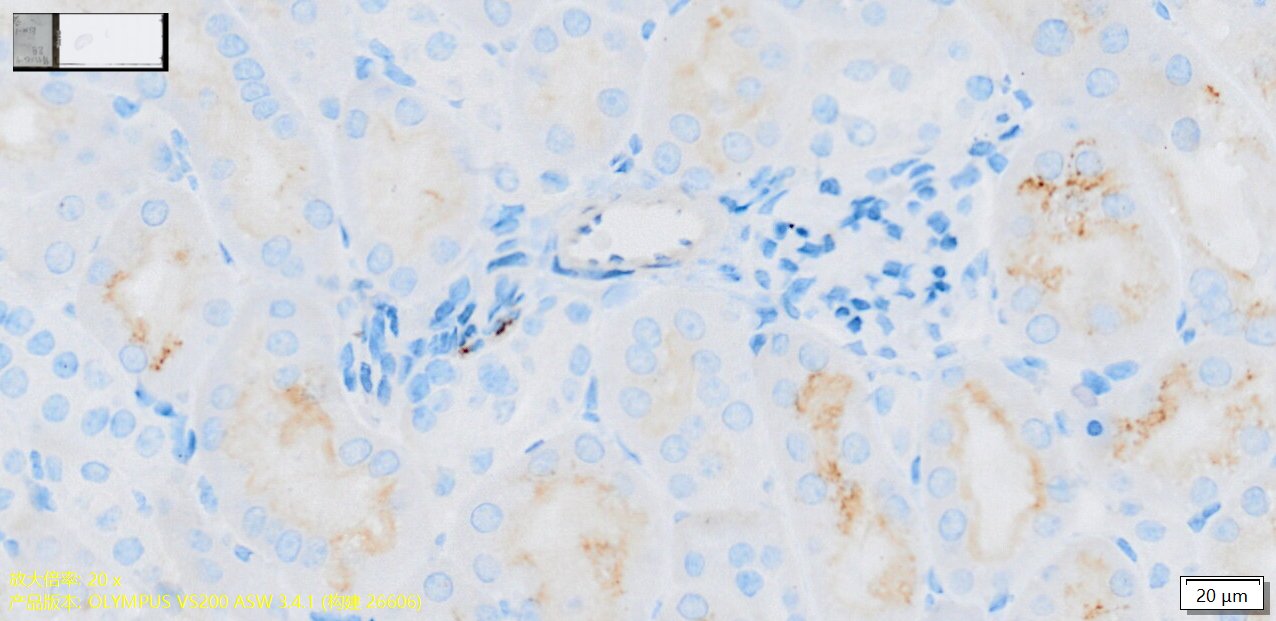


RIRI


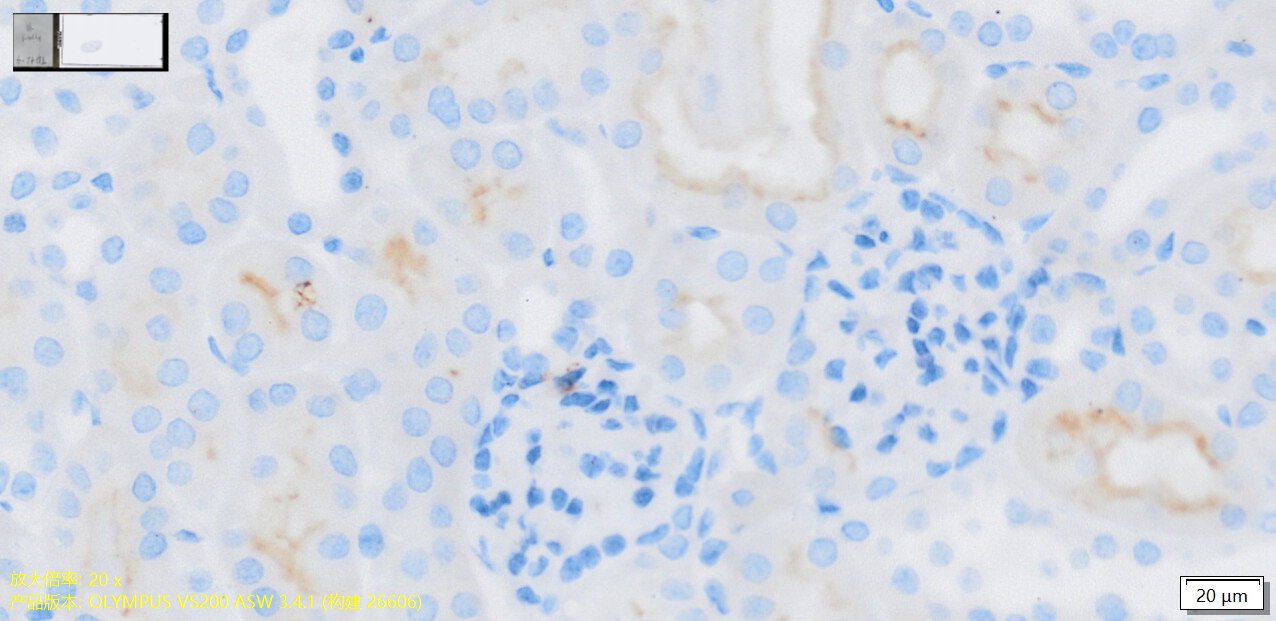


SA-L


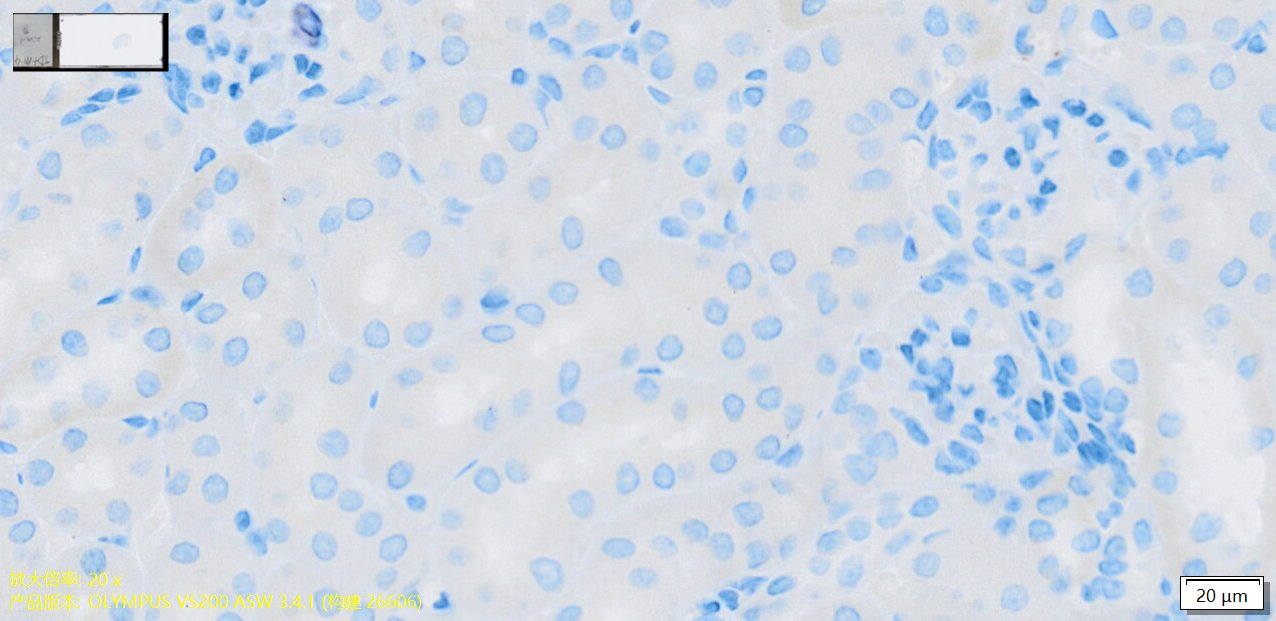


SA-H

Figure 1E NGAL


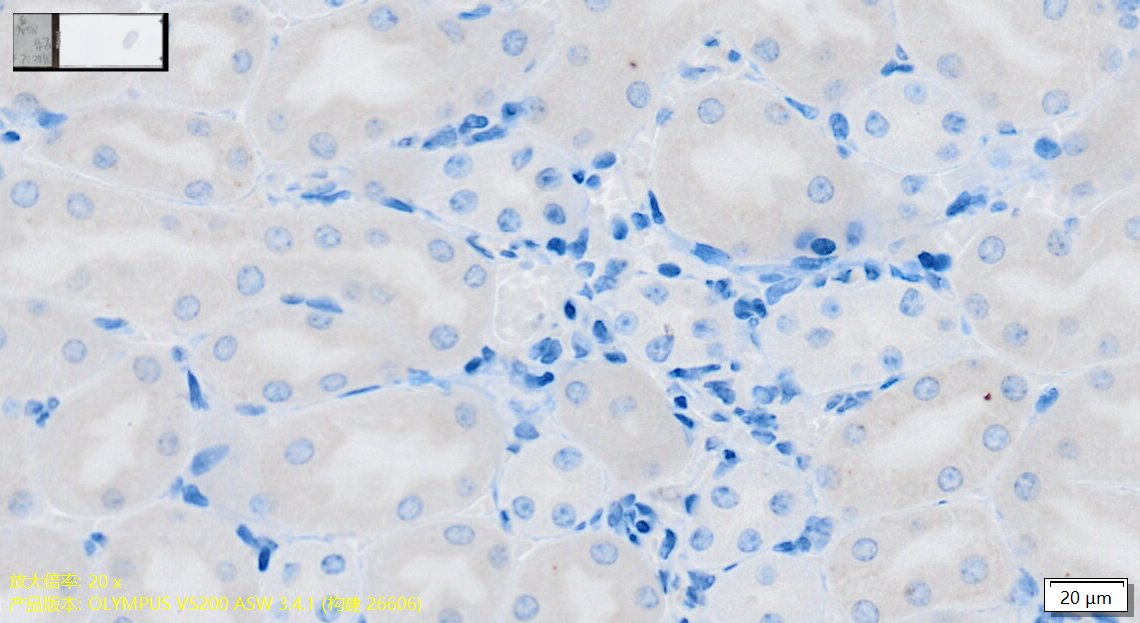


Sham


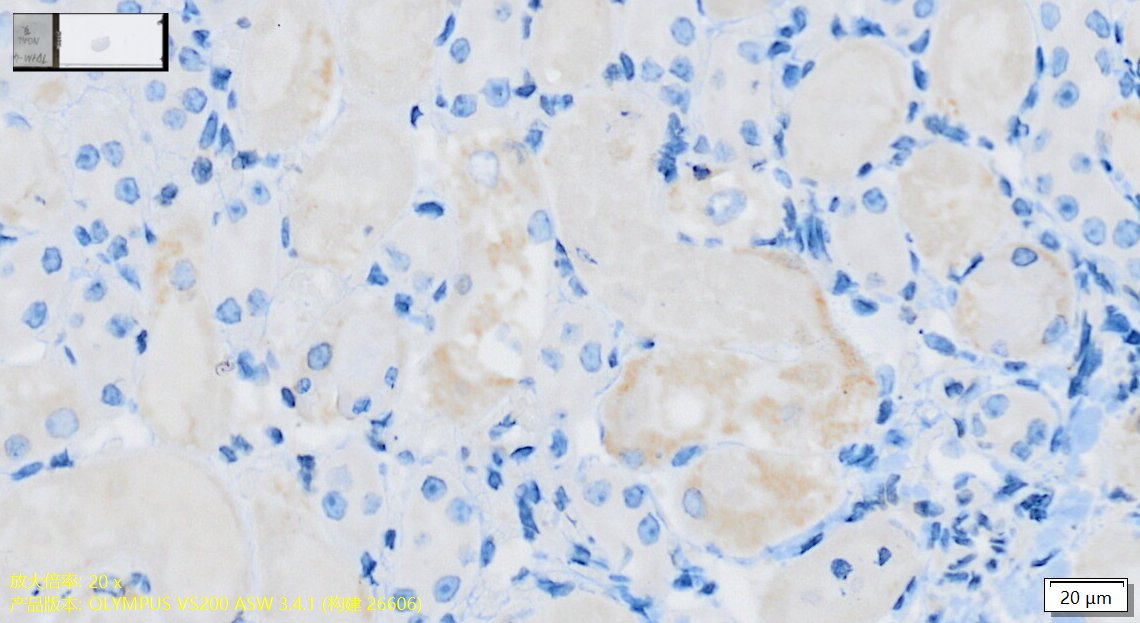


RIRI


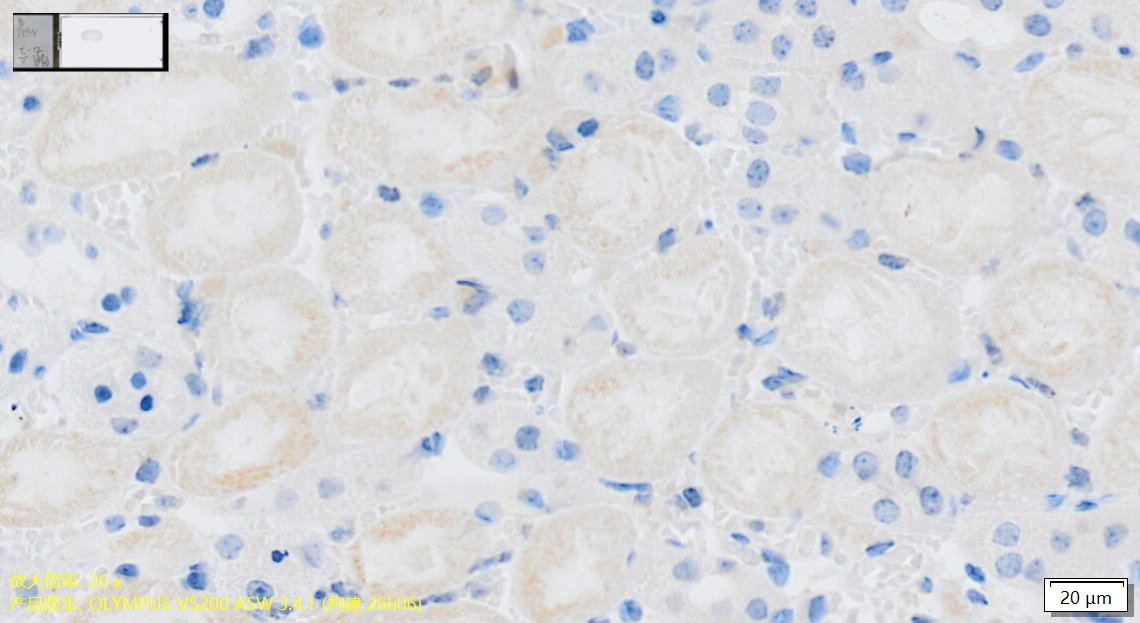


SA-L


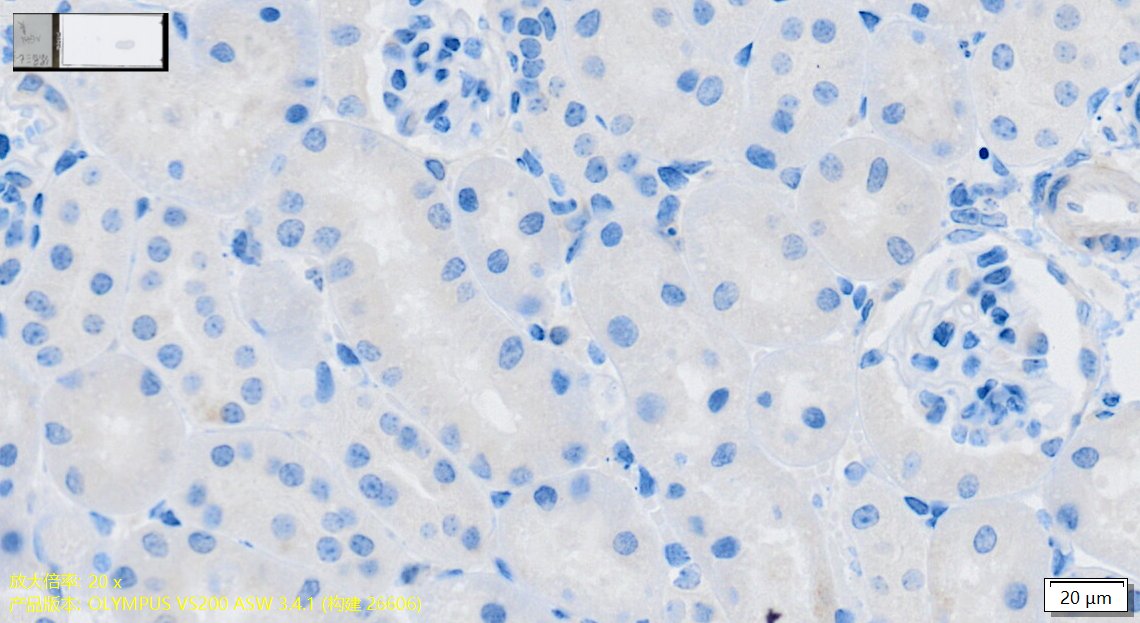


Sa-H

Figure 1H


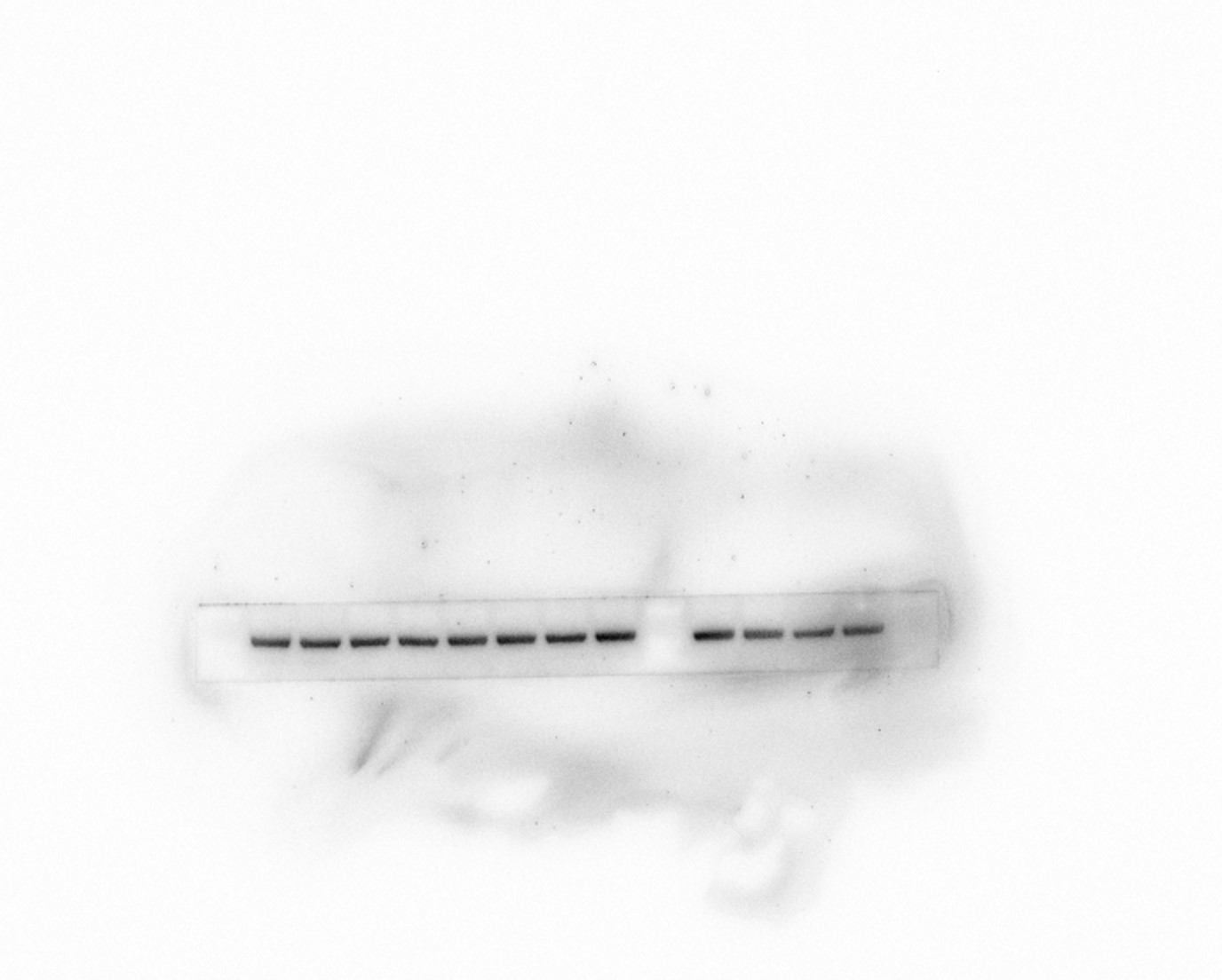


Actin





KIM-1


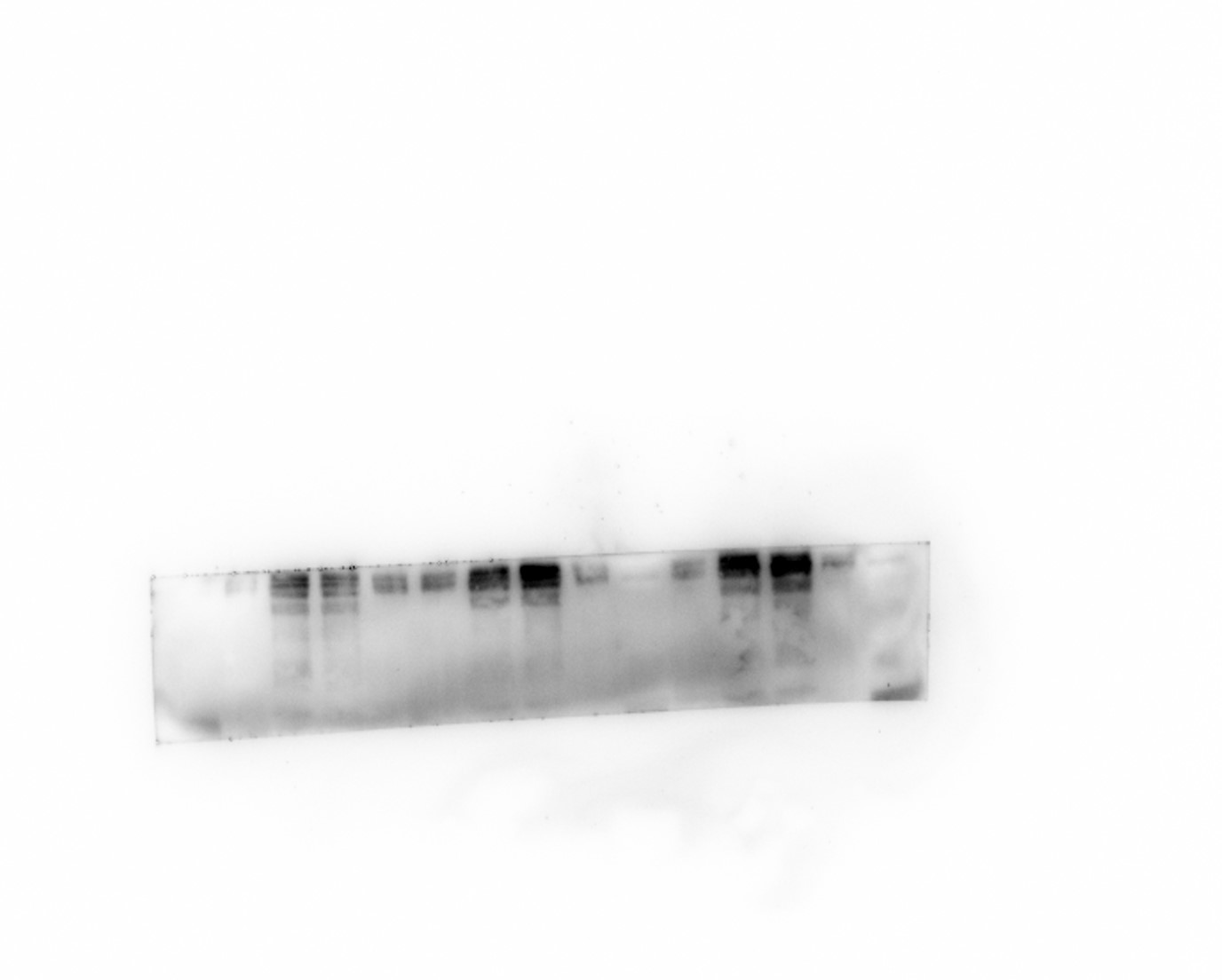


NGAL

Figure 2F


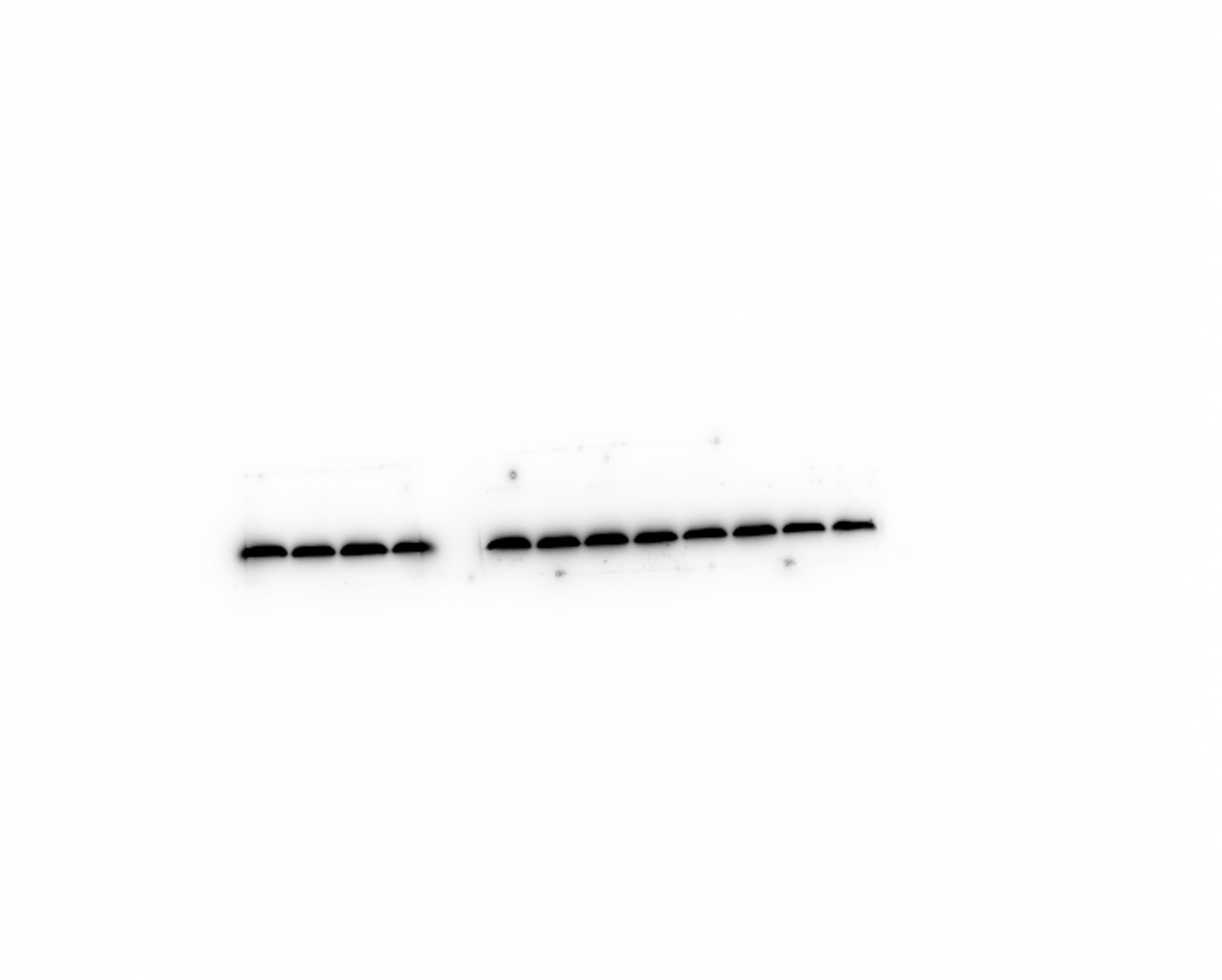


Actin


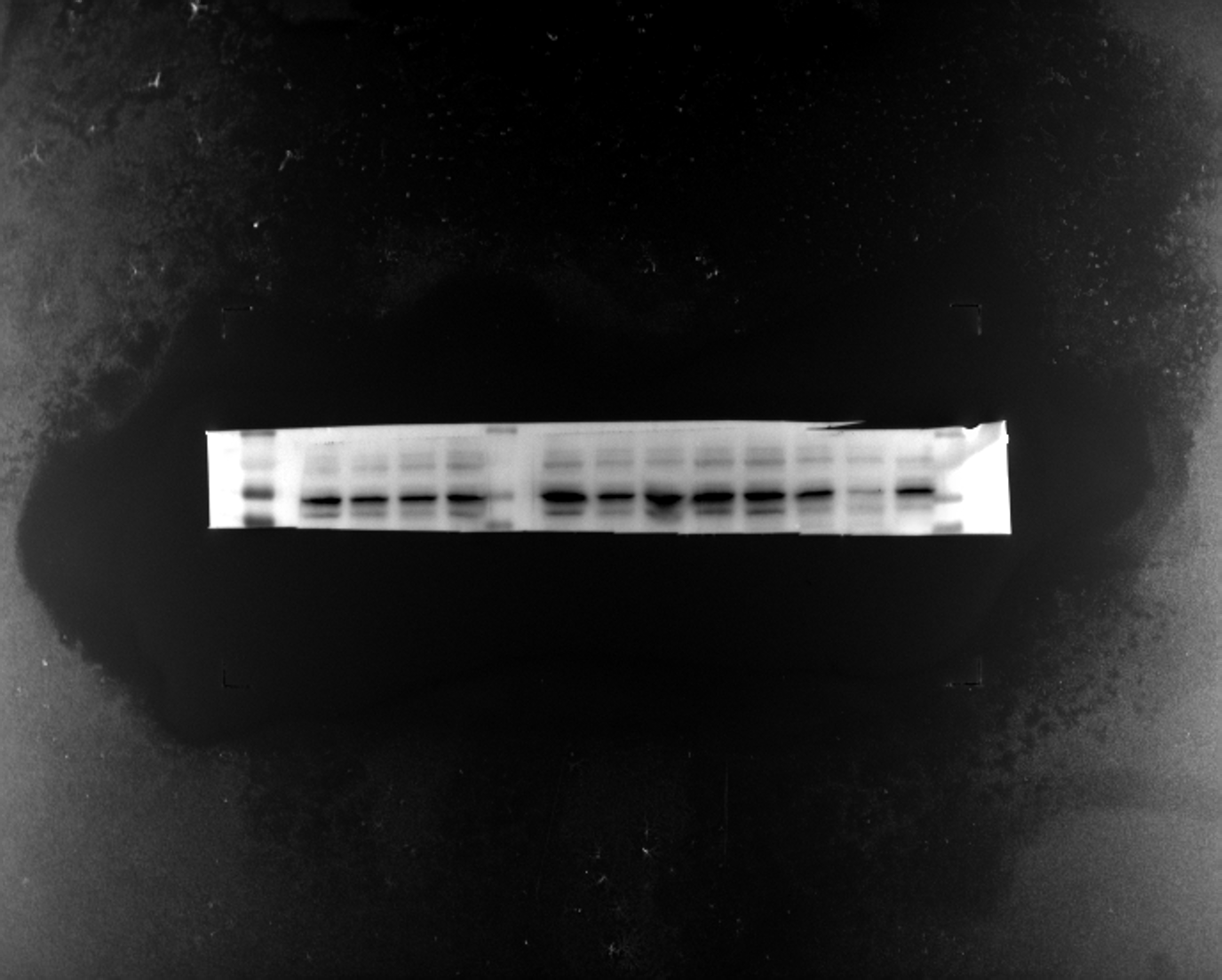


AKT





P-AKT

Figure 4

IL-1β


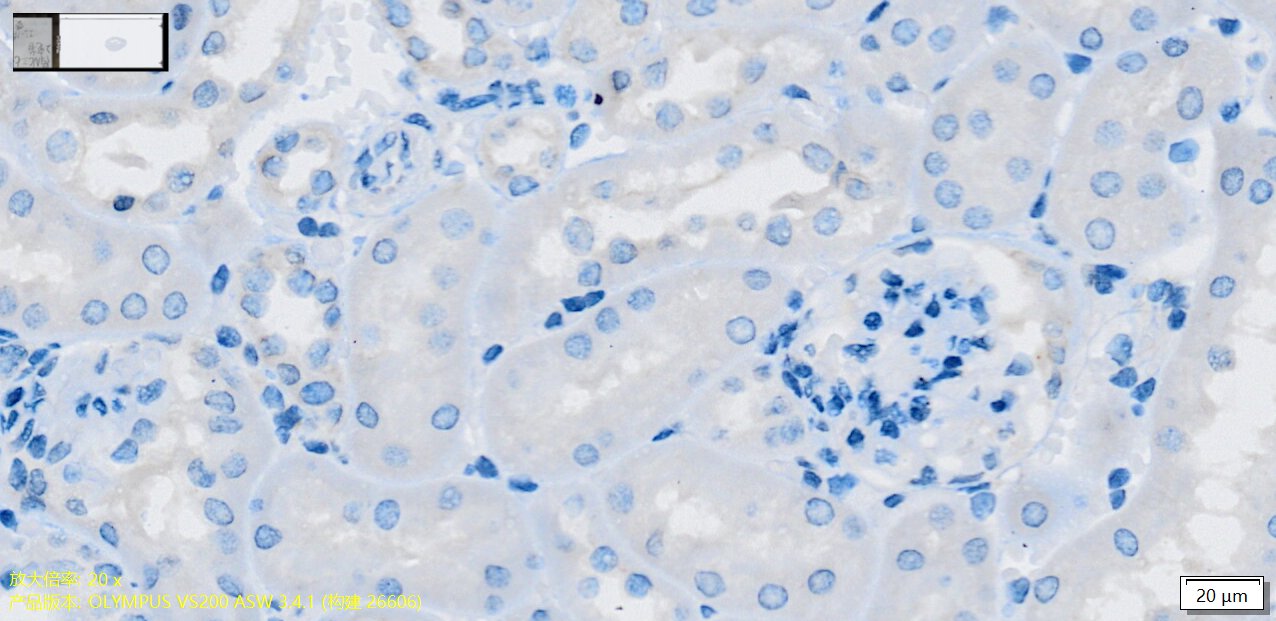


Sham


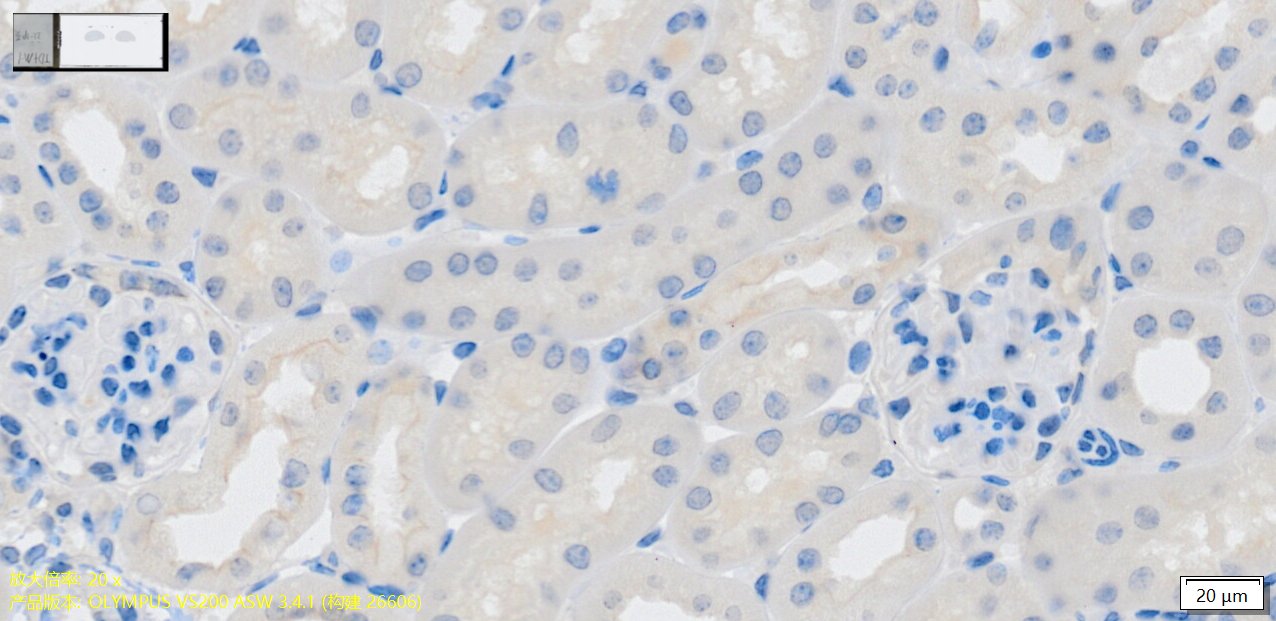


RIRI


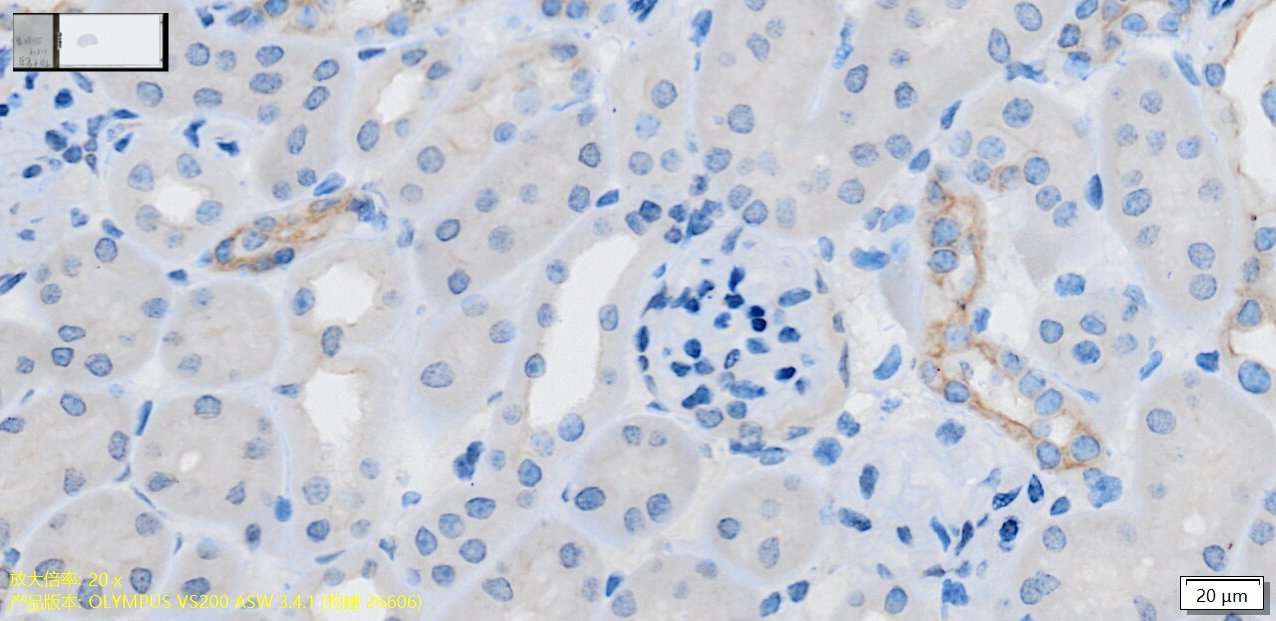


SA-L


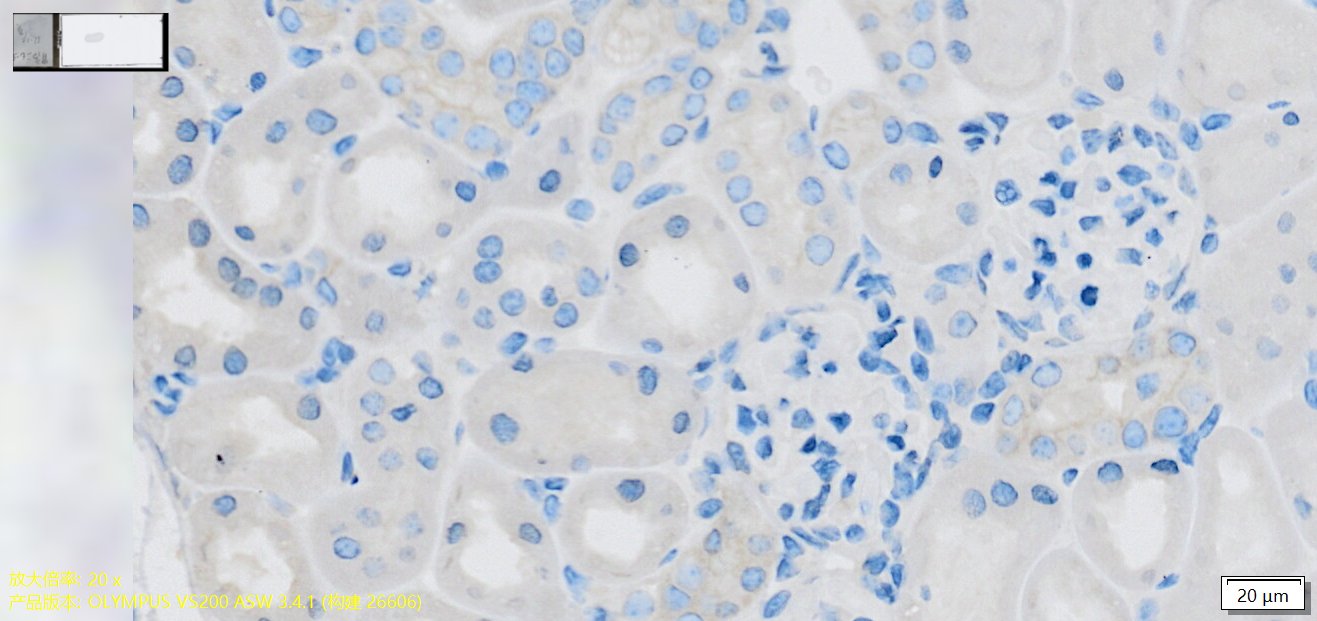


SA-H

Figure 4

NLRP3


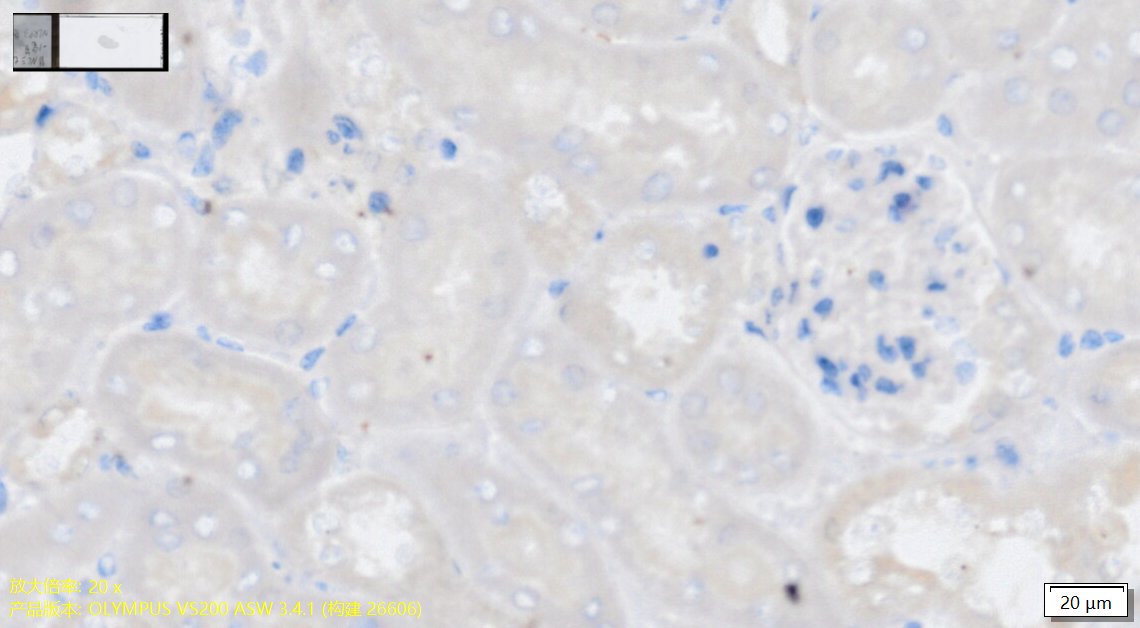


Sham


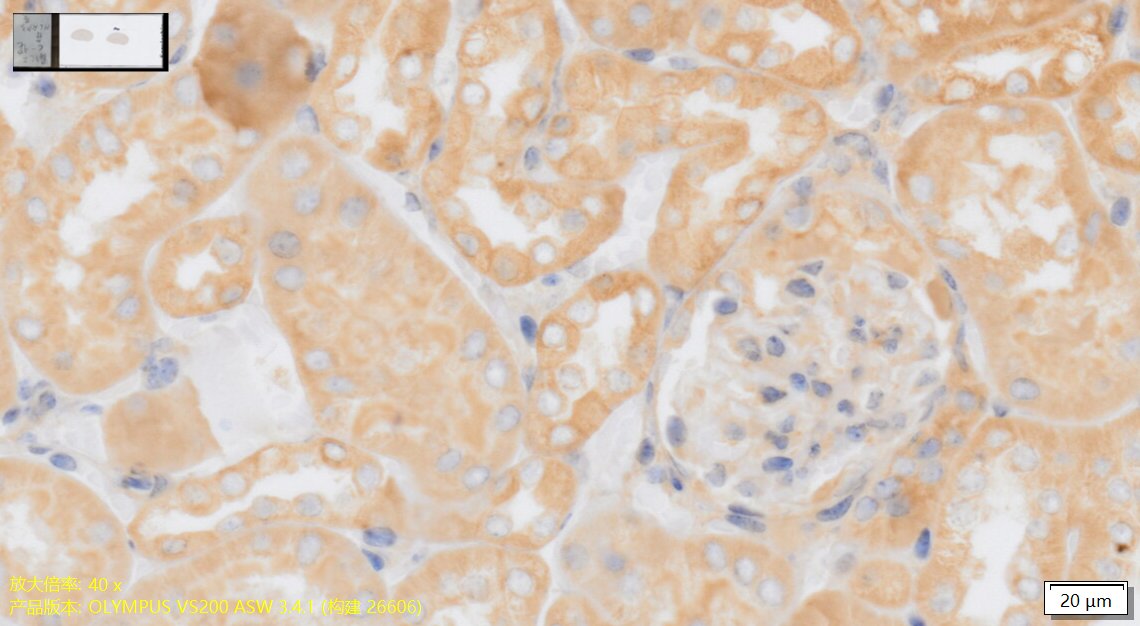


RIRI


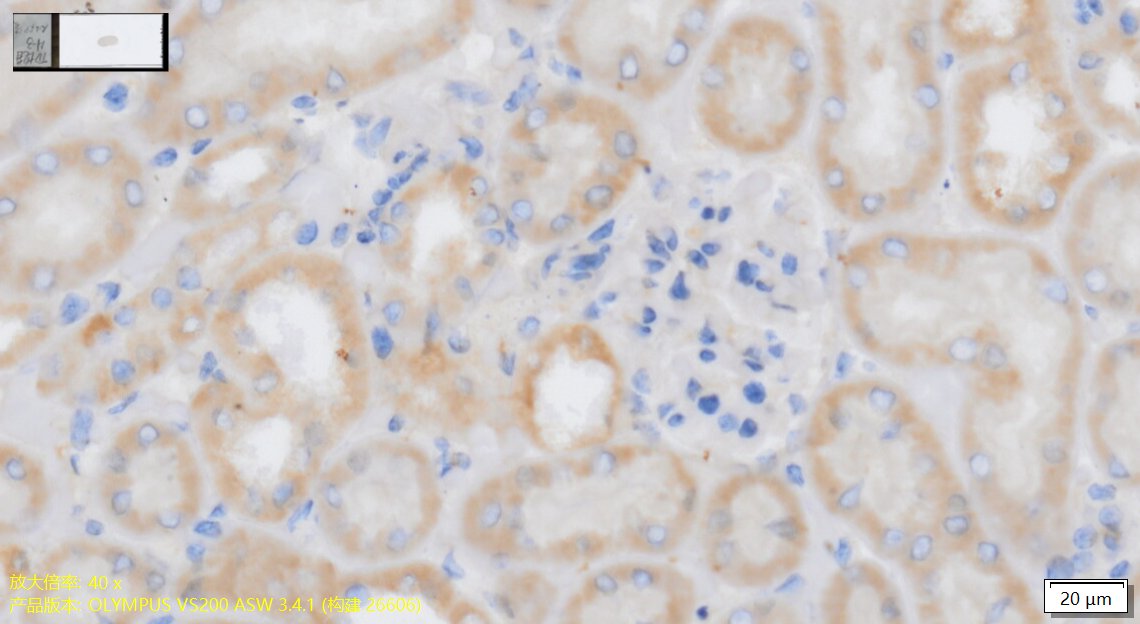


SA-L


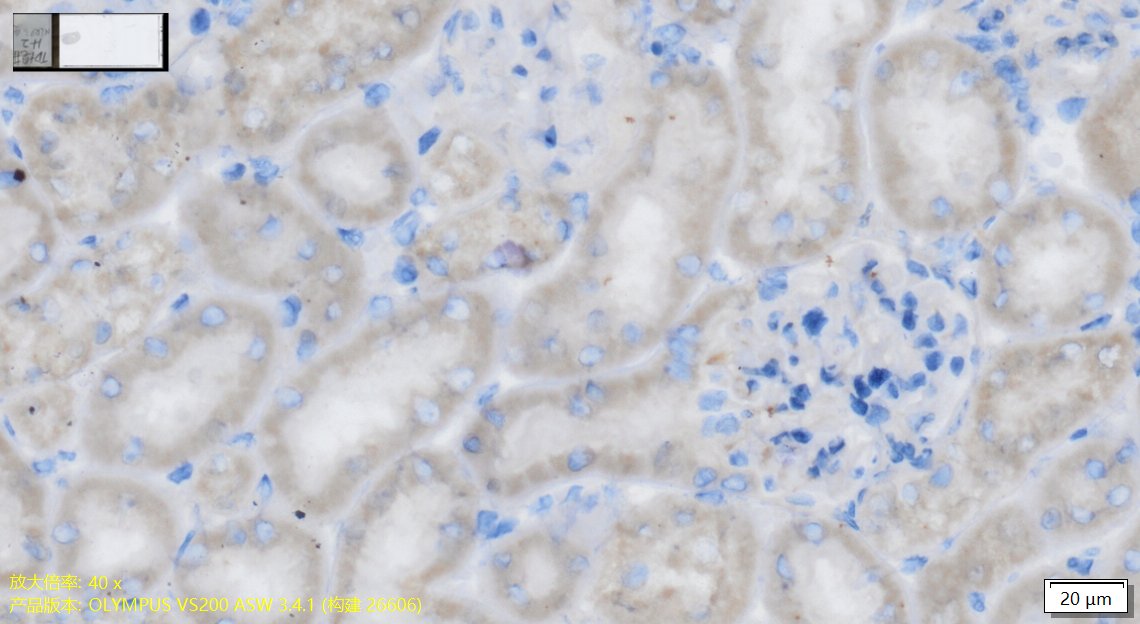


SA-H

Figure 6D


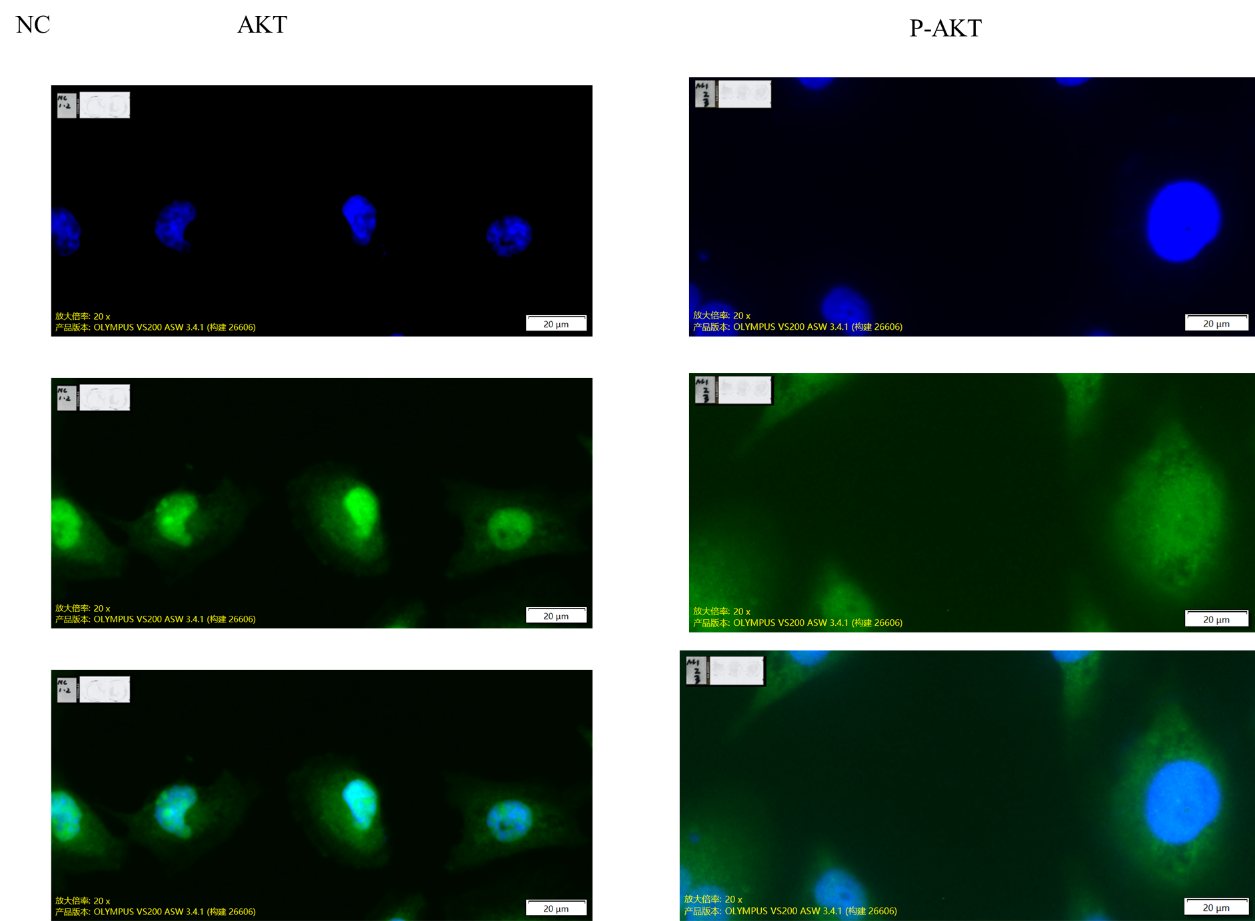


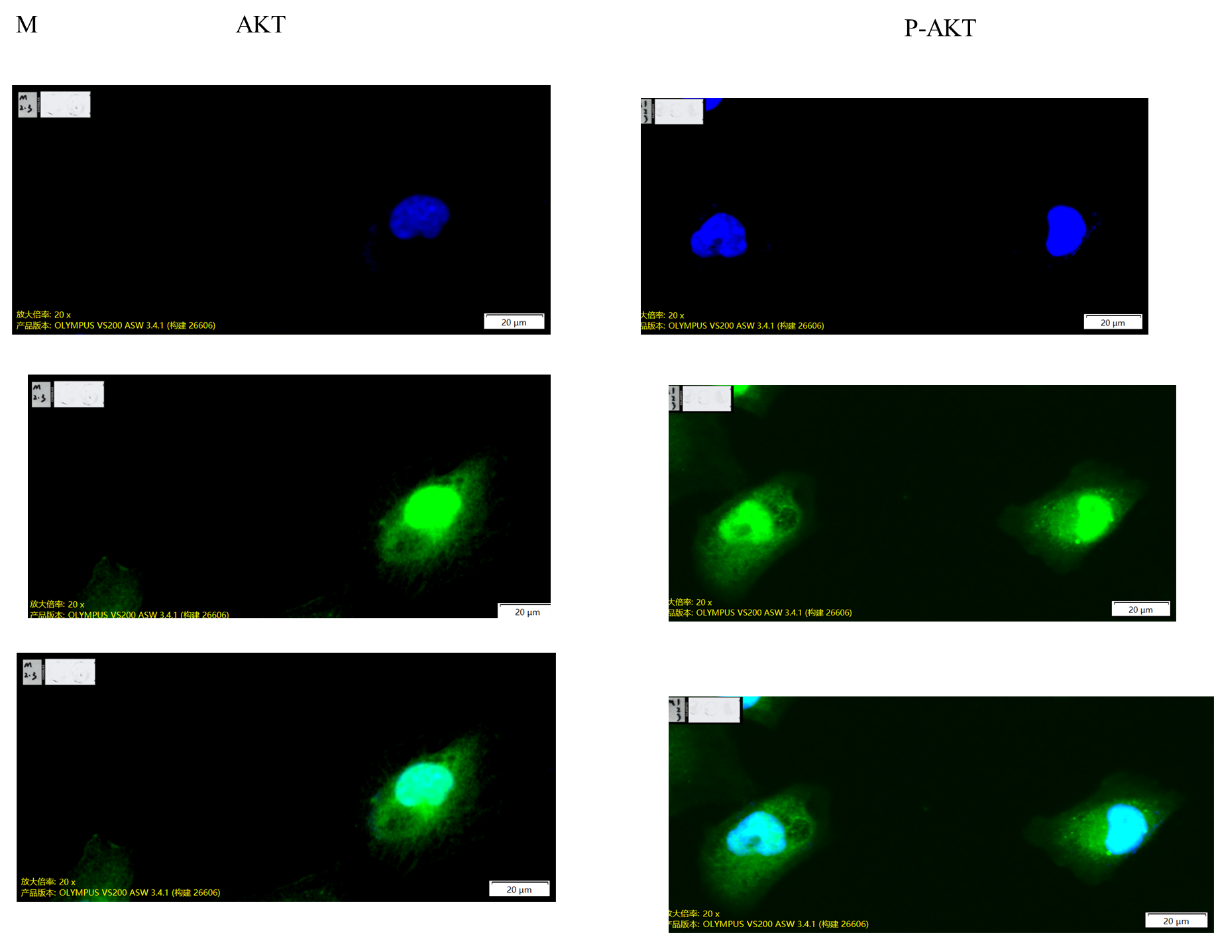


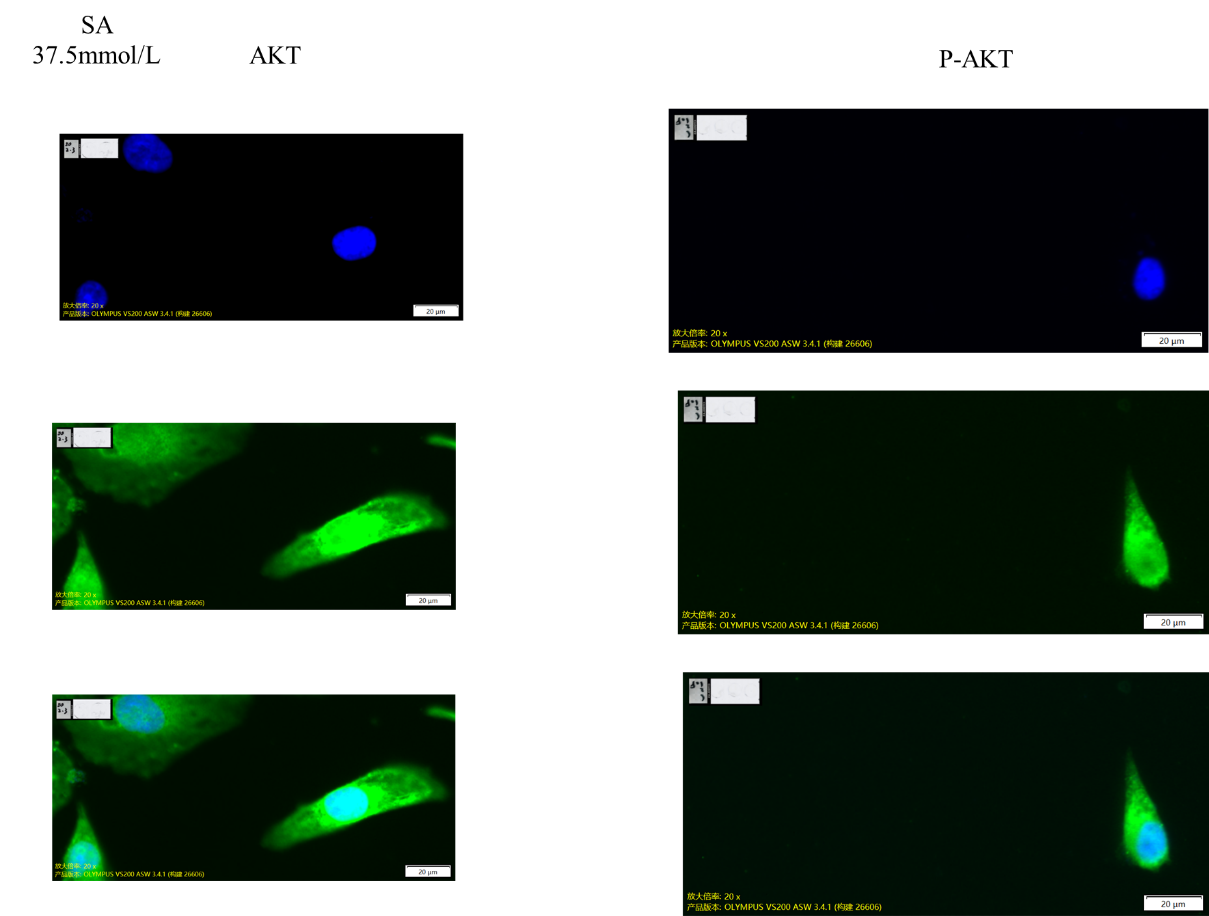


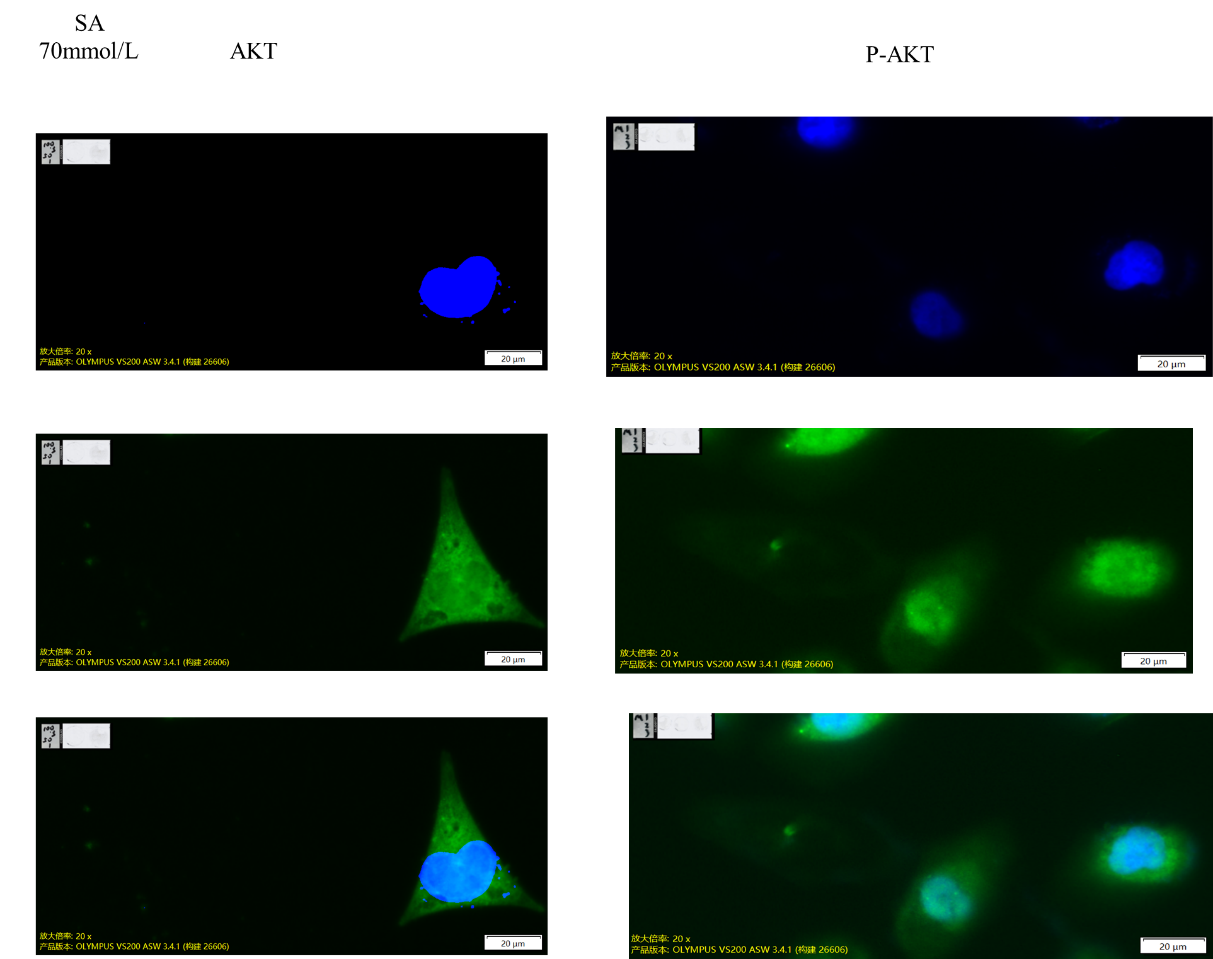


Figure 6E


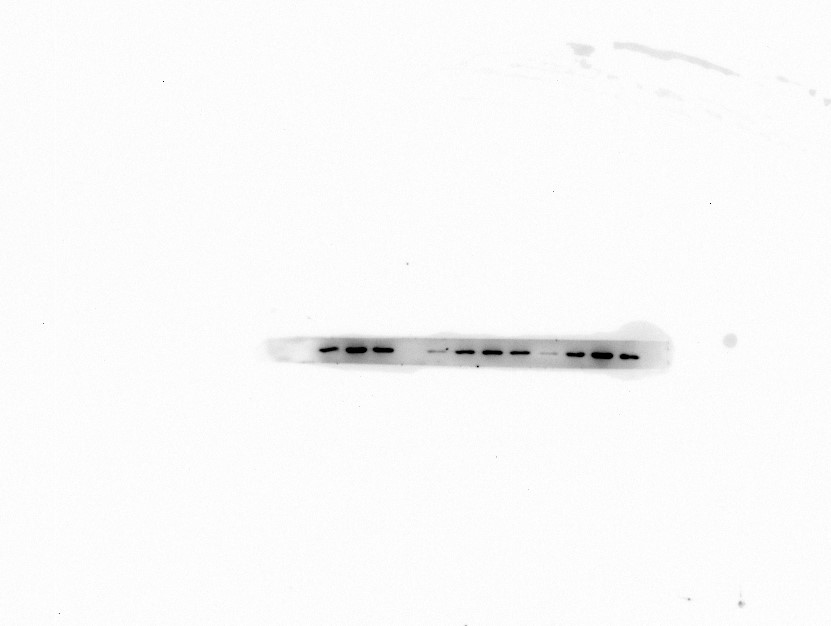


P-AKT


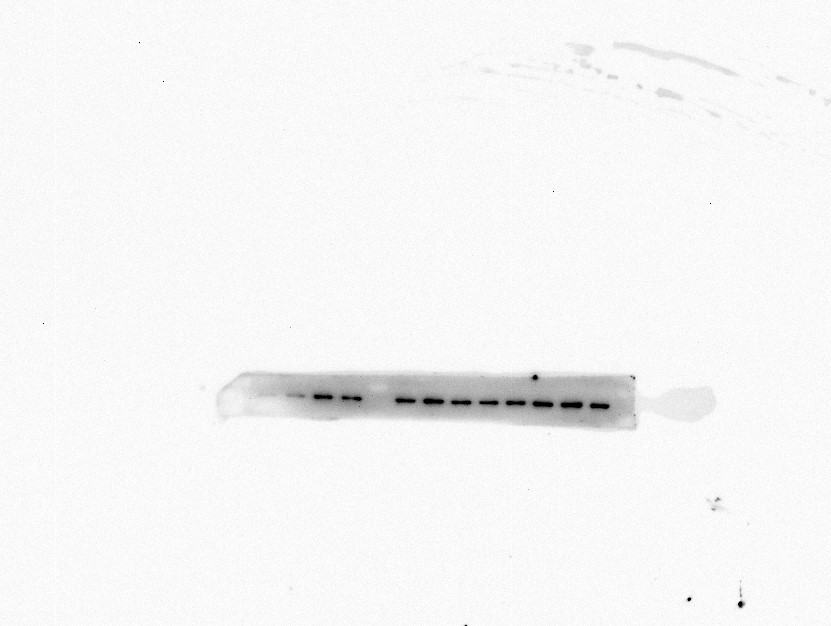


AKT


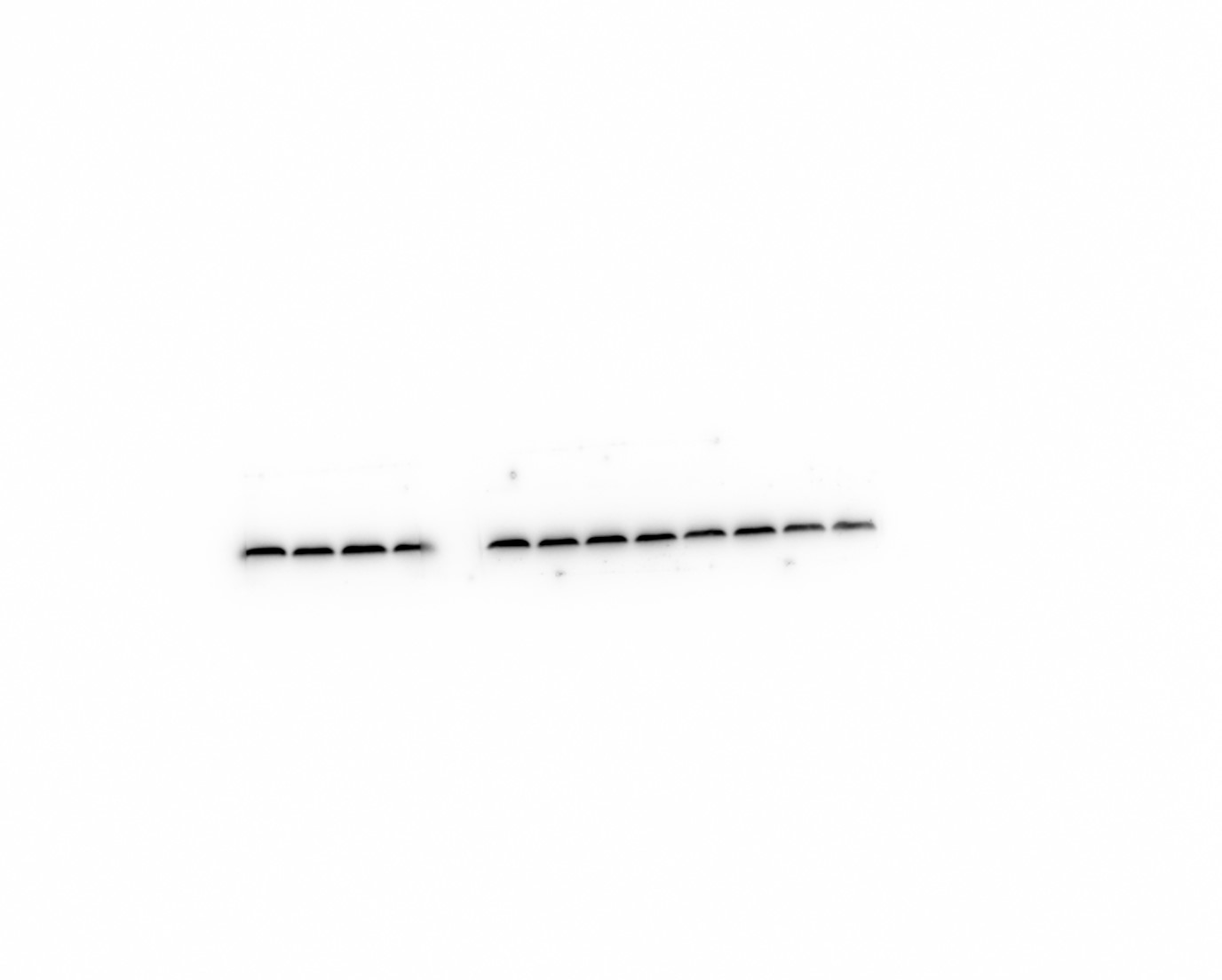


Actin
